# Transcriptomic Profiling Reveals Inflammatory, Fibrotic, and Apoptotic Signatures in a Methionine–Choline-Deficient Diet-Induced Murine Model of Metabolism-Dysfunction-Associated Steatohepatitis

**DOI:** 10.3390/ijms27136033

**Published:** 2026-07-05

**Authors:** Yih-Dih Cheng, Hong-Yi Chiu, Yu-Jen Chiu, Miau-Rong Lee, Shih-Chang Tsai, Jai-Sing Yang

**Affiliations:** 1 Department of Pharmacy, China Medical University Hospital, Taichung 404327, Taiwan; tovis168@gmail.com; 2 School of Pharmacy, College of Pharmacy, China Medical University, Taichung 406040, Taiwan; 3 Department of Pharmacy, Hualien Tzu Chi Hospital, Buddhist Tzu Chi Medical Foundation, Hualien 970, Taiwan; mumu6292@gmail.com; 4 Graduate Institute of Clinical Pharmacy, College of Medicine, Tzu Chi University, Hualien 970, Taiwan; 5 Department of Pharmacy, College of Medicine, Tzu Chi University, Hualien 970, Taiwan; 6 Division of Plastic and Reconstructive Surgery, Department of Surgery, Taipei Veterans General Hospital, Taipei 112201, Taiwan; chiou70202@gmail.com; 7 Department of Surgery, School of Medicine, National Yang Ming Chiao Tung University, Taipei 112304, Taiwan; 8 Department of Biochemistry, China Medical University, Taichung 404, Taiwan; miauwith@gmail.com; 9 Department of Biological Science and Technology, China Medical University, Taichung 406040, Taiwan; 10 Department of Medical Research, China Medical University Hospital, China Medical University, Taichung 404327, Taiwan

**Keywords:** methionine–choline-deficient diet (MCD), metabolic dysfunction-associated steatohepatitis (MASH), RNA sequencing, oxidative stress, liver injury, toxicogenomics

## Abstract

Metabolic dysfunction-associated steatohepatitis (MASH; formerly non-alcoholic steatohepatitis, NASH) is characterized by oxidative stress, inflammatory activation, hepatocellular injury, and progressive liver dysfunction. However, the global transcriptomic landscape underlying stress-induced hepatic injury remains incompletely understood. In this study, we employed a methionine–choline-deficient (MCD) diet-induced murine model to characterize the phenotypic and transcriptomic alterations associated with liver injury. Male C57BL/6J mice were fed either a control or MCD diet, and hepatotoxicity was assessed by survival analysis, body and liver weight measurements, serum alanine aminotransferase (ALT) and aspartate aminotransferase (AST) levels, histopathological examination, RNA sequencing, quantitative real-time PCR (qRT-PCR), and tumor necrosis factor-alpha (TNF-α) enzyme-linked immunosorbent assay (ELISA). MCD feeding markedly reduced survival and body weight while inducing hepatomegaly and significant elevations in serum ALT and AST, indicating severe hepatocellular injury. Histopathological analysis demonstrated hepatic steatosis, hepatocellular ballooning, and lobular inflammation without histological evidence of fibrosis. Transcriptomic profiling revealed extensive gene expression remodeling, characterized by activation of inflammatory pathways, enrichment of MAPK-related signaling, dysregulation of lipid metabolism, suppression of antioxidant defense systems, impairment of cytochrome P450-mediated detoxification, and upregulation of apoptosis-associated genes. qRT-PCR further validated the differential expression of representative genes involved in inflammatory signaling (*Tlr4*, *Nfkb1*, *Nlrp3*, and *Casp1*), MAPK signaling (*Fos*), xenobiotic metabolism (*Cyp4f18*), lipid metabolism (*Apoa4* and *Lpl*), extracellular matrix remodeling (*Mmp12*), and oxidative stress responses (*Sod1* and *Gstp1*). In addition, elevated serum TNF-α levels provided protein-level evidence supporting activation of the TLR4/NF-κB/TNF-α/NLRP3 inflammatory axis. Although fibrosis-associated transcriptional responses were detected, the absence of histological fibrosis suggests transcriptional priming of fibrogenic pathways rather than established fibrogenesis. Collectively, these findings provide a transcriptomic framework linking oxidative stress, impaired detoxification, inflammatory activation, and stress-responsive signaling to MCD-induced hepatic injury. The MCD model provides a valuable experimental platform for characterizing hepatic stress-response transcriptomes and for generating hypotheses that can subsequently be evaluated in environmentally relevant toxicological models. Nevertheless, caution should be exercised when extrapolating these findings to obesity-associated human MASLD, as the MCD model lacks key metabolic features of the human disease, including obesity and insulin resistance. Therefore, the present findings should be interpreted primarily as transcriptomic signatures of stress-induced hepatic injury rather than as a direct representation of the pathophysiological processes underlying human obesity-associated MASLD.

## 1. Introduction

Metabolic dysfunction-associated steatotic liver disease (MASLD), formerly known as non-alcoholic fatty liver disease (NAFLD), is one of the most prevalent chronic liver diseases worldwide and encompasses a pathological spectrum ranging from simple steatosis to metabolic dysfunction-associated steatohepatitis (MASH; formerly NASH), a progressive inflammatory phenotype characterized by hepatocellular injury and chronic inflammation [1,2,3]. MASH may further progress to fibrosis, cirrhosis, and hepatocellular carcinoma, ultimately leading to liver-related mortality and imposing a substantial global health burden [4,5,6,7]. MASLD affects approximately 30% of the global adult population and has become the leading cause of chronic liver disease worldwide. Its prevalence varies considerably across geographic regions, with particularly high rates reported in Latin America, the Middle East and North Africa, North America, and several Asian countries [8]. The increasing prevalence of MASLD parallels the global rise in obesity, type 2 diabetes mellitus, and metabolic syndrome. Disease onset and progression are multifactorial and are strongly associated with obesity, insulin resistance, dyslipidemia, sedentary lifestyles, and dietary factors, all of which promote metabolic dysfunction and hepatic lipid accumulation [8,9,10]. Beyond these metabolic determinants, genetic susceptibility, oxidative stress, inflammatory signaling, environmental exposures, and xenobiotic-induced hepatotoxicity have also been implicated in disease progression, underscoring the complex and multifactorial pathogenesis of MASLD [11,12]. Consequently, transcriptomic and toxicogenomic approaches may provide valuable insights into the molecular networks underlying liver injury and stress-induced hepatotoxicity, thereby facilitating a more comprehensive understanding of the pathogenic mechanisms involved in MASLD progression.

Environmental toxicants have been reported to activate oxidative stress, inflammatory signaling, and hepatocellular injury through molecular mechanisms that substantially overlap with those induced by metabolic stress. Exposure to environmental toxicants has been reported to disrupt hepatic redox homeostasis, alter lipid metabolism, and promote inflammatory signaling, thereby facilitating the progression of MASH [13,14,15,16]. Mechanistically, MASH pathogenesis involves a complex network of oxidative stress imbalance, mitochondrial dysfunction, cytokine-mediated inflammation, and hepatocyte apoptosis, all of which are regulated by interconnected signaling pathways, including kinase-dependent stress responses and inflammatory cascades [17,18,19]. Transcriptomic profiling has emerged as a powerful approach for characterizing hepatic stress responses and identifying molecular networks underlying liver injury. In parallel, toxicogenomic analyses have further expanded our understanding of the molecular mechanisms associated with environmentally induced hepatotoxicity, providing valuable insights into shared stress-responsive pathways. However, despite extensive investigations, the global transcriptomic architecture underlying toxic stress-associated MASH progression remains incompletely defined, limiting the identification of effective therapeutic targets [20,21,22,23].

Experimental animal models are essential for mechanistic toxicology studies aimed at dissecting molecular pathways involved in MASH development [24,25]. Among these, the methionine–choline-deficient (MCD) diet is a well-established approach that induces MASH-like pathology through impaired very-low-density lipoprotein synthesis and increased oxidative stress [25,26]. Feeding an MCD diet rapidly triggers hepatic steatosis, inflammatory responses, and hepatocellular injury, recapitulating key histopathological and biochemical features of human MASH and providing a robust platform for studying stress-driven liver toxicity [27,28,29,30,31].

In the present study, we performed comprehensive RNA sequencing (RNA-seq)-based transcriptomic profiling to characterize gene expression alterations in a murine MASH model induced by a methionine-choline-deficient (MCD) diet. By integrating phenotypic assessments, histopathological evaluation, and pathway enrichment analyses, we sought to identify key genetic signatures and stress-responsive regulatory networks underlying MASH progression. Our findings provide a comprehensive overview of the transcriptomic and toxicogenomic responses associated with hepatic injury and stress-induced molecular alterations. These data may facilitate mechanistic investigations of hepatotoxicity and serve as a valuable resource for future studies to validate therapeutic targets in more metabolically representative MASLD models.

## 2. Results

### 2.1. MCD Diet Markedly Reduced Survival in Mice

**Figure 1:**
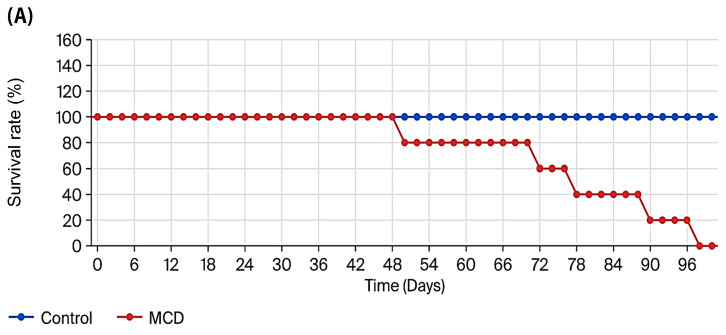

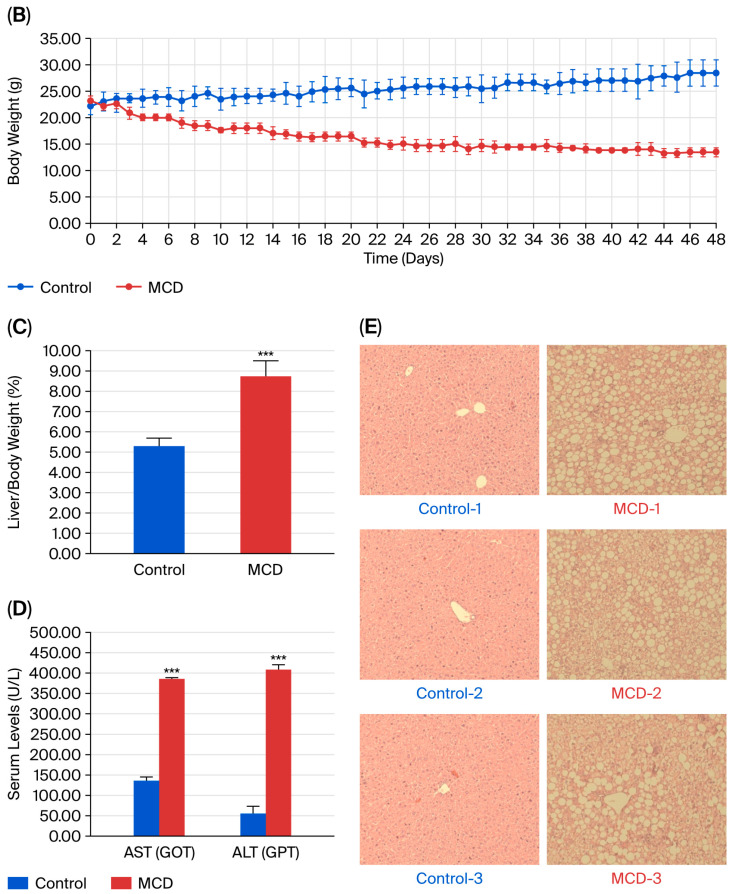
Phenotypic, biochemical, and histopathological characterization of MCD diet-induced liver injury in C57BL/6J mice. (**A**) Kaplan–Meier survival curves of mice fed a control chow diet (blue) or a methionine–choline-deficient (MCD) diet (red) over 96 days (*n* = 5 per group). (**B**) Body weight changes over 48 days in control (blue) and MCD-fed (red) mice. (**C**) Liver-to-body weight ratio at day 48. (**D**) Serum levels of aspartate aminotransferase (AST/GOT) and alanine aminotransferase (ALT/GPT). (**E**) Representative hematoxylin and eosin (HE)-stained liver sections from control (**left** panels) and MCD-fed mice (**right** panels) showing pronounced steatosis, hepatocellular ballooning, and lobular inflammation in the MCD group. Original magnification, ×200. Data are presented as mean ± SD; *** *p* < 0.001.

As shown in Figure 1A, mice fed the MCD diet exhibited a pronounced reduction in survival compared with control animals. Mortality in the MCD group was first observed on day 48, and all MCD-fed mice died by day 96, whereas no mortality occurred in the control group during the experimental period. These findings indicate that prolonged MCD feeding induces severe systemic stress and progressive lethality.

### 2.2. Effects of MCD Diet on Body Weight, Liver Index, and Serum Biochemical Parameters

Mice were sacrificed on day 48 for phenotypic and biochemical evaluation. As illustrated in Figure 1B, MCD-fed mice displayed significantly lower body weights than controls, with an approximate 10% reduction by day 48. Despite overall weight loss, the liver-to-body weight ratio was significantly increased in the MCD group (Figure 1C), indicative of hepatomegaly [32,33]. Serum biochemical analysis demonstrated marked elevations in AST and ALT levels in MCD-fed mice compared with controls (Figure 1D), consistent with hepatocellular injury [34,35,36]. Histological examination (Figure 1E) and MASH activity scoring (Table 1) revealed pronounced steatosis, hepatocellular ballooning, and lobular inflammation without overt fibrosis, resulting in significantly higher MAS values in the MCD group.

### 2.3. Global Transcriptomic Remodeling in MCD-Induced MASH

**Figure 2:**
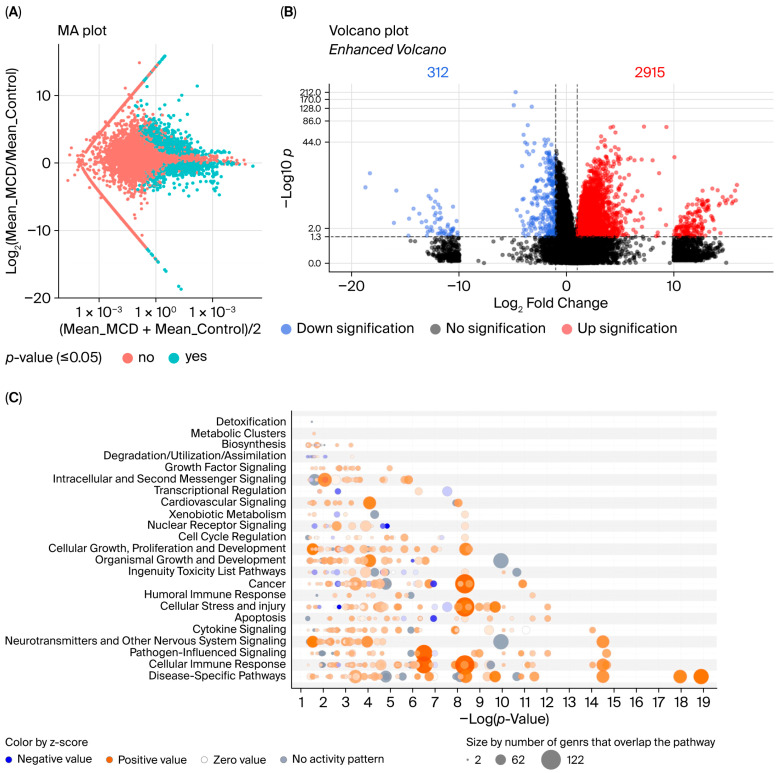
Global transcriptomic remodeling in livers of MCD-fed mice revealed by RNA sequencing. (**A**) MA plot illustrating differential gene expression between MCD-fed and control mice; cyan dots indicate significantly differentially expressed genes (*p* ≤ 0.05), and salmon dots indicate non-significant changes. (**B**) Volcano plot showing 2915 upregulated (red) and 312 downregulated (blue) genes in MCD-fed mice relative to controls (cutoff: |log~2~ fold change| ≥ 1, *p* ≤ 0.05). (**C**) Bubble plot of Ingenuity Pathway Analysis (IPA) categories enriched in MCD-fed mice. Bubble size reflects the number of genes overlapping each pathway; color indicates predicted activation z-score (orange, positive; blue, negative).

**Figure 3:**
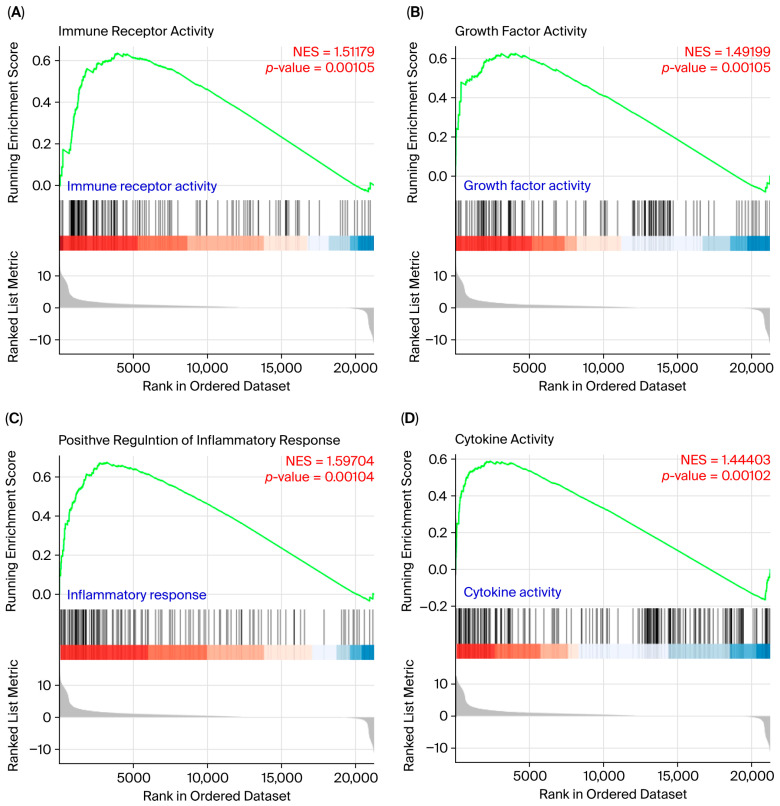
Gene set enrichment analysis (GSEA) of major functional categories altered by MCD feeding. Enrichment plots of the top four significantly enriched gene sets: (**A**) immune receptor activity, (**B**) growth factor activity, (**C**) inflammatory response, and (**D**) cytokine activity. Normalized enrichment scores (NES) and *p*-values are indicated for each gene set. Vertical black lines denote the position of gene set members in the ranked gene list; the green curve represents the running enrichment score.

RNA sequencing analysis revealed substantial transcriptional alterations between control and MCD-fed mice. The MA plot (Figure 2A) demonstrates clear divergence in gene expression patterns, while the volcano plot analysis (Figure 2B) identified 2915 upregulated and 312 downregulated genes in the MCD group (FDR-adjusted *p* < 0.05 and |log2FC| ≥ 1) (Supplementary Table S1). KEGG enrichment analysis highlighted multiple significantly altered pathways (Figure 2C). Gene set enrichment analysis further showed enrichment of immune receptor activity, growth factor signaling, cytokine-mediated pathways, and inflammatory responses in MCD-fed mice (Figure 3), indicating widespread activation of stress-responsive transcriptional programs. The differentially expressed genes were predominantly upregulated, consistent with the strong pro-inflammatory and stress-responsive transcriptional induction elicited by MCD feeding, in which the coordinated activation of inflammatory, MAPK, and apoptosis-related programs outweighs transcriptional repression.

### 2.4. Activation of Early Fibrogenic Signaling-Associated Transcriptional Signatures in MCD-Fed Mice

**Figure 4:**
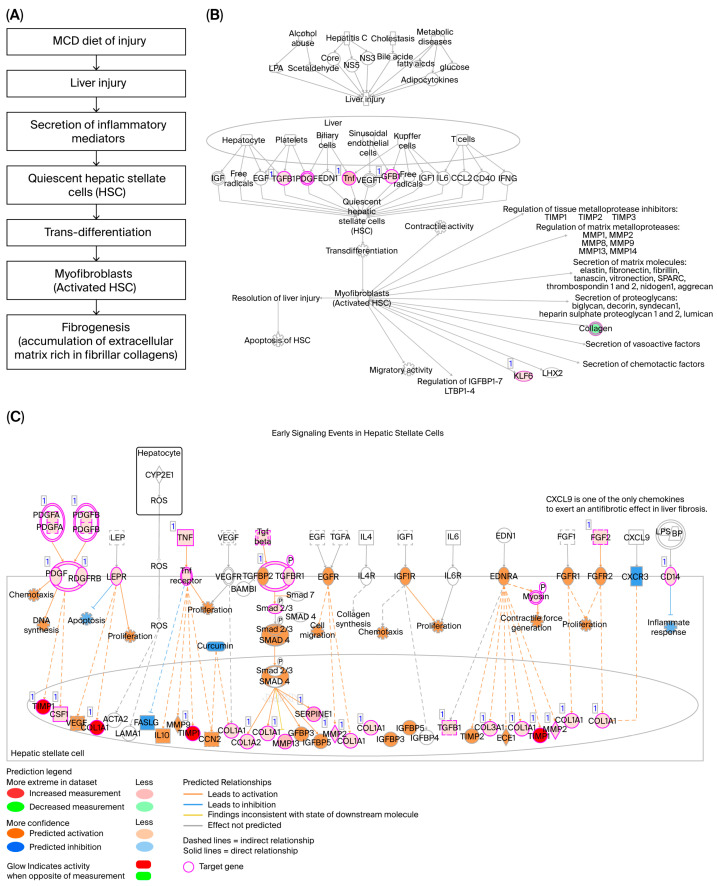
Activation of hepatic stellate cell (HSC)-related fibrogenic signaling pathways in MCD-fed mice identified by IPA. (**A**) Quiescent HSC signaling pathway with differentially expressed genes highlighted in magenta. (**B**) Early signaling events during HSC activation, illustrating transcriptional enrichment of PDGF-, TGFβ-, and EDN1-mediated cascades together with downstream extracellular matrix and collagen-related targets. (**C**) Signaling events in fully activated HSCs (myofibroblasts), showing convergent activation of TLR4, CCR5, NF-κB, and STAT signaling networks. Magenta circles indicate target genes identified as significantly differentially expressed in MCD-fed mice. Color intensity reflects the predicted activation (orange) or inhibition (blue).

Pathway analysis demonstrated enrichment of transcriptional programs associated with early fibrogenic signaling in MCD-fed mice (Figure 4). Transcriptional alterations were observed across multiple stages of hepatic stellate cell (HSC) activation, including quiescent HSC signaling, early activation events, and fully activated HSC pathways. Gene-cluster analysis demonstrated enrichment of extracellular matrix remodeling signatures, including multiple collagen isoforms (COL1A1, COL1A2, COL3A1, COL4A1, COL4A2, COL4A5, COL5A1, COL5A2, COL6A2, COL6A3, COL8A1, COL12A1, COL15A1, and COL20A1) together with matrix regulators (MMP2, MMP13, TIMP1, and SERPINE1), suggesting transcriptional activation of extracellular matrix remodeling programs. Inflammatory and chemokine-related signaling components (CCL5, CCR5, CCR7, CD14, CXCL3, ICAM1, VCAM1, TNF, and TLR4) were concurrently upregulated, reflecting immune-mediated activation of fibrogenic pathways. Growth factor-driven signaling involving the PDGF-TGFβ axis (PDGFA, PDGFB, PDGFRA, PDGFRB, TGFB1, and TGFBR1) further supports progressive HSC activation under toxic stress conditions. In addition, transcriptional regulators and immune signaling mediators (NFKB1, NFKB2, REL, RELB, STAT1, KLF6, IFNGR1, and IL10RA) suggest integration of inflammatory and stress-responsive signaling networks (Figure 4B,C). Detailed gene-level information and full pathway datasets are provided in Supplementary Table S2. Collectively, these findings demonstrate activation of early fibrosis-associated transcriptional programs despite the absence of histological evidence of fibrosis, suggesting transcriptional priming of fibrogenic responses rather than established fibrogenesis.

### 2.5. Dysregulation of Inflammatory, Oxidative Stress, and Lipid Metabolism Pathways

**Figure 5:**
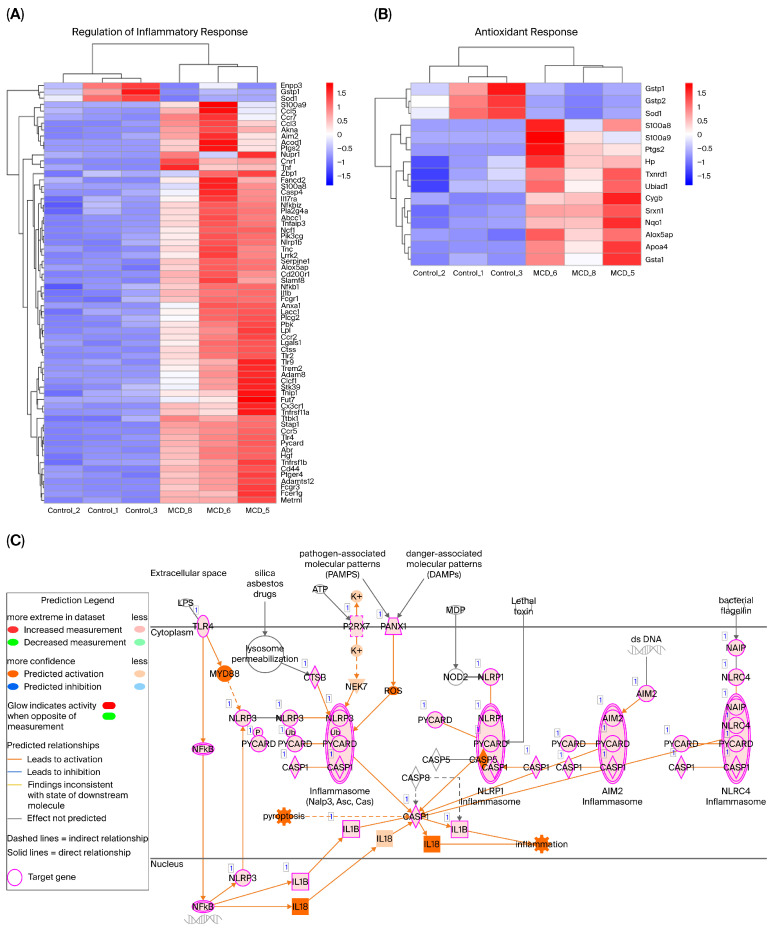
Transcriptomic analysis of inflammatory and antioxidant responses in MCD-induced MASH. (**A**) Heatmap and hierarchical clustering of differentially expressed genes involved in regulating the inflammatory response. (**B**) Heatmap and hierarchical clustering of antioxidant response-related genes. Upregulated genes are shown in red and downregulated genes in blue (log~10~-transformed values; scale bar). (**C**) IPA-generated network analysis illustrating activation of inflammasome signaling cascades (NLRP3, NLRP1, AIM2, and NLRC4 inflammasomes) in MCD-fed mice. Magenta circles indicate target genes identified in this study.

**Figure 6:**
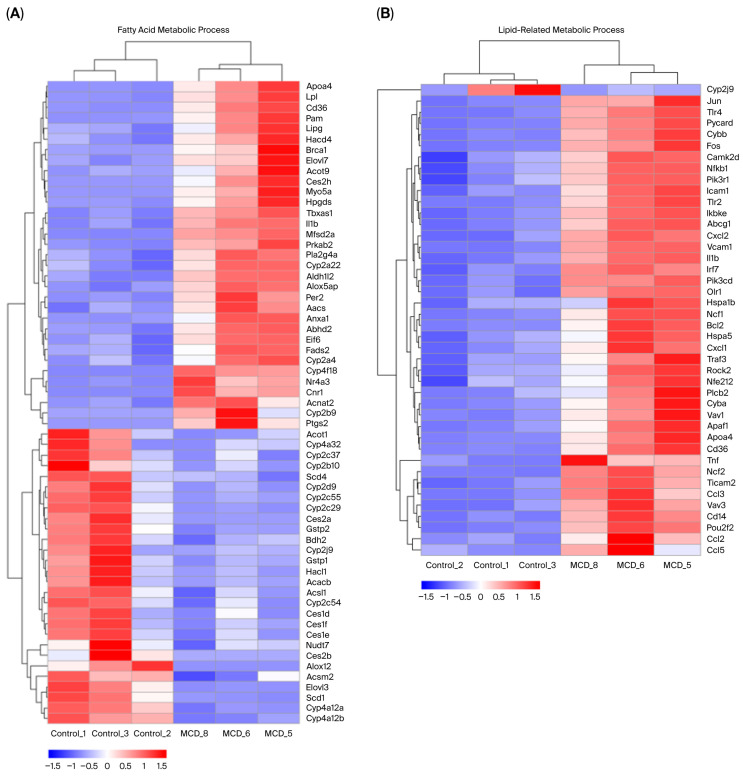

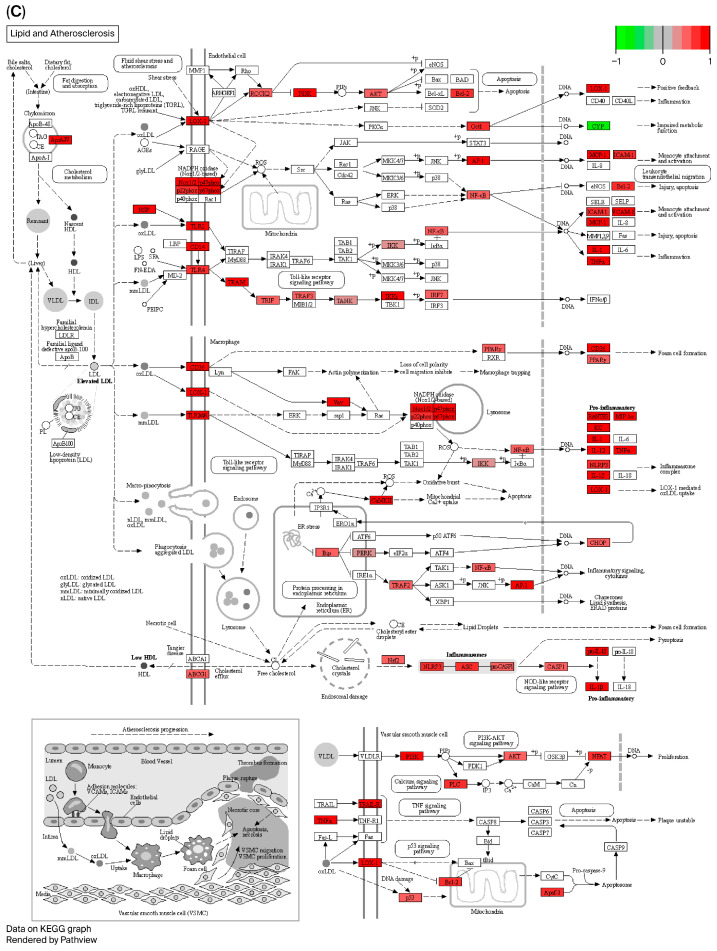
Transcriptomic alterations in fatty acid and lipid metabolism pathways. (**A**) Heatmap of differentially expressed genes involved in fatty acid metabolic processes. (**B**) Heatmap of genes related to lipid-associated metabolic pathways. (**C**) KEGG pathway map of “Lipid and atherosclerosis” highlighting upregulated genes in red. Heatmap color scale represents log~10~-transformed expression values (red, upregulated; blue, downregulated) (available at https://www.kegg.jp, 27 June 2023).

Hierarchical clustering revealed clear segregation between experimental groups based on inflammatory and antioxidant gene signatures (Figure 5A,B). Pro-inflammatory genes were predominantly upregulated in MCD-fed mice, whereas genes associated with antioxidant defense were downregulated. Pathway mapping further illustrated that inflammatory signaling networks (Figure 5C) and lipid metabolic pathways (Figure 6B) were markedly dysregulated. KEGG analysis of lipid metabolism and atherosclerosis pathways (Figure 6C) revealed enrichment of pro-inflammatory mediators and concurrent downregulation of cytochrome P450 family genes, suggesting impaired detoxification capacity. Detailed gene-level information and full pathway datasets are provided in Supplementary Table S2.

### 2.6. Activation of Kinase Signaling, MAPK Pathways, and Apoptosis-Related Gene Networks

**Figure 7:**
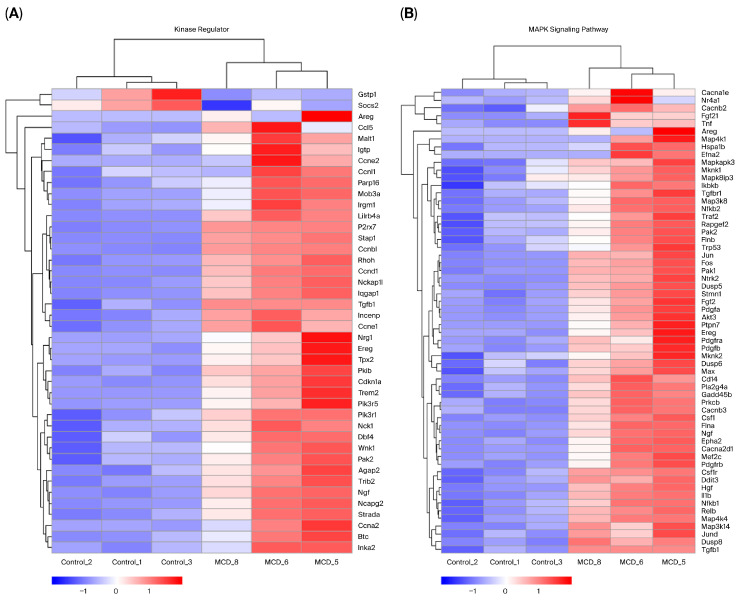

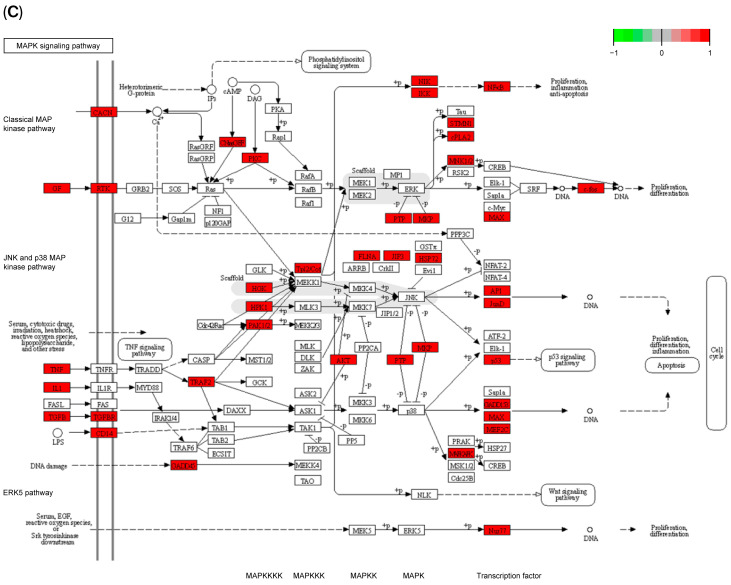
Activation of kinase regulatory and MAPK signaling pathways in MCD-fed mice. (**A**) Heatmap of differentially expressed genes involved in kinase regulatory networks. (**B**) Heatmap of genes associated with the MAPK signaling pathway. (**C**) KEGG pathway map of MAPK signaling, including the classical Ras-Raf-MEK-ERK axis, JNK and p38 MAPK pathways, and ERK5 pathway, with upregulated components highlighted in red (available at https://www.kegg.jp, 27 June 2023).

**Figure 8:**
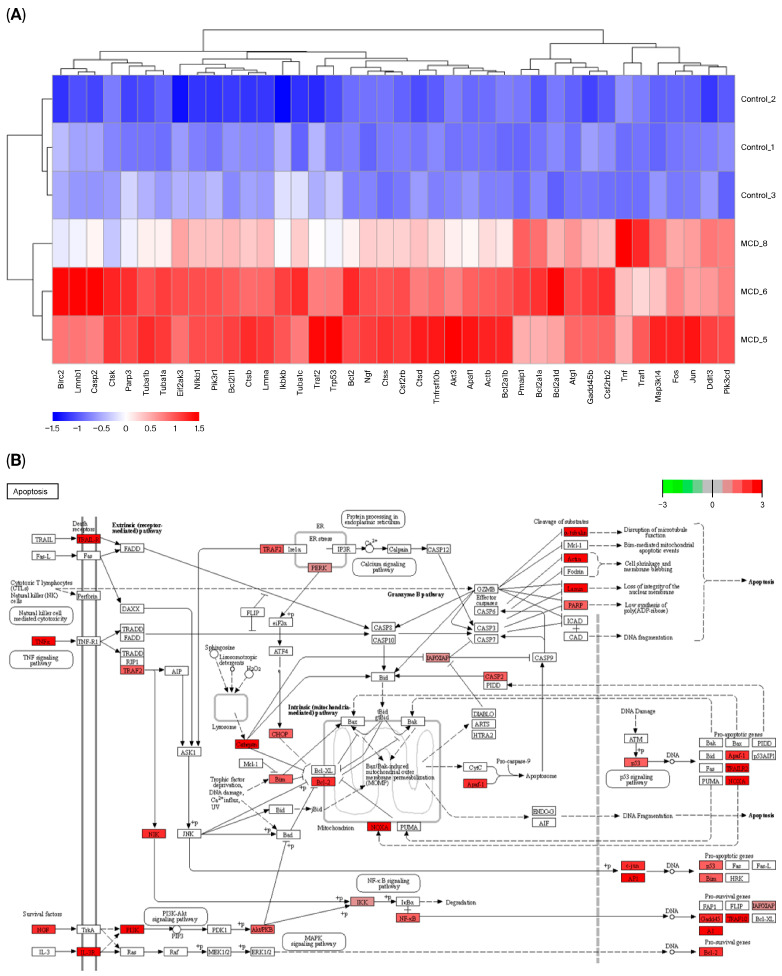

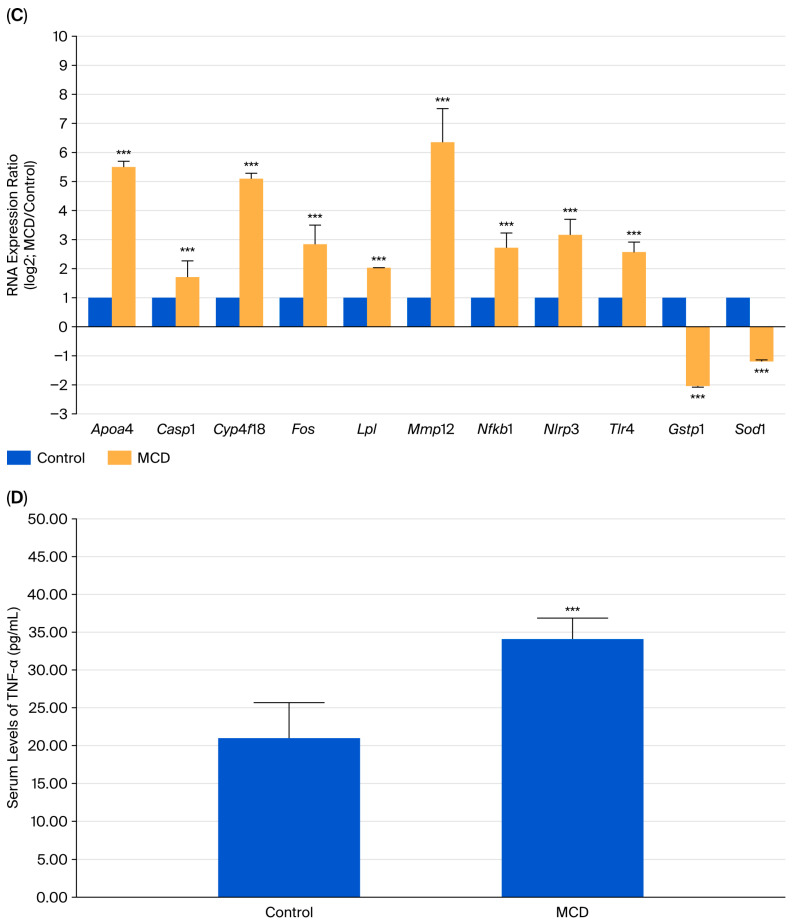
Activation of apoptosis-related signaling networks and qRT-PCR validation of selected genes. (**A**) Heatmap of differentially expressed genes involved in apoptotic regulation in livers of control and MCD-fed mice. (**B**) KEGG pathway map of apoptosis signaling, illustrating activation of both the extrinsic (death receptor-mediated) and intrinsic (mitochondria-mediated) pathways; upregulated genes are highlighted in red (available at https://www.kegg.jp, 27 June 2023). (**C**) Validation of selected differentially expressed genes by quantitative real-time PCR. Relative mRNA expression levels (log2 fold change; MCD/control) of Apoa4, Casp1, Cyp4f18, Fos, Lpl, Mmp12, Nfkb1, Nlrp3, Tlr4, Sod1, and Gstp1 in control (blue) and MCD-fed (yellow) mice. (**D**) Serum tumor necrosis factor-alpha (TNF-α) concentrations in control and MCD-fed mice. Data are presented as mean ± SD; *** *p* < 0.001.

Analysis of kinase regulatory pathways (Figure 7A) and MAPK signaling (Figure 7B) demonstrates significant transcriptional activation in MCD-fed mice. Upregulated genes were primarily associated with inflammatory signaling and cellular stress responses. Apoptosis-related genes (including Birc2, Lmnb1, Casp2, Parp3, Tuba1b, Tuba1a, Eif2ak3, Nfkb1, Pik3r1, Bcl2l11, Ctsb, Lmna, Ikbkb, Tuba1c, Traf2, Trp53, Bcl2, Ngf, Ctss, Csf2rb, Ctsd, Tnfrsf10b, Akt3, Apaf1, Actb, Bcl2a1b, Pmaip1, Bcl2a1a, Bcl2a1d, Actg1, Gadd45b, Csf2rb2, Tnf, Traf1, Map3k14, Jun, Ddit3, and Pik3cd) were consistently upregulated (Figure 8A). KEGG pathway analysis confirmed enrichment of apoptotic signaling cascades (Figure 8B), indicating enhanced hepatocellular apoptosis following MCD feeding. Detailed gene-level information and complete pathway datasets are available in Supplementary Table S2.

### 2.7. Validation of RNA-Seq Data and Identification of the TLR4/NF-κB/NLRP3 Inflammatory Axis

To further validate the RNA-seq results, representative genes involved in multiple biologically relevant pathways were selected for quantitative real-time PCR (qRT-PCR) analysis. Genes associated with inflammatory signaling (*Tlr4*, *Nfkb1*, *Nlrp3*, and *Casp1*), MAPK signaling (*Fos*), xenobiotic metabolism (*Cyp4f18*), lipid metabolism (*Apoa4* and *Lpl*), and extracellular matrix remodeling (*Mmp12*) were significantly upregulated in MCD-fed mice, suggesting activation of fibrosis-associated transcriptional responses, extracellular matrix remodeling, and altered lipid metabolism (Figure 8C). Moreover, the upregulation of *Casp1* further supports the activation of inflammasome-associated cell death pathways (Figure 8C). In contrast, antioxidant-related genes, including superoxide dismutase 1 (Sod1) and glutathione S-transferase pi 1 (*Gstp1*), were significantly downregulated, supporting oxidative stress dysregulation (Figure 8C). Overall, the qRT-PCR results corroborated the RNA-seq findings and strengthened the robustness of the transcriptomic analysis, providing independent validation of representative RNA-seq findings and further supporting the involvement of inflammatory, oxidative stress-related, metabolic, and stress-responsive signaling pathways in MCD-induced liver injury.

To identify the upstream regulatory mechanisms underlying the observed inflammatory transcriptional responses, upstream regulator analysis was performed using Ingenuity Pathway Analysis (IPA). Among the predicted activated upstream regulators, TLR4/NF-κB signaling emerged as a central inflammatory pathway, consistent with the qRT-PCR-confirmed upregulation of *Tlr4*, Nfkb1, Nlrp3, and Casp1. At the protein level, serum TNF-α concentrations were significantly elevated in MCD-fed mice (Figure 8D), providing independent protein-level validation of the inflammatory activation identified by RNA sequencing and qRT-PCR. Collectively, these findings support activation of the TLR4/NF-κB/TNF-α/NLRP3 inflammatory axis and are consistent with a role for inflammasome-associated signaling in the development of MCD-induced hepatic injury and inflammatory progression.

## 3. Discussion

Although the methionine–choline-deficient (MCD) diet is among the most widely used dietary triggers of MASH-like injury, the mechanistic chain linking its initiating metabolic insult to the genome-wide transcriptional response it provokes remains only partially resolved [38,39]. The integrative analysis presented here allows reconstruction of a plausible mechanistic framework linking these events. Methionine and choline restriction simultaneously impairs hepatic very-low-density lipoprotein assembly and depletes the principal methyl-donor and glutathione-precursor pools, so that lipid which would normally be exported is instead retained and progressively oxidized within the hepatocyte. Viewed in this light, the steep loss of survival and body weight together with hepatomegaly and the marked elevation of serum ALT and AST (Figure 1) are best interpreted not as a set of independent toxic endpoints but as the convergent readout of a single upstream lesion: a hepatocyte burdened with substrate it can neither export nor safely detoxify. The fact that this injury developed in the context of weight loss rather than adiposity is mechanistically informative, because it localizes the primary insult to intracellular redox and methylation stress rather than to systemic metabolic overload [38,39]. The histological triad of steatosis, hepatocellular ballooning, and lobular inflammation in the absence of bridging fibrosis (Table 1) further places the model at an early, pre-fibrotic stage of disease, and this temporal positioning is essential for interpreting the transcriptomic signatures discussed below as molecular antecedents of, rather than evidence for, established structural remodeling [40]. From a mechanistic standpoint, the early divergence in survival and biochemistry also implies that the transition from tolerable steatosis to injurious steatohepatitis is governed less by the absolute quantity of stored lipid than by the residual capacity of the hepatocyte to buffer the oxidative consequences of that storage.

A central interpretive question raised by our data is why an extensive collagen- and extracellular-matrix-remodeling transcriptional program (Figure 4) coexists with histologically undetectable fibrosis. We interpret this dissociation as the signature of a particular biological state, transcriptional priming of hepatic stellate cells (HSCs), rather than as a discrepancy between measurement modalities. Early toxic and inflammatory stress is well recognized as licensing HSC transactivation before any measurable matrix deposition [41,42]. Consistent with such priming, the enrichment we detected spans the entire activation trajectory, from quiescent through early to transcriptional signatures characteristic of activated HSCs, and tightly couples a PDGF–TGFβ growth-factor axis to NF-κB- and STAT-linked inflammatory mediators (Figure 4B,C). This co-enrichment suggests that the inflammatory milieu captured by our immune-receptor, cytokine, and growth-factor signatures (Figure 3) is itself the proximate signal that licenses fibrogenic transcription, so that inflammation and fibrogenic commitment may be mechanistically continuous rather than temporally separable events [43,44]. The selective induction of *Apoa4*, *Mmp12*, and *Lpl* (Figure 8C) is consistent with this framework: because hepatic *Lpl* in MASH is induced predominantly within stellate rather than parenchymal cells, its upregulation here most plausibly reflects a stress-responsive lipid-remodeling state in the activating mesenchymal compartment rather than the obesity-linked induction driven by free fatty acids, leptin, and interleukin-6 that characterizes metabolic MASH [45]. The model, therefore, appears to capture the molecular commitment to fibrogenesis that anticipates and helps explain the eventual emergence of overt matrix deposition, even though that endpoint is not yet structurally realized in the present cohort.

The coordinated transcriptional suppression of antioxidant and xenobiotic-handling capacity offers a possible mechanistic explanation for why this injury may be potentially self-amplifying. Depletion of the choline- and methionine-dependent glutathione supply is expected to lower the threshold at which reactive oxygen species damage membrane lipids and proteins, and our data show precisely this vulnerability being encoded at the transcriptional level: *Sod1* and *Gstp1*, which together dismutate superoxide and conjugate the electrophilic by-products of lipid peroxidation, were concordantly downregulated by both RNA-seq and qRT-PCR (Figure 8C) [46,47]. The parallel suppression of cytochrome P450 family members, including the qRT-PCR-validated *Cyp4f18* (Figure 8C), suggests that the same stress simultaneously erodes phase-I detoxification, so that bioactive lipid mediators and xenobiotic intermediates are cleared less efficiently at exactly the moment when oxidative load is rising [48,49]. Superimposed on the lipid-metabolic dysregulation evident in Figure 6, this convergence is consistent with a feed-forward circuit in which impaired lipid export, defective detoxification, and a reduced antioxidant reserve reinforce one another [50,51]. The directionality of these changes is itself diagnostic: a hepatocyte that downregulates its first-line antioxidant and detoxification enzymes while accumulating oxidizable substrate is, in effect, lowering its own injury threshold, which helps to explain why the biochemical and histological damage in this model is disproportionate to the relatively modest steatotic load. Collectively, these findings suggest that oxidative stress, P450 suppression, and lipid dysregulation should not be read as three separate findings but as one coordinated collapse of hepatic redox homeostasis—consistent with the multiple-hit paradigm in which the second and subsequent hits may be generated internally by the injured hepatocyte rather than supplied from without [52,53].

Kinase signaling represents a mechanistic relay that converts this metabolic and redox stress into a committed inflammatory and apoptotic outcome. The pronounced activation of MAPK and broader kinase-regulatory networks (Figure 7), corroborated by the induction of the immediate-early effector Fos (Figure 8C), is consistent with MAPK cascades functioning as stress sensors that decode oxidative and cytokine inputs into transcriptional programs governing the balance between survival and death [54,55,56]. That the same livers display coordinated enrichment of apoptotic regulators spanning both the extrinsic death-receptor and intrinsic mitochondrial arms (Figure 8A,B) indicates that this signaling does not merely accompany injury but actively biases hepatocytes toward apoptotic execution [57,58,59,60,61]. Because apoptotic and stressed hepatocytes release damage-associated molecular patterns that further engage innate immune receptors, kinase-driven cell death and the inflammatory activation described above constitute a reciprocal, self-reinforcing circuit rather than a linear sequence. In mechanistic terms, oxidative imbalance, kinase-mediated stress signaling, and apoptotic commitment therefore represent successive and mutually amplifying nodes of a single injury cascade, furnishing a coherent route from the initiating metabolic lesion to progressive hepatocyte loss and the secondary recruitment of inflammatory effectors.

These individual axes ultimately converge on an important integrating hub: the TLR4/NF-κB/TNF-α/NLRP3 inflammasome circuit. Upstream-regulator analysis nominated TLR4/NF-κB as a predicted master activator, and this prediction was substantiated at both transcript and protein levels, with concordant induction of *Tlr4*, *Nfkb1*, *Nlrp3*, and *Casp1* (Figure 8C) and a significant rise in circulating TNF-α (Figure 8D). The mechanistic significance of this hub is that it can be engaged directly by the very upstream events documented here: peroxidized lipids and oxidative by-products are established endogenous priming signals for TLR4 and for NLRP3 inflammasome assembly. Through this dependency, the redox collapse, the inflammatory transcriptome, and the *Casp1*-dependent inflammasome output (Figure 8C) become causally linked rather than merely coincident. Importantly, this positions the inflammasome not at the apex but at the integrating midpoint of the cascade, receiving redox- and lipid-derived priming signals from upstream while dispatching cytokine and cell-death outputs downstream, which is why interventions targeting this interface would be expected to attenuate several otherwise distinct injury readouts simultaneously. Positioned in this manner, TLR4/NF-κB/NLRP3 functions as the node at which impaired detoxification and lipid peroxidation are translated into amplified cytokine production and into pyroptotic and apoptotic signaling, providing a unifying explanation for the breadth of the inflammatory response observed across our datasets and identifying a mechanistically tractable point of intervention situated upstream of multiple downstream injury programs.

From a toxicogenomic perspective, the principal value of the present findings lies not merely in identifying differentially expressed genes but in characterizing a coordinated hepatic stress-response signature. The molecular alterations observed in this study—including innate immune activation, oxidative stress imbalance, suppression of cytochrome P450-mediated detoxification, and activation of kinase- and apoptosis-related signaling- are broadly consistent with transcriptomic responses reported in environmentally induced hepatotoxicity, including exposure to particulate matter, polychlorinated biphenyls, per- and polyfluoroalkyl substances, and endocrine-disrupting chemicals [62,63]. The convergence of dietary methyl-donor deficiency and chemically diverse hepatotoxic insults on common inflammatory, redox, and detoxification pathways suggests that these molecular responses may represent conserved stress-response mechanisms rather than MCD-specific phenomena. Accordingly, the present findings support the utility of the MCD model as an experimental platform for investigating early molecular events associated with hepatic stress and for generating mechanistic hypotheses that can be further evaluated in environmentally relevant toxicological models. The mechanistic interpretation of the present findings should be considered within the biological context of the MCD model. Because this model induces hepatic injury in the absence of obesity and insulin resistance, the identified transcriptomic signatures primarily reflect stress-induced liver injury rather than the full spectrum of human obesity-associated MASLD. Future studies employing metabolically relevant models, such as Western diet-, AMLN diet-, or genetically induced MASLD models, will be necessary to determine whether these molecular signatures are conserved across different pathogenic contexts and to further establish their translational relevance.

Because MCD feeding induces weight loss and preserves insulin sensitivity, the cascade we describe is driven by intrahepatic methylation and redox stress rather than by the systemic adiposity, insulin resistance, and dyslipidemia that initiate human MASLD [38,39]. The pathways identified should therefore be read as the injury-execution machinery that operates downstream of, and is largely shared with, metabolic disease, rather than as a model of its upstream metabolic triggers. For the same reason, the fibrogenic signatures are best interpreted as transcriptional priming rather than established fibrogenesis, since their functional consequence—quantifiable matrix deposition—had not yet been histologically expressed in our cohort. Recognizing these constraints also clarifies which therapeutic inferences are reasonable: agents that blunt the redox–inflammasome relay should remain relevant across metabolic and toxic etiologies, whereas conclusions regarding obesity-specific or insulin-dependent mechanisms cannot be drawn from this system and must be deferred to complementary models. Far from undermining the mechanistic account, these limitations specify precisely where it applies, indicating how, once engaged, oxidative, inflammatory, and apoptotic programs become wired together in the injured liver, while deliberately leaving the metabolic initiation of that injury to be modeled in obesity- and insulin-resistance-competent systems.

Taken together, the transcriptomic, qRT-PCR, and ELISA evidence assembles into a coherent working model in which impaired lipid export and methyl-donor depletion precipitate a hepatic redox collapse, that redox collapse engages TLR4/NF-κB/NLRP3 inflammasome signaling, and kinase-driven cascades convert the resulting inflammatory state into coordinated hepatocyte apoptosis and fibrogenic priming. By indicating how these nodes are causally connected, rather than simply documenting that each is individually altered, the present study provides a framework on which targeted interventions directed in particular at the redox–inflammasome interface that links upstream metabolic stress to downstream cell death can be rationally designed and subsequently validated in metabolically and toxicologically representative models of human liver disease.

## 4. Materials and Methods

### 4.1. Chemicals and Diets

A standard chow diet (Prolab^®^ RMH2500, 5P14) was obtained from LabDiet, Inc. (St. Louis, MO, USA). An MCD diet (A02082002B) was purchased from Research Diets, Inc. (New Brunswick, NJ, USA) [64]. All reagents used in this study were of analytical grade unless otherwise specified.

### 4.2. Animal Studies

All animal procedures were approved by the Institutional Animal Care and Use Committee of China Medical University (Approval No. CMUIACUC-2020-117) and conducted in accordance with institutional and international guidelines for laboratory animal care. Male, eight-week-old C57BL/6J mice were obtained from the National Laboratory Animal Center (Taipei, Taiwan). Animals were maintained under controlled environmental conditions (12 h light/dark cycle, 23 ± 1 °C, relative humidity 60%) with ad libitum access to food and water.

The in vivo study consisted of two experimental arms. For survival analysis (Supplementary Figure S1A), mice were randomly divided into a control diet group and an MCD diet group (*n* = 5 per group) and monitored for approximately 96 days. For biochemical and transcriptomic analyses (Supplementary Figure S1B), mice were randomly assigned to chow or MCD diets (*n* = 3 per group) and sacrificed on day 48. Body weight, liver weight, serum biochemical parameters, and liver tissues were collected for histological examination, RNA sequencing using formalin-fixed paraffin-embedded (FFPE) liver tissues, pathway analysis, and quantitative PCR validation.

### 4.3. Serum Biochemical and TNF-Alpha Analysis

Serum alanine aminotransferase (ALT) and aspartate aminotransferase (AST) levels were measured using a Fujifilm Dri-Chem NX-500 automated clinical chemistry analyzer (Fujifilm, Tokyo, Japan). Commercial assay slides, including GOT/AST-P III (#3150), GPT/ALT-P III (#3250), and TG-P III (#1650), were used according to the manufacturer’s instructions. Calibration and quality control procedures were performed before each analysis [64]. Serum tumor necrosis factor-alpha (TNF-α) concentrations were determined using a commercially available enzyme-linked immunosorbent assay (ELISA) kit (Cat. No. 865000192; Bertin Bioreagent, Alfatech S.p.A., Genova, Italy) according to the manufacturer’s instructions. Briefly, serum samples were added to antibody-coated 96-well microplates and incubated with the supplied detection reagents. After the washing steps, absorbance was measured using a microplate reader, and TNF-α concentrations were calculated from a standard curve generated with recombinant TNF-α standards [65].

### 4.4. Histopathological Assessment

Liver tissues were fixed in 10% neutral-buffered formalin, dehydrated, paraffin-embedded, and sectioned into 5 μm slices. Sections were stained with hematoxylin and eosin (H&E) and evaluated for histological alterations. The MASH activity score (MAS) was determined based on established criteria from the World Gastroenterology Organization and included steatosis, hepatocellular ballooning, lobular inflammation, and fibrosis [37]. Samples with MAS ≥ 5 were classified as MASH, whereas samples with MAS ≤ 3 were considered non-MASH [64].

### 4.5. RNA Sequencing and Transcriptomic Analysis

To characterize molecular alterations associated with MCD-induced MASH, RNA sequencing (RNA-seq) was performed on liver tissues from control and MCD-fed mice. Total RNA extraction, library preparation, and sequencing were conducted according to Illumina standard protocols. Raw base-call files were converted to FASTQ format using bcl2fastq (v2.20), and adapter sequences were removed before downstream analysis. Reads were aligned to the reference genome using HISAT2 with default parameters. Differential gene expression analysis was performed using StringTie (v2.1.3) and DESeq2 (v1.28.1) within Welgene Biotech’s analysis pipeline. Genes that met the criteria of a Benjamini–Hochberg false discovery rate (FDR)-adjusted *p*-value < 0.05 and an absolute log2 fold change |log2FC| ≥ 1 were considered significantly differentially expressed. Functional enrichment analyses were conducted using clusterProfiler (v3.6), the Kyoto Encyclopedia of Genes and Genomes (KEGG), and Ingenuity Pathway Analysis (IPA) to identify enriched signaling pathways and regulatory networks [66].

### 4.6. Quantitative Reverse Transcription-Polymerase Chain Reaction (qRT-PCR)

Complementary DNA was synthesized from total RNA using M-MLV reverse transcriptase (Promega, Madison, WI, USA) following the manufacturer’s protocol and stored at −20 °C until use. Quantitative PCR was performed using an AriaMx Real-Time PCR System (Agilent Technologies, Santa Clara, CA, USA) with Brilliant III Ultra-Fast SYBR Green Low ROX qPCR Master Mix (Agilent Technologies). Gene-specific primer sequences are listed in Table 2 [66].

### 4.7. Statistical Analysis

Data are presented as mean ± standard deviation (SD). Statistical analyses were performed using SPSS (Statistics v31, IBM Corp., Armonk, NY, USA). Differences between groups were analyzed via one-way analysis of variance (ANOVA) followed by Tukey’s post hoc test. A *p*-value < 0.05 was considered statistically significant.

## 5. Conclusions

In summary, transcriptomic profiling of the MCD diet-induced MASH model provides a comprehensive overview of the molecular alterations associated with stress-induced hepatic injury. Integrated RNA sequencing, qRT-PCR, and serum TNF-α analyses consistently demonstrate coordinated activation of oxidative stress, inflammatory signaling, impaired cytochrome P450-mediated detoxification, and apoptosis-related pathways. The accompanying fibrosis-associated transcriptional changes are most appropriately interpreted as evidence of early fibrogenic priming rather than established fibrosis. Although the MCD model does not recapitulate the key metabolic characteristics of human obesity-associated MASLD, including obesity and insulin resistance, the identified molecular signatures likely reflect conserved downstream stress-response pathways involved in hepatic injury. Collectively, these findings provide a resource for comparative analyses using metabolically representative MASLD models and environmentally relevant hepatotoxicity models.

## 6. Study Limitations

A limitation of the present study is that hepatic fibrosis was assessed primarily by conventional histopathological examination. Although multiple collagen genes and extracellular matrix remodeling factors were transcriptionally upregulated, fibrosis-specific assessments, including Sirius Red staining, Masson’s trichrome staining, α-SMA immunostaining, and hydroxyproline quantification, were not performed. Consequently, these molecular alterations should be regarded as transcriptional signatures indicative of early fibrogenic priming rather than definitive evidence of established fibrosis.

Furthermore, although representative genes from multiple biological pathways were validated by qRT-PCR and inflammatory activation was further supported by serum TNF-α ELISA, comprehensive protein-level and functional validation of MAPK signaling, apoptosis, oxidative stress responses, and cytochrome P450-mediated detoxification was not undertaken. Accordingly, the pathway alterations identified in this study should be considered transcriptomic signatures that warrant further mechanistic and functional investigation.

Finally, because transcriptomic profiling was conducted at a single experimental time point (day 48), the temporal dynamics of molecular responses during disease progression could not be evaluated. Future studies incorporating longitudinal sampling across different stages of disease development will be important for defining the sequential molecular events underlying MASH progression.

## Figures and Tables

**Table 1:** Metabolic dysfunction-associated steatohepatitis activity scores in liver tissue.

Group	Number	Steatosis	Inflammation	Ballooning	Fibrosis	Sum	Mean ± SD
Control	No 1	0	0	0	0	0	0.00 ± 0.00
	No 2	0	0	0	0	0	
	No 3	0	0	0	0	0	
MCD mice	No 1	3	3	3	0	9	8.60 ± 0.58 ***
	No 2	3	3	3	0	9	
	No 3	3	3	2	0	8	

Metabolic dysfunction-associated steatohepatitis activity score (MAS) of control and MCD-fed mice, including steatosis, hepatocellular ballooning, lobular inflammation, and fibrosis subscores [37]. *** indicates *p* < 0.001 vs. the control group.

**Table 2:** List of primers used for RT-qPCR analysis.

Primer ID	Gene Symbol	Accession Number	Forward Primer (5′–3′)	Reverse Primer (5′–3′)
MT00313-2	*Apoa4*	NM_007468	GTTCAACAAGGCTCTGGTGC	TTTCCACCTCCCCCGAATTG
MP201790	*Casp1*	NM_009807	GGCACATTTCCAGGACTGACTG	GCAAGACGTGTACGAGTGGTTG
MP203320	*Cyp4f18*	NM_024444	GGAAAGGCTCTGTCTGATGAGG	TGGGTGTCTTGCCAGGTTGTAC
MP204664	*Fos*	NM_010234	GGGAATGGTGAAGACCGTGTCA	GCAGCCATCTTATTCCGTTCCC
MP205604	*Gapdh*	NM_008084	AGGTCGGTGTGAACGGATTTG	TGTAGACCATGTAGTTGAGGTCA
MT00029	*Gstp1*	NM_013541	TGTCACCCTCATCTACACCAAC	GGACAGCAGGGTCTCAAAAG
MT00117	*Lpl*	NM_008509	ATGCTGGAATGTTAGCCCTTGC	CCAAACTTGATGAAATCGGTCACC
MT00314	*Mmp12*	NM_008605	GCAGCTGTCTTTGACCCACT	TGGAAATCAGCTTGGGGTAA
MP209060	*Nfkb1*	NM_008689	GCTGCCAAAGAAGGACACGACA	GGCAGGCTATTGCTCATCACAG
MP208965	*Nlrp3*	NM_145827	TCACAACTCGCCCAAGGAGGAA	AAGAGACCACGGCAGAAGCTAG
MT00315	*Sod1*	NM_011434	GGGAAGCATGGCGATGAAAG	GGTTCACCGCTTGCCTTCTG
NP100055	*Tlr4*	NM_021297	AGCTTCTCCAATTTTTCAGAACTTC	TGAGAGGTGGTGTAAGCCATGC

Gene-specific primer sequences used for qRT-PCR validation.

## Data Availability

The data presented in this study are available upon request from the corresponding authors. The RNA sequencing datasets generated during the current study are available from the corresponding authors upon reasonable request.

## Supplementary Materials

The following supporting information can be downloaded at: https://www.mdpi.com/article/10.3390/ijms27136033/s1.
