# Supplementary material for: Transcriptomic Profiling Reveals Inflammatory, Fibrotic, and Apoptotic Signatures in a Methionine–Choline-Deficient Diet-Induced Murine Model of Metabolism-Dysfunction-Associated Steatohepatitis
Source: Int J Mol Sci. 2026 Jul 5;27(13):6033. doi: 10.3390/ijms27136033 (PMC13362325; doi:10.3390/ijms27136033)

Supplementary Figure S1A

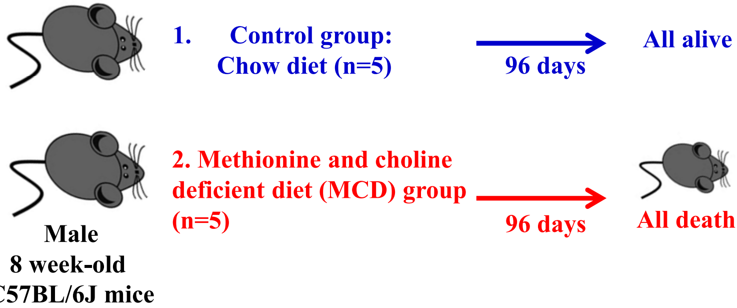

Supplement: Supplementary file 1 [file ijms-27-06033-s001.zip › Supplementary Figure S1A-20260706.pdf]
