# Supplementary material for: Transcriptomic Profiling Reveals Inflammatory, Fibrotic, and Apoptotic Signatures in a Methionine–Choline-Deficient Diet-Induced Murine Model of Metabolism-Dysfunction-Associated Steatohepatitis
Source: Int J Mol Sci. 2026 Jul 5;27(13):6033. doi: 10.3390/ijms27136033 (PMC13362325; doi:10.3390/ijms27136033)
Supplement: Supplementary file 1 [file ijms-27-06033-s001.zip › Supplementary Table S1.pdf]

Supplementary Table S1:Transcriptome sequence analysis results of gene expression profiles on MCD-induced NASH mice				
**Gene ID**	**Gene Name**	**Gene Description**	**Log2 Ratio (MCD/Control)**	**P_Value**
ENSMUSG00000030144	Clec4d	"C-type lectin domain family 4, member d [Source:MGI Symbol;Acc:MGI:1298389]"	15.868	2.05E-11
ENSMUSG00000085651	Gm11695	predicted gene 11695 [Source:MGI Symbol;Acc:MGI:3649841]	15.765	6.25E-07
ENSMUSG00000116048	Septin2	septin 2 [Source:MGI Symbol;Acc:MGI:97298]	15.694	1.68E-09
ENSMUSG00000056054	S100a8	S100 calcium binding protein A8 (calgranulin A) [Source:MGI Symbol;Acc:MGI:88244]	15.515	1.19E-06
ENSMUSG00000056071	S100a9	S100 calcium binding protein A9 (calgranulin B) [Source:MGI Symbol;Acc:MGI:1338947]	15.410	2.80E-06
ENSMUSG00000050359	Sprr1a	small proline-rich protein 1A [Source:MGI Symbol;Acc:MGI:106660]	15.328	7.24E-08
ENSMUSG00000039131	Gipc2	"GIPC PDZ domain containing family, member 2 [Source:MGI Symbol;Acc:MGI:1889209]"	14.995	4.51E-07
ENSMUSG00000018930	Ccl4	chemokine (C-C motif) ligand 4 [Source:MGI Symbol;Acc:MGI:98261]	14.841	2.52E-06
ENSMUSG00000001131	Timp1	tissue inhibitor of metalloproteinase 1 [Source:MGI Symbol;Acc:MGI:98752]	14.740	7.10E-07
ENSMUSG00000029272	Sult1e1	"sulfotransferase family 1E, member 1 [Source:MGI Symbol;Acc:MGI:98431]"	14.545	1.03E-07
ENSMUSG00000093625	Gm18953	"predicted gene, 18953 [Source:MGI Symbol;Acc:MGI:5011138]"	14.333	2.68E-02
ENSMUSG00000023132	Gzma	granzyme A [Source:MGI Symbol;Acc:MGI:109266]	14.276	3.23E-06
ENSMUSG00000017002	Slpi	secretory leukocyte peptidase inhibitor [Source:MGI Symbol;Acc:MGI:109297]	14.180	7.19E-06
ENSMUSG00000051225	Fam83a	"family with sequence similarity 83, member A [Source:MGI Symbol;Acc:MGI:2447773]"	14.075	1.30E-09
ENSMUSG00000085873	Ttc39aos1	Ttc39a opposite strand RNA 1 [Source:MGI Symbol;Acc:MGI:3651956]	13.782	1.20E-02
ENSMUSG00000100593	1700119H24Rik	RIKEN cDNA 1700119H24 gene [Source:MGI Symbol;Acc:MGI:1923899]	13.676	1.43E-03
ENSMUSG00000085295	4930430E12Rik	RIKEN cDNA 4930430E12 gene [Source:MGI Symbol;Acc:MGI:1918889]	13.598	1.05E-03
ENSMUSG00000030147	Clec4b1	"C-type lectin domain family 4, member b1 [Source:MGI Symbol;Acc:MGI:1917060]"	13.588	6.06E-04
ENSMUSG00000104068	Gm37199	"predicted gene, 37199 [Source:MGI Symbol;Acc:MGI:5610427]"	13.576	3.80E-10
ENSMUSG00000101389	Ms4a4a	"membrane-spanning 4-domains, subfamily A, member 4A [Source:MGI Symbol;Acc:MGI:3643932]"	13.474	4.06E-04
ENSMUSG00000085317	Gssos2	"glutathione synthase, opposite strand 2 [Source:MGI Symbol;Acc:MGI:3702171]"	13.287	2.05E-02
ENSMUSG00000049598	Vsig8	V-set and immunoglobulin domain containing 8 [Source:MGI Symbol;Acc:MGI:3642995]	13.242	7.39E-06
ENSMUSG00000021384	Susd3	sushi domain containing 3 [Source:MGI Symbol;Acc:MGI:1913579]	13.233	3.90E-05
ENSMUSG00000040809	Chil3	chitinase-like 3 [Source:MGI Symbol;Acc:MGI:1330860]	13.188	1.72E-05
ENSMUSG00000038379	Ttk	Ttk protein kinase [Source:MGI Symbol;Acc:MGI:1194921]	13.180	6.90E-07
ENSMUSG00000063506	Arhgap22	Rho GTPase activating protein 22 [Source:MGI Symbol;Acc:MGI:2443418]	13.116	7.51E-07
ENSMUSG00000003484	Cyp4f18	"cytochrome P450, family 4, subfamily f, polypeptide 18 [Source:MGI Symbol;Acc:MGI:1919304]"	13.106	1.33E-05
ENSMUSG00000006398	Cdc20	cell division cycle 20 [Source:MGI Symbol;Acc:MGI:1859866]	13.102	2.74E-05
ENSMUSG00000022126	Acod1	aconitate decarboxylase 1 [Source:MGI Symbol;Acc:MGI:103206]	13.028	1.13E-06
ENSMUSG00000066607	Insyn1	inhibitory synaptic factor 1 [Source:MGI Symbol;Acc:MGI:2442108]	13.011	6.61E-05
ENSMUSG00000108190	Gm44731	predicted gene 44731 [Source:MGI Symbol;Acc:MGI:5753307]	12.939	4.13E-02
ENSMUSG00000029371	Cxcl5	chemokine (C-X-C motif) ligand 5 [Source:MGI Symbol;Acc:MGI:1096868]	12.920	4.35E-05
ENSMUSG00000087060	Eldr	Egfr long non-coding downstream RNA [Source:MGI Symbol;Acc:MGI:1919985]	12.910	1.73E-03
ENSMUSG00000049630	C1ql3	C1q-like 3 [Source:MGI Symbol;Acc:MGI:2387350]	12.811	3.99E-06
ENSMUSG00000080115	Eef1akmt3	EEF1A lysine methyltransferase 3 [Source:MGI Symbol;Acc:MGI:3645330]	12.735	3.45E-06
ENSMUSG00000069910	Spdl1	spindle apparatus coiled-coil protein 1 [Source:MGI Symbol;Acc:MGI:1917635]	12.733	1.37E-05
ENSMUSG00000027994	Mcub	mitochondrial calcium uniporter dominant negative beta subunit [Source:MGI Symbol;Acc:MGI:1914065]	12.710	7.84E-03
ENSMUSG00000085890	Tnfsf13os	"tumor necrosis factor (ligand) superfamily, member 13, opposite strand [Source:MGI Symbol;Acc:MGI:1919587]"	12.700	1.10E-02
ENSMUSG00000091572	Vmn2r3	"vomeronasal 2, receptor 3 [Source:MGI Symbol;Acc:MGI:3643995]"	12.671	1.76E-09
ENSMUSG00000022340	Sybu	syntabulin (syntaxin-interacting) [Source:MGI Symbol;Acc:MGI:2442392]	12.667	1.32E-06
ENSMUSG00000039013	Siglecf	sialic acid binding Ig-like lectin F [Source:MGI Symbol;Acc:MGI:2681107]	12.662	5.51E-06
ENSMUSG00000075224	Lrrc55	leucine rich repeat containing 55 [Source:MGI Symbol;Acc:MGI:2685197]	12.644	5.33E-07
ENSMUSG00000081005	Gm13198	predicted gene 13198 [Source:MGI Symbol;Acc:MGI:3651172]	12.637	8.26E-03
ENSMUSG00000029378	Areg	amphiregulin [Source:MGI Symbol;Acc:MGI:88068]	12.607	1.41E-02
ENSMUSG00000022422	Dscc1	DNA replication and sister chromatid cohesion 1 [Source:MGI Symbol;Acc:MGI:1919357]	12.602	4.68E-06
ENSMUSG00000037544	Dlgap5	DLG associated protein 5 [Source:MGI Symbol;Acc:MGI:2183453]	12.602	4.26E-06
ENSMUSG00000026928	Card9	"caspase recruitment domain family, member 9 [Source:MGI Symbol;Acc:MGI:2685628]"	12.600	3.25E-03
ENSMUSG00000058523	Mup5	major urinary protein 5 [Source:MGI Symbol;Acc:MGI:104974]	12.596	3.77E-02

ENSMUSG00000037337	Map4k1	mitogen-activated protein kinase kinase kinase kinase 1 [Source:MGI Symbol;Acc:MGI:1346882]	12.581	3.66E-03
ENSMUSG00000112267	Gm47903	"predicted gene, 47903 [Source:MGI Symbol;Acc:MGI:6097142]"	12.571	2.47E-02
ENSMUSG00000064246	Chil1	chitinase-like 1 [Source:MGI Symbol;Acc:MGI:1340899]	12.547	1.00E-02
ENSMUSG00000103174	Gm37168	"predicted gene, 37168 [Source:MGI Symbol;Acc:MGI:5610396]"	12.519	1.23E-03
ENSMUSG00000030117	Gdf3	growth differentiation factor 3 [Source:MGI Symbol;Acc:MGI:95686]	12.518	5.55E-04
ENSMUSG00000034394	Lif	leukemia inhibitory factor [Source:MGI Symbol;Acc:MGI:96787]	12.438	7.76E-05
ENSMUSG00000030703	Gdpd3	glycerophosphodiester phosphodiesterase domain containing 3 [Source:MGI Symbol;Acc:MGI:1915866]	12.418	3.92E-02
ENSMUSG00000036587	Fut7	fucosyltransferase 7 [Source:MGI Symbol;Acc:MGI:107692]	12.372	3.76E-04
ENSMUSG00000004933	Matk	megakaryocyte-associated tyrosine kinase [Source:MGI Symbol;Acc:MGI:99259]	12.371	7.32E-05
ENSMUSG00000116024	Gm49527	"predicted gene, 49527 [Source:MGI Symbol;Acc:MGI:6155227]"	12.360	3.85E-03
ENSMUSG00000043939	A530064D06Rik	RIKEN cDNA A530064D06 gene [Source:MGI Symbol;Acc:MGI:2443476]	12.341	7.42E-07
ENSMUSG00000097471	5830432E09Rik	RIKEN cDNA 5830432E09 gene [Source:MGI Symbol;Acc:MGI:1915015]	12.317	2.78E-05
ENSMUSG00000113695	9330159N22Rik	RIKEN cDNA 9330159N22 gene [Source:MGI Symbol;Acc:MGI:1924453]	12.280	7.04E-03
ENSMUSG00000108105	Gm5340	predicted gene 5340 [Source:MGI Symbol;Acc:MGI:3779484]	12.259	2.31E-02
ENSMUSG00000028347	Tmeff1	transmembrane protein with EGF-like and two follistatin-like domains 1 [Source:MGI Symbol;Acc:MGI:1926810]	12.211	1.02E-04
ENSMUSG00000102895	5830415G21Rik	RIKEN cDNA 5830415G21 gene [Source:MGI Symbol;Acc:MGI:1923259]	12.209	6.97E-03
ENSMUSG00000116957	Gm34680	"predicted gene, 34680 [Source:MGI Symbol;Acc:MGI:5593839]"	12.208	6.75E-03
ENSMUSG00000036067	Slc2a6	"solute carrier family 2 (facilitated glucose transporter), member 6 [Source:MGI Symbol;Acc:MGI:2443286]"	12.188	7.39E-05
ENSMUSG00000058290	Espl1	"extra spindle pole bodies 1, separase [Source:MGI Symbol;Acc:MGI:2146156]"	12.174	3.27E-07
ENSMUSG00000079553	Kifc1	kinesin family member C1 [Source:MGI Symbol;Acc:MGI:109596]	12.152	3.81E-04
ENSMUSG00000029641	Rasl11a	"RAS-like, family 11, member A [Source:MGI Symbol;Acc:MGI:1916145]"	12.146	2.97E-02
ENSMUSG00000111960	Gm46349	"predicted gene, 46349 [Source:MGI Symbol;Acc:MGI:5825986]"	12.143	1.90E-02
ENSMUSG00000099398	Ms4a14	"membrane-spanning 4-domains, subfamily A, member 14 [Source:MGI Symbol;Acc:MGI:2686122]"	12.132	4.52E-06
ENSMUSG00000105447	Gm43653	predicted gene 43653 [Source:MGI Symbol;Acc:MGI:5663790]	12.106	1.38E-02
ENSMUSG00000109311	AI314278	expressed sequence AI314278 [Source:MGI Symbol;Acc:MGI:2141898]	12.076	5.69E-03
ENSMUSG00000096960	A230028O05Rik	RIKEN cDNA A230028O05 gene [Source:MGI Symbol;Acc:MGI:2442126]	12.072	1.20E-04
ENSMUSG00000017400	Stac2	SH3 and cysteine rich domain 2 [Source:MGI Symbol;Acc:MGI:2144518]	11.972	1.02E-04
ENSMUSG00000113136	Gm19951	"predicted gene, 19951 [Source:MGI Symbol;Acc:MGI:5012136]"	11.954	8.10E-03
ENSMUSG00000056735	A930024E05Rik	RIKEN cDNA A930024E05 gene [Source:MGI Symbol;Acc:MGI:1924414]	11.935	1.47E-03
ENSMUSG00000097021	4933433G15Rik	RIKEN cDNA 4933433G15 gene [Source:MGI Symbol;Acc:MGI:1918524]	11.935	4.69E-02
ENSMUSG00000028463	Car9	carbonic anhydrase 9 [Source:MGI Symbol;Acc:MGI:2447188]	11.924	1.37E-02
ENSMUSG00000114448	C630044B11Rik	RIKEN cDNA C630044B11 gene [Source:MGI Symbol;Acc:MGI:1925962]	11.917	1.31E-03
ENSMUSG00000054510	Gm14461	predicted gene 14461 [Source:MGI Symbol;Acc:MGI:3651589]	11.902	4.84E-02
ENSMUSG00000028587	Orc1	"origin recognition complex, subunit 1 [Source:MGI Symbol;Acc:MGI:1328337]"	11.876	3.14E-03
ENSMUSG00000084020	Wsb2-ps	"WD repeat and SOCS box-containing 2, pseudogene [Source:MGI Symbol;Acc:MGI:3649891]"	11.866	1.73E-02
ENSMUSG00000022303	Dcstamp	dendrocyte expressed seven transmembrane protein [Source:MGI Symbol;Acc:MGI:1923016]	11.861	1.53E-03
ENSMUSG00000004296	Il12b	interleukin 12b [Source:MGI Symbol;Acc:MGI:96540]	11.855	8.48E-03
ENSMUSG00000073402	Gm8909	predicted gene 8909 [Source:MGI Symbol;Acc:MGI:3704134]	11.798	2.68E-02
ENSMUSG00000067714	Lpar5	lysophosphatidic acid receptor 5 [Source:MGI Symbol;Acc:MGI:2685918]	11.795	4.43E-02
ENSMUSG00000091813	Ces2h	carboxylesterase 2H [Source:MGI Symbol;Acc:MGI:3648740]	11.779	1.24E-03
ENSMUSG00000090799	Klhl33	kelch-like 33 [Source:MGI Symbol;Acc:MGI:3644593]	11.746	2.57E-04
ENSMUSG00000025582	Nptx1	neuronal pentraxin 1 [Source:MGI Symbol;Acc:MGI:107811]	11.725	4.74E-06
ENSMUSG00000091476	Catspere2	cation channel sperm associated auxiliary subunit epsilon 2 [Source:MGI Symbol;Acc:MGI:5589632]	11.696	2.50E-03
ENSMUSG00000052125	F730043M19Rik	RIKEN cDNA F730043M19 gene [Source:MGI Symbol;Acc:MGI:2443237]	11.683	2.09E-04
ENSMUSG00000101969	Gm20125	"predicted gene, 20125 [Source:MGI Symbol;Acc:MGI:5012310]"	11.654	4.61E-03
ENSMUSG00000031562	Dctd	dCMP deaminase [Source:MGI Symbol;Acc:MGI:2444529]	11.644	3.59E-02
ENSMUSG00000086291	Gm15513	predicted gene 15513 [Source:MGI Symbol;Acc:MGI:3782961]	11.644	9.30E-03
ENSMUSG00000105139	Gm19391	"predicted gene, 19391 [Source:MGI Symbol;Acc:MGI:5011576]"	11.575	5.29E-03
ENSMUSG00000035285	Nat14	N-acetyltransferase 14 [Source:MGI Symbol;Acc:MGI:3039561]	11.574	4.78E-02
ENSMUSG00000085355	3010003L21Rik	RIKEN cDNA 3010003L21 gene [Source:MGI Symbol;Acc:MGI:1924094]	11.547	4.43E-02

ENSMUSG00000044103	Il1f9	"interleukin 1 family, member 9 [Source:MGI Symbol;Acc:MGI:2449929]"	11.508	2.60E-02
ENSMUSG00000035279	Ssc5d	"scavenger receptor cysteine rich family, 5 domains [Source:MGI Symbol;Acc:MGI:3606211]"	11.480	1.17E-04
ENSMUSG00000093765	Gm20658	predicted gene 20658 [Source:MGI Symbol;Acc:MGI:5313105]	11.471	3.98E-02
ENSMUSG00000020325	Fstl3	follistatin-like 3 [Source:MGI Symbol;Acc:MGI:1890391]	11.468	5.74E-03
ENSMUSG00000111692	Gm49373	"predicted gene, 49373 [Source:MGI Symbol;Acc:MGI:6121591]"	11.462	4.13E-02
ENSMUSG00000019992	Mtfr2	mitochondrial fission regulator 2 [Source:MGI Symbol;Acc:MGI:1919054]	11.381	4.18E-02
ENSMUSG00000104690	Gm47304	"predicted gene, 47304 [Source:MGI Symbol;Acc:MGI:6096173]"	11.375	6.82E-04
ENSMUSG00000097467	Gm26737	"predicted gene, 26737 [Source:MGI Symbol;Acc:MGI:5477231]"	11.373	2.64E-02
ENSMUSG00000051074	4930579K19Rik	RIKEN cDNA 4930579K19 gene [Source:MGI Symbol;Acc:MGI:1923131]	11.355	4.36E-02
ENSMUSG00000097331	F420014N23Rik	RIKEN cDNA F420014N23 gene [Source:MGI Symbol;Acc:MGI:3642477]	11.347	2.87E-03
ENSMUSG00000041552	Ptchd1	patched domain containing 1 [Source:MGI Symbol;Acc:MGI:2685233]	11.341	1.88E-02
ENSMUSG00000010154	Spire2	spire type actin nucleation factor 2 [Source:MGI Symbol;Acc:MGI:2446256]	11.339	1.06E-02
ENSMUSG00000085687	Gm16153	predicted gene 16153 [Source:MGI Symbol;Acc:MGI:3802057]	11.336	1.38E-04
ENSMUSG00000103907	Gm37498	"predicted gene, 37498 [Source:MGI Symbol;Acc:MGI:5610726]"	11.336	4.90E-06
ENSMUSG00000090118	Gm16163	predicted gene 16163 [Source:MGI Symbol;Acc:MGI:3833836]	11.317	4.64E-02
ENSMUSG00000116665	E130310I04Rik	RIKEN cDNA E130310I04 gene [Source:MGI Symbol;Acc:MGI:3046461]	11.315	6.99E-03
ENSMUSG00000105302	Gm19817	"predicted gene, 19817 [Source:MGI Symbol;Acc:MGI:5012002]"	11.309	9.00E-03
ENSMUSG00000032487	Ptgs2	prostaglandin-endoperoxide synthase 2 [Source:MGI Symbol;Acc:MGI:97798]	11.300	1.84E-04
ENSMUSG00000003070	Efna2	ephrin A2 [Source:MGI Symbol;Acc:MGI:102707]	11.277	4.40E-02
ENSMUSG00000010660	Plcd1	"phospholipase C, delta 1 [Source:MGI Symbol;Acc:MGI:97614]"	11.274	2.10E-02
ENSMUSG00000022385	Gtse1	G two S phase expressed protein 1 [Source:MGI Symbol;Acc:MGI:1352755]	11.251	2.10E-02
ENSMUSG00000102715	Gm6209	predicted gene 6209 [Source:MGI Symbol;Acc:MGI:3643374]	11.244	1.27E-02
ENSMUSG00000085589	A430078I02Rik	RIKEN cDNA A430078I02 gene [Source:MGI Symbol;Acc:MGI:2444132]	11.229	4.26E-03
ENSMUSG00000049420	Tmem200a	transmembrane protein 200A [Source:MGI Symbol;Acc:MGI:1924470]	11.202	9.72E-04
ENSMUSG00000071550	Cfap44	cilia and flagella associated protein 44 [Source:MGI Symbol;Acc:MGI:1277238]	11.195	1.60E-05
ENSMUSG00000106385	Gm42943	predicted gene 42943 [Source:MGI Symbol;Acc:MGI:5663080]	11.188	1.91E-03
ENSMUSG00000029228	Lnx1	ligand of numb-protein X 1 [Source:MGI Symbol;Acc:MGI:1278335]	11.165	1.54E-02
ENSMUSG00000102549	Gm38137	"predicted gene, 38137 [Source:MGI Symbol;Acc:MGI:5611365]"	11.162	4.19E-02
ENSMUSG00000051354	Samd3	sterile alpha motif domain containing 3 [Source:MGI Symbol;Acc:MGI:2685469]	11.151	7.06E-03
ENSMUSG00000032446	Eomes	eomesodermin [Source:MGI Symbol;Acc:MGI:1201683]	11.134	6.89E-03
ENSMUSG00000056598	Drc3	dynein regulatory complex subunit 3 [Source:MGI Symbol;Acc:MGI:1921915]	11.104	2.68E-02
ENSMUSG00000096221	1500002C15Rik	RIKEN cDNA 1500002C15 gene [Source:MGI Symbol;Acc:MGI:1916196]	11.102	8.64E-03
ENSMUSG00000051515	Fam181b	"family with sequence similarity 181, member B [Source:MGI Symbol;Acc:MGI:1930951]"	11.096	1.93E-02
ENSMUSG00000038112	AW551984	expressed sequence AW551984 [Source:MGI Symbol;Acc:MGI:2143322]	11.051	5.57E-04
ENSMUSG00000005800	Mmp8	matrix metallopeptidase 8 [Source:MGI Symbol;Acc:MGI:1202395]	11.046	3.74E-02
ENSMUSG00000042109	Csdc2	"cold shock domain containing C2, RNA binding [Source:MGI Symbol;Acc:MGI:2146027]"	11.006	1.60E-02
ENSMUSG00000058447	Gm26920	"predicted gene, 26920 [Source:MGI Symbol;Acc:MGI:5504035]"	10.994	3.55E-02
ENSMUSG00000036412	Arsi	arylsulfatase i [Source:MGI Symbol;Acc:MGI:2670959]	10.987	3.56E-03
ENSMUSG00000110388	Gm30329	"predicted gene, 30329 [Source:MGI Symbol;Acc:MGI:5589488]"	10.967	1.99E-02
ENSMUSG00000027612	Mmp24	matrix metallopeptidase 24 [Source:MGI Symbol;Acc:MGI:1341867]	10.928	7.58E-03
ENSMUSG00000033644	Piwil2	piwi-like RNA-mediated gene silencing 2 [Source:MGI Symbol;Acc:MGI:1930036]	10.921	6.33E-03
ENSMUSG00000068452	Duox2	dual oxidase 2 [Source:MGI Symbol;Acc:MGI:3036280]	10.900	5.50E-04
ENSMUSG00000040714	Klc3	kinesin light chain 3 [Source:MGI Symbol;Acc:MGI:1277971]	10.881	2.63E-02
ENSMUSG00000040434	Large2	LARGE xylosyl- and glucuronyltransferase 2 [Source:MGI Symbol;Acc:MGI:2443769]	10.877	2.10E-02
ENSMUSG00000071506	Tmem139	transmembrane protein 139 [Source:MGI Symbol;Acc:MGI:1924444]	10.873	1.88E-02
ENSMUSG00000019214	Chtf18	"CTF18, chromosome transmission fidelity factor 18 [Source:MGI Symbol;Acc:MGI:2384887]"	10.758	1.41E-02
ENSMUSG00000021062	Rab15	"RAB15, member RAS oncogene family [Source:MGI Symbol;Acc:MGI:1916865]"	10.738	6.60E-03
ENSMUSG00000105041	Gm42676	predicted gene 42676 [Source:MGI Symbol;Acc:MGI:5662813]	10.682	1.83E-02
ENSMUSG00000044288	Cnr1	cannabinoid receptor 1 (brain) [Source:MGI Symbol;Acc:MGI:104615]	10.647	1.41E-03
ENSMUSG00000049848	Ceacam19	carcinoembryonic antigen-related cell adhesion molecule 19 [Source:MGI Symbol;Acc:MGI:2443001]	10.639	8.54E-03

ENSMUSG00000023903	Mmp25	matrix metallopeptidase 25 [Source:MGI Symbol;Acc:MGI:2443938]	10.638	1.70E-03
ENSMUSG00000032358	Fam83b	"family with sequence similarity 83, member B [Source:MGI Symbol;Acc:MGI:2685362]"	10.616	4.01E-03
ENSMUSG00000037991	Rmi2	RecQ mediated genome instability 2 [Source:MGI Symbol;Acc:MGI:2685383]	10.611	7.59E-03
ENSMUSG00000106247	Gm43720	predicted gene 43720 [Source:MGI Symbol;Acc:MGI:5663857]	10.601	5.03E-04
ENSMUSG00000032507	Fbxl2	F-box and leucine-rich repeat protein 2 [Source:MGI Symbol;Acc:MGI:1919429]	10.597	4.04E-02
ENSMUSG00000111398	D330037F02Rik	RIKEN cDNA D330037F02 gene [Source:MGI Symbol;Acc:MGI:3041221]	10.585	3.57E-02
ENSMUSG00000118004	BC026513	cDNA sequence BC026513 [Source:MGI Symbol;Acc:MGI:2652849]	10.522	4.59E-02
ENSMUSG00000040729	Cep126	centrosomal protein 126 [Source:MGI Symbol;Acc:MGI:2680221]	10.506	9.16E-03
ENSMUSG00000061577	Adgrg5	adhesion G protein-coupled receptor G5 [Source:MGI Symbol;Acc:MGI:2685955]	10.500	3.92E-02
ENSMUSG00000081138	Gm12834	predicted gene 12834 [Source:MGI Symbol;Acc:MGI:3649428]	10.405	3.45E-02
ENSMUSG00000061451	Tmem151a	transmembrane protein 151A [Source:MGI Symbol;Acc:MGI:2147713]	10.397	8.10E-03
ENSMUSG00000043385	Olfr267	olfactory receptor 267 [Source:MGI Symbol;Acc:MGI:3030101]	10.390	7.26E-03
ENSMUSG00000035849	Krt222	keratin 222 [Source:MGI Symbol;Acc:MGI:2442728]	10.354	4.81E-02
ENSMUSG00000117239	Gpr31c	"G protein-coupled receptor 31, D17Leh66c region [Source:MGI Symbol;Acc:MGI:1354371]"	10.263	7.51E-03
ENSMUSG00000050967	Creg2	cellular repressor of E1A-stimulated genes 2 [Source:MGI Symbol;Acc:MGI:1928333]	10.249	1.47E-02
ENSMUSG00000008461	Fut1	fucosyltransferase 1 [Source:MGI Symbol;Acc:MGI:109375]	10.248	3.57E-02
ENSMUSG00000106547	B230303O12Rik	RIKEN cDNA B230303O12 gene [Source:MGI Symbol;Acc:MGI:2442644]	10.237	1.12E-02
ENSMUSG00000026413	Pkp1	plakophilin 1 [Source:MGI Symbol;Acc:MGI:1328359]	10.213	1.77E-02
ENSMUSG00000037139	Myom3	"myomesin family, member 3 [Source:MGI Symbol;Acc:MGI:2685280]"	10.209	1.06E-02
ENSMUSG00000037188	Grhl3	grainyhead like transcription factor 3 [Source:MGI Symbol;Acc:MGI:2655333]	10.190	3.62E-02
ENSMUSG00000049556	Lingo1	leucine rich repeat and Ig domain containing 1 [Source:MGI Symbol;Acc:MGI:1915522]	10.067	1.70E-02
ENSMUSG00000029816	Gpnmb	glycoprotein (transmembrane) nmb [Source:MGI Symbol;Acc:MGI:1934765]	10.049	1.13E-27
ENSMUSG00000087266	Gm15991	predicted gene 15991 [Source:MGI Symbol;Acc:MGI:3801813]	9.998	3.45E-02
ENSMUSG00000049723	Mmp12	matrix metallopeptidase 12 [Source:MGI Symbol;Acc:MGI:97005]	9.306	8.56E-72
ENSMUSG00000103596	Gm37354	"predicted gene, 37354 [Source:MGI Symbol;Acc:MGI:5610582]"	9.269	4.75E-03
ENSMUSG00000099519	Gm29253	predicted gene 29253 [Source:MGI Symbol;Acc:MGI:5579959]	8.716	3.81E-05
ENSMUSG00000023992	Trem2	triggering receptor expressed on myeloid cells 2 [Source:MGI Symbol;Acc:MGI:1913150]	8.625	1.25E-16
ENSMUSG00000103445	Gm36948	"predicted gene, 36948 [Source:MGI Symbol;Acc:MGI:5610176]"	8.498	2.38E-02
ENSMUSG00000085998	AW822252	expressed sequence AW822252 [Source:MGI Symbol;Acc:MGI:2148030]	8.494	4.25E-02
ENSMUSG00000107436	Gm44416	"predicted gene, 44416 [Source:MGI Symbol;Acc:MGI:5690808]"	8.334	4.57E-02
ENSMUSG00000027737	Slc7a11	"solute carrier family 7 (cationic amino acid transporter, y+ system), member 11 [Source:MGI Symbol;Acc:MGI:1347355]"	8.018	4.93E-25
ENSMUSG00000026822	Lcn2	lipocalin 2 [Source:MGI Symbol;Acc:MGI:96757]	7.215	1.09E-72
ENSMUSG00000025473	Adam8	a disintegrin and metallopeptidase domain 8 [Source:MGI Symbol;Acc:MGI:107825]	7.128	8.34E-04
ENSMUSG00000040660	Cyp2b9	"cytochrome P450, family 2, subfamily b, polypeptide 9 [Source:MGI Symbol;Acc:MGI:88600]"	7.045	4.38E-08
ENSMUSG00000034799	Unc13a	unc-13 homolog A [Source:MGI Symbol;Acc:MGI:3051532]	7.025	7.24E-03
ENSMUSG00000026628	Atf3	activating transcription factor 3 [Source:MGI Symbol;Acc:MGI:109384]	6.999	1.09E-25
ENSMUSG00000115074	Ndor1	NADPH dependent diflavin oxidoreductase 1 [Source:MGI Symbol;Acc:MGI:1926047]	6.917	1.58E-04
ENSMUSG00000035186	Ubd	ubiquitin D [Source:MGI Symbol;Acc:MGI:1344410]	6.701	1.41E-03
ENSMUSG00000030142	Clec4e	"C-type lectin domain family 4, member e [Source:MGI Symbol;Acc:MGI:1861232]"	6.616	2.46E-09
ENSMUSG00000117813	Gm5823	predicted gene 5823 [Source:MGI Symbol;Acc:MGI:3647390]	6.463	2.33E-02
ENSMUSG00000029161	Cgref1	cell growth regulator with EF hand domain 1 [Source:MGI Symbol;Acc:MGI:1915817]	6.407	1.20E-07
ENSMUSG00000109618	Gm45527	predicted gene 45527 [Source:MGI Symbol;Acc:MGI:5791363]	6.370	1.38E-03
ENSMUSG00000034634	Ly6d	"lymphocyte antigen 6 complex, locus D [Source:MGI Symbol;Acc:MGI:96881]"	6.333	4.72E-25
ENSMUSG00000110440	Gm45894	predicted gene 45894 [Source:MGI Symbol;Acc:MGI:5805009]	6.228	1.39E-02
ENSMUSG00000090675	Olfr111	olfactory receptor 111 [Source:MGI Symbol;Acc:MGI:2177494]	6.186	6.32E-07
ENSMUSG00000022861	Dgkg	"diacylglycerol kinase, gamma [Source:MGI Symbol;Acc:MGI:105060]"	5.966	9.11E-05
ENSMUSG00000026358	Rgs1	regulator of G-protein signaling 1 [Source:MGI Symbol;Acc:MGI:1354694]	5.965	4.10E-27
ENSMUSG00000110018	5430437J10Rik	RIKEN cDNA 5430437J10 gene [Source:MGI Symbol;Acc:MGI:1918682]	5.911	7.39E-05
ENSMUSG00000114608	Gm36161	"predicted gene, 36161 [Source:MGI Symbol;Acc:MGI:5595320]"	5.814	9.13E-16
ENSMUSG00000100277	1810053B23Rik	RIKEN cDNA 1810053B23 gene [Source:MGI Symbol;Acc:MGI:1917107]	5.812	4.72E-05

ENSMUSG00000095609	Gm21188	"predicted gene, 21188 [Source:MGI Symbol;Acc:MGI:5434543]"	5.740	6.80E-26
ENSMUSG00000064452	Gm24564	"predicted gene, 24564 [Source:MGI Symbol;Acc:MGI:5454341]"	5.735	4.24E-02
ENSMUSG00000067879	Vxn	vexin [Source:MGI Symbol;Acc:MGI:1924232]	5.639	7.27E-05
ENSMUSG00000028427	Aqp7	aquaporin 7 [Source:MGI Symbol;Acc:MGI:1314647]	5.630	1.58E-04
ENSMUSG00000111684	Gm8543	predicted gene 8543 [Source:MGI Symbol;Acc:MGI:3648878]	5.547	2.96E-02
ENSMUSG00000098243	Gm4258	predicted gene 4258 [Source:MGI Symbol;Acc:MGI:3782435]	5.524	2.36E-03
ENSMUSG00000040907	Atp1a3	"ATPase, Na+/K+ transporting, alpha 3 polypeptide [Source:MGI Symbol;Acc:MGI:88107]"	5.478	2.19E-08
ENSMUSG00000038550	Ciart	circadian associated repressor of transcription [Source:MGI Symbol;Acc:MGI:2684975]	5.477	4.56E-05
ENSMUSG00000050578	Mmp13	matrix metallopeptidase 13 [Source:MGI Symbol;Acc:MGI:1340026]	5.452	8.36E-04
ENSMUSG00000104017	Gm37363	"predicted gene, 37363 [Source:MGI Symbol;Acc:MGI:5610591]"	5.399	3.69E-02
ENSMUSG00000056737	Capg	"capping protein (actin filament), gelsolin-like [Source:MGI Symbol;Acc:MGI:1098259]"	5.392	3.52E-14
ENSMUSG00000113184	Gm49654	"predicted gene, 49654 [Source:MGI Symbol;Acc:MGI:6215088]"	5.264	3.84E-02
ENSMUSG00000015880	Ncapg	"non-SMC condensin I complex, subunit G [Source:MGI Symbol;Acc:MGI:1930197]"	5.259	9.85E-07
ENSMUSG00000052271	Bhlha15	"basic helix-loop-helix family, member a15 [Source:MGI Symbol;Acc:MGI:891976]"	5.227	2.66E-05
ENSMUSG00000021403	Serpinb9b	"serine (or cysteine) peptidase inhibitor, clade B, member 9b [Source:MGI Symbol;Acc:MGI:894668]"	5.226	9.79E-03
ENSMUSG00000039396	Neil3	nei like 3 (E. coli) [Source:MGI Symbol;Acc:MGI:2384588]	5.205	1.53E-02
ENSMUSG00000078771	Evi2a	ecotropic viral integration site 2a [Source:MGI Symbol;Acc:MGI:95458]	5.156	3.35E-02
ENSMUSG00000037868	Egr2	early growth response 2 [Source:MGI Symbol;Acc:MGI:95296]	5.119	1.20E-11
ENSMUSG00000095028	Sirpb1b	signal-regulatory protein beta 1B [Source:MGI Symbol;Acc:MGI:3779828]	5.118	3.47E-04
ENSMUSG00000117575	Gm36486	"predicted gene, 36486 [Source:MGI Symbol;Acc:MGI:5595645]"	5.092	1.87E-03
ENSMUSG00000011008	Mcoln2	mucolipin 2 [Source:MGI Symbol;Acc:MGI:1915529]	5.086	5.37E-03
ENSMUSG00000008318	Relt	RELT tumor necrosis factor receptor [Source:MGI Symbol;Acc:MGI:2443373]	5.075	2.34E-03
ENSMUSG00000113259	Gm35190	"predicted gene, 35190 [Source:MGI Symbol;Acc:MGI:5594349]"	5.067	8.01E-03
ENSMUSG00000025912	Mybl1	myeloblastosis oncogene-like 1 [Source:MGI Symbol;Acc:MGI:99925]	5.049	4.61E-26
ENSMUSG00000000982	Ccl3	chemokine (C-C motif) ligand 3 [Source:MGI Symbol;Acc:MGI:98260]	4.991	1.05E-04
ENSMUSG00000037411	Serpine1	"serine (or cysteine) peptidase inhibitor, clade E, member 1 [Source:MGI Symbol;Acc:MGI:97608]"	4.972	1.51E-14
ENSMUSG00000030278	Cidec	cell death-inducing DFFA-like effector c [Source:MGI Symbol;Acc:MGI:95585]	4.970	6.78E-11
ENSMUSG00000044162	Tnip3	TNFAIP3 interacting protein 3 [Source:MGI Symbol;Acc:MGI:3041165]	4.915	5.29E-10
ENSMUSG00000035373	Ccl7	chemokine (C-C motif) ligand 7 [Source:MGI Symbol;Acc:MGI:99512]	4.914	1.88E-02
ENSMUSG00000030789	Itgax	integrin alpha X [Source:MGI Symbol;Acc:MGI:96609]	4.912	1.94E-22
ENSMUSG00000053687	Dpep2	dipeptidase 2 [Source:MGI Symbol;Acc:MGI:2442042]	4.895	2.81E-03
ENSMUSG00000050335	Lgals3	"lectin, galactose binding, soluble 3 [Source:MGI Symbol;Acc:MGI:96778]"	4.885	3.13E-28
ENSMUSG00000116902	Gm49660	"predicted gene, 49660 [Source:MGI Symbol;Acc:MGI:6215098]"	4.878	3.63E-06
ENSMUSG00000104088	Gm38275	"predicted gene, 38275 [Source:MGI Symbol;Acc:MGI:5611503]"	4.870	1.85E-02
ENSMUSG00000040751	Lat2	"linker for activation of T cells family, member 2 [Source:MGI Symbol;Acc:MGI:1926479]"	4.855	1.16E-09
ENSMUSG00000083070	Gm13350	predicted gene 13350 [Source:MGI Symbol;Acc:MGI:3651066]	4.850	2.65E-02
ENSMUSG00000103313	Gm38357	"predicted gene, 38357 [Source:MGI Symbol;Acc:MGI:5611585]"	4.841	9.89E-36
ENSMUSG00000028555	Ttc39a	tetratricopeptide repeat domain 39A [Source:MGI Symbol;Acc:MGI:2444350]	4.834	2.98E-10
ENSMUSG00000117872	A530088E08Rik	RIKEN cDNA A530088E08 gene [Source:MGI Symbol;Acc:MGI:3603459]	4.824	1.18E-02
ENSMUSG00000028238	Atp6v0d2	"ATPase, H+ transporting, lysosomal V0 subunit D2 [Source:MGI Symbol;Acc:MGI:1924415]"	4.823	1.64E-24
ENSMUSG00000057359	Gm17494	"predicted gene, 17494 [Source:MGI Symbol;Acc:MGI:4937128]"	4.804	3.58E-02
ENSMUSG00000034593	Myo5a	myosin VA [Source:MGI Symbol;Acc:MGI:105976]	4.789	1.01E-25
ENSMUSG00000107177	Gm43845	predicted gene 43845 [Source:MGI Symbol;Acc:MGI:5663982]	4.782	2.76E-03
ENSMUSG00000001020	S100a4	S100 calcium binding protein A4 [Source:MGI Symbol;Acc:MGI:1330282]	4.778	1.37E-04
ENSMUSG00000114425	Gm33447	"predicted gene, 33447 [Source:MGI Symbol;Acc:MGI:5592606]"	4.768	2.56E-02
ENSMUSG00000029254	Stap1	signal transducing adaptor family member 1 [Source:MGI Symbol;Acc:MGI:1926193]	4.766	2.09E-27
ENSMUSG00000024640	Psat1	phosphoserine aminotransferase 1 [Source:MGI Symbol;Acc:MGI:2183441]	4.760	8.95E-08
ENSMUSG00000097961	Gm27000	"predicted gene, 27000 [Source:MGI Symbol;Acc:MGI:5504115]"	4.745	5.75E-04
ENSMUSG00000089054	Gm23350	"predicted gene, 23350 [Source:MGI Symbol;Acc:MGI:5453127]"	4.737	1.32E-02
ENSMUSG00000030584	Dpf1	"D4, zinc and double PHD fingers family 1 [Source:MGI Symbol;Acc:MGI:1352748]"	4.726	4.56E-02

ENSMUSG00000087362	Gm13710	predicted gene 13710 [Source:MGI Symbol;Acc:MGI:3650894]	4.723	7.50E-03
ENSMUSG00000038252	Ncapd2	"non-SMC condensin I complex, subunit D2 [Source:MGI Symbol;Acc:MGI:1915548]"	4.721	2.25E-04
ENSMUSG00000038963	Slco4a1	"solute carrier organic anion transporter family, member 4a1 [Source:MGI Symbol;Acc:MGI:1351866]"	4.709	4.10E-02
ENSMUSG00000117850	Gm50443	"predicted gene, 50443 [Source:MGI Symbol;Acc:MGI:6303379]"	4.692	4.64E-02
ENSMUSG00000102975	Gm37347	"predicted gene, 37347 [Source:MGI Symbol;Acc:MGI:5610575]"	4.690	1.13E-02
ENSMUSG00000072621	Slfn10-ps	"schlafen 10, pseudogene [Source:MGI Symbol;Acc:MGI:3512288]"	4.686	1.38E-04
ENSMUSG00000053977	Cd8a	"CD8 antigen, alpha chain [Source:MGI Symbol;Acc:MGI:88346]"	4.679	8.08E-06
ENSMUSG00000000031	H19	"H19, imprinted maternally expressed transcript [Source:MGI Symbol;Acc:MGI:95891]"	4.666	1.27E-04
ENSMUSG00000040133	Gpr176	G protein-coupled receptor 176 [Source:MGI Symbol;Acc:MGI:2685858]	4.650	4.12E-04
ENSMUSG00000049109	Themis	thymocyte selection associated [Source:MGI Symbol;Acc:MGI:2443552]	4.635	5.71E-17
ENSMUSG00000108596	Gm49368	"predicted gene, 49368 [Source:MGI Symbol;Acc:MGI:6121583]"	4.624	1.80E-08
ENSMUSG00000111951	Gm48755	"predicted gene, 48755 [Source:MGI Symbol;Acc:MGI:6098434]"	4.615	1.09E-02
ENSMUSG00000102316	Gm37629	"predicted gene, 37629 [Source:MGI Symbol;Acc:MGI:5610857]"	4.613	1.59E-03
ENSMUSG00000079293	Clec7a	"C-type lectin domain family 7, member a [Source:MGI Symbol;Acc:MGI:1861431]"	4.600	1.24E-30
ENSMUSG00000112067	Gm48015	"predicted gene, 48015 [Source:MGI Symbol;Acc:MGI:6097323]"	4.598	8.06E-06
ENSMUSG00000024401	Tnf	tumor necrosis factor [Source:MGI Symbol;Acc:MGI:104798]	4.584	3.61E-03
ENSMUSG00000034206	Polq	"polymerase (DNA directed), theta [Source:MGI Symbol;Acc:MGI:2155399]"	4.578	1.38E-03
ENSMUSG00000050370	Ch25h	cholesterol 25-hydroxylase [Source:MGI Symbol;Acc:MGI:1333869]	4.573	4.47E-02
ENSMUSG00000086443	4933421A08Rik	RIKEN cDNA 4933421A08 gene [Source:MGI Symbol;Acc:MGI:1918347]	4.572	2.30E-04
ENSMUSG00000038352	Arl5c	ADP-ribosylation factor-like 5C [Source:MGI Symbol;Acc:MGI:3028577]	4.571	3.65E-02
ENSMUSG00000106646	Gm42483	predicted gene 42483 [Source:MGI Symbol;Acc:MGI:5662620]	4.570	5.53E-03
ENSMUSG00000103332	Pcdhga2	"protocadherin gamma subfamily A, 2 [Source:MGI Symbol;Acc:MGI:1935214]"	4.558	1.14E-02
ENSMUSG00000112023	Lilr4b	"leukocyte immunoglobulin-like receptor, subfamily B, member 4B [Source:MGI Symbol;Acc:MGI:102702]"	4.558	2.78E-60
ENSMUSG00000031398	Plxna3	plexin A3 [Source:MGI Symbol;Acc:MGI:107683]	4.552	1.37E-02
ENSMUSG00000107198	Gm19619	"predicted gene, 19619 [Source:MGI Symbol;Acc:MGI:5011804]"	4.510	6.04E-38
ENSMUSG00000118507	AL807811.1	aldo-keto reductase family 7 member A2 (AKR7A2) pseudogene	4.508	2.74E-02
ENSMUSG00000111341	Gm47111	"predicted gene, 47111 [Source:MGI Symbol;Acc:MGI:6095851]"	4.504	1.70E-02
ENSMUSG00000102418	Sh2d1b1	SH2 domain containing 1B1 [Source:MGI Symbol;Acc:MGI:1349420]	4.482	1.36E-04
ENSMUSG00000104871	Gm42639	predicted gene 42639 [Source:MGI Symbol;Acc:MGI:5662776]	4.480	1.19E-07
ENSMUSG00000116740	Gm49659	"predicted gene, 49659 [Source:MGI Symbol;Acc:MGI:6215096]"	4.468	2.71E-04
ENSMUSG00000095788	Sirpb1a	signal-regulatory protein beta 1A [Source:MGI Symbol;Acc:MGI:2444824]	4.464	3.06E-03
ENSMUSG00000109574	B230206I08Rik	RIKEN cDNA B230206I08 gene [Source:MGI Symbol;Acc:MGI:1926094]	4.451	9.15E-03
ENSMUSG00000082676	Gm11843	predicted gene 11843 [Source:MGI Symbol;Acc:MGI:3651166]	4.450	5.81E-06
ENSMUSG00000063193	Cd300lb	CD300 molecule like family member B [Source:MGI Symbol;Acc:MGI:2685099]	4.436	3.26E-22
ENSMUSG00000024579	Pcyox1l	prenylcysteine oxidase 1 like [Source:MGI Symbol;Acc:MGI:3606062]	4.428	2.32E-02
ENSMUSG00000118257	9630014M24Rik	RIKEN cDNA 9630014M24 gene [Source:MGI Symbol;Acc:MGI:3588234]	4.426	1.82E-02
ENSMUSG00000052026	Slc6a7	"solute carrier family 6 (neurotransmitter transporter, L-proline), member 7 [Source:MGI Symbol;Acc:MGI:2147363]"	4.424	2.90E-03
ENSMUSG00000028655	Mfsd2a	major facilitator superfamily domain containing 2A [Source:MGI Symbol;Acc:MGI:1923824]	4.402	3.96E-24
ENSMUSG00000099930	Gm2396	predicted gene 2396 [Source:MGI Symbol;Acc:MGI:3780564]	4.398	7.79E-03
ENSMUSG00000071324	Armc2	armadillo repeat containing 2 [Source:MGI Symbol;Acc:MGI:1916449]	4.391	1.68E-03
ENSMUSG00000105877	Gm42479	predicted gene 42479 [Source:MGI Symbol;Acc:MGI:5662616]	4.382	2.67E-05
ENSMUSG00000030827	Fgf21	fibroblast growth factor 21 [Source:MGI Symbol;Acc:MGI:1861377]	4.374	8.55E-04
ENSMUSG00000047507	Baiap3	BAI1-associated protein 3 [Source:MGI Symbol;Acc:MGI:2685783]	4.371	1.64E-02
ENSMUSG00000110235	Gm5086	predicted gene 5086 [Source:MGI Symbol;Acc:MGI:3644417]	4.347	6.78E-03
ENSMUSG00000088775	Gm26037	"predicted gene, 26037 [Source:MGI Symbol;Acc:MGI:5455814]"	4.346	1.76E-02
ENSMUSG00000112148	Lilrb4a	"leukocyte immunoglobulin-like receptor, subfamily B, member 4A [Source:MGI Symbol;Acc:MGI:102701]"	4.344	1.47E-72
ENSMUSG00000020905	Usp43	ubiquitin specific peptidase 43 [Source:MGI Symbol;Acc:MGI:2444541]	4.336	1.16E-02
ENSMUSG00000031906	Smpd3	"sphingomyelin phosphodiesterase 3, neutral [Source:MGI Symbol;Acc:MGI:1927578]"	4.327	1.61E-20
ENSMUSG00000089669	Tnfsf13	"tumor necrosis factor (ligand) superfamily, member 13 [Source:MGI Symbol;Acc:MGI:1916833]"	4.325	2.58E-02
ENSMUSG00000047534	Mis18bp1	MIS18 binding protein 1 [Source:MGI Symbol;Acc:MGI:2145099]	4.323	1.95E-04

ENSMUSG00000031262	Cenpi	centromere protein I [Source:MGI Symbol;Acc:MGI:2147897]	4.305	2.38E-03
ENSMUSG00000022033	Pbk	PDZ binding kinase [Source:MGI Symbol;Acc:MGI:1289156]	4.283	3.54E-03
ENSMUSG00000111271	Gm48127	"predicted gene, 48127 [Source:MGI Symbol;Acc:MGI:6097485]"	4.272	4.66E-02
ENSMUSG00000103475	Gm37697	"predicted gene, 37697 [Source:MGI Symbol;Acc:MGI:5610925]"	4.255	1.20E-02
ENSMUSG00000024672	Ms4a7	"membrane-spanning 4-domains, subfamily A, member 7 [Source:MGI Symbol;Acc:MGI:1918846]"	4.254	5.77E-16
ENSMUSG00000034040	Galnt17	polypeptide N-acetylgalactosaminyltransferase 17 [Source:MGI Symbol;Acc:MGI:2137594]	4.252	4.94E-02
ENSMUSG00000030162	Olr1	oxidized low density lipoprotein (lectin-like) receptor 1 [Source:MGI Symbol;Acc:MGI:1261434]	4.245	5.81E-04
ENSMUSG00000020635	Fkbp1b	FK506 binding protein 1b [Source:MGI Symbol;Acc:MGI:1336205]	4.245	2.88E-02
ENSMUSG00000091194	Gm19840	"predicted gene, 19840 [Source:MGI Symbol;Acc:MGI:5012025]"	4.240	8.26E-03
ENSMUSG00000037466	Tedc1	tubulin epsilon and delta complex 1 [Source:MGI Symbol;Acc:MGI:2144738]	4.236	8.70E-03
ENSMUSG00000074677	Sirpb1c	signal-regulatory protein beta 1C [Source:MGI Symbol;Acc:MGI:3807521]	4.218	1.89E-05
ENSMUSG00000115067	Dpep2	dipeptidase 2 [Source:MGI Symbol;Acc:MGI:2442042]	4.210	3.90E-02
ENSMUSG00000050022	Amz1	archaelysin family metallopeptidase 1 [Source:MGI Symbol;Acc:MGI:2442258]	4.193	1.03E-04
ENSMUSG00000020805	Slc13a5	"solute carrier family 13 (sodium-dependent citrate transporter), member 5 [Source:MGI Symbol;Acc:MGI:3037150]"	4.193	2.91E-06
ENSMUSG00000030346	Rad51ap1	RAD51 associated protein 1 [Source:MGI Symbol;Acc:MGI:1098224]	4.185	3.18E-02
ENSMUSG00000020901	Pik3r5	phosphoinositide-3-kinase regulatory subunit 5 [Source:MGI Symbol;Acc:MGI:2443588]	4.180	6.35E-11
ENSMUSG00000074999	Gm10797	predicted gene 10797 [Source:MGI Symbol;Acc:MGI:3642860]	4.180	2.16E-02
ENSMUSG00000025014	Dntt	"deoxynucleotidyltransferase, terminal [Source:MGI Symbol;Acc:MGI:98659]"	4.175	1.88E-02
ENSMUSG00000037474	Dtl	denticleless E3 ubiquitin protein ligase [Source:MGI Symbol;Acc:MGI:1924093]	4.175	8.83E-15
ENSMUSG00000102721	A630081D01Rik	RIKEN cDNA A630081D01 gene [Source:MGI Symbol;Acc:MGI:2138649]	4.175	2.03E-09
ENSMUSG00000021208	Ifi27l2b	"interferon, alpha-inducible protein 27 like 2B [Source:MGI Symbol;Acc:MGI:1916390]"	4.145	3.39E-13
ENSMUSG00000021268	Meg3	maternally expressed 3 [Source:MGI Symbol;Acc:MGI:1202886]	4.140	3.03E-34
ENSMUSG00000043644	0610009L18Rik	RIKEN cDNA 0610009L18 gene [Source:MGI Symbol;Acc:MGI:1914088]	4.134	2.04E-02
ENSMUSG00000045679	Pqlc3	PQ loop repeat containing [Source:MGI Symbol;Acc:MGI:2444067]	4.132	7.91E-04
ENSMUSG00000029130	Rnf32	ring finger protein 32 [Source:MGI Symbol;Acc:MGI:1861747]	4.126	3.13E-02
ENSMUSG00000020256	Aldh1l2	"aldehyde dehydrogenase 1 family, member L2 [Source:MGI Symbol;Acc:MGI:2444680]"	4.114	3.54E-03
ENSMUSG00000032080	Apoa4	apolipoprotein A-IV [Source:MGI Symbol;Acc:MGI:88051]	4.103	1.58E-70
ENSMUSG00000016200	Syt14	synaptotagmin XIV [Source:MGI Symbol;Acc:MGI:2444490]	4.089	2.73E-02
ENSMUSG00000108297	Gm44167	"predicted gene, 44167 [Source:MGI Symbol;Acc:MGI:5690559]"	4.084	3.03E-04
ENSMUSG00000102657	Gm37899	"predicted gene, 37899 [Source:MGI Symbol;Acc:MGI:5611127]"	4.084	7.98E-21
ENSMUSG00000050010	Shisa3	shisa family member 3 [Source:MGI Symbol;Acc:MGI:3041225]	4.071	2.31E-03
ENSMUSG00000108053	Gm43890	"predicted gene, 43890 [Source:MGI Symbol;Acc:MGI:5690282]"	4.061	2.55E-02
ENSMUSG00000103390	Gm38301	"predicted gene, 38301 [Source:MGI Symbol;Acc:MGI:5611529]"	4.060	2.72E-02
ENSMUSG00000105050	Gm42482	predicted gene 42482 [Source:MGI Symbol;Acc:MGI:5662619]	4.041	2.57E-06
ENSMUSG00000029822	Osbpl3	oxysterol binding protein-like 3 [Source:MGI Symbol;Acc:MGI:1918970]	4.039	1.88E-14
ENSMUSG00000055407	Map6	microtubule-associated protein 6 [Source:MGI Symbol;Acc:MGI:1201690]	4.031	1.88E-02
ENSMUSG00000118325	Gm50324	"predicted gene, 50324 [Source:MGI Symbol;Acc:MGI:6303187]"	4.029	2.96E-03
ENSMUSG00000039629	Strip2	striatin interacting protein 2 [Source:MGI Symbol;Acc:MGI:2444363]	4.028	4.85E-03
ENSMUSG00000056888	Glipr1	GLI pathogenesis-related 1 (glioma) [Source:MGI Symbol;Acc:MGI:1920940]	4.003	5.80E-05
ENSMUSG00000087221	BC037032	cDNA Sequence BC037032 [Source:MGI Symbol;Acc:MGI:3040924]	4.001	4.89E-02
ENSMUSG00000115771	Gm49312	"predicted gene, 49312 [Source:MGI Symbol;Acc:MGI:6118817]"	3.997	4.68E-02
ENSMUSG00000102813	Gm37795	"predicted gene, 37795 [Source:MGI Symbol;Acc:MGI:5611023]"	3.995	2.03E-06
ENSMUSG00000040675	Mthfd1l	methylenetetrahydrofolate dehydrogenase (NADP+ dependent) 1-like [Source:MGI Symbol;Acc:MGI:1924836]	3.990	1.20E-06
ENSMUSG00000045775	Slc16a5	"solute carrier family 16 (monocarboxylic acid transporters), member 5 [Source:MGI Symbol;Acc:MGI:2443515]"	3.981	1.08E-12
ENSMUSG00000103197	Gm37642	"predicted gene, 37642 [Source:MGI Symbol;Acc:MGI:5610870]"	3.981	2.54E-05
ENSMUSG00000025938	Slco5a1	"solute carrier organic anion transporter family, member 5A1 [Source:MGI Symbol;Acc:MGI:2443431]"	3.974	4.34E-03
ENSMUSG00000052336	Cx3cr1	chemokine (C-X3-C motif) receptor 1 [Source:MGI Symbol;Acc:MGI:1333815]	3.970	7.39E-11
ENSMUSG00000029915	Clec5a	"C-type lectin domain family 5, member a [Source:MGI Symbol;Acc:MGI:1345151]"	3.965	2.63E-06
ENSMUSG00000108597	Gm44708	predicted gene 44708 [Source:MGI Symbol;Acc:MGI:5753284]	3.964	4.10E-02
ENSMUSG00000055639	Dach1	dachshund family transcription factor 1 [Source:MGI Symbol;Acc:MGI:1277991]	3.957	5.18E-03

ENSMUSG00000017167	Cntnap1	contactin associated protein-like 1 [Source:MGI Symbol;Acc:MGI:1858201]	3.951	6.44E-09
ENSMUSG00000101268	2010310C07Rik	RIKEN cDNA 2010310C07 gene [Source:MGI Symbol;Acc:MGI:1919381]	3.937	5.51E-04
ENSMUSG00000037944	Ccr7	chemokine (C-C motif) receptor 7 [Source:MGI Symbol;Acc:MGI:103011]	3.932	6.03E-03
ENSMUSG00000072980	Oip5	Opa interacting protein 5 [Source:MGI Symbol;Acc:MGI:1917895]	3.928	7.03E-03
ENSMUSG00000034023	Fancd2	"Fanconi anemia, complementation group D2 [Source:MGI Symbol;Acc:MGI:2448480]"	3.922	1.18E-03
ENSMUSG00000067276	Capn6	calpain 6 [Source:MGI Symbol;Acc:MGI:1100850]	3.914	1.78E-06
ENSMUSG00000112980	D430020J02Rik	RIKEN cDNA D430020J02 gene [Source:MGI Symbol;Acc:MGI:2442237]	3.912	7.15E-04
ENSMUSG00000031506	Ptpn7	"protein tyrosine phosphatase, non-receptor type 7 [Source:MGI Symbol;Acc:MGI:2156893]"	3.910	4.31E-05
ENSMUSG00000032122	Slc37a2	"solute carrier family 37 (glycerol-3-phosphate transporter), member 2 [Source:MGI Symbol;Acc:MGI:1929693]"	3.898	3.57E-23
ENSMUSG00000021613	Hapln1	hyaluronan and proteoglycan link protein 1 [Source:MGI Symbol;Acc:MGI:1337006]	3.896	1.18E-07
ENSMUSG00000104488	Gm38062	"predicted gene, 38062 [Source:MGI Symbol;Acc:MGI:5611290]"	3.893	1.08E-05
ENSMUSG00000046223	Plaur	"plasminogen activator, urokinase receptor [Source:MGI Symbol;Acc:MGI:97612]"	3.887	4.55E-06
ENSMUSG00000003352	Cacnb3	"calcium channel, voltage-dependent, beta 3 subunit [Source:MGI Symbol;Acc:MGI:103307]"	3.885	2.31E-02
ENSMUSG00000062991	Nrg1	neuregulin 1 [Source:MGI Symbol;Acc:MGI:96083]	3.878	3.61E-17
ENSMUSG00000016763	Scube1	"signal peptide, CUB domain, EGF-like 1 [Source:MGI Symbol;Acc:MGI:1890616]"	3.867	1.60E-03
ENSMUSG00000087606	Gm16121	predicted gene 16121 [Source:MGI Symbol;Acc:MGI:3802131]	3.866	6.02E-03
ENSMUSG00000057465	Saa2	serum amyloid A 2 [Source:MGI Symbol;Acc:MGI:98222]	3.851	2.70E-15
ENSMUSG00000020399	Havcr2	hepatitis A virus cellular receptor 2 [Source:MGI Symbol;Acc:MGI:2159682]	3.850	8.87E-04
ENSMUSG00000035439	Haus8	"4HAUS augmin-like complex, subunit 8 [Source:MGI Symbol;Acc:MGI:1923728]"	3.845	1.51E-10
ENSMUSG00000027408	Cpxm1	carboxypeptidase X 1 (M14 family) [Source:MGI Symbol;Acc:MGI:1934569]	3.845	8.83E-04
ENSMUSG00000031150	Ccdc120	coiled-coil domain containing 120 [Source:MGI Symbol;Acc:MGI:1859619]	3.842	1.31E-09
ENSMUSG00000022876	Samsn1	"SAM domain, SH3 domain and nuclear localization signals, 1 [Source:MGI Symbol;Acc:MGI:1914992]"	3.838	2.96E-04
ENSMUSG00000033777	Tlr13	toll-like receptor 13 [Source:MGI Symbol;Acc:MGI:3045213]	3.832	7.92E-25
ENSMUSG00000099974	Bcl2a1d	B cell leukemia/lymphoma 2 related protein A1d [Source:MGI Symbol;Acc:MGI:1278325]	3.830	2.49E-04
ENSMUSG00000105637	Gm42480	predicted gene 42480 [Source:MGI Symbol;Acc:MGI:5662617]	3.827	1.05E-06
ENSMUSG00000054932	Afp	alpha fetoprotein [Source:MGI Symbol;Acc:MGI:87951]	3.826	2.24E-02
ENSMUSG00000103364	Gm38157	"predicted gene, 38157 [Source:MGI Symbol;Acc:MGI:5611385]"	3.825	1.48E-03
ENSMUSG00000087613	Gm13855	predicted gene 13855 [Source:MGI Symbol;Acc:MGI:3652198]	3.824	3.89E-02
ENSMUSG00000092247	Gm20426	predicted gene 20426 [Source:MGI Symbol;Acc:MGI:5141891]	3.824	4.18E-02
ENSMUSG00000105940	Gm42635	predicted gene 42635 [Source:MGI Symbol;Acc:MGI:5662772]	3.820	9.92E-10
ENSMUSG00000049130	C5ar1	complement component 5a receptor 1 [Source:MGI Symbol;Acc:MGI:88232]	3.819	1.49E-13
ENSMUSG00000060568	Fam78b	"family with sequence similarity 78, member B [Source:MGI Symbol;Acc:MGI:2443050]"	3.811	3.22E-03
ENSMUSG00000102349	Gm37376	"predicted gene, 37376 [Source:MGI Symbol;Acc:MGI:5610604]"	3.798	7.71E-03
ENSMUSG00000043157	Arl11	ADP-ribosylation factor-like 11 [Source:MGI Symbol;Acc:MGI:2444054]	3.796	6.79E-04
ENSMUSG00000031779	Ccl22	chemokine (C-C motif) ligand 22 [Source:MGI Symbol;Acc:MGI:1306779]	3.779	5.49E-04
ENSMUSG00000057948	Unc13d	unc-13 homolog D [Source:MGI Symbol;Acc:MGI:1917700]	3.777	3.38E-02
ENSMUSG00000025351	Cd63	CD63 antigen [Source:MGI Symbol;Acc:MGI:99529]	3.768	5.20E-09
ENSMUSG00000028364	Tnc	tenascin C [Source:MGI Symbol;Acc:MGI:101922]	3.766	1.81E-06
ENSMUSG00000032750	Gab3	growth factor receptor bound protein 2-associated protein 3 [Source:MGI Symbol;Acc:MGI:2387324]	3.758	3.22E-03
ENSMUSG00000107603	Gm43921	"predicted gene, 43921 [Source:MGI Symbol;Acc:MGI:5690313]"	3.756	1.77E-06
ENSMUSG00000035385	Ccl2	chemokine (C-C motif) ligand 2 [Source:MGI Symbol;Acc:MGI:98259]	3.754	2.81E-04
ENSMUSG00000028664	Ephb2	Eph receptor B2 [Source:MGI Symbol;Acc:MGI:99611]	3.753	1.37E-02
ENSMUSG00000033949	Trim36	tripartite motif-containing 36 [Source:MGI Symbol;Acc:MGI:106264]	3.744	1.17E-04
ENSMUSG00000108693	Gm45153	predicted gene 45153 [Source:MGI Symbol;Acc:MGI:5753729]	3.742	7.57E-03
ENSMUSG00000024935	Slc1a1	"solute carrier family 1 (neuronal/epithelial high affinity glutamate transporter, system Xag), member 1 [Source:MGI Symbol;Acc:MGI:105083]"	3.742	2.29E-02
ENSMUSG00000089769	Gm16574	predicted gene 16574 [Source:MGI Symbol;Acc:MGI:4414994]	3.741	1.60E-02
ENSMUSG00000023087	Noct	nocturnin [Source:MGI Symbol;Acc:MGI:109382]	3.740	3.97E-27
ENSMUSG00000097451	Rian	RNA imprinted and accumulated in nucleus [Source:MGI Symbol;Acc:MGI:1922995]	3.731	8.87E-23
ENSMUSG00000103948	4930594C11Rik	RIKEN cDNA 4930594C11 gene [Source:MGI Symbol;Acc:MGI:1924883]	3.726	1.87E-02
ENSMUSG00000055866	Per2	period circadian clock 2 [Source:MGI Symbol;Acc:MGI:1195265]	3.723	5.50E-28

ENSMUSG00000106337	Gm42478	predicted gene 42478 [Source:MGI Symbol;Acc:MGI:5662615]	3.722	1.14E-02
ENSMUSG00000111709	Gm3776	predicted gene 3776 [Source:MGI Symbol;Acc:MGI:3826440]	3.720	3.20E-13
ENSMUSG00000104002	Gm38336	"predicted gene, 38336 [Source:MGI Symbol;Acc:MGI:5611564]"	3.720	1.71E-02
ENSMUSG00000002055	Spag5	sperm associated antigen 5 [Source:MGI Symbol;Acc:MGI:1927470]	3.710	2.80E-02
ENSMUSG00000069515	Lyz1	lysozyme 1 [Source:MGI Symbol;Acc:MGI:96902]	3.699	3.30E-04
ENSMUSG00000029919	Hpgds	hematopoietic prostaglandin D synthase [Source:MGI Symbol;Acc:MGI:1859384]	3.696	3.95E-19
ENSMUSG00000045382	Cxcr4	chemokine (C-X-C motif) receptor 4 [Source:MGI Symbol;Acc:MGI:109563]	3.693	3.91E-10
ENSMUSG00000108317	Gm44663	predicted gene 44663 [Source:MGI Symbol;Acc:MGI:5753239]	3.684	1.67E-04
ENSMUSG00000115150	Gm10370	predicted gene 10370 [Source:MGI Symbol;Acc:MGI:3642773]	3.683	1.36E-08
ENSMUSG00000032010	Usp2	ubiquitin specific peptidase 2 [Source:MGI Symbol;Acc:MGI:1858178]	3.682	1.31E-15
ENSMUSG00000104938	Gm42840	predicted gene 42840 [Source:MGI Symbol;Acc:MGI:5662977]	3.672	4.75E-03
ENSMUSG00000048612	Myof	myoferlin [Source:MGI Symbol;Acc:MGI:1919192]	3.670	1.77E-21
ENSMUSG00000024300	Myo1f	myosin IF [Source:MGI Symbol;Acc:MGI:107711]	3.656	2.55E-28
ENSMUSG00000102142	Gm26930	"predicted gene, 26930 [Source:MGI Symbol;Acc:MGI:5504045]"	3.654	9.93E-03
ENSMUSG00000028702	Rad54l	RAD54 like (S. cerevisiae) [Source:MGI Symbol;Acc:MGI:894697]	3.651	3.28E-04
ENSMUSG00000006435	Neurl1a	neuralized E3 ubiquitin protein ligase 1A [Source:MGI Symbol;Acc:MGI:1334263]	3.646	6.65E-03
ENSMUSG00000098318	Lockd	lncRNA downstream of Cdkn1b [Source:MGI Symbol;Acc:MGI:1915081]	3.646	2.09E-02
ENSMUSG00000024529	Lox	lysyl oxidase [Source:MGI Symbol;Acc:MGI:96817]	3.643	1.08E-07
ENSMUSG00000039942	Ptger4	prostaglandin E receptor 4 (subtype EP4) [Source:MGI Symbol;Acc:MGI:104311]	3.642	1.00E-03
ENSMUSG00000030786	Itgam	integrin alpha M [Source:MGI Symbol;Acc:MGI:96607]	3.642	2.81E-14
ENSMUSG00000078122	F630028O10Rik	RIKEN cDNA F630028O10 gene [Source:MGI Symbol;Acc:MGI:3641813]	3.627	1.62E-38
ENSMUSG00000082956	Naip3	"NLR family, apoptosis inhibitory protein 3 [Source:MGI Symbol;Acc:MGI:1298225]"	3.617	4.83E-02
ENSMUSG00000001506	Col1a1	"collagen, type I, alpha 1 [Source:MGI Symbol;Acc:MGI:88467]"	3.600	8.28E-04
ENSMUSG00000097715	Gpr137b-ps	"G protein-coupled receptor 137B, pseudogene [Source:MGI Symbol;Acc:MGI:3710533]"	3.599	6.68E-18
ENSMUSG00000102037	Bcl2a1a	B cell leukemia/lymphoma 2 related protein A1a [Source:MGI Symbol;Acc:MGI:102687]	3.597	9.89E-04
ENSMUSG00000103885	Gm37006	"predicted gene, 37006 [Source:MGI Symbol;Acc:MGI:5610234]"	3.592	8.69E-05
ENSMUSG00000049103	Ccr2	chemokine (C-C motif) receptor 2 [Source:MGI Symbol;Acc:MGI:106185]	3.589	2.09E-16
ENSMUSG00000063430	Wscd2	WSC domain containing 2 [Source:MGI Symbol;Acc:MGI:2445030]	3.584	3.56E-02
ENSMUSG00000105986	Gm43065	predicted gene 43065 [Source:MGI Symbol;Acc:MGI:5663202]	3.573	1.09E-04
ENSMUSG00000001228	Uhrf1	"ubiquitin-like, containing PHD and RING finger domains, 1 [Source:MGI Symbol;Acc:MGI:1338889]"	3.568	2.91E-08
ENSMUSG00000116832	Gm49658	"predicted gene, 49658 [Source:MGI Symbol;Acc:MGI:6215094]"	3.568	1.58E-07
ENSMUSG00000031558	Slit2	slit guidance ligand 2 [Source:MGI Symbol;Acc:MGI:1315205]	3.566	1.38E-02
ENSMUSG00000105356	Gm42603	predicted gene 42603 [Source:MGI Symbol;Acc:MGI:5662740]	3.564	1.14E-08
ENSMUSG00000022667	Cd200r1	CD200 receptor 1 [Source:MGI Symbol;Acc:MGI:1889024]	3.562	1.23E-09
ENSMUSG00000113669	Gm36723	"predicted gene, 36723 [Source:MGI Symbol;Acc:MGI:5595882]"	3.560	3.79E-03
ENSMUSG00000070348	Ccnd1	cyclin D1 [Source:MGI Symbol;Acc:MGI:88313]	3.557	2.09E-40
ENSMUSG00000104291	A130071D04Rik	RIKEN cDNA A130071D04 gene [Source:MGI Symbol;Acc:MGI:2444749]	3.555	8.16E-48
ENSMUSG00000105711	Gm42598	predicted gene 42598 [Source:MGI Symbol;Acc:MGI:5662735]	3.551	4.81E-03
ENSMUSG00000034612	Chst11	carbohydrate sulfotransferase 11 [Source:MGI Symbol;Acc:MGI:1927166]	3.551	1.80E-04
ENSMUSG00000103748	Gm38243	"predicted gene, 38243 [Source:MGI Symbol;Acc:MGI:5611471]"	3.546	4.18E-02
ENSMUSG00000015316	Slamf1	signaling lymphocytic activation molecule family member 1 [Source:MGI Symbol;Acc:MGI:1351314]	3.538	1.39E-02
ENSMUSG00000025804	Ccr1	chemokine (C-C motif) receptor 1 [Source:MGI Symbol;Acc:MGI:104618]	3.537	3.86E-04
ENSMUSG00000002204	Napsa	napsin A aspartic peptidase [Source:MGI Symbol;Acc:MGI:109365]	3.534	1.43E-03
ENSMUSG00000070323	Mmp27	matrix metallopeptidase 27 [Source:MGI Symbol;Acc:MGI:3039232]	3.533	5.74E-03
ENSMUSG00000004952	Rasa4	RAS p21 protein activator 4 [Source:MGI Symbol;Acc:MGI:1858600]	3.532	9.80E-10
ENSMUSG00000105160	A530030E21Rik	RIKEN cDNA A530030E21 gene [Source:MGI Symbol;Acc:MGI:2444617]	3.530	1.35E-04
ENSMUSG00000022952	Runx1	runt related transcription factor 1 [Source:MGI Symbol;Acc:MGI:99852]	3.512	6.18E-21
ENSMUSG00000113119	Gm48883	"predicted gene, 48883 [Source:MGI Symbol;Acc:MGI:6098639]"	3.510	4.32E-02
ENSMUSG00000104709	Gm42484	predicted gene 42484 [Source:MGI Symbol;Acc:MGI:5662621]	3.499	3.72E-04
ENSMUSG00000097352	C920009B18Rik	RIKEN cDNA C920009B18 gene [Source:MGI Symbol;Acc:MGI:3583961]	3.498	9.42E-08

ENSMUSG00000109243	Gm45867	predicted gene 45867 [Source:MGI Symbol;Acc:MGI:5804982]	3.496	2.81E-07
ENSMUSG00000089929	Bcl2a1b	B cell leukemia/lymphoma 2 related protein A1b [Source:MGI Symbol;Acc:MGI:1278326]	3.495	1.76E-06
ENSMUSG00000017499	Cdc6	cell division cycle 6 [Source:MGI Symbol;Acc:MGI:1345150]	3.483	2.87E-03
ENSMUSG00000042041	2010003K11Rik	RIKEN cDNA 2010003K11 gene [Source:MGI Symbol;Acc:MGI:1917111]	3.468	7.14E-19
ENSMUSG00000027323	Rad51	RAD51 recombinase [Source:MGI Symbol;Acc:MGI:97890]	3.466	1.21E-05
ENSMUSG00000030717	Nupr1	nuclear protein transcription regulator 1 [Source:MGI Symbol;Acc:MGI:1891834]	3.459	2.74E-03
ENSMUSG00000034755	Pcdh11x	protocadherin 11 X-linked [Source:MGI Symbol;Acc:MGI:2442849]	3.444	9.24E-03
ENSMUSG00000036446	Lum	lumican [Source:MGI Symbol;Acc:MGI:109347]	3.440	1.48E-10
ENSMUSG00000054342	Kcnn4	"potassium intermediate/small conductance calcium-activated channel, subfamily N, member 4 [Source:MGI Symbol;Acc:MGI:1277957]"	3.435	4.37E-02
ENSMUSG00000021306	Gpr137b	G protein-coupled receptor 137B [Source:MGI Symbol;Acc:MGI:1891463]	3.435	1.49E-22
ENSMUSG00000091900	Gm4353	predicted gene 4353 [Source:MGI Symbol;Acc:MGI:3782538]	3.434	2.04E-02
ENSMUSG00000059493	Nhs	NHS actin remodeling regulator [Source:MGI Symbol;Acc:MGI:2684894]	3.431	1.07E-02
ENSMUSG00000112756	Gm48014	"predicted gene, 48014 [Source:MGI Symbol;Acc:MGI:6097321]"	3.431	2.07E-05
ENSMUSG00000049307	Fut4	fucosyltransferase 4 [Source:MGI Symbol;Acc:MGI:95594]	3.427	4.02E-04
ENSMUSG00000084964	Gm15503	predicted gene 15503 [Source:MGI Symbol;Acc:MGI:3782950]	3.425	2.45E-02
ENSMUSG00000002668	Dennd1c	DENN/MADD domain containing 1C [Source:MGI Symbol;Acc:MGI:1918035]	3.424	4.62E-06
ENSMUSG00000034855	Cxcl10	chemokine (C-X-C motif) ligand 10 [Source:MGI Symbol;Acc:MGI:1352450]	3.423	4.39E-12
ENSMUSG00000025001	Hells	"helicase, lymphoid specific [Source:MGI Symbol;Acc:MGI:106209]"	3.419	3.59E-11
ENSMUSG00000004709	Cd244a	CD244 molecule A [Source:MGI Symbol;Acc:MGI:109294]	3.418	1.34E-03
ENSMUSG00000053101	Gpr141	G protein-coupled receptor 141 [Source:MGI Symbol;Acc:MGI:2672983]	3.417	1.45E-07
ENSMUSG00000113601	Gm48735	"predicted gene, 48735 [Source:MGI Symbol;Acc:MGI:6098399]"	3.412	2.07E-03
ENSMUSG00000025648	Pfkfb4	"6-phosphofructo-2-kinase/fructose-2,6-biphosphatase 4 [Source:MGI Symbol;Acc:MGI:2687284]"	3.403	1.20E-06
ENSMUSG00000104821	Gm42481	predicted gene 42481 [Source:MGI Symbol;Acc:MGI:5662618]	3.403	3.52E-05
ENSMUSG00000057969	Sema3b	"sema domain, immunoglobulin domain (Ig), short basic domain, secreted, (semaphorin) 3B [Source:MGI Symbol;Acc:MGI:107561]"	3.402	2.94E-03
ENSMUSG00000052516	Robo2	roundabout guidance receptor 2 [Source:MGI Symbol;Acc:MGI:1890110]	3.400	1.61E-14
ENSMUSG00000105431	Gm42640	predicted gene 42640 [Source:MGI Symbol;Acc:MGI:5662777]	3.397	3.65E-04
ENSMUSG00000040552	C3ar1	complement component 3a receptor 1 [Source:MGI Symbol;Acc:MGI:1097680]	3.394	5.69E-24
ENSMUSG00000022102	Dok2	docking protein 2 [Source:MGI Symbol;Acc:MGI:1332623]	3.392	2.87E-06
ENSMUSG00000098202	B830012L14Rik	RIKEN cDNA B830012L14 gene [Source:MGI Symbol;Acc:MGI:2443332]	3.384	6.16E-19
ENSMUSG00000028359	Orm3	orosomucoid 3 [Source:MGI Symbol;Acc:MGI:97445]	3.382	1.13E-05
ENSMUSG00000021451	Sema4d	"sema domain, immunoglobulin domain (Ig), transmembrane domain (TM) and short cytoplasmic domain, (semaphorin) 4D [Source:MGI Symbol;Acc:MGI:109244]"	3.381	1.28E-12
ENSMUSG00000107136	Gm42466	predicted gene 42466 [Source:MGI Symbol;Acc:MGI:5662603]	3.378	3.35E-03
ENSMUSG00000111116	Gm48065	"predicted gene, 48065 [Source:MGI Symbol;Acc:MGI:6097392]"	3.377	2.91E-02
ENSMUSG00000025909	Sntg1	"syntrophin, gamma 1 [Source:MGI Symbol;Acc:MGI:1918346]"	3.376	8.27E-07
ENSMUSG00000049493	Pls1	plastin 1 (I-isoform) [Source:MGI Symbol;Acc:MGI:104809]	3.375	2.45E-04
ENSMUSG00000030657	Xylt1	xylosyltransferase 1 [Source:MGI Symbol;Acc:MGI:2451073]	3.371	8.61E-07
ENSMUSG00000030276	Ttll3	"tubulin tyrosine ligase-like family, member 3 [Source:MGI Symbol;Acc:MGI:2141418]"	3.370	2.51E-02
ENSMUSG00000038843	Gcnt1	"glucosaminyl (N-acetyl) transferase 1, core 2 [Source:MGI Symbol;Acc:MGI:95676]"	3.369	4.74E-06
ENSMUSG00000104145	D130019J16Rik	RIKEN cDNA D130019J16 gene [Source:MGI Symbol;Acc:MGI:2443523]	3.361	2.11E-04
ENSMUSG00000097391	Mirg	miRNA containing gene [Source:MGI Symbol;Acc:MGI:3781106]	3.360	6.14E-05
ENSMUSG00000061132	Blnk	B cell linker [Source:MGI Symbol;Acc:MGI:96878]	3.350	1.30E-08
ENSMUSG00000023067	Cdkn1a	cyclin-dependent kinase inhibitor 1A (P21) [Source:MGI Symbol;Acc:MGI:104556]	3.339	7.21E-06
ENSMUSG00000040711	Sh3pxd2b	SH3 and PX domains 2B [Source:MGI Symbol;Acc:MGI:2442062]	3.336	1.04E-15
ENSMUSG00000107865	Gm44417	"predicted gene, 44417 [Source:MGI Symbol;Acc:MGI:5690809]"	3.334	3.01E-02
ENSMUSG00000098858	Mir6541	microRNA 6541 [Source:MGI Symbol;Acc:MGI:5530693]	3.334	3.24E-02
ENSMUSG00000085786	Gm15987	predicted gene 15987 [Source:MGI Symbol;Acc:MGI:3801849]	3.330	7.92E-04
ENSMUSG00000029082	Bst1	bone marrow stromal cell antigen 1 [Source:MGI Symbol;Acc:MGI:105370]	3.327	3.02E-02
ENSMUSG00000007805	Twist2	twist basic helix-loop-helix transcription factor 2 [Source:MGI Symbol;Acc:MGI:104685]	3.317	2.28E-02
ENSMUSG00000103883	Gm37010	"predicted gene, 37010 [Source:MGI Symbol;Acc:MGI:5610238]"	3.311	3.29E-06
ENSMUSG00000058427	Cxcl2	chemokine (C-X-C motif) ligand 2 [Source:MGI Symbol;Acc:MGI:1340094]	3.310	4.94E-03

ENSMUSG00000078700	D030028A08Rik	RIKEN cDNA D030028A08 gene [Source:MGI Symbol;Acc:MGI:2441931]	3.310	3.63E-04
ENSMUSG00000049985	Ankrd55	ankyrin repeat domain 55 [Source:MGI Symbol;Acc:MGI:1924568]	3.307	1.25E-03
ENSMUSG00000003882	Il7r	interleukin 7 receptor [Source:MGI Symbol;Acc:MGI:96562]	3.300	1.51E-08
ENSMUSG00000094350	Gm10931	predicted gene 10931 [Source:MGI Symbol;Acc:MGI:3779142]	3.288	7.10E-03
ENSMUSG00000036768	Kif15	kinesin family member 15 [Source:MGI Symbol;Acc:MGI:1098258]	3.281	3.58E-04
ENSMUSG00000019876	Pkib	"protein kinase inhibitor beta, cAMP dependent, testis specific [Source:MGI Symbol;Acc:MGI:101937]"	3.278	2.05E-11
ENSMUSG00000105750	Gm43130	predicted gene 43130 [Source:MGI Symbol;Acc:MGI:5663267]	3.277	7.83E-03
ENSMUSG00000016458	Wt1	Wilms tumor 1 homolog [Source:MGI Symbol;Acc:MGI:98968]	3.274	1.23E-02
ENSMUSG00000039153	Runx2	runt related transcription factor 2 [Source:MGI Symbol;Acc:MGI:99829]	3.270	7.84E-10
ENSMUSG00000104615	Gm43804	predicted gene 43804 [Source:MGI Symbol;Acc:MGI:5663941]	3.267	2.89E-02
ENSMUSG00000113687	Gm49703	"predicted gene, 49703 [Source:MGI Symbol;Acc:MGI:6215164]"	3.266	1.72E-02
ENSMUSG00000052776	Oas1a	2'-5' oligoadenylate synthetase 1A [Source:MGI Symbol;Acc:MGI:2180860]	3.265	2.73E-07
ENSMUSG00000000244	Tspan32	tetraspanin 32 [Source:MGI Symbol;Acc:MGI:1350360]	3.260	2.26E-03
ENSMUSG00000038179	Slamf7	SLAM family member 7 [Source:MGI Symbol;Acc:MGI:1922595]	3.252	1.73E-11
ENSMUSG00000078190	Dnm3os	"dynamin 3, opposite strand [Source:MGI Symbol;Acc:MGI:3052332]"	3.247	1.22E-22
ENSMUSG00000028613	Lrp8	"low density lipoprotein receptor-related protein 8, apolipoprotein e receptor [Source:MGI Symbol;Acc:MGI:1340044]"	3.246	1.12E-02
ENSMUSG00000037594	Clba1	clathrin binding box of aftiphilin containing 1 [Source:MGI Symbol;Acc:MGI:2443738]	3.243	3.03E-02
ENSMUSG00000000204	Slfn4	schlafen 4 [Source:MGI Symbol;Acc:MGI:1329010]	3.243	1.81E-03
ENSMUSG00000037913	Tmem156	transmembrane protein 156 [Source:MGI Symbol;Acc:MGI:2685292]	3.241	9.75E-03
ENSMUSG00000027397	Slc20a1	"solute carrier family 20, member 1 [Source:MGI Symbol;Acc:MGI:108392]"	3.236	3.58E-45
ENSMUSG00000020974	Pole2	"polymerase (DNA directed), epsilon 2 (p59 subunit) [Source:MGI Symbol;Acc:MGI:1197514]"	3.234	5.40E-03
ENSMUSG00000032690	Oas2	2'-5' oligoadenylate synthetase 2 [Source:MGI Symbol;Acc:MGI:2180852]	3.227	3.35E-08
ENSMUSG00000108455	Gm44745	predicted gene 44745 [Source:MGI Symbol;Acc:MGI:5753321]	3.225	4.66E-07
ENSMUSG00000026395	Ptprc	"protein tyrosine phosphatase, receptor type, C [Source:MGI Symbol;Acc:MGI:97810]"	3.222	2.38E-53
ENSMUSG00000103163	Gm38146	"predicted gene, 38146 [Source:MGI Symbol;Acc:MGI:5611374]"	3.212	3.53E-03
ENSMUSG00000053063	Clec12a	"C-type lectin domain family 12, member a [Source:MGI Symbol;Acc:MGI:3040968]"	3.211	4.91E-14
ENSMUSG00000031963	Bmper	BMP-binding endothelial regulator [Source:MGI Symbol;Acc:MGI:1920480]	3.208	2.62E-02
ENSMUSG00000027699	Ect2	ect2 oncogene [Source:MGI Symbol;Acc:MGI:95281]	3.196	1.78E-05
ENSMUSG00000024883	Rin1	Ras and Rab interactor 1 [Source:MGI Symbol;Acc:MGI:2385695]	3.194	2.90E-02
ENSMUSG00000024659	Anxa1	annexin A1 [Source:MGI Symbol;Acc:MGI:96819]	3.193	2.95E-08
ENSMUSG00000106856	Gm43761	predicted gene 43761 [Source:MGI Symbol;Acc:MGI:5663898]	3.187	3.56E-02
ENSMUSG00000049580	Tsku	"tsukushi, small leucine rich proteoglycan [Source:MGI Symbol;Acc:MGI:2443855]"	3.184	4.45E-24
ENSMUSG00000078780	Gm5150	predicted gene 5150 [Source:MGI Symbol;Acc:MGI:3779469]	3.183	7.63E-03
ENSMUSG00000023905	Tnfrsf12a	"tumor necrosis factor receptor superfamily, member 12a [Source:MGI Symbol;Acc:MGI:1351484]"	3.181	8.75E-11
ENSMUSG00000023088	Abcc1	"ATP-binding cassette, sub-family C (CFTR/MRP), member 1 [Source:MGI Symbol;Acc:MGI:102676]"	3.177	3.83E-22
ENSMUSG00000085337	Gm15964	predicted gene 15964 [Source:MGI Symbol;Acc:MGI:3802003]	3.172	4.16E-02
ENSMUSG00000023913	Pla2g7	"phospholipase A2, group VII (platelet-activating factor acetylhydrolase, plasma) [Source:MGI Symbol;Acc:MGI:1351327]"	3.171	2.95E-13
ENSMUSG00000046591	Ticrr	TOPBP1-interacting checkpoint and replication regulator [Source:MGI Symbol;Acc:MGI:1924261]	3.168	3.16E-03
ENSMUSG00000043017	Ptgir	prostaglandin I receptor (IP) [Source:MGI Symbol;Acc:MGI:99535]	3.168	1.08E-03
ENSMUSG00000039193	Nlrc4	"NLR family, CARD domain containing 4 [Source:MGI Symbol;Acc:MGI:3036243]"	3.167	6.62E-05
ENSMUSG00000029484	Anxa3	annexin A3 [Source:MGI Symbol;Acc:MGI:1201378]	3.166	9.11E-10
ENSMUSG00000051998	Lax1	lymphocyte transmembrane adaptor 1 [Source:MGI Symbol;Acc:MGI:2443362]	3.165	3.90E-02
ENSMUSG00000053168	9030619P08Rik	RIKEN cDNA 9030619P08 gene [Source:MGI Symbol;Acc:MGI:3612405]	3.164	1.42E-10
ENSMUSG00000024679	Ms4a6d	"membrane-spanning 4-domains, subfamily A, member 6D [Source:MGI Symbol;Acc:MGI:1916024]"	3.163	3.14E-07
ENSMUSG00000030745	Il21r	interleukin 21 receptor [Source:MGI Symbol;Acc:MGI:1890475]	3.163	3.42E-03
ENSMUSG00000004707	Ly9	lymphocyte antigen 9 [Source:MGI Symbol;Acc:MGI:96885]	3.162	5.16E-06
ENSMUSG00000051504	Siglech	sialic acid binding Ig-like lectin H [Source:MGI Symbol;Acc:MGI:2443256]	3.160	3.33E-07
ENSMUSG00000029061	Mmp23	matrix metallopeptidase 23 [Source:MGI Symbol;Acc:MGI:1347361]	3.160	3.00E-02
ENSMUSG00000019982	Myb	myeloblastosis oncogene [Source:MGI Symbol;Acc:MGI:97249]	3.159	2.15E-02
ENSMUSG00000020493	Prr11	proline rich 11 [Source:MGI Symbol;Acc:MGI:2444496]	3.159	9.44E-05

ENSMUSG00000026875	Traf1	TNF receptor-associated factor 1 [Source:MGI Symbol;Acc:MGI:101836]	3.158	1.80E-04
ENSMUSG00000006219	Fblim1	filamin binding LIM protein 1 [Source:MGI Symbol;Acc:MGI:1921452]	3.152	3.33E-06
ENSMUSG00000031016	Wee1	WEE 1 homolog 1 (S. pombe) [Source:MGI Symbol;Acc:MGI:103075]	3.151	3.29E-19
ENSMUSG00000055254	Ntrk2	"neurotrophic tyrosine kinase, receptor, type 2 [Source:MGI Symbol;Acc:MGI:97384]"	3.150	6.27E-08
ENSMUSG00000112532	Gm36283	"predicted gene, 36283 [Source:MGI Symbol;Acc:MGI:5595442]"	3.148	2.12E-04
ENSMUSG00000020330	Hmmr	hyaluronan mediated motility receptor (RHAMM) [Source:MGI Symbol;Acc:MGI:104667]	3.148	2.98E-04
ENSMUSG00000026480	Ncf2	neutrophil cytosolic factor 2 [Source:MGI Symbol;Acc:MGI:97284]	3.145	3.87E-09
ENSMUSG00000022947	Cbr3	carbonyl reductase 3 [Source:MGI Symbol;Acc:MGI:1309992]	3.144	3.02E-03
ENSMUSG00000029661	Col1a2	"collagen, type I, alpha 2 [Source:MGI Symbol;Acc:MGI:88468]"	3.143	1.61E-03
ENSMUSG00000112478	Gm47761	"predicted gene, 47761 [Source:MGI Symbol;Acc:MGI:6096912]"	3.142	4.11E-02
ENSMUSG00000029108	Pcdh7	protocadherin 7 [Source:MGI Symbol;Acc:MGI:1860487]	3.142	1.16E-07
ENSMUSG00000109051	Gm44913	predicted gene 44913 [Source:MGI Symbol;Acc:MGI:5753489]	3.133	1.44E-04
ENSMUSG00000111394	Gm49759	"predicted gene, 49759 [Source:MGI Symbol;Acc:MGI:6215263]"	3.133	4.03E-12
ENSMUSG00000026981	Il1rn	interleukin 1 receptor antagonist [Source:MGI Symbol;Acc:MGI:96547]	3.132	6.53E-17
ENSMUSG00000060131	Atp8b4	"ATPase, class I, type 8B, member 4 [Source:MGI Symbol;Acc:MGI:1859664]"	3.131	1.43E-11
ENSMUSG00000024803	Ankrd1	ankyrin repeat domain 1 (cardiac muscle) [Source:MGI Symbol;Acc:MGI:1097717]	3.129	2.64E-02
ENSMUSG00000039457	Ppl	periplakin [Source:MGI Symbol;Acc:MGI:1194898]	3.128	6.53E-25
ENSMUSG00000043903	Zfp469	zinc finger protein 469 [Source:MGI Symbol;Acc:MGI:2684868]	3.125	2.34E-03
ENSMUSG00000051439	Cd14	CD14 antigen [Source:MGI Symbol;Acc:MGI:88318]	3.125	6.47E-08
ENSMUSG00000069516	Lyz2	lysozyme 2 [Source:MGI Symbol;Acc:MGI:96897]	3.125	1.64E-21
ENSMUSG00000027331	Knstrn	kinetochore-localized astrin/SPAG5 binding [Source:MGI Symbol;Acc:MGI:1289298]	3.123	1.77E-05
ENSMUSG00000025586	Cpeb1	cytoplasmic polyadenylation element binding protein 1 [Source:MGI Symbol;Acc:MGI:108442]	3.117	2.89E-08
ENSMUSG00000056130	Ticam2	toll-like receptor adaptor molecule 2 [Source:MGI Symbol;Acc:MGI:3040056]	3.116	2.34E-05
ENSMUSG00000047497	Adamts12	"a disintegrin-like and metallopeptidase (reprolysin type) with thrombospondin type 1 motif, 12 [Source:MGI Symbol;Acc:MGI:2146046]"	3.115	2.14E-08
ENSMUSG00000029530	Ccr9	chemokine (C-C motif) receptor 9 [Source:MGI Symbol;Acc:MGI:1341902]	3.115	1.04E-02
ENSMUSG00000079014	Serpina3i	"serine (or cysteine) peptidase inhibitor, clade A, member 3I [Source:MGI Symbol;Acc:MGI:2182841]"	3.113	4.80E-02
ENSMUSG00000105392	Gm42684	predicted gene 42684 [Source:MGI Symbol;Acc:MGI:5662821]	3.113	2.37E-03
ENSMUSG00000067656	Slc22a27	"solute carrier family 22, member 27 [Source:MGI Symbol;Acc:MGI:3042283]"	3.112	5.67E-04
ENSMUSG00000033730	Egr3	early growth response 3 [Source:MGI Symbol;Acc:MGI:1306780]	3.107	5.34E-03
ENSMUSG00000030047	Arhgap25	Rho GTPase activating protein 25 [Source:MGI Symbol;Acc:MGI:2443687]	3.107	1.26E-13
ENSMUSG00000003051	Elf3	E74-like factor 3 [Source:MGI Symbol;Acc:MGI:1101781]	3.107	1.90E-02
ENSMUSG00000005410	Mcm5	minichromosome maintenance complex component 5 [Source:MGI Symbol;Acc:MGI:103197]	3.102	3.00E-05
ENSMUSG00000020354	Sgcd	"sarcoglycan, delta (dystrophin-associated glycoprotein) [Source:MGI Symbol;Acc:MGI:1346525]"	3.102	8.14E-03
ENSMUSG00000056529	Ptafr	platelet-activating factor receptor [Source:MGI Symbol;Acc:MGI:106066]	3.102	1.85E-12
ENSMUSG00000020120	Plek	pleckstrin [Source:MGI Symbol;Acc:MGI:1860485]	3.101	8.13E-12
ENSMUSG00000048368	Omd	osteomodulin [Source:MGI Symbol;Acc:MGI:1350918]	3.099	2.64E-02
ENSMUSG00000017146	Brca1	"breast cancer 1, early onset [Source:MGI Symbol;Acc:MGI:104537]"	3.098	4.54E-05
ENSMUSG00000019850	Tnfaip3	"tumor necrosis factor, alpha-induced protein 3 [Source:MGI Symbol;Acc:MGI:1196377]"	3.097	2.33E-15
ENSMUSG00000113910	Gm48286	"predicted gene, 48286 [Source:MGI Symbol;Acc:MGI:6097722]"	3.095	5.11E-03
ENSMUSG00000106202	Gm43727	predicted gene 43727 [Source:MGI Symbol;Acc:MGI:5663864]	3.092	1.18E-04
ENSMUSG00000097113	Gm19705	"predicted gene, 19705 [Source:MGI Symbol;Acc:MGI:5011890]"	3.090	7.67E-04
ENSMUSG00000052688	Rab7b	"RAB7B, member RAS oncogene family [Source:MGI Symbol;Acc:MGI:2442295]"	3.089	5.50E-05
ENSMUSG00000020363	Gfpt2	glutamine fructose-6-phosphate transaminase 2 [Source:MGI Symbol;Acc:MGI:1338883]	3.085	4.22E-04
ENSMUSG00000021596	Mctp1	"multiple C2 domains, transmembrane 1 [Source:MGI Symbol;Acc:MGI:1926021]"	3.084	3.05E-03
ENSMUSG00000027797	Dclk1	doublecortin-like kinase 1 [Source:MGI Symbol;Acc:MGI:1330861]	3.082	4.21E-02
ENSMUSG00000112592	Gm19972	"predicted gene, 19972 [Source:MGI Symbol;Acc:MGI:5012157]"	3.079	6.49E-04
ENSMUSG00000030145	Zfp248	zinc finger protein 248 [Source:MGI Symbol;Acc:MGI:1919970]	3.078	3.89E-02
ENSMUSG00000029925	Tbxas1	"thromboxane A synthase 1, platelet [Source:MGI Symbol;Acc:MGI:98497]"	3.076	2.62E-12
ENSMUSG00000035455	Fignl1	fidgetin-like 1 [Source:MGI Symbol;Acc:MGI:1890648]	3.072	9.13E-04
ENSMUSG00000102749	Gm37598	"predicted gene, 37598 [Source:MGI Symbol;Acc:MGI:5610826]"	3.065	5.35E-03

ENSMUSG00000089998	Phtf1os	"putative homeodomain transcription factor 1, opposite strand [Source:MGI Symbol;Acc:MGI:3698050]"	3.063	2.28E-03
ENSMUSG00000074183	Gsta1	"glutathione S-transferase, alpha 1 (Ya) [Source:MGI Symbol;Acc:MGI:1095417]"	3.059	5.11E-08
ENSMUSG00000032231	Anxa2	annexin A2 [Source:MGI Symbol;Acc:MGI:88246]	3.057	2.10E-10
ENSMUSG00000021262	Evl	Ena-vasodilator stimulated phosphoprotein [Source:MGI Symbol;Acc:MGI:1194884]	3.053	6.24E-05
ENSMUSG00000028341	Nr4a3	"nuclear receptor subfamily 4, group A, member 3 [Source:MGI Symbol;Acc:MGI:1352457]"	3.050	1.96E-04
ENSMUSG00000110631	Gm42047	"predicted gene, 42047 [Source:MGI Symbol;Acc:MGI:5624932]"	3.048	8.08E-14
ENSMUSG00000106212	Gm43112	predicted gene 43112 [Source:MGI Symbol;Acc:MGI:5663249]	3.046	3.96E-03
ENSMUSG00000059060	Rad51b	RAD51 paralog B [Source:MGI Symbol;Acc:MGI:1099436]	3.044	5.01E-05
ENSMUSG00000006403	Adamts4	"a disintegrin-like and metallopeptidase (reprolysin type) with thrombospondin type 1 motif, 4 [Source:MGI Symbol;Acc:MGI:1339949]"	3.040	5.81E-03
ENSMUSG00000027834	Serpini1	"serine (or cysteine) peptidase inhibitor, clade I, member 1 [Source:MGI Symbol;Acc:MGI:1194506]"	3.040	1.99E-02
ENSMUSG00000045322	Tlr9	toll-like receptor 9 [Source:MGI Symbol;Acc:MGI:1932389]	3.039	6.36E-04
ENSMUSG00000021280	Exoc3l4	exocyst complex component 3-like 4 [Source:MGI Symbol;Acc:MGI:1921363]	3.037	4.24E-02
ENSMUSG00000024776	Stambpl1	STAM binding protein like 1 [Source:MGI Symbol;Acc:MGI:1923880]	3.037	5.26E-05
ENSMUSG00000030208	Emp1	epithelial membrane protein 1 [Source:MGI Symbol;Acc:MGI:107941]	3.033	2.99E-03
ENSMUSG00000002808	Epdr1	ependymin related protein 1 (zebrafish) [Source:MGI Symbol;Acc:MGI:2145369]	3.032	1.93E-03
ENSMUSG00000048458	Inka2	inka box actin regulator 2 [Source:MGI Symbol;Acc:MGI:1923497]	3.029	4.87E-02
ENSMUSG00000021250	Fos	FBJ osteosarcoma oncogene [Source:MGI Symbol;Acc:MGI:95574]	3.028	7.08E-08
ENSMUSG00000105864	Gm10484	predicted gene 10484 [Source:MGI Symbol;Acc:MGI:3642756]	3.026	1.52E-06
ENSMUSG00000026429	Ube2t	ubiquitin-conjugating enzyme E2T [Source:MGI Symbol;Acc:MGI:1914446]	3.025	3.27E-04
ENSMUSG00000026676	Ccdc3	coiled-coil domain containing 3 [Source:MGI Symbol;Acc:MGI:1921436]	3.023	3.89E-05
ENSMUSG00000099889	Mrgprb11-ps	"MAS-related GPR, member B11, pseudogene [Source:MGI Symbol;Acc:MGI:3033189]"	3.019	1.49E-02
ENSMUSG00000098641	Rnu3b4	U3B small nuclear RNA 4 [Source:MGI Symbol;Acc:MGI:97988]	3.012	1.45E-02
ENSMUSG00000036036	Zfp57	zinc finger protein 57 [Source:MGI Symbol;Acc:MGI:99204]	3.011	2.51E-02
ENSMUSG00000079227	Ccr5	chemokine (C-C motif) receptor 5 [Source:MGI Symbol;Acc:MGI:107182]	3.011	1.24E-25
ENSMUSG00000041801	Phlda3	"pleckstrin homology like domain, family A, member 3 [Source:MGI Symbol;Acc:MGI:1351485]"	3.009	3.02E-02
ENSMUSG00000028931	Kcnab2	"potassium voltage-gated channel, shaker-related subfamily, beta member 2 [Source:MGI Symbol;Acc:MGI:109239]"	3.009	1.25E-04
ENSMUSG00000037887	Dusp8	dual specificity phosphatase 8 [Source:MGI Symbol;Acc:MGI:106626]	3.008	2.60E-05
ENSMUSG00000006218	Fam131c	"family with sequence similarity 131, member C [Source:MGI Symbol;Acc:MGI:2685539]"	3.003	1.73E-02
ENSMUSG00000047420	Fam180a	"family with sequence similarity 180, member A [Source:MGI Symbol;Acc:MGI:3039626]"	3.001	2.95E-04
ENSMUSG00000018008	Cyth4	cytohesin 4 [Source:MGI Symbol;Acc:MGI:2441702]	3.000	1.26E-22
ENSMUSG00000031362	Xlr4c	X-linked lymphocyte-regulated 4C [Source:MGI Symbol;Acc:MGI:3574099]	2.998	1.59E-02
ENSMUSG00000027496	Aurka	aurora kinase A [Source:MGI Symbol;Acc:MGI:894678]	2.998	2.57E-02
ENSMUSG00000028111	Ctsk	cathepsin K [Source:MGI Symbol;Acc:MGI:107823]	2.996	9.33E-04
ENSMUSG00000028859	Csf3r	colony stimulating factor 3 receptor (granulocyte) [Source:MGI Symbol;Acc:MGI:1339755]	2.994	7.48E-06
ENSMUSG00000105062	Gm43113	predicted gene 43113 [Source:MGI Symbol;Acc:MGI:5663250]	2.993	9.90E-03
ENSMUSG00000019942	Cdk1	cyclin-dependent kinase 1 [Source:MGI Symbol;Acc:MGI:88351]	2.988	2.91E-02
ENSMUSG00000102950	Gm37018	"predicted gene, 37018 [Source:MGI Symbol;Acc:MGI:5610246]"	2.987	3.72E-03
ENSMUSG00000028873	Cdca8	cell division cycle associated 8 [Source:MGI Symbol;Acc:MGI:1196274]	2.987	3.54E-02
ENSMUSG00000026043	Col3a1	"collagen, type III, alpha 1 [Source:MGI Symbol;Acc:MGI:88453]"	2.984	1.23E-03
ENSMUSG00000085058	8030453O22Rik	RIKEN cDNA 8030453O22 gene [Source:MGI Symbol;Acc:MGI:1924459]	2.983	3.69E-02
ENSMUSG00000115471	C230086J09Rik	RIKEN cDNA C230086J09 gene [Source:MGI Symbol;Acc:MGI:2443423]	2.983	9.50E-03
ENSMUSG00000040003	Magi2	"membrane associated guanylate kinase, WW and PDZ domain containing 2 [Source:MGI Symbol;Acc:MGI:1354953]"	2.982	2.73E-02
ENSMUSG00000028766	Alpl	"alkaline phosphatase, liver/bone/kidney [Source:MGI Symbol;Acc:MGI:87983]"	2.982	3.21E-13
ENSMUSG00000105980	Gm42597	predicted gene 42597 [Source:MGI Symbol;Acc:MGI:5662734]	2.981	2.57E-02
ENSMUSG00000109268	9530078K11Rik	RIKEN cDNA 9530078K11 gene [Source:MGI Symbol;Acc:MGI:1925814]	2.978	2.74E-02
ENSMUSG00000032740	Ccdc88a	coiled coil domain containing 88A [Source:MGI Symbol;Acc:MGI:1925177]	2.977	3.34E-20
ENSMUSG00000032643	Fhl3	four and a half LIM domains 3 [Source:MGI Symbol;Acc:MGI:1341092]	2.975	4.80E-02
ENSMUSG00000106758	Gm42731	predicted gene 42731 [Source:MGI Symbol;Acc:MGI:5662868]	2.975	1.06E-03
ENSMUSG00000078763	Slfn1	schlafen 1 [Source:MGI Symbol;Acc:MGI:1313259]	2.975	6.76E-04
ENSMUSG00000030365	Clec2i	"C-type lectin domain family 2, member i [Source:MGI Symbol;Acc:MGI:2136650]"	2.974	2.41E-04

ENSMUSG00000110914	Gm48611	"predicted gene, 48611 [Source:MGI Symbol;Acc:MGI:6098198]"	2.974	3.02E-09
ENSMUSG00000038642	Ctss	cathepsin S [Source:MGI Symbol;Acc:MGI:107341]	2.969	1.56E-16
ENSMUSG00000115200	Gm46516	"predicted gene, 46516 [Source:MGI Symbol;Acc:MGI:5826153]"	2.966	9.68E-09
ENSMUSG00000112324	Gm47939	"predicted gene, 47939 [Source:MGI Symbol;Acc:MGI:6097203]"	2.966	3.55E-03
ENSMUSG00000105287	Gm43577	predicted gene 43577 [Source:MGI Symbol;Acc:MGI:5663714]	2.966	1.28E-03
ENSMUSG00000015396	Cd83	CD83 antigen [Source:MGI Symbol;Acc:MGI:1328316]	2.956	4.70E-08
ENSMUSG00000105701	Gm42587	predicted gene 42587 [Source:MGI Symbol;Acc:MGI:5662724]	2.949	7.08E-04
ENSMUSG00000037239	Spred3	sprouty-related EVH1 domain containing 3 [Source:MGI Symbol;Acc:MGI:2142186]	2.946	1.48E-03
ENSMUSG00000065049	Gm24041	"predicted gene, 24041 [Source:MGI Symbol;Acc:MGI:5453818]"	2.946	1.67E-03
ENSMUSG00000056145	AI504432	expressed sequence AI504432 [Source:MGI Symbol;Acc:MGI:2139742]	2.944	2.58E-11
ENSMUSG00000047414	Flrt2	fibronectin leucine rich transmembrane protein 2 [Source:MGI Symbol;Acc:MGI:3603594]	2.942	4.80E-09
ENSMUSG00000073125	Xlr3b	X-linked lymphocyte-regulated 3B [Source:MGI Symbol;Acc:MGI:109505]	2.941	1.08E-02
ENSMUSG00000056738	A730036I17Rik	RIKEN cDNA A730036I17 gene [Source:MGI Symbol;Acc:MGI:3041182]	2.938	3.81E-03
ENSMUSG00000038679	Trps1	transcriptional repressor GATA binding 1 [Source:MGI Symbol;Acc:MGI:1927616]	2.936	4.32E-15
ENSMUSG00000040473	Cfap69	cilia and flagella associated protein 69 [Source:MGI Symbol;Acc:MGI:2443778]	2.936	3.24E-02
ENSMUSG00000085180	AI838599	expressed sequence AI838599 [Source:MGI Symbol;Acc:MGI:3510989]	2.936	1.04E-02
ENSMUSG00000015568	Lpl	lipoprotein lipase [Source:MGI Symbol;Acc:MGI:96820]	2.932	4.45E-37
ENSMUSG00000000290	Itgb2	integrin beta 2 [Source:MGI Symbol;Acc:MGI:96611]	2.930	1.59E-15
ENSMUSG00000020695	Mrc2	"mannose receptor, C type 2 [Source:MGI Symbol;Acc:MGI:107818]"	2.927	6.12E-06
ENSMUSG00000026956	Uap1l1	UDP-N-acteylglucosamine pyrophosphorylase 1-like 1 [Source:MGI Symbol;Acc:MGI:2443318]	2.919	6.16E-12
ENSMUSG00000108521	Gm44639	predicted gene 44639 [Source:MGI Symbol;Acc:MGI:5753215]	2.918	3.04E-09
ENSMUSG00000069920	B3gnt9	"UDP-GlcNAc:betaGal beta-1,3-N-acetylglucosaminyltransferase 9 [Source:MGI Symbol;Acc:MGI:2142841]"	2.915	1.02E-02
ENSMUSG00000029377	Ereg	epiregulin [Source:MGI Symbol;Acc:MGI:107508]	2.914	2.15E-03
ENSMUSG00000079419	Ms4a6c	"membrane-spanning 4-domains, subfamily A, member 6C [Source:MGI Symbol;Acc:MGI:2385644]"	2.912	7.72E-11
ENSMUSG00000027009	Itga4	integrin alpha 4 [Source:MGI Symbol;Acc:MGI:96603]	2.911	6.97E-14
ENSMUSG00000024677	Ms4a6b	"membrane-spanning 4-domains, subfamily A, member 6B [Source:MGI Symbol;Acc:MGI:1917024]"	2.911	9.72E-07
ENSMUSG00000056069	Otulinl	OTU deubiquitinase with linear linkage specificity like [Source:MGI Symbol;Acc:MGI:2687281]	2.910	5.21E-07
ENSMUSG00000037572	Wdhd1	WD repeat and HMG-box DNA binding protein 1 [Source:MGI Symbol;Acc:MGI:2443514]	2.909	8.04E-09
ENSMUSG00000023908	Pkmyt1	"protein kinase, membrane associated tyrosine/threonine 1 [Source:MGI Symbol;Acc:MGI:2137630]"	2.907	1.03E-02
ENSMUSG00000106837	D630030B08Rik	RIKEN cDNA D630030B08 gene [Source:MGI Symbol;Acc:MGI:2443017]	2.906	1.31E-05
ENSMUSG00000026832	Cytip	cytohesin 1 interacting protein [Source:MGI Symbol;Acc:MGI:2183535]	2.905	1.06E-05
ENSMUSG00000027230	Creb3l1	cAMP responsive element binding protein 3-like 1 [Source:MGI Symbol;Acc:MGI:1347062]	2.905	2.72E-05
ENSMUSG00000034457	Eda2r	ectodysplasin A2 receptor [Source:MGI Symbol;Acc:MGI:2442860]	2.901	4.83E-05
ENSMUSG00000100666	1700007F19Rik	RIKEN cDNA 1700007F19 gene [Source:MGI Symbol;Acc:MGI:1915100]	2.900	4.32E-08
ENSMUSG00000104476	Gm38211	"predicted gene, 38211 [Source:MGI Symbol;Acc:MGI:5611439]"	2.900	4.49E-10
ENSMUSG00000029283	Cdc7	cell division cycle 7 (S. cerevisiae) [Source:MGI Symbol;Acc:MGI:1309511]	2.898	1.30E-02
ENSMUSG00000026207	Speg	SPEG complex locus [Source:MGI Symbol;Acc:MGI:109282]	2.898	5.71E-03
ENSMUSG00000028369	Svep1	"sushi, von Willebrand factor type A, EGF and pentraxin domain containing 1 [Source:MGI Symbol;Acc:MGI:1928849]"	2.894	2.29E-03
ENSMUSG00000021696	Elovl7	"ELOVL family member 7, elongation of long chain fatty acids (yeast) [Source:MGI Symbol;Acc:MGI:1921809]"	2.894	4.25E-04
ENSMUSG00000104034	2900092N22Rik	RIKEN cDNA 2900092N22 gene [Source:MGI Symbol;Acc:MGI:1920357]	2.890	6.99E-03
ENSMUSG00000026321	Tnfrsf11a	"tumor necrosis factor receptor superfamily, member 11a, NFKB activator [Source:MGI Symbol;Acc:MGI:1314891]"	2.889	1.83E-09
ENSMUSG00000053318	Slamf8	SLAM family member 8 [Source:MGI Symbol;Acc:MGI:1921998]	2.888	1.09E-04
ENSMUSG00000023349	Clec4n	"C-type lectin domain family 4, member n [Source:MGI Symbol;Acc:MGI:1861231]"	2.885	1.01E-05
ENSMUSG00000020656	Grhl1	grainyhead like transcription factor 1 [Source:MGI Symbol;Acc:MGI:2182540]	2.885	2.62E-04
ENSMUSG00000020437	Myo1g	myosin IG [Source:MGI Symbol;Acc:MGI:1927091]	2.880	3.31E-07
ENSMUSG00000056413	Adap1	ArfGAP with dual PH domains 1 [Source:MGI Symbol;Acc:MGI:2442201]	2.878	1.63E-04
ENSMUSG00000089722	Cd300ld5	CD300 molecule like family member D5 [Source:MGI Symbol;Acc:MGI:3702661]	2.875	9.06E-03
ENSMUSG00000044827	Tlr1	toll-like receptor 1 [Source:MGI Symbol;Acc:MGI:1341295]	2.872	1.17E-11
ENSMUSG00000092528	Nlrp1c-ps	"NLR family, pyrin domain containing 1C, pseudogene [Source:MGI Symbol;Acc:MGI:3582962]"	2.869	3.49E-04
ENSMUSG00000085457	1110046J04Rik	RIKEN cDNA 1110046J04 gene [Source:MGI Symbol;Acc:MGI:1916058]	2.868	3.24E-02

ENSMUSG00000040522	Tlr8	toll-like receptor 8 [Source:MGI Symbol;Acc:MGI:2176887]	2.866	3.82E-09
ENSMUSG00000018774	Cd68	CD68 antigen [Source:MGI Symbol;Acc:MGI:88342]	2.865	3.68E-09
ENSMUSG00000031659	Adcy7	adenylate cyclase 7 [Source:MGI Symbol;Acc:MGI:102891]	2.863	1.10E-18
ENSMUSG00000040212	Emp3	epithelial membrane protein 3 [Source:MGI Symbol;Acc:MGI:1098729]	2.862	1.91E-06
ENSMUSG00000087213	2810408I11Rik	RIKEN cDNA 2810408I11 gene [Source:MGI Symbol;Acc:MGI:1917191]	2.861	3.09E-05
ENSMUSG00000072623	Zfp9	zinc finger protein 9 [Source:MGI Symbol;Acc:MGI:99210]	2.859	3.35E-09
ENSMUSG00000037816	Fbxw17	F-box and WD-40 domain protein 17 [Source:MGI Symbol;Acc:MGI:1923584]	2.858	1.38E-03
ENSMUSG00000070691	Runx3	runt related transcription factor 3 [Source:MGI Symbol;Acc:MGI:102672]	2.856	1.72E-02
ENSMUSG00000032020	Ubash3b	"ubiquitin associated and SH3 domain containing, B [Source:MGI Symbol;Acc:MGI:1920078]"	2.853	1.28E-07
ENSMUSG00000028212	Ccne2	cyclin E2 [Source:MGI Symbol;Acc:MGI:1329034]	2.851	4.75E-03
ENSMUSG00000021822	Plau	"plasminogen activator, urokinase [Source:MGI Symbol;Acc:MGI:97611]"	2.851	1.82E-04
ENSMUSG00000031934	Panx1	pannexin 1 [Source:MGI Symbol;Acc:MGI:1860055]	2.850	6.14E-04
ENSMUSG00000103749	Pcdhgb5	"protocadherin gamma subfamily B, 5 [Source:MGI Symbol;Acc:MGI:1935196]"	2.847	1.84E-04
ENSMUSG00000021508	Cxcl14	chemokine (C-X-C motif) ligand 14 [Source:MGI Symbol;Acc:MGI:1888514]	2.846	9.02E-06
ENSMUSG00000031904	Slc7a6	"solute carrier family 7 (cationic amino acid transporter, y+ system), member 6 [Source:MGI Symbol;Acc:MGI:2142598]"	2.841	1.06E-02
ENSMUSG00000071714	Csf2rb2	"colony stimulating factor 2 receptor, beta 2, low-affinity (granulocyte-macrophage) [Source:MGI Symbol;Acc:MGI:1339760]"	2.840	2.78E-11
ENSMUSG00000027004	Frzb	frizzled-related protein [Source:MGI Symbol;Acc:MGI:892032]	2.839	4.76E-05
ENSMUSG00000074115	Saa1	serum amyloid A 1 [Source:MGI Symbol;Acc:MGI:98221]	2.839	1.15E-09
ENSMUSG00000097493	9930014A18Rik	RIKEN cDNA 9930014A18 gene [Source:MGI Symbol;Acc:MGI:2444091]	2.836	3.42E-02
ENSMUSG00000026039	Sgo2a	shugoshin 2A [Source:MGI Symbol;Acc:MGI:1098767]	2.836	7.41E-03
ENSMUSG00000000386	Mx1	MX dynamin-like GTPase 1 [Source:MGI Symbol;Acc:MGI:97243]	2.835	4.27E-07
ENSMUSG00000103088	Pcdhgb6	"protocadherin gamma subfamily B, 6 [Source:MGI Symbol;Acc:MGI:1935197]"	2.827	1.89E-03
ENSMUSG00000032344	Cgas	cyclic GMP-AMP synthase [Source:MGI Symbol;Acc:MGI:2442261]	2.825	3.95E-04
ENSMUSG00000112820	Gm48298	"predicted gene, 48298 [Source:MGI Symbol;Acc:MGI:6097740]"	2.824	9.56E-03
ENSMUSG00000105971	Gm43805	predicted gene 43805 [Source:MGI Symbol;Acc:MGI:5663942]	2.824	7.59E-03
ENSMUSG00000027995	Tlr2	toll-like receptor 2 [Source:MGI Symbol;Acc:MGI:1346060]	2.822	1.21E-10
ENSMUSG00000031538	Plat	"plasminogen activator, tissue [Source:MGI Symbol;Acc:MGI:97610]"	2.822	3.04E-02
ENSMUSG00000028059	Arhgef2	rho/rac guanine nucleotide exchange factor (GEF) 2 [Source:MGI Symbol;Acc:MGI:103264]	2.813	6.25E-16
ENSMUSG00000028459	Cd72	CD72 antigen [Source:MGI Symbol;Acc:MGI:88345]	2.813	1.79E-05
ENSMUSG00000028337	Coro2a	"coronin, actin binding protein 2A [Source:MGI Symbol;Acc:MGI:1345966]"	2.813	6.82E-03
ENSMUSG00000109358	Gm44716	predicted gene 44716 [Source:MGI Symbol;Acc:MGI:5753292]	2.812	2.81E-02
ENSMUSG00000009092	Derl3	"Der1-like domain family, member 3 [Source:MGI Symbol;Acc:MGI:1917627]"	2.811	1.37E-02
ENSMUSG00000102427	Gm37463	"predicted gene, 37463 [Source:MGI Symbol;Acc:MGI:5610691]"	2.810	3.39E-05
ENSMUSG00000002944	Cd36	CD36 molecule [Source:MGI Symbol;Acc:MGI:107899]	2.809	8.94E-18
ENSMUSG00000048126	Col6a3	"collagen, type VI, alpha 3 [Source:MGI Symbol;Acc:MGI:88461]"	2.809	1.02E-08
ENSMUSG00000045934	Mtmr11	myotubularin related protein 11 [Source:MGI Symbol;Acc:MGI:2652817]	2.806	1.04E-06
ENSMUSG00000027907	S100a11	S100 calcium binding protein A11 [Source:MGI Symbol;Acc:MGI:1338798]	2.806	3.64E-05
ENSMUSG00000024696	Lpxn	leupaxin [Source:MGI Symbol;Acc:MGI:2147677]	2.801	1.57E-05
ENSMUSG00000045763	Basp1	"brain abundant, membrane attached signal protein 1 [Source:MGI Symbol;Acc:MGI:1917600]"	2.801	7.11E-06
ENSMUSG00000030283	St8sia1	"ST8 alpha-N-acetyl-neuraminide alpha-2,8-sialyltransferase 1 [Source:MGI Symbol;Acc:MGI:106011]"	2.796	1.76E-03
ENSMUSG00000040026	Saa3	serum amyloid A 3 [Source:MGI Symbol;Acc:MGI:98223]	2.791	9.38E-05
ENSMUSG00000027339	Rassf2	Ras association (RalGDS/AF-6) domain family member 2 [Source:MGI Symbol;Acc:MGI:2442060]	2.788	1.34E-15
ENSMUSG00000029659	Medag	mesenteric estrogen dependent adipogenesis [Source:MGI Symbol;Acc:MGI:1917967]	2.787	4.40E-02
ENSMUSG00000040990	Sh3kbp1	SH3-domain kinase binding protein 1 [Source:MGI Symbol;Acc:MGI:1889583]	2.783	4.45E-08
ENSMUSG00000072812	Ahnak2	AHNAK nucleoprotein 2 [Source:MGI Symbol;Acc:MGI:2144831]	2.781	2.81E-03
ENSMUSG00000031762	Mt2	metallothionein 2 [Source:MGI Symbol;Acc:MGI:97172]	2.781	2.58E-03
ENSMUSG00000030148	Clec4a2	"C-type lectin domain family 4, member a2 [Source:MGI Symbol;Acc:MGI:1349412]"	2.779	1.29E-05
ENSMUSG00000031367	Ap1s2	"adaptor-related protein complex 1, sigma 2 subunit [Source:MGI Symbol;Acc:MGI:1889383]"	2.778	2.18E-10
ENSMUSG00000072596	Ear2	"eosinophil-associated, ribonuclease A family, member 2 [Source:MGI Symbol;Acc:MGI:108020]"	2.775	3.40E-05
ENSMUSG00000037313	Tacc3	"transforming, acidic coiled-coil containing protein 3 [Source:MGI Symbol;Acc:MGI:1341163]"	2.772	3.66E-02

ENSMUSG00000021624	Cd180	CD180 antigen [Source:MGI Symbol;Acc:MGI:1194924]	2.771	1.79E-13
ENSMUSG00000103427	Gm37534	"predicted gene, 37534 [Source:MGI Symbol;Acc:MGI:5610762]"	2.768	7.24E-06
ENSMUSG00000040710	St8sia4	"ST8 alpha-N-acetyl-neuraminide alpha-2,8-sialyltransferase 4 [Source:MGI Symbol;Acc:MGI:106018]"	2.768	2.35E-24
ENSMUSG00000115975	Gm49526	"predicted gene, 49526 [Source:MGI Symbol;Acc:MGI:6155225]"	2.768	1.04E-07
ENSMUSG00000026548	Slamf9	SLAM family member 9 [Source:MGI Symbol;Acc:MGI:1923692]	2.767	4.06E-03
ENSMUSG00000048922	Cdca2	cell division cycle associated 2 [Source:MGI Symbol;Acc:MGI:1919787]	2.765	4.16E-02
ENSMUSG00000025058	5430427O19Rik	RIKEN cDNA 5430427O19 gene [Source:MGI Symbol;Acc:MGI:1918648]	2.763	5.93E-03
ENSMUSG00000106528	Gm42841	predicted gene 42841 [Source:MGI Symbol;Acc:MGI:5662978]	2.759	1.41E-02
ENSMUSG00000089129	Gm26407	"predicted gene, 26407 [Source:MGI Symbol;Acc:MGI:5456184]"	2.759	2.33E-03
ENSMUSG00000034652	Cd300a	CD300A molecule [Source:MGI Symbol;Acc:MGI:2443411]	2.758	6.95E-07
ENSMUSG00000106339	Gm43489	predicted gene 43489 [Source:MGI Symbol;Acc:MGI:5663626]	2.758	8.09E-07
ENSMUSG00000029204	Rhoh	ras homolog family member H [Source:MGI Symbol;Acc:MGI:1921984]	2.756	2.08E-10
ENSMUSG00000018507	Trpv2	"transient receptor potential cation channel, subfamily V, member 2 [Source:MGI Symbol;Acc:MGI:1341836]"	2.755	3.83E-03
ENSMUSG00000040289	Hey1	hairy/enhancer-of-split related with YRPW motif 1 [Source:MGI Symbol;Acc:MGI:1341800]	2.753	5.39E-03
ENSMUSG00000070390	Nlrp1b	"NLR family, pyrin domain containing 1B [Source:MGI Symbol;Acc:MGI:3582959]"	2.753	3.74E-05
ENSMUSG00000109940	Gm45399	predicted gene 45399 [Source:MGI Symbol;Acc:MGI:5791235]	2.753	9.75E-03
ENSMUSG00000025165	Sectm1a	secreted and transmembrane 1A [Source:MGI Symbol;Acc:MGI:2384805]	2.752	4.40E-03
ENSMUSG00000108460	Gm39041	"predicted gene, 39041 [Source:MGI Symbol;Acc:MGI:5621926]"	2.751	1.93E-12
ENSMUSG00000039109	F13a1	"coagulation factor XIII, A1 subunit [Source:MGI Symbol;Acc:MGI:1921395]"	2.751	1.60E-02
ENSMUSG00000026271	Gpr35	G protein-coupled receptor 35 [Source:MGI Symbol;Acc:MGI:1929509]	2.751	1.78E-06
ENSMUSG00000025877	Hk3	hexokinase 3 [Source:MGI Symbol;Acc:MGI:2670962]	2.751	6.03E-07
ENSMUSG00000020057	Dram1	DNA-damage regulated autophagy modulator 1 [Source:MGI Symbol;Acc:MGI:1918962]	2.745	9.35E-07
ENSMUSG00000032661	Oas3	2'-5' oligoadenylate synthetase 3 [Source:MGI Symbol;Acc:MGI:2180850]	2.740	1.48E-04
ENSMUSG00000104867	Gm43728	predicted gene 43728 [Source:MGI Symbol;Acc:MGI:5663865]	2.738	1.86E-04
ENSMUSG00000042029	Ncapg2	"non-SMC condensin II complex, subunit G2 [Source:MGI Symbol;Acc:MGI:1923294]"	2.738	5.27E-13
ENSMUSG00000063160	Numbl	numb-like [Source:MGI Symbol;Acc:MGI:894702]	2.736	1.97E-04
ENSMUSG00000026458	Ppfia4	"protein tyrosine phosphatase, receptor type, f polypeptide (PTPRF), interacting protein (liprin), alpha 4 [Source:MGI Symbol;Acc:MGI:1915757]"	2.736	2.29E-03
ENSMUSG00000066861	Oas1g	2'-5' oligoadenylate synthetase 1G [Source:MGI Symbol;Acc:MGI:97429]	2.734	9.92E-05
ENSMUSG00000037946	Fgd3	"FYVE, RhoGEF and PH domain containing 3 [Source:MGI Symbol;Acc:MGI:1353657]"	2.732	1.51E-04
ENSMUSG00000030223	Ptpro	"protein tyrosine phosphatase, receptor type, O [Source:MGI Symbol;Acc:MGI:1097152]"	2.732	7.54E-03
ENSMUSG00000032322	Pstpip1	proline-serine-threonine phosphatase-interacting protein 1 [Source:MGI Symbol;Acc:MGI:1321396]	2.731	1.94E-03
ENSMUSG00000026786	Apbb1ip	"amyloid beta (A4) precursor protein-binding, family B, member 1 interacting protein [Source:MGI Symbol;Acc:MGI:1861354]"	2.726	5.97E-17
ENSMUSG00000095580	Rnu1b1	U1b1 small nuclear RNA [Source:MGI Symbol;Acc:MGI:97974]	2.726	7.87E-07
ENSMUSG00000048677	Tpcn2	two pore segment channel 2 [Source:MGI Symbol;Acc:MGI:2385297]	2.726	1.54E-08
ENSMUSG00000031965	Tbx20	T-box 20 [Source:MGI Symbol;Acc:MGI:1888496]	2.726	1.66E-09
ENSMUSG00000017631	Abr	active BCR-related gene [Source:MGI Symbol;Acc:MGI:107771]	2.724	3.79E-09
ENSMUSG00000102376	Gm37975	"predicted gene, 37975 [Source:MGI Symbol;Acc:MGI:5611203]"	2.721	7.08E-03
ENSMUSG00000112816	Gm48798	"predicted gene, 48798 [Source:MGI Symbol;Acc:MGI:6098503]"	2.721	9.43E-03
ENSMUSG00000020143	Dock2	dedicator of cyto-kinesis 2 [Source:MGI Symbol;Acc:MGI:2149010]	2.719	3.22E-20
ENSMUSG00000048865	Arhgap30	Rho GTPase activating protein 30 [Source:MGI Symbol;Acc:MGI:2684948]	2.712	4.52E-17
ENSMUSG00000105128	Gm42870	predicted gene 42870 [Source:MGI Symbol;Acc:MGI:5663007]	2.710	4.13E-02
ENSMUSG00000023034	Nr4a1	"nuclear receptor subfamily 4, group A, member 1 [Source:MGI Symbol;Acc:MGI:1352454]"	2.705	4.77E-03
ENSMUSG00000108897	Gm44861	predicted gene 44861 [Source:MGI Symbol;Acc:MGI:5753437]	2.705	1.24E-04
ENSMUSG00000026042	Col5a2	"collagen, type V, alpha 2 [Source:MGI Symbol;Acc:MGI:88458]"	2.701	1.34E-06
ENSMUSG00000028926	Cdk14	cyclin-dependent kinase 14 [Source:MGI Symbol;Acc:MGI:894318]	2.701	8.44E-06
ENSMUSG00000110866	Gm48362	"predicted gene, 48362 [Source:MGI Symbol;Acc:MGI:6097831]"	2.700	2.49E-02
ENSMUSG00000059326	Csf2ra	"colony stimulating factor 2 receptor, alpha, low-affinity (granulocyte-macrophage) [Source:MGI Symbol;Acc:MGI:1339754]"	2.700	1.03E-04
ENSMUSG00000030302	Atp2b2	"ATPase, Ca++ transporting, plasma membrane 2 [Source:MGI Symbol;Acc:MGI:105368]"	2.698	1.60E-07
ENSMUSG00000045404	Kcnk13	"potassium channel, subfamily K, member 13 [Source:MGI Symbol;Acc:MGI:2384976]"	2.698	6.11E-03
ENSMUSG00000084087	Gm13650	predicted gene 13650 [Source:MGI Symbol;Acc:MGI:3649525]	2.697	3.12E-02

ENSMUSG00000005267	Zfp287	zinc finger protein 287 [Source:MGI Symbol;Acc:MGI:2176561]	2.694	4.32E-03
ENSMUSG00000116639	Gm49730	"predicted gene, 49730 [Source:MGI Symbol;Acc:MGI:6215212]"	2.693	4.55E-05
ENSMUSG00000074802	Gas2l3	growth arrest-specific 2 like 3 [Source:MGI Symbol;Acc:MGI:1918780]	2.691	5.15E-14
ENSMUSG00000097636	Mirt1	myocardial infarction associated transcript 1 [Source:MGI Symbol;Acc:MGI:1922001]	2.690	2.00E-02
ENSMUSG00000038260	Trpm4	"transient receptor potential cation channel, subfamily M, member 4 [Source:MGI Symbol;Acc:MGI:1915917]"	2.689	1.32E-04
ENSMUSG00000106121	Gm42679	predicted gene 42679 [Source:MGI Symbol;Acc:MGI:5662816]	2.685	1.30E-08
ENSMUSG00000049313	Sorl1	"sortilin-related receptor, LDLR class A repeats-containing [Source:MGI Symbol;Acc:MGI:1202296]"	2.684	2.96E-10
ENSMUSG00000103192	Gm37645	"predicted gene, 37645 [Source:MGI Symbol;Acc:MGI:5610873]"	2.683	4.76E-04
ENSMUSG00000024940	Ltbp3	latent transforming growth factor beta binding protein 3 [Source:MGI Symbol;Acc:MGI:1101355]	2.682	2.75E-05
ENSMUSG00000040152	Thbs1	thrombospondin 1 [Source:MGI Symbol;Acc:MGI:98737]	2.680	1.89E-03
ENSMUSG00000115898	Gm49524	"predicted gene, 49524 [Source:MGI Symbol;Acc:MGI:6155221]"	2.679	3.81E-04
ENSMUSG00000035042	Ccl5	chemokine (C-C motif) ligand 5 [Source:MGI Symbol;Acc:MGI:98262]	2.678	6.44E-03
ENSMUSG00000030165	Klrd1	"killer cell lectin-like receptor, subfamily D, member 1 [Source:MGI Symbol;Acc:MGI:1196275]"	2.676	2.22E-02
ENSMUSG00000049744	Arhgap15	Rho GTPase activating protein 15 [Source:MGI Symbol;Acc:MGI:1923367]	2.674	1.23E-09
ENSMUSG00000090164	BC035044	cDNA sequence BC035044 [Source:MGI Symbol;Acc:MGI:2448540]	2.672	1.23E-02
ENSMUSG00000073492	Gm10521	predicted gene 10521 [Source:MGI Symbol;Acc:MGI:3642358]	2.672	1.35E-09
ENSMUSG00000042349	Ikbke	inhibitor of kappaB kinase epsilon [Source:MGI Symbol;Acc:MGI:1929612]	2.671	1.61E-13
ENSMUSG00000029442	Wdr66	WD repeat domain 66 [Source:MGI Symbol;Acc:MGI:1918495]	2.671	4.80E-03
ENSMUSG00000027848	Olfml3	olfactomedin-like 3 [Source:MGI Symbol;Acc:MGI:1914877]	2.669	1.46E-04
ENSMUSG00000107215	Gm43197	predicted gene 43197 [Source:MGI Symbol;Acc:MGI:5663334]	2.667	1.06E-06
ENSMUSG00000043740	B430306N03Rik	RIKEN cDNA B430306N03 gene [Source:MGI Symbol;Acc:MGI:2443478]	2.666	6.21E-08
ENSMUSG00000097760	6030442K20Rik	RIKEN cDNA 6030442K20 gene [Source:MGI Symbol;Acc:MGI:1925126]	2.665	4.34E-04
ENSMUSG00000054640	Slc8a1	"solute carrier family 8 (sodium/calcium exchanger), member 1 [Source:MGI Symbol;Acc:MGI:107956]"	2.665	2.62E-10
ENSMUSG00000022074	Tnfrsf10b	"tumor necrosis factor receptor superfamily, member 10b [Source:MGI Symbol;Acc:MGI:1341090]"	2.665	1.43E-02
ENSMUSG00000015340	Cybb	"cytochrome b-245, beta polypeptide [Source:MGI Symbol;Acc:MGI:88574]"	2.664	2.92E-23
ENSMUSG00000006360	Crip1	cysteine-rich protein 1 (intestinal) [Source:MGI Symbol;Acc:MGI:88501]	2.663	6.80E-04
ENSMUSG00000045502	Hcar2	hydroxycarboxylic acid receptor 2 [Source:MGI Symbol;Acc:MGI:1933383]	2.663	4.80E-03
ENSMUSG00000108752	Gm45191	predicted gene 45191 [Source:MGI Symbol;Acc:MGI:5753767]	2.662	2.51E-09
ENSMUSG00000032089	Il10ra	"interleukin 10 receptor, alpha [Source:MGI Symbol;Acc:MGI:96538]"	2.661	1.25E-11
ENSMUSG00000107041	Gm42735	predicted gene 42735 [Source:MGI Symbol;Acc:MGI:5662872]	2.652	1.53E-02
ENSMUSG00000102253	Gm38259	"predicted gene, 38259 [Source:MGI Symbol;Acc:MGI:5611487]"	2.649	4.29E-02
ENSMUSG00000093834	Rnu1b2	U1b2 small nuclear RNA [Source:MGI Symbol;Acc:MGI:104624]	2.648	1.62E-06
ENSMUSG00000024660	Incenp	inner centromere protein [Source:MGI Symbol;Acc:MGI:1313288]	2.648	6.80E-04
ENSMUSG00000031389	Arhgap4	Rho GTPase activating protein 4 [Source:MGI Symbol;Acc:MGI:2159577]	2.647	1.08E-04
ENSMUSG00000020473	Aebp1	AE binding protein 1 [Source:MGI Symbol;Acc:MGI:1197012]	2.647	4.75E-02
ENSMUSG00000022488	Nckap1l	NCK associated protein 1 like [Source:MGI Symbol;Acc:MGI:1926063]	2.645	4.46E-17
ENSMUSG00000052736	Klrc2	"killer cell lectin-like receptor subfamily C, member 2 [Source:MGI Symbol;Acc:MGI:1336162]"	2.644	1.43E-03
ENSMUSG00000029651	Mtus2	microtubule associated tumor suppressor candidate 2 [Source:MGI Symbol;Acc:MGI:1915388]	2.642	1.87E-02
ENSMUSG00000053007	Creb5	cAMP responsive element binding protein 5 [Source:MGI Symbol;Acc:MGI:2443973]	2.641	4.91E-06
ENSMUSG00000027843	Ptpn22	"protein tyrosine phosphatase, non-receptor type 22 (lymphoid) [Source:MGI Symbol;Acc:MGI:107170]"	2.640	9.18E-06
ENSMUSG00000031740	Mmp2	matrix metallopeptidase 2 [Source:MGI Symbol;Acc:MGI:97009]	2.638	2.75E-04
ENSMUSG00000113680	Gm47405	"predicted gene, 47405 [Source:MGI Symbol;Acc:MGI:6096336]"	2.637	6.67E-03
ENSMUSG00000021943	Gdf10	growth differentiation factor 10 [Source:MGI Symbol;Acc:MGI:95684]	2.637	1.29E-03
ENSMUSG00000038147	Cd84	CD84 antigen [Source:MGI Symbol;Acc:MGI:1336885]	2.636	7.03E-26
ENSMUSG00000104140	Gm37140	"predicted gene, 37140 [Source:MGI Symbol;Acc:MGI:5610368]"	2.635	2.71E-04
ENSMUSG00000056290	Ms4a4b	"membrane-spanning 4-domains, subfamily A, member 4B [Source:MGI Symbol;Acc:MGI:1913083]"	2.635	8.71E-08
ENSMUSG00000027469	Tpx2	"TPX2, microtubule-associated [Source:MGI Symbol;Acc:MGI:1919369]"	2.634	6.95E-03
ENSMUSG00000044811	Cd300c2	CD300C molecule 2 [Source:MGI Symbol;Acc:MGI:2153249]	2.634	4.19E-05
ENSMUSG00000104888	1500005C15Rik	RIKEN cDNA 1500005C15 gene [Source:MGI Symbol;Acc:MGI:1919269]	2.634	9.49E-03
ENSMUSG00000041431	Ccnb1	cyclin B1 [Source:MGI Symbol;Acc:MGI:88302]	2.633	3.98E-03

ENSMUSG00000106334	Gm43549	predicted gene 43549 [Source:MGI Symbol;Acc:MGI:5663686]	2.631	2.24E-04
ENSMUSG00000026196	Bard1	BRCA1 associated RING domain 1 [Source:MGI Symbol;Acc:MGI:1328361]	2.631	1.32E-03
ENSMUSG00000024965	Fermt3	fermitin family member 3 [Source:MGI Symbol;Acc:MGI:2147790]	2.630	3.66E-07
ENSMUSG00000049303	Syt12	synaptotagmin XII [Source:MGI Symbol;Acc:MGI:2159601]	2.630	1.54E-03
ENSMUSG00000006641	Slc5a6	"solute carrier family 5 (sodium-dependent vitamin transporter), member 6 [Source:MGI Symbol;Acc:MGI:2660847]"	2.629	9.42E-08
ENSMUSG00000028037	Ifi44	interferon-induced protein 44 [Source:MGI Symbol;Acc:MGI:2443016]	2.629	3.42E-10
ENSMUSG00000072244	Trim6	tripartite motif-containing 6 [Source:MGI Symbol;Acc:MGI:2137352]	2.627	3.19E-03
ENSMUSG00000015950	Ncf1	neutrophil cytosolic factor 1 [Source:MGI Symbol;Acc:MGI:97283]	2.626	1.84E-10
ENSMUSG00000056498	Tmem154	transmembrane protein 154 [Source:MGI Symbol;Acc:MGI:2444725]	2.624	7.97E-03
ENSMUSG00000104936	2610011E03Rik	RIKEN cDNA 2610011E03 gene [Source:MGI Symbol;Acc:MGI:1919710]	2.623	1.55E-02
ENSMUSG00000024521	Pmaip1	phorbol-12-myristate-13-acetate-induced protein 1 [Source:MGI Symbol;Acc:MGI:1930146]	2.621	4.57E-05
ENSMUSG00000019874	Fabp7	"fatty acid binding protein 7, brain [Source:MGI Symbol;Acc:MGI:101916]"	2.621	1.20E-06
ENSMUSG00000028874	Fgr	"FGR proto-oncogene, Src family tyrosine kinase [Source:MGI Symbol;Acc:MGI:95527]"	2.621	5.77E-05
ENSMUSG00000003541	Ier3	immediate early response 3 [Source:MGI Symbol;Acc:MGI:104814]	2.620	1.15E-02
ENSMUSG00000103123	Gm37390	"predicted gene, 37390 [Source:MGI Symbol;Acc:MGI:5610618]"	2.614	1.66E-04
ENSMUSG00000037902	Sirpa	signal-regulatory protein alpha [Source:MGI Symbol;Acc:MGI:108563]	2.607	1.33E-14
ENSMUSG00000005087	Cd44	CD44 antigen [Source:MGI Symbol;Acc:MGI:88338]	2.607	7.17E-10
ENSMUSG00000021886	Gpr65	G-protein coupled receptor 65 [Source:MGI Symbol;Acc:MGI:108031]	2.606	3.13E-08
ENSMUSG00000054115	Skp2	S-phase kinase-associated protein 2 (p45) [Source:MGI Symbol;Acc:MGI:1351663]	2.604	2.59E-05
ENSMUSG00000113966	Gm47980	"predicted gene, 47980 [Source:MGI Symbol;Acc:MGI:6097267]"	2.603	3.86E-02
ENSMUSG00000107017	Gm43196	predicted gene 43196 [Source:MGI Symbol;Acc:MGI:5663333]	2.602	1.01E-03
ENSMUSG00000021087	Rtn1	reticulon 1 [Source:MGI Symbol;Acc:MGI:1933947]	2.601	3.50E-04
ENSMUSG00000002831	Plin4	perilipin 4 [Source:MGI Symbol;Acc:MGI:1929709]	2.600	5.23E-05
ENSMUSG00000026600	Soat1	sterol O-acyltransferase 1 [Source:MGI Symbol;Acc:MGI:104665]	2.597	2.76E-13
ENSMUSG00000041064	Pif1	PIF1 5'-to-3' DNA helicase [Source:MGI Symbol;Acc:MGI:2143057]	2.595	4.90E-02
ENSMUSG00000048779	P2ry6	"pyrimidinergic receptor P2Y, G-protein coupled, 6 [Source:MGI Symbol;Acc:MGI:2673874]"	2.595	1.39E-04
ENSMUSG00000035372	1810055G02Rik	RIKEN cDNA 1810055G02 gene [Source:MGI Symbol;Acc:MGI:1919306]	2.594	2.01E-09
ENSMUSG00000030616	Sytl2	synaptotagmin-like 2 [Source:MGI Symbol;Acc:MGI:1933366]	2.593	1.56E-03
ENSMUSG00000060317	Acnat2	acyl-coenzyme A amino acid N-acyltransferase 2 [Source:MGI Symbol;Acc:MGI:2444345]	2.592	8.02E-10
ENSMUSG00000025287	Acot9	acyl-CoA thioesterase 9 [Source:MGI Symbol;Acc:MGI:1928939]	2.592	2.08E-04
ENSMUSG00000020099	Unc5b	unc-5 netrin receptor B [Source:MGI Symbol;Acc:MGI:894703]	2.592	6.51E-06
ENSMUSG00000041498	Kif14	kinesin family member 14 [Source:MGI Symbol;Acc:MGI:1098226]	2.589	2.37E-02
ENSMUSG00000034765	Dusp5	dual specificity phosphatase 5 [Source:MGI Symbol;Acc:MGI:2685183]	2.589	2.05E-04
ENSMUSG00000019558	Slc6a8	"solute carrier family 6 (neurotransmitter transporter, creatine), member 8 [Source:MGI Symbol;Acc:MGI:2147834]"	2.589	1.31E-09
ENSMUSG00000104118	Gm37298	"predicted gene, 37298 [Source:MGI Symbol;Acc:MGI:5610526]"	2.588	3.42E-05
ENSMUSG00000079685	Ulbp1	UL16 binding protein 1 [Source:MGI Symbol;Acc:MGI:1925027]	2.587	3.97E-05
ENSMUSG00000038059	Smim3	small integral membrane protein 3 [Source:MGI Symbol;Acc:MGI:1917088]	2.585	2.42E-04
ENSMUSG00000041695	Kcnj2	"potassium inwardly-rectifying channel, subfamily J, member 2 [Source:MGI Symbol;Acc:MGI:104744]"	2.584	6.93E-09
ENSMUSG00000027635	Dsn1	"DSN1 homolog, MIS12 kinetochore complex component [Source:MGI Symbol;Acc:MGI:1914184]"	2.581	9.20E-04
ENSMUSG00000116083	Gm5469	predicted gene 5469 [Source:MGI Symbol;Acc:MGI:3645122]	2.581	2.42E-05
ENSMUSG00000043079	Synpo	synaptopodin [Source:MGI Symbol;Acc:MGI:1099446]	2.579	1.24E-07
ENSMUSG00000097910	5033428I22Rik	RIKEN cDNA 5033428I22 gene [Source:MGI Symbol;Acc:MGI:1923246]	2.577	5.87E-05
ENSMUSG00000079547	H2-DMb1	"histocompatibility 2, class II, locus Mb1 [Source:MGI Symbol;Acc:MGI:95922]"	2.574	1.77E-04
ENSMUSG00000104735	C130075A20Rik	RIKEN cDNA C130075A20 gene [Source:MGI Symbol;Acc:MGI:2443940]	2.571	3.48E-10
ENSMUSG00000019975	Ikbip	IKBKB interacting protein [Source:MGI Symbol;Acc:MGI:1914704]	2.569	3.06E-05
ENSMUSG00000074813	Morrbid	myeloid RNA regulator of BCL2L11 induced cell death [Source:MGI Symbol;Acc:MGI:3652191]	2.564	8.61E-05
ENSMUSG00000037419	Endod1	endonuclease domain containing 1 [Source:MGI Symbol;Acc:MGI:1919196]	2.562	5.94E-06
ENSMUSG00000044583	Tlr7	toll-like receptor 7 [Source:MGI Symbol;Acc:MGI:2176882]	2.561	5.15E-08
ENSMUSG00000110238	Gm19269	"predicted gene, 19269 [Source:MGI Symbol;Acc:MGI:5011454]"	2.561	1.80E-02
ENSMUSG00000041313	Slc7a1	"solute carrier family 7 (cationic amino acid transporter, y+ system), member 1 [Source:MGI Symbol;Acc:MGI:88117]"	2.560	1.62E-04

ENSMUSG00000031264	Btk	Bruton agammaglobulinemia tyrosine kinase [Source:MGI Symbol;Acc:MGI:88216]	2.557	2.97E-04
ENSMUSG00000000861	Bcl11a	B cell CLL/lymphoma 11A (zinc finger protein) [Source:MGI Symbol;Acc:MGI:106190]	2.556	5.93E-09
ENSMUSG00000000732	Icosl	icos ligand [Source:MGI Symbol;Acc:MGI:1354701]	2.555	1.76E-02
ENSMUSG00000027452	Acss1	acyl-CoA synthetase short-chain family member 1 [Source:MGI Symbol;Acc:MGI:1915988]	2.552	1.28E-02
ENSMUSG00000001473	Tubb6	"tubulin, beta 6 class V [Source:MGI Symbol;Acc:MGI:1915201]"	2.550	2.13E-07
ENSMUSG00000026536	Ifi211	interferon activated gene 211 [Source:MGI Symbol;Acc:MGI:3041120]	2.550	1.65E-05
ENSMUSG00000110289	4930412F12Rik	RIKEN cDNA 4930412F12 gene [Source:MGI Symbol;Acc:MGI:1922099]	2.549	2.30E-02
ENSMUSG00000030536	Iqgap1	IQ motif containing GTPase activating protein 1 [Source:MGI Symbol;Acc:MGI:1352757]	2.548	9.22E-16
ENSMUSG00000021725	Parp8	"poly (ADP-ribose) polymerase family, member 8 [Source:MGI Symbol;Acc:MGI:1098713]"	2.548	4.15E-09
ENSMUSG00000109863	Gm45643	predicted gene 45643 [Source:MGI Symbol;Acc:MGI:5791479]	2.547	1.57E-03
ENSMUSG00000028957	Per3	period circadian clock 3 [Source:MGI Symbol;Acc:MGI:1277134]	2.546	9.57E-14
ENSMUSG00000106734	Gm20559	"predicted gene, 20559 [Source:MGI Symbol;Acc:MGI:5295666]"	2.544	1.20E-16
ENSMUSG00000055541	Lair1	leukocyte-associated Ig-like receptor 1 [Source:MGI Symbol;Acc:MGI:105492]	2.544	2.06E-19
ENSMUSG00000032221	Mns1	meiosis-specific nuclear structural protein 1 [Source:MGI Symbol;Acc:MGI:107933]	2.544	1.91E-02
ENSMUSG00000017176	Nt5c3b	"5'-nucleotidase, cytosolic IIIB [Source:MGI Symbol;Acc:MGI:1915356]"	2.544	3.71E-03
ENSMUSG00000113183	Gm47664	"predicted gene, 47664 [Source:MGI Symbol;Acc:MGI:6096754]"	2.541	6.58E-05
ENSMUSG00000103348	Gm37053	"predicted gene, 37053 [Source:MGI Symbol;Acc:MGI:5610281]"	2.541	4.68E-02
ENSMUSG00000022489	Pde1b	"phosphodiesterase 1B, Ca2+-calmodulin dependent [Source:MGI Symbol;Acc:MGI:97523]"	2.540	8.10E-03
ENSMUSG00000021457	Syk	spleen tyrosine kinase [Source:MGI Symbol;Acc:MGI:99515]	2.540	8.03E-17
ENSMUSG00000040747	Cd53	CD53 antigen [Source:MGI Symbol;Acc:MGI:88341]	2.540	3.41E-07
ENSMUSG00000033854	Kcnk10	"potassium channel, subfamily K, member 10 [Source:MGI Symbol;Acc:MGI:1919508]"	2.539	4.93E-05
ENSMUSG00000074579	Lekr1	"leucine, glutamate and lysine rich 1 [Source:MGI Symbol;Acc:MGI:3645902]"	2.539	2.21E-03
ENSMUSG00000064065	Ipcef1	interaction protein for cytohesin exchange factors 1 [Source:MGI Symbol;Acc:MGI:2444159]	2.537	2.22E-03
ENSMUSG00000022822	Abcc5	"ATP-binding cassette, sub-family C (CFTR/MRP), member 5 [Source:MGI Symbol;Acc:MGI:1351644]"	2.537	2.94E-24
ENSMUSG00000046245	Pilra	paired immunoglobin-like type 2 receptor alpha [Source:MGI Symbol;Acc:MGI:2450529]	2.537	1.10E-07
ENSMUSG00000022372	Sla	src-like adaptor [Source:MGI Symbol;Acc:MGI:104295]	2.534	3.70E-13
ENSMUSG00000040183	Ankrd6	ankyrin repeat domain 6 [Source:MGI Symbol;Acc:MGI:2154278]	2.534	3.39E-02
ENSMUSG00000003348	Mob3a	MOB kinase activator 3A [Source:MGI Symbol;Acc:MGI:3050117]	2.531	2.02E-05
ENSMUSG00000022021	Diaph3	diaphanous related formin 3 [Source:MGI Symbol;Acc:MGI:1927222]	2.531	3.82E-02
ENSMUSG00000110605	Gm32856	"predicted gene, 32856 [Source:MGI Symbol;Acc:MGI:5592015]"	2.530	3.68E-03
ENSMUSG00000102428	Pcdhga12	"protocadherin gamma subfamily A, 12 [Source:MGI Symbol;Acc:MGI:1935229]"	2.529	5.04E-03
ENSMUSG00000105990	Gm43307	predicted gene 43307 [Source:MGI Symbol;Acc:MGI:5663444]	2.529	1.98E-03
ENSMUSG00000103828	2610001A08Rik	RIKEN cDNA 2610001A08 gene [Source:MGI Symbol;Acc:MGI:1917358]	2.529	1.03E-02
ENSMUSG00000023886	Smoc2	SPARC related modular calcium binding 2 [Source:MGI Symbol;Acc:MGI:1929881]	2.527	4.70E-04
ENSMUSG00000013921	Clip3	CAP-GLY domain containing linker protein 3 [Source:MGI Symbol;Acc:MGI:1923936]	2.527	2.51E-02
ENSMUSG00000034116	Vav1	vav 1 oncogene [Source:MGI Symbol;Acc:MGI:98923]	2.525	6.21E-05
ENSMUSG00000024675	Ms4a4c	"membrane-spanning 4-domains, subfamily A, member 4C [Source:MGI Symbol;Acc:MGI:1927656]"	2.524	3.83E-04
ENSMUSG00000026274	Pask	PAS domain containing serine/threonine kinase [Source:MGI Symbol;Acc:MGI:2155936]	2.524	2.51E-02
ENSMUSG00000058470	Gm8369	predicted gene 8369 [Source:MGI Symbol;Acc:MGI:3645380]	2.521	1.71E-02
ENSMUSG00000059049	Frem1	Fras1 related extracellular matrix protein 1 [Source:MGI Symbol;Acc:MGI:2670972]	2.517	7.15E-04
ENSMUSG00000027962	Vcam1	vascular cell adhesion molecule 1 [Source:MGI Symbol;Acc:MGI:98926]	2.516	9.58E-13
ENSMUSG00000105660	Gm42975	predicted gene 42975 [Source:MGI Symbol;Acc:MGI:5663112]	2.515	7.90E-05
ENSMUSG00000024299	Adamts10	"a disintegrin-like and metallopeptidase (reprolysin type) with thrombospondin type 1 motif, 10 [Source:MGI Symbol;Acc:MGI:2449112]"	2.514	8.64E-12
ENSMUSG00000078247	Airn	antisense Igf2r RNA [Source:MGI Symbol;Acc:MGI:1353471]	2.514	7.24E-04
ENSMUSG00000053040	Aph1c	"aph1 homolog C, gamma secretase subunit [Source:MGI Symbol;Acc:MGI:1915568]"	2.513	1.94E-04
ENSMUSG00000000628	Hk2	hexokinase 2 [Source:MGI Symbol;Acc:MGI:1315197]	2.512	1.16E-05
ENSMUSG00000104398	Gm37964	"predicted gene, 37964 [Source:MGI Symbol;Acc:MGI:5611192]"	2.512	7.04E-03
ENSMUSG00000100510	Hand2os1	"Hand2, opposite strand 1 [Source:MGI Symbol;Acc:MGI:5578769]"	2.510	2.04E-17
ENSMUSG00000036894	Rap2b	"RAP2B, member of RAS oncogene family [Source:MGI Symbol;Acc:MGI:1921262]"	2.510	3.95E-09
ENSMUSG00000104591	Gm43145	predicted gene 43145 [Source:MGI Symbol;Acc:MGI:5663282]	2.510	1.09E-02

ENSMUSG00000025154	Arhgap19	Rho GTPase activating protein 19 [Source:MGI Symbol;Acc:MGI:1918335]	2.509	1.80E-09
ENSMUSG00000103646	Gm37706	"predicted gene, 37706 [Source:MGI Symbol;Acc:MGI:5610934]"	2.508	4.74E-08
ENSMUSG00000029673	Auts2	autism susceptibility candidate 2 [Source:MGI Symbol;Acc:MGI:1919847]	2.503	4.12E-04
ENSMUSG00000026748	Plxdc2	plexin domain containing 2 [Source:MGI Symbol;Acc:MGI:1914698]	2.501	7.12E-03
ENSMUSG00000037860	Aim2	absent in melanoma 2 [Source:MGI Symbol;Acc:MGI:2686159]	2.499	2.28E-03
ENSMUSG00000021728	Emb	embigin [Source:MGI Symbol;Acc:MGI:95321]	2.498	5.75E-05
ENSMUSG00000018927	Ccl6	chemokine (C-C motif) ligand 6 [Source:MGI Symbol;Acc:MGI:98263]	2.498	5.03E-10
ENSMUSG00000102850	Gm37082	"predicted gene, 37082 [Source:MGI Symbol;Acc:MGI:5610310]"	2.496	7.67E-06
ENSMUSG00000054203	Ifi205	interferon activated gene 205 [Source:MGI Symbol;Acc:MGI:101847]	2.495	5.91E-05
ENSMUSG00000022797	Tfrc	transferrin receptor [Source:MGI Symbol;Acc:MGI:98822]	2.493	1.39E-08
ENSMUSG00000023885	Thbs2	thrombospondin 2 [Source:MGI Symbol;Acc:MGI:98738]	2.491	1.61E-03
ENSMUSG00000044694	2010007H06Rik	RIKEN cDNA 2010007H06 gene [Source:MGI Symbol;Acc:MGI:1917099]	2.491	1.30E-02
ENSMUSG00000047798	Cd300lf	CD300 molecule like family member F [Source:MGI Symbol;Acc:MGI:2442359]	2.490	7.90E-04
ENSMUSG00000038807	Rap1gap2	RAP1 GTPase activating protein 2 [Source:MGI Symbol;Acc:MGI:3028623]	2.488	2.42E-03
ENSMUSG00000022439	Parvg	"parvin, gamma [Source:MGI Symbol;Acc:MGI:2158329]"	2.487	3.40E-02
ENSMUSG00000033453	Adamts15	"a disintegrin-like and metallopeptidase (reprolysin type) with thrombospondin type 1 motif, 15 [Source:MGI Symbol;Acc:MGI:2449569]"	2.487	2.77E-04
ENSMUSG00000108614	2610306O10Rik	RIKEN cDNA 2610306O10 gene [Source:MGI Symbol;Acc:MGI:1917710]	2.487	8.77E-03
ENSMUSG00000026728	Vim	vimentin [Source:MGI Symbol;Acc:MGI:98932]	2.486	2.91E-07
ENSMUSG00000110148	5830408C22Rik	RIKEN cDNA 5830408C22 gene [Source:MGI Symbol;Acc:MGI:1921994]	2.484	7.92E-03
ENSMUSG00000105217	Gm42873	predicted gene 42873 [Source:MGI Symbol;Acc:MGI:5663010]	2.484	4.70E-04
ENSMUSG00000020627	Klhl29	kelch-like 29 [Source:MGI Symbol;Acc:MGI:2683857]	2.482	3.62E-02
ENSMUSG00000027715	Ccna2	cyclin A2 [Source:MGI Symbol;Acc:MGI:108069]	2.481	2.32E-03
ENSMUSG00000036545	Adamts2	"a disintegrin-like and metallopeptidase (reprolysin type) with thrombospondin type 1 motif, 2 [Source:MGI Symbol;Acc:MGI:1347356]"	2.477	2.02E-04
ENSMUSG00000043263	Ifi209	interferon activated gene 209 [Source:MGI Symbol;Acc:MGI:2138243]	2.474	1.59E-07
ENSMUSG00000034675	Dbn1	drebrin 1 [Source:MGI Symbol;Acc:MGI:1931838]	2.470	4.31E-02
ENSMUSG00000020893	Per1	period circadian clock 1 [Source:MGI Symbol;Acc:MGI:1098283]	2.466	1.42E-04
ENSMUSG00000028028	Alpk1	alpha-kinase 1 [Source:MGI Symbol;Acc:MGI:1918731]	2.465	7.38E-05
ENSMUSG00000022504	Ciita	class II transactivator [Source:MGI Symbol;Acc:MGI:108445]	2.465	9.28E-06
ENSMUSG00000025856	Pdgfa	"platelet derived growth factor, alpha [Source:MGI Symbol;Acc:MGI:97527]"	2.464	3.60E-05
ENSMUSG00000057762	Gm6169	predicted gene 6169 [Source:MGI Symbol;Acc:MGI:3646298]	2.464	1.12E-02
ENSMUSG00000097336	Fendrr	Foxf1 adjacent non-coding developmental regulatory RNA [Source:MGI Symbol;Acc:MGI:1916040]	2.457	9.41E-03
ENSMUSG00000104154	Gm38104	"predicted gene, 38104 [Source:MGI Symbol;Acc:MGI:5611332]"	2.452	1.38E-04
ENSMUSG00000030579	Tyrobp	TYRO protein tyrosine kinase binding protein [Source:MGI Symbol;Acc:MGI:1277211]	2.449	8.60E-10
ENSMUSG00000031004	Mki67	antigen identified by monoclonal antibody Ki 67 [Source:MGI Symbol;Acc:MGI:106035]	2.447	2.93E-08
ENSMUSG00000031508	Ankrd10	ankyrin repeat domain 10 [Source:MGI Symbol;Acc:MGI:1921840]	2.447	2.47E-09
ENSMUSG00000022951	Rcan1	regulator of calcineurin 1 [Source:MGI Symbol;Acc:MGI:1890564]	2.446	1.19E-09
ENSMUSG00000064267	Hvcn1	hydrogen voltage-gated channel 1 [Source:MGI Symbol;Acc:MGI:1921346]	2.443	1.18E-04
ENSMUSG00000027555	Car13	carbonic anhydrase 13 [Source:MGI Symbol;Acc:MGI:1931322]	2.443	4.38E-02
ENSMUSG00000050921	P2ry10	"purinergic receptor P2Y, G-protein coupled 10 [Source:MGI Symbol;Acc:MGI:1926076]"	2.443	1.54E-02
ENSMUSG00000004110	Cacna1e	"calcium channel, voltage-dependent, R type, alpha 1E subunit [Source:MGI Symbol;Acc:MGI:106217]"	2.442	3.58E-03
ENSMUSG00000030759	Far1	fatty acyl CoA reductase 1 [Source:MGI Symbol;Acc:MGI:1914670]	2.441	3.38E-07
ENSMUSG00000010461	Eya4	EYA transcriptional coactivator and phosphatase 4 [Source:MGI Symbol;Acc:MGI:1337104]	2.440	6.62E-03
ENSMUSG00000029552	Tes	testis derived transcript [Source:MGI Symbol;Acc:MGI:105081]	2.439	8.19E-06
ENSMUSG00000020389	Cdkl3	cyclin-dependent kinase-like 3 [Source:MGI Symbol;Acc:MGI:2388268]	2.438	1.79E-02
ENSMUSG00000038943	Prc1	protein regulator of cytokinesis 1 [Source:MGI Symbol;Acc:MGI:1858961]	2.438	5.76E-03
ENSMUSG00000085400	Pard3bos3	"par-3 family cell polarity regulator beta, opposite strand 3 [Source:MGI Symbol;Acc:MGI:3651159]"	2.437	1.84E-02
ENSMUSG00000094595	Fsbp	fibrinogen silencer binding protein [Source:MGI Symbol;Acc:MGI:5301008]	2.436	2.28E-03
ENSMUSG00000107583	Gm44104	"predicted gene, 44104 [Source:MGI Symbol;Acc:MGI:5690496]"	2.435	9.17E-03
ENSMUSG00000022360	Atad2	"ATPase family, AAA domain containing 2 [Source:MGI Symbol;Acc:MGI:1917722]"	2.432	4.26E-10
ENSMUSG00000002870	Mcm2	minichromosome maintenance complex component 2 [Source:MGI Symbol;Acc:MGI:105380]	2.430	7.48E-07

ENSMUSG00000070720	Tmem200b	transmembrane protein 200B [Source:MGI Symbol;Acc:MGI:3646343]	2.426	1.48E-02
ENSMUSG00000087107	AI662270	expressed sequence AI662270 [Source:MGI Symbol;Acc:MGI:2144254]	2.424	1.90E-05
ENSMUSG00000027398	Il1b	interleukin 1 beta [Source:MGI Symbol;Acc:MGI:96543]	2.422	2.37E-04
ENSMUSG00000026872	Zeb2	zinc finger E-box binding homeobox 2 [Source:MGI Symbol;Acc:MGI:1344407]	2.413	2.10E-10
ENSMUSG00000020886	Dlg4	discs large MAGUK scaffold protein 4 [Source:MGI Symbol;Acc:MGI:1277959]	2.413	6.29E-05
ENSMUSG00000095457	Gm8989	predicted gene 8989 [Source:MGI Symbol;Acc:MGI:3704114]	2.412	3.34E-04
ENSMUSG00000026103	Gls	glutaminase [Source:MGI Symbol;Acc:MGI:95752]	2.406	3.14E-18
ENSMUSG00000082436	Gm11688	predicted gene 11688 [Source:MGI Symbol;Acc:MGI:3650868]	2.405	4.41E-02
ENSMUSG00000073008	Gpr174	G protein-coupled receptor 174 [Source:MGI Symbol;Acc:MGI:2685222]	2.405	9.55E-03
ENSMUSG00000010067	Rassf1	Ras association (RalGDS/AF-6) domain family member 1 [Source:MGI Symbol;Acc:MGI:1928386]	2.405	5.00E-07
ENSMUSG00000106691	2700029L08Rik	RIKEN cDNA 2700029L08 gene [Source:MGI Symbol;Acc:MGI:1924056]	2.404	1.61E-02
ENSMUSG00000025997	Ikzf2	IKAROS family zinc finger 2 [Source:MGI Symbol;Acc:MGI:1342541]	2.403	3.67E-05
ENSMUSG00000026288	Inpp5d	inositol polyphosphate-5-phosphatase D [Source:MGI Symbol;Acc:MGI:107357]	2.403	2.74E-08
ENSMUSG00000038151	Prdm1	"PR domain containing 1, with ZNF domain [Source:MGI Symbol;Acc:MGI:99655]"	2.403	4.65E-03
ENSMUSG00000110972	5830462O15Rik	RIKEN cDNA 5830462O15 gene [Source:MGI Symbol;Acc:MGI:1923360]	2.402	3.85E-02
ENSMUSG00000030031	Kbtbd8	kelch repeat and BTB (POZ) domain containing 8 [Source:MGI Symbol;Acc:MGI:2661430]	2.402	6.64E-07
ENSMUSG00000078942	Naip6	"NLR family, apoptosis inhibitory protein 6 [Source:MGI Symbol;Acc:MGI:1298222]"	2.402	9.16E-12
ENSMUSG00000022901	Cd86	CD86 antigen [Source:MGI Symbol;Acc:MGI:101773]	2.398	1.73E-03
ENSMUSG00000027171	Prrg4	proline rich Gla (G-carboxyglutamic acid) 4 (transmembrane) [Source:MGI Symbol;Acc:MGI:2442211]	2.396	1.57E-03
ENSMUSG00000040663	Clcf1	cardiotrophin-like cytokine factor 1 [Source:MGI Symbol;Acc:MGI:1930088]	2.394	1.01E-03
ENSMUSG00000033952	Aspm	abnormal spindle microtubule assembly [Source:MGI Symbol;Acc:MGI:1334448]	2.391	8.24E-04
ENSMUSG00000115399	9430068D22Rik	RIKEN cDNA 9430068D22 gene [Source:MGI Symbol;Acc:MGI:1924554]	2.391	4.66E-02
ENSMUSG00000067341	H2-Eb2	"histocompatibility 2, class II antigen E beta2 [Source:MGI Symbol;Acc:MGI:95902]"	2.390	2.07E-02
ENSMUSG00000073491	Ifi213	interferon activated gene 213 [Source:MGI Symbol;Acc:MGI:3695276]	2.388	3.21E-10
ENSMUSG00000031832	Taf1c	"TATA-box binding protein associated factor, RNA polymerase I, C [Source:MGI Symbol;Acc:MGI:109576]"	2.386	2.57E-03
ENSMUSG00000025007	Aldh18a1	"aldehyde dehydrogenase 18 family, member A1 [Source:MGI Symbol;Acc:MGI:1888908]"	2.386	6.91E-05
ENSMUSG00000027859	Ngf	nerve growth factor [Source:MGI Symbol;Acc:MGI:97321]	2.385	6.98E-03
ENSMUSG00000071713	Csf2rb	"colony stimulating factor 2 receptor, beta, low-affinity (granulocyte-macrophage) [Source:MGI Symbol;Acc:MGI:1339759]"	2.384	4.63E-10
ENSMUSG00000002111	Spi1	spleen focus forming virus (SFFV) proviral integration oncogene [Source:MGI Symbol;Acc:MGI:98282]	2.384	4.92E-07
ENSMUSG00000091575	2010016I18Rik	RIKEN cDNA 2010016I18 gene [Source:MGI Symbol;Acc:MGI:1916456]	2.382	6.67E-04
ENSMUSG00000082185	Gm12428	predicted gene 12428 [Source:MGI Symbol;Acc:MGI:3650848]	2.379	6.57E-03
ENSMUSG00000118501	AC020971.1	protocadherin gamma subfamily (PCDHG) pseudogene.	2.379	5.79E-03
ENSMUSG00000052684	Jun	jun proto-oncogene [Source:MGI Symbol;Acc:MGI:96646]	2.378	8.72E-13
ENSMUSG00000036902	Neto2	neuropilin (NRP) and tolloid (TLL)-like 2 [Source:MGI Symbol;Acc:MGI:1921763]	2.378	8.64E-03
ENSMUSG00000046805	Mpeg1	macrophage expressed gene 1 [Source:MGI Symbol;Acc:MGI:1333743]	2.378	3.86E-13
ENSMUSG00000032113	Chek1	checkpoint kinase 1 [Source:MGI Symbol;Acc:MGI:1202065]	2.375	1.37E-03
ENSMUSG00000074480	Mex3a	mex3 RNA binding family member A [Source:MGI Symbol;Acc:MGI:1919890]	2.375	4.33E-02
ENSMUSG00000020941	Map3k14	mitogen-activated protein kinase kinase kinase 14 [Source:MGI Symbol;Acc:MGI:1858204]	2.374	2.87E-04
ENSMUSG00000037185	Krt80	keratin 80 [Source:MGI Symbol;Acc:MGI:1921377]	2.374	2.02E-02
ENSMUSG00000034438	Gbp8	guanylate-binding protein 8 [Source:MGI Symbol;Acc:MGI:1923324]	2.372	6.21E-07
ENSMUSG00000110419	AW046200	expressed sequence AW046200 [Source:MGI Symbol;Acc:MGI:2142745]	2.371	1.04E-02
ENSMUSG00000053303	Slc22a26	"solute carrier family 22 (organic cation transporter), member 26 [Source:MGI Symbol;Acc:MGI:2385316]"	2.364	1.21E-02
ENSMUSG00000109727	Gm45464	predicted gene 45464 [Source:MGI Symbol;Acc:MGI:5791300]	2.363	3.56E-02
ENSMUSG00000052760	A630001G21Rik	RIKEN cDNA A630001G21 gene [Source:MGI Symbol;Acc:MGI:2443131]	2.362	1.04E-04
ENSMUSG00000032374	Plod2	"procollagen lysine, 2-oxoglutarate 5-dioxygenase 2 [Source:MGI Symbol;Acc:MGI:1347007]"	2.361	3.98E-03
ENSMUSG00000032691	Nlrp3	"NLR family, pyrin domain containing 3 [Source:MGI Symbol;Acc:MGI:2653833]"	2.359	3.33E-02
ENSMUSG00000053980	Gm9930	predicted gene 9930 [Source:MGI Symbol;Acc:MGI:3642400]	2.357	8.83E-03
ENSMUSG00000107478	Gm45234	predicted gene 45234 [Source:MGI Symbol;Acc:MGI:5753810]	2.354	2.19E-02
ENSMUSG00000020914	Top2a	topoisomerase (DNA) II alpha [Source:MGI Symbol;Acc:MGI:98790]	2.353	2.75E-07
ENSMUSG00000006445	Epha2	Eph receptor A2 [Source:MGI Symbol;Acc:MGI:95278]	2.350	1.98E-05

ENSMUSG00000008193	Spib	Spi-B transcription factor (Spi-1/PU.1 related) [Source:MGI Symbol;Acc:MGI:892986]	2.347	4.31E-03
ENSMUSG00000109841	E330011O21Rik	RIKEN cDNA E330011O21 gene [Source:MGI Symbol;Acc:MGI:3759668]	2.346	2.21E-03
ENSMUSG00000021720	Rnf180	ring finger protein 180 [Source:MGI Symbol;Acc:MGI:1919066]	2.346	9.90E-03
ENSMUSG00000029675	Eln	elastin [Source:MGI Symbol;Acc:MGI:95317]	2.344	2.23E-04
ENSMUSG00000033685	Ucp2	"uncoupling protein 2 (mitochondrial, proton carrier) [Source:MGI Symbol;Acc:MGI:109354]"	2.342	4.39E-10
ENSMUSG00000114996	Gm48958	"predicted gene, 48958 [Source:MGI Symbol;Acc:MGI:6118289]"	2.342	5.89E-07
ENSMUSG00000023169	Slc38a1	"solute carrier family 38, member 1 [Source:MGI Symbol;Acc:MGI:2145895]"	2.341	1.62E-10
ENSMUSG00000113649	Gm47665	"predicted gene, 47665 [Source:MGI Symbol;Acc:MGI:6096756]"	2.340	1.53E-02
ENSMUSG00000073176	Zfp449	zinc finger protein 449 [Source:MGI Symbol;Acc:MGI:1925869]	2.340	5.67E-03
ENSMUSG00000105720	Gm42440	predicted gene 42440 [Source:MGI Symbol;Acc:MGI:5662577]	2.339	4.14E-02
ENSMUSG00000040061	Plcb2	"phospholipase C, beta 2 [Source:MGI Symbol;Acc:MGI:107465]"	2.338	6.40E-04
ENSMUSG00000041153	Osgin2	oxidative stress induced growth inhibitor family member 2 [Source:MGI Symbol;Acc:MGI:2384798]	2.338	3.95E-05
ENSMUSG00000029561	Oasl2	2'-5' oligoadenylate synthetase-like 2 [Source:MGI Symbol;Acc:MGI:1344390]	2.338	4.00E-07
ENSMUSG00000029591	Ung	uracil DNA glycosylase [Source:MGI Symbol;Acc:MGI:109352]	2.337	9.06E-06
ENSMUSG00000040118	Cacna2d1	"calcium channel, voltage-dependent, alpha2/delta subunit 1 [Source:MGI Symbol;Acc:MGI:88295]"	2.336	2.04E-03
ENSMUSG00000102344	9430053O09Rik	RIKEN cDNA 9430053O09 gene [Source:MGI Symbol;Acc:MGI:2442604]	2.335	3.13E-02
ENSMUSG00000038702	Dsel	dermatan sulfate epimerase-like [Source:MGI Symbol;Acc:MGI:2442948]	2.335	3.82E-03
ENSMUSG00000027641	Rbl1	RB transcriptional corepressor like 1 [Source:MGI Symbol;Acc:MGI:103300]	2.334	7.67E-07
ENSMUSG00000055805	Fmnl1	formin-like 1 [Source:MGI Symbol;Acc:MGI:1888994]	2.334	1.53E-05
ENSMUSG00000026683	Nuf2	"NUF2, NDC80 kinetochore complex component [Source:MGI Symbol;Acc:MGI:1914227]"	2.332	1.42E-02
ENSMUSG00000036944	Tmem71	transmembrane protein 71 [Source:MGI Symbol;Acc:MGI:2146049]	2.330	3.48E-03
ENSMUSG00000032294	Pkm	"pyruvate kinase, muscle [Source:MGI Symbol;Acc:MGI:97591]"	2.330	8.13E-06
ENSMUSG00000033697	Arhgap39	Rho GTPase activating protein 39 [Source:MGI Symbol;Acc:MGI:107858]	2.327	6.09E-05
ENSMUSG00000069793	Slfn9	schlafen 9 [Source:MGI Symbol;Acc:MGI:2445121]	2.327	8.88E-10
ENSMUSG00000099954	Gm28112	predicted gene 28112 [Source:MGI Symbol;Acc:MGI:5578818]	2.326	1.48E-02
ENSMUSG00000051682	Treml4	triggering receptor expressed on myeloid cells-like 4 [Source:MGI Symbol;Acc:MGI:1923239]	2.326	3.47E-04
ENSMUSG00000028832	Stmn1	stathmin 1 [Source:MGI Symbol;Acc:MGI:96739]	2.326	6.90E-03
ENSMUSG00000026355	Mcm6	minichromosome maintenance complex component 6 [Source:MGI Symbol;Acc:MGI:1298227]	2.326	3.13E-07
ENSMUSG00000029482	Aacs	acetoacetyl-CoA synthetase [Source:MGI Symbol;Acc:MGI:1926144]	2.322	1.17E-05
ENSMUSG00000070868	Skint3	selection and upkeep of intraepithelial T cells 3 [Source:MGI Symbol;Acc:MGI:3045331]	2.320	2.48E-02
ENSMUSG00000102466	Gm38200	"predicted gene, 38200 [Source:MGI Symbol;Acc:MGI:5611428]"	2.320	4.11E-02
ENSMUSG00000030107	Usp18	ubiquitin specific peptidase 18 [Source:MGI Symbol;Acc:MGI:1344364]	2.318	3.27E-06
ENSMUSG00000018654	Ikzf1	IKAROS family zinc finger 1 [Source:MGI Symbol;Acc:MGI:1342540]	2.318	4.04E-06
ENSMUSG00000114496	Gm47601	"predicted gene, 47601 [Source:MGI Symbol;Acc:MGI:6096654]"	2.313	1.39E-02
ENSMUSG00000036040	Adamtsl2	ADAMTS-like 2 [Source:MGI Symbol;Acc:MGI:1925044]	2.312	1.33E-05
ENSMUSG00000090622	A930033H14Rik	RIKEN cDNA A930033H14 gene [Source:MGI Symbol;Acc:MGI:2444562]	2.311	2.34E-03
ENSMUSG00000071103	1700029J07Rik	RIKEN cDNA 1700029J07 gene [Source:MGI Symbol;Acc:MGI:1916729]	2.311	2.15E-02
ENSMUSG00000038608	Dock10	dedicator of cytokinesis 10 [Source:MGI Symbol;Acc:MGI:2146320]	2.311	9.10E-14
ENSMUSG00000042190	Cmklr1	chemokine-like receptor 1 [Source:MGI Symbol;Acc:MGI:109603]	2.311	1.52E-03
ENSMUSG00000026547	Tagln2	transgelin 2 [Source:MGI Symbol;Acc:MGI:1312985]	2.310	1.14E-04
ENSMUSG00000047735	Samd9l	sterile alpha motif domain containing 9-like [Source:MGI Symbol;Acc:MGI:1343184]	2.307	3.36E-14
ENSMUSG00000033581	Igf2bp2	insulin-like growth factor 2 mRNA binding protein 2 [Source:MGI Symbol;Acc:MGI:1890358]	2.307	3.97E-02
ENSMUSG00000034459	Ifit1	interferon-induced protein with tetratricopeptide repeats 1 [Source:MGI Symbol;Acc:MGI:99450]	2.307	3.31E-08
ENSMUSG00000059498	Fcgr3	"Fc receptor, IgG, low affinity III [Source:MGI Symbol;Acc:MGI:95500]"	2.306	2.55E-05
ENSMUSG00000032802	Srxn1	sulfiredoxin 1 homolog (S. cerevisiae) [Source:MGI Symbol;Acc:MGI:104971]	2.305	6.72E-18
ENSMUSG00000071203	Naip5	"NLR family, apoptosis inhibitory protein 5 [Source:MGI Symbol;Acc:MGI:1298220]"	2.302	1.83E-07
ENSMUSG00000104891	2510017J16Rik	RIKEN cDNA 2510017J16 gene [Source:MGI Symbol;Acc:MGI:1913824]	2.301	2.37E-02
ENSMUSG00000043889	Gm8399	predicted gene 8399 [Source:MGI Symbol;Acc:MGI:3647971]	2.301	7.65E-06
ENSMUSG00000099757	BE692007	expressed sequence BE692007 [Source:MGI Symbol;Acc:MGI:3035348]	2.300	4.47E-05
ENSMUSG00000049804	Armcx4	"armadillo repeat containing, X-linked 4 [Source:MGI Symbol;Acc:MGI:2147887]"	2.299	2.49E-07

ENSMUSG00000031165	Was	Wiskott-Aldrich syndrome [Source:MGI Symbol;Acc:MGI:105059]	2.299	1.21E-03
ENSMUSG00000039936	Pik3cd	"phosphatidylinositol-4,5-bisphosphate 3-kinase catalytic subunit delta [Source:MGI Symbol;Acc:MGI:1098211]"	2.298	2.65E-05
ENSMUSG00000022440	C1qtnf6	C1q and tumor necrosis factor related protein 6 [Source:MGI Symbol;Acc:MGI:1919959]	2.297	4.66E-02
ENSMUSG00000103899	Gm37940	"predicted gene, 37940 [Source:MGI Symbol;Acc:MGI:5611168]"	2.297	2.01E-06
ENSMUSG00000078956	Gm14221	predicted gene 14221 [Source:MGI Symbol;Acc:MGI:3650696]	2.295	4.18E-03
ENSMUSG00000031662	Snx20	sorting nexin 20 [Source:MGI Symbol;Acc:MGI:1918857]	2.292	5.77E-04
ENSMUSG00000097217	Gm26549	"predicted gene, 26549 [Source:MGI Symbol;Acc:MGI:5477043]"	2.292	4.12E-02
ENSMUSG00000033721	Vav3	vav 3 oncogene [Source:MGI Symbol;Acc:MGI:1888518]	2.292	3.38E-05
ENSMUSG00000112944	Gm48885	"predicted gene, 48885 [Source:MGI Symbol;Acc:MGI:6098643]"	2.291	1.86E-04
ENSMUSG00000001870	Ltbp1	latent transforming growth factor beta binding protein 1 [Source:MGI Symbol;Acc:MGI:109151]	2.290	3.94E-03
ENSMUSG00000038305	Spats2l	"spermatogenesis associated, serine-rich 2-like [Source:MGI Symbol;Acc:MGI:1914448]"	2.287	2.82E-02
ENSMUSG00000031802	Phxr4	per-hexamer repeat gene 4 [Source:MGI Symbol;Acc:MGI:104522]	2.287	2.76E-04
ENSMUSG00000038623	Tm6sf1	transmembrane 6 superfamily member 1 [Source:MGI Symbol;Acc:MGI:1933209]	2.284	7.35E-11
ENSMUSG00000020573	Pik3cg	"phosphatidylinositol-4,5-bisphosphate 3-kinase catalytic subunit gamma [Source:MGI Symbol;Acc:MGI:1353576]"	2.284	4.28E-07
ENSMUSG00000039005	Tlr4	toll-like receptor 4 [Source:MGI Symbol;Acc:MGI:96824]	2.284	2.41E-07
ENSMUSG00000036943	Rab8b	"RAB8B, member RAS oncogene family [Source:MGI Symbol;Acc:MGI:2442982]"	2.281	1.20E-09
ENSMUSG00000041488	Stx3	syntaxin 3 [Source:MGI Symbol;Acc:MGI:103077]	2.281	1.11E-04
ENSMUSG00000015355	Cd48	CD48 antigen [Source:MGI Symbol;Acc:MGI:88339]	2.281	1.86E-03
ENSMUSG00000089706	B230216N24Rik	RIKEN cDNA B230216N24 gene [Source:MGI Symbol;Acc:MGI:1925853]	2.280	4.18E-08
ENSMUSG00000029314	Gpat3	glycerol-3-phosphate acyltransferase 3 [Source:MGI Symbol;Acc:MGI:3603816]	2.280	1.74E-08
ENSMUSG00000070284	Gmppb	GDP-mannose pyrophosphorylase B [Source:MGI Symbol;Acc:MGI:2660880]	2.279	7.39E-09
ENSMUSG00000079442	St6galnac4	"ST6 (alpha-N-acetyl-neuraminyl-2,3-beta-galactosyl-1,3)-N-acetylgalactosaminide alpha-2,6-sialyltransferase 4 [Source:MGI Symbol;Acc:MGI:1341894]"	2.279	4.38E-03
ENSMUSG00000052013	Btla	B and T lymphocyte associated [Source:MGI Symbol;Acc:MGI:2658978]	2.278	1.29E-04
ENSMUSG00000022378	Fam49b	"family with sequence similarity 49, member B [Source:MGI Symbol;Acc:MGI:1923520]"	2.275	6.39E-21
ENSMUSG00000031103	Elf4	E74-like factor 4 (ets domain transcription factor) [Source:MGI Symbol;Acc:MGI:1928377]	2.275	2.36E-06
ENSMUSG00000024795	Kif20b	kinesin family member 20B [Source:MGI Symbol;Acc:MGI:2444576]	2.274	1.13E-03
ENSMUSG00000074978	Actg-ps1	"actin, gamma, pseudogene 1 [Source:MGI Symbol;Acc:MGI:87907]"	2.273	2.20E-02
ENSMUSG00000060063	Alox5ap	arachidonate 5-lipoxygenase activating protein [Source:MGI Symbol;Acc:MGI:107505]	2.272	4.79E-03
ENSMUSG00000019979	Apaf1	apoptotic peptidase activating factor 1 [Source:MGI Symbol;Acc:MGI:1306796]	2.271	4.75E-10
ENSMUSG00000108359	C430039J01Rik	RIKEN cDNA C430039J01 gene [Source:MGI Symbol;Acc:MGI:1924901]	2.268	7.48E-03
ENSMUSG00000039542	Ncam1	neural cell adhesion molecule 1 [Source:MGI Symbol;Acc:MGI:97281]	2.268	3.64E-02
ENSMUSG00000026110	Mgat4a	"mannoside acetylglucosaminyltransferase 4, isoenzyme A [Source:MGI Symbol;Acc:MGI:2662992]"	2.267	7.36E-07
ENSMUSG00000030554	Synm	"synemin, intermediate filament protein [Source:MGI Symbol;Acc:MGI:2661187]"	2.266	3.11E-02
ENSMUSG00000047492	Inhbe	inhibin beta-E [Source:MGI Symbol;Acc:MGI:109269]	2.265	6.50E-18
ENSMUSG00000031093	Dock11	dedicator of cytokinesis 11 [Source:MGI Symbol;Acc:MGI:1923224]	2.264	1.73E-07
ENSMUSG00000030774	Pak1	p21 (RAC1) activated kinase 1 [Source:MGI Symbol;Acc:MGI:1339975]	2.262	2.26E-06
ENSMUSG00000000682	Cd52	CD52 antigen [Source:MGI Symbol;Acc:MGI:1346088]	2.262	1.70E-03
ENSMUSG00000050721	Plekho2	"pleckstrin homology domain containing, family O member 2 [Source:MGI Symbol;Acc:MGI:2143132]"	2.262	4.15E-07
ENSMUSG00000020589	Fam49a	"family with sequence similarity 49, member A [Source:MGI Symbol;Acc:MGI:1261783]"	2.261	3.82E-08
ENSMUSG00000081058	H3c15	H3 clustered histone 15 [Source:MGI Symbol;Acc:MGI:2448357]	2.261	9.73E-03
ENSMUSG00000044748	Defb1	defensin beta 1 [Source:MGI Symbol;Acc:MGI:1096878]	2.261	1.90E-04
ENSMUSG00000003418	St8sia6	"ST8 alpha-N-acetyl-neuraminide alpha-2,8-sialyltransferase 6 [Source:MGI Symbol;Acc:MGI:2386797]"	2.259	3.04E-02
ENSMUSG00000102133	Gm37106	"predicted gene, 37106 [Source:MGI Symbol;Acc:MGI:5610334]"	2.259	4.50E-02
ENSMUSG00000102516	Gm38340	"predicted gene, 38340 [Source:MGI Symbol;Acc:MGI:5611568]"	2.257	2.88E-04
ENSMUSG00000115222	Gm49747	"predicted gene, 49747 [Source:MGI Symbol;Acc:MGI:6215240]"	2.255	1.04E-03
ENSMUSG00000002068	Ccne1	cyclin E1 [Source:MGI Symbol;Acc:MGI:88316]	2.253	1.58E-02
ENSMUSG00000113894	Gm48838	"predicted gene, 48838 [Source:MGI Symbol;Acc:MGI:6098568]"	2.253	2.60E-05
ENSMUSG00000110141	Gm45684	predicted gene 45684 [Source:MGI Symbol;Acc:MGI:5791520]	2.252	1.90E-02
ENSMUSG00000035967	Ints6l	integrator complex subunit 6 like [Source:MGI Symbol;Acc:MGI:2442593]	2.250	1.73E-10
ENSMUSG00000075585	6330403L08Rik	RIKEN cDNA 6330403L08 gene [Source:MGI Symbol;Acc:MGI:1917994]	2.249	6.20E-04

ENSMUSG00000026994	Galnt3	polypeptide N-acetylgalactosaminyltransferase 3 [Source:MGI Symbol;Acc:MGI:894695]	2.249	2.04E-02
ENSMUSG00000020227	Irak3	interleukin-1 receptor-associated kinase 3 [Source:MGI Symbol;Acc:MGI:1921164]	2.247	2.11E-02
ENSMUSG00000094812	Gm22614	"predicted gene, 22614 [Source:MGI Symbol;Acc:MGI:5452391]"	2.247	1.63E-07
ENSMUSG00000026177	Slc11a1	"solute carrier family 11 (proton-coupled divalent metal ion transporters), member 1 [Source:MGI Symbol;Acc:MGI:1345275]"	2.247	9.24E-09
ENSMUSG00000053846	Lipg	"lipase, endothelial [Source:MGI Symbol;Acc:MGI:1341803]"	2.246	1.18E-06
ENSMUSG00000037552	Plekhg2	"pleckstrin homology domain containing, family G (with RhoGef domain) member 2 [Source:MGI Symbol;Acc:MGI:2141874]"	2.246	1.52E-02
ENSMUSG00000041515	Irf8	interferon regulatory factor 8 [Source:MGI Symbol;Acc:MGI:96395]	2.245	1.11E-05
ENSMUSG00000070392	Gm20634	predicted gene 20634 [Source:MGI Symbol;Acc:MGI:5313081]	2.244	1.86E-03
ENSMUSG00000069607	Cd300ld3	CD300 molecule like family member D3 [Source:MGI Symbol;Acc:MGI:2687214]	2.244	2.78E-02
ENSMUSG00000082503	Gm12229	predicted gene 12229 [Source:MGI Symbol;Acc:MGI:3649743]	2.242	1.48E-02
ENSMUSG00000009687	Fxyd5	FXYD domain-containing ion transport regulator 5 [Source:MGI Symbol;Acc:MGI:1201785]	2.242	5.48E-05
ENSMUSG00000078502	Gm13212	predicted gene 13212 [Source:MGI Symbol;Acc:MGI:3651014]	2.242	4.02E-07
ENSMUSG00000041112	Elmo1	engulfment and cell motility 1 [Source:MGI Symbol;Acc:MGI:2153044]	2.242	2.73E-10
ENSMUSG00000053182	Gm609	predicted gene 609 [Source:MGI Symbol;Acc:MGI:2685455]	2.241	2.07E-02
ENSMUSG00000027326	Knl1	kinetochore scaffold 1 [Source:MGI Symbol;Acc:MGI:1923714]	2.240	3.63E-03
ENSMUSG00000012428	Steap4	STEAP family member 4 [Source:MGI Symbol;Acc:MGI:1923560]	2.239	1.09E-17
ENSMUSG00000021692	Dimt1	DIM1 dimethyladenosine transferase 1-like (S. cerevisiae) [Source:MGI Symbol;Acc:MGI:1913504]	2.238	6.74E-04
ENSMUSG00000023073	Slc10a2	"solute carrier family 10, member 2 [Source:MGI Symbol;Acc:MGI:1201406]"	2.236	1.41E-12
ENSMUSG00000018819	Lsp1	lymphocyte specific 1 [Source:MGI Symbol;Acc:MGI:96832]	2.236	1.13E-04
ENSMUSG00000104388	Gm37033	"predicted gene, 37033 [Source:MGI Symbol;Acc:MGI:5610261]"	2.235	5.63E-14
ENSMUSG00000071037	Camkmt	calmodulin-lysine N-methyltransferase [Source:MGI Symbol;Acc:MGI:1920832]	2.233	8.23E-03
ENSMUSG00000097705	Gm26740	"predicted gene, 26740 [Source:MGI Symbol;Acc:MGI:5477234]"	2.233	2.31E-04
ENSMUSG00000062210	Tnfaip8	"tumor necrosis factor, alpha-induced protein 8 [Source:MGI Symbol;Acc:MGI:2147191]"	2.231	1.38E-04
ENSMUSG00000021948	Prkcd	"protein kinase C, delta [Source:MGI Symbol;Acc:MGI:97598]"	2.231	4.81E-12
ENSMUSG00000054277	Arfgap3	ADP-ribosylation factor GTPase activating protein 3 [Source:MGI Symbol;Acc:MGI:1913501]	2.230	3.42E-08
ENSMUSG00000047747	Rnf150	ring finger protein 150 [Source:MGI Symbol;Acc:MGI:2443860]	2.230	6.89E-05
ENSMUSG00000001918	Slc1a5	"solute carrier family 1 (neutral amino acid transporter), member 5 [Source:MGI Symbol;Acc:MGI:105305]"	2.229	2.22E-02
ENSMUSG00000044770	Scml4	Scm polycomb group protein like 4 [Source:MGI Symbol;Acc:MGI:2446140]	2.228	3.17E-02
ENSMUSG00000028370	Pappa	pregnancy-associated plasma protein A [Source:MGI Symbol;Acc:MGI:97479]	2.227	2.49E-02
ENSMUSG00000037921	Ddx60	DEAD (Asp-Glu-Ala-Asp) box polypeptide 60 [Source:MGI Symbol;Acc:MGI:2384570]	2.226	2.70E-10
ENSMUSG00000000385	Tmprss2	"transmembrane protease, serine 2 [Source:MGI Symbol;Acc:MGI:1354381]"	2.226	1.67E-06
ENSMUSG00000009633	G0s2	G0/G1 switch gene 2 [Source:MGI Symbol;Acc:MGI:1316737]	2.226	1.15E-06
ENSMUSG00000049625	Tifab	"TRAF-interacting protein with forkhead-associated domain, family member B [Source:MGI Symbol;Acc:MGI:2385852]"	2.224	7.84E-05
ENSMUSG00000072647	Adam1a	a disintegrin and metallopeptidase domain 1a [Source:MGI Symbol;Acc:MGI:2429504]	2.223	3.96E-02
ENSMUSG00000015599	Ttbk1	tau tubulin kinase 1 [Source:MGI Symbol;Acc:MGI:2147036]	2.222	4.61E-03
ENSMUSG00000031709	Tbc1d9	"TBC1 domain family, member 9 [Source:MGI Symbol;Acc:MGI:1918560]"	2.221	1.74E-07
ENSMUSG00000041827	Oasl1	2'-5' oligoadenylate synthetase-like 1 [Source:MGI Symbol;Acc:MGI:2180849]	2.219	1.35E-05
ENSMUSG00000056394	Lig1	"ligase I, DNA, ATP-dependent [Source:MGI Symbol;Acc:MGI:101789]"	2.215	9.32E-03
ENSMUSG00000082361	Btc	"betacellulin, epidermal growth factor family member [Source:MGI Symbol;Acc:MGI:99439]"	2.214	3.47E-07
ENSMUSG00000085939	Cd63-ps	"CD63 antigen, pseudogene [Source:MGI Symbol;Acc:MGI:105972]"	2.211	4.71E-03
ENSMUSG00000027544	Nfatc2	"nuclear factor of activated T cells, cytoplasmic, calcineurin dependent 2 [Source:MGI Symbol;Acc:MGI:102463]"	2.210	3.25E-02
ENSMUSG00000030256	Bhlhe41	"basic helix-loop-helix family, member e41 [Source:MGI Symbol;Acc:MGI:1930704]"	2.210	7.93E-03
ENSMUSG00000008496	Pou2f2	"POU domain, class 2, transcription factor 2 [Source:MGI Symbol;Acc:MGI:101897]"	2.210	3.78E-05
ENSMUSG00000037280	Galnt6	polypeptide N-acetylgalactosaminyltransferase 6 [Source:MGI Symbol;Acc:MGI:1891640]	2.209	1.26E-03
ENSMUSG00000028581	Laptm5	lysosomal-associated protein transmembrane 5 [Source:MGI Symbol;Acc:MGI:108046]	2.209	2.23E-06
ENSMUSG00000035692	Isg15	ISG15 ubiquitin-like modifier [Source:MGI Symbol;Acc:MGI:1855694]	2.209	2.96E-04
ENSMUSG00000106475	Gm43011	predicted gene 43011 [Source:MGI Symbol;Acc:MGI:5663148]	2.207	2.22E-08
ENSMUSG00000022665	Ccdc80	coiled-coil domain containing 80 [Source:MGI Symbol;Acc:MGI:1915146]	2.206	1.38E-04
ENSMUSG00000058818	Pirb	paired Ig-like receptor B [Source:MGI Symbol;Acc:MGI:894311]	2.206	1.53E-06
ENSMUSG00000108522	Gm44664	predicted gene 44664 [Source:MGI Symbol;Acc:MGI:5753240]	2.206	7.26E-03

ENSMUSG00000028843	Sh3bgrl3	SH3 domain binding glutamic acid-rich protein-like 3 [Source:MGI Symbol;Acc:MGI:1920973]	2.205	2.74E-03
ENSMUSG00000108738	Gm44777	predicted gene 44777 [Source:MGI Symbol;Acc:MGI:5753353]	2.205	9.54E-04
ENSMUSG00000034205	Loxl2	lysyl oxidase-like 2 [Source:MGI Symbol;Acc:MGI:2137913]	2.204	1.01E-04
ENSMUSG00000039911	Spsb1	splA/ryanodine receptor domain and SOCS box containing 1 [Source:MGI Symbol;Acc:MGI:1921896]	2.203	7.45E-04
ENSMUSG00000102831	Gm37382	"predicted gene, 37382 [Source:MGI Symbol;Acc:MGI:5610610]"	2.201	7.30E-03
ENSMUSG00000021177	Tdp1	tyrosyl-DNA phosphodiesterase 1 [Source:MGI Symbol;Acc:MGI:1920036]	2.200	2.45E-02
ENSMUSG00000024897	Apba1	"amyloid beta (A4) precursor protein binding, family A, member 1 [Source:MGI Symbol;Acc:MGI:1860297]"	2.199	7.57E-03
ENSMUSG00000033538	Casp4	"caspase 4, apoptosis-related cysteine peptidase [Source:MGI Symbol;Acc:MGI:107700]"	2.199	8.15E-04
ENSMUSG00000109378	Gm49396	"predicted gene, 49396 [Source:MGI Symbol;Acc:MGI:6121629]"	2.198	6.41E-03
ENSMUSG00000030707	Coro1a	"coronin, actin binding protein 1A [Source:MGI Symbol;Acc:MGI:1345961]"	2.198	1.44E-05
ENSMUSG00000117869	Snhg4	small nucleolar RNA host gene 4 [Source:MGI Symbol;Acc:MGI:4937091]	2.197	8.22E-04
ENSMUSG00000112736	Gm48718	"predicted gene, 48718 [Source:MGI Symbol;Acc:MGI:6098369]"	2.197	3.68E-02
ENSMUSG00000039697	Ncoa7	nuclear receptor coactivator 7 [Source:MGI Symbol;Acc:MGI:2444847]	2.196	7.53E-05
ENSMUSG00000032311	Nrg4	neuregulin 4 [Source:MGI Symbol;Acc:MGI:1933833]	2.195	1.69E-05
ENSMUSG00000057329	Bcl2	B cell leukemia/lymphoma 2 [Source:MGI Symbol;Acc:MGI:88138]	2.195	1.66E-07
ENSMUSG00000107178	Gm42531	predicted gene 42531 [Source:MGI Symbol;Acc:MGI:5662668]	2.195	2.06E-02
ENSMUSG00000108169	Gm43958	"predicted gene, 43958 [Source:MGI Symbol;Acc:MGI:5690350]"	2.193	4.90E-03
ENSMUSG00000043832	Clec4a3	"C-type lectin domain family 4, member a3 [Source:MGI Symbol;Acc:MGI:1920399]"	2.192	3.30E-04
ENSMUSG00000060671	Atp8b2	"ATPase, class I, type 8B, member 2 [Source:MGI Symbol;Acc:MGI:1859660]"	2.191	4.14E-03
ENSMUSG00000000489	Pdgfb	"platelet derived growth factor, B polypeptide [Source:MGI Symbol;Acc:MGI:97528]"	2.191	1.04E-02
ENSMUSG00000109585	Gm45358	predicted gene 45358 [Source:MGI Symbol;Acc:MGI:5791194]	2.190	9.76E-03
ENSMUSG00000001270	Ckb	"creatine kinase, brain [Source:MGI Symbol;Acc:MGI:88407]"	2.188	3.64E-02
ENSMUSG00000056124	B4galt6	"UDP-Gal:betaGlcNAc beta 1,4-galactosyltransferase, polypeptide 6 [Source:MGI Symbol;Acc:MGI:1928380]"	2.187	3.34E-06
ENSMUSG00000086813	Gm13657	predicted gene 13657 [Source:MGI Symbol;Acc:MGI:3650031]	2.186	6.22E-03
ENSMUSG00000003452	Bicd1	BICD cargo adaptor 1 [Source:MGI Symbol;Acc:MGI:1101760]	2.183	3.68E-02
ENSMUSG00000020142	Slc1a4	"solute carrier family 1 (glutamate/neutral amino acid transporter), member 4 [Source:MGI Symbol;Acc:MGI:2135601]"	2.183	1.93E-06
ENSMUSG00000013707	Tnfaip8l2	"tumor necrosis factor, alpha-induced protein 8-like 2 [Source:MGI Symbol;Acc:MGI:1917019]"	2.182	2.17E-02
ENSMUSG00000031712	Il15	interleukin 15 [Source:MGI Symbol;Acc:MGI:103014]	2.181	8.63E-03
ENSMUSG00000035778	Ggta1	"glycoprotein galactosyltransferase alpha 1, 3 [Source:MGI Symbol;Acc:MGI:95704]"	2.181	1.13E-03
ENSMUSG00000002983	Relb	avian reticuloendotheliosis viral (v-rel) oncogene related B [Source:MGI Symbol;Acc:MGI:103289]	2.179	6.64E-06
ENSMUSG00000068220	Lgals1	"lectin, galactose binding, soluble 1 [Source:MGI Symbol;Acc:MGI:96777]"	2.176	1.04E-03
ENSMUSG00000035126	Wdr78	WD repeat domain 78 [Source:MGI Symbol;Acc:MGI:2385328]	2.176	3.48E-02
ENSMUSG00000047415	Gpr68	G protein-coupled receptor 68 [Source:MGI Symbol;Acc:MGI:2441763]	2.175	2.65E-02
ENSMUSG00000089407	Gm24878	"predicted gene, 24878 [Source:MGI Symbol;Acc:MGI:5454655]"	2.173	2.01E-02
ENSMUSG00000035004	Igsf6	"immunoglobulin superfamily, member 6 [Source:MGI Symbol;Acc:MGI:1891393]"	2.172	3.85E-07
ENSMUSG00000096780	Tmem181b-ps	"transmembrane protein 181B, pseudogene [Source:MGI Symbol;Acc:MGI:3779544]"	2.171	1.96E-03
ENSMUSG00000040483	Xaf1	XIAP associated factor 1 [Source:MGI Symbol;Acc:MGI:3772572]	2.170	2.82E-07
ENSMUSG00000027188	Pamr1	peptidase domain containing associated with muscle regeneration 1 [Source:MGI Symbol;Acc:MGI:2445082]	2.169	7.51E-05
ENSMUSG00000096751	Gm28373	predicted gene 28373 [Source:MGI Symbol;Acc:MGI:5579079]	2.169	2.32E-03
ENSMUSG00000025870	Arl10	ADP-ribosylation factor-like 10 [Source:MGI Symbol;Acc:MGI:1930788]	2.167	2.75E-02
ENSMUSG00000071722	Spin4	"spindlin family, member 4 [Source:MGI Symbol;Acc:MGI:2444925]"	2.167	4.88E-02
ENSMUSG00000062825	Actg1	"actin, gamma, cytoplasmic 1 [Source:MGI Symbol;Acc:MGI:87906]"	2.167	1.54E-11
ENSMUSG00000034317	Trim59	tripartite motif-containing 59 [Source:MGI Symbol;Acc:MGI:1914199]	2.165	3.50E-03
ENSMUSG00000058715	Fcer1g	"Fc receptor, IgE, high affinity I, gamma polypeptide [Source:MGI Symbol;Acc:MGI:95496]"	2.165	9.21E-05
ENSMUSG00000031843	Mphosph6	M phase phosphoprotein 6 [Source:MGI Symbol;Acc:MGI:1915783]	2.164	1.47E-02
ENSMUSG00000047822	Angptl8	angiopoietin-like 8 [Source:MGI Symbol;Acc:MGI:3643534]	2.164	1.27E-06
ENSMUSG00000106943	Dancr	differentiation antagonizing non-protein coding RNA [Source:MGI Symbol;Acc:MGI:1917286]	2.164	3.20E-03
ENSMUSG00000058099	Nfam1	Nfat activating molecule with ITAM motif 1 [Source:MGI Symbol;Acc:MGI:1921289]	2.162	2.31E-07
ENSMUSG00000048938	Nr1h5	"nuclear receptor subfamily 1, group H, member 5 [Source:MGI Symbol;Acc:MGI:3026618]"	2.162	4.86E-05
ENSMUSG00000026390	Marco	macrophage receptor with collagenous structure [Source:MGI Symbol;Acc:MGI:1309998]	2.160	1.59E-02

ENSMUSG00000030022	Adamts9	"a disintegrin-like and metallopeptidase (reprolysin type) with thrombospondin type 1 motif, 9 [Source:MGI Symbol;Acc:MGI:1916320]"	2.158	5.08E-09
ENSMUSG00000051506	Wdfy4	WD repeat and FYVE domain containing 4 [Source:MGI Symbol;Acc:MGI:3584510]	2.158	3.95E-10
ENSMUSG00000035158	Mitf	melanogenesis associated transcription factor [Source:MGI Symbol;Acc:MGI:104554]	2.157	6.02E-05
ENSMUSG00000002458	Rgs19	regulator of G-protein signaling 19 [Source:MGI Symbol;Acc:MGI:1915153]	2.157	1.85E-03
ENSMUSG00000051220	Ercc6l	excision repair cross-complementing rodent repair deficiency complementation group 6 like [Source:MGI Symbol;Acc:MGI:2654144]	2.156	2.59E-02
ENSMUSG00000039316	Rftn1	raftlin lipid raft linker 1 [Source:MGI Symbol;Acc:MGI:1923688]	2.155	7.74E-03
ENSMUSG00000006519	Cyba	"cytochrome b-245, alpha polypeptide [Source:MGI Symbol;Acc:MGI:1316658]"	2.155	1.01E-05
ENSMUSG00000035208	Slfn8	schlafen 8 [Source:MGI Symbol;Acc:MGI:2672859]	2.154	3.96E-05
ENSMUSG00000105586	Gm43009	predicted gene 43009 [Source:MGI Symbol;Acc:MGI:5663146]	2.154	4.24E-07
ENSMUSG00000029913	Prdm5	PR domain containing 5 [Source:MGI Symbol;Acc:MGI:1918029]	2.151	1.26E-02
ENSMUSG00000024737	Slc15a3	"solute carrier family 15, member 3 [Source:MGI Symbol;Acc:MGI:1929691]"	2.150	9.15E-09
ENSMUSG00000106032	Gm42463	predicted gene 42463 [Source:MGI Symbol;Acc:MGI:5662600]	2.148	1.12E-02
ENSMUSG00000103227	Gm37733	"predicted gene, 37733 [Source:MGI Symbol;Acc:MGI:5610961]"	2.147	3.41E-04
ENSMUSG00000019261	Map1s	microtubule-associated protein 1S [Source:MGI Symbol;Acc:MGI:2443304]	2.146	1.06E-02
ENSMUSG00000097080	1700086O06Rik	RIKEN cDNA 1700086O06 gene [Source:MGI Symbol;Acc:MGI:1920766]	2.145	7.56E-03
ENSMUSG00000059791	Nrm	nurim (nuclear envelope membrane protein) [Source:MGI Symbol;Acc:MGI:2146855]	2.144	4.32E-02
ENSMUSG00000051444	Bbs12	Bardet-Biedl syndrome 12 (human) [Source:MGI Symbol;Acc:MGI:2686651]	2.144	3.55E-02
ENSMUSG00000037997	Parp11	"poly (ADP-ribose) polymerase family, member 11 [Source:MGI Symbol;Acc:MGI:2141505]"	2.143	2.29E-07
ENSMUSG00000105259	Gm42874	predicted gene 42874 [Source:MGI Symbol;Acc:MGI:5663011]	2.143	2.18E-02
ENSMUSG00000105848	Gm42683	predicted gene 42683 [Source:MGI Symbol;Acc:MGI:5662820]	2.142	1.70E-03
ENSMUSG00000037465	Klf10	Kruppel-like factor 10 [Source:MGI Symbol;Acc:MGI:1101353]	2.141	1.98E-06
ENSMUSG00000114255	Gm10734	predicted gene 10734 [Source:MGI Symbol;Acc:MGI:3642559]	2.140	1.91E-03
ENSMUSG00000015314	Slamf6	SLAM family member 6 [Source:MGI Symbol;Acc:MGI:1353620]	2.139	3.41E-02
ENSMUSG00000109231	Gm45737	predicted gene 45737 [Source:MGI Symbol;Acc:MGI:5804852]	2.138	2.95E-02
ENSMUSG00000097855	A930007I19Rik	RIKEN cDNA A930007I19 gene [Source:MGI Symbol;Acc:MGI:1925029]	2.136	3.42E-02
ENSMUSG00000033287	Kctd17	potassium channel tetramerisation domain containing 17 [Source:MGI Symbol;Acc:MGI:1920094]	2.135	2.49E-03
ENSMUSG00000034595	Ppp1r18	"protein phosphatase 1, regulatory subunit 18 [Source:MGI Symbol;Acc:MGI:1923698]"	2.133	3.65E-04
ENSMUSG00000030098	Grip2	glutamate receptor interacting protein 2 [Source:MGI Symbol;Acc:MGI:2681173]	2.132	1.48E-03
ENSMUSG00000020599	Rgs9	regulator of G-protein signaling 9 [Source:MGI Symbol;Acc:MGI:1338824]	2.132	3.56E-02
ENSMUSG00000023272	Creld2	cysteine-rich with EGF-like domains 2 [Source:MGI Symbol;Acc:MGI:1923987]	2.130	7.04E-09
ENSMUSG00000042759	Apobr	apolipoprotein B receptor [Source:MGI Symbol;Acc:MGI:2176230]	2.130	2.86E-03
ENSMUSG00000030921	Trim30a	tripartite motif-containing 30A [Source:MGI Symbol;Acc:MGI:98178]	2.129	1.65E-07
ENSMUSG00000037613	Tnfrsf23	"tumor necrosis factor receptor superfamily, member 23 [Source:MGI Symbol;Acc:MGI:1930269]"	2.129	1.75E-03
ENSMUSG00000052749	Trim30b	tripartite motif-containing 30B [Source:MGI Symbol;Acc:MGI:4821256]	2.129	3.88E-02
ENSMUSG00000030245	Golt1b	golgi transport 1B [Source:MGI Symbol;Acc:MGI:1914214]	2.127	2.51E-06
ENSMUSG00000107370	Gm43588	predicted gene 43588 [Source:MGI Symbol;Acc:MGI:5663725]	2.127	1.16E-02
ENSMUSG00000106643	Gm43422	predicted gene 43422 [Source:MGI Symbol;Acc:MGI:5663559]	2.124	2.77E-02
ENSMUSG00000102324	Gm19721	"predicted gene, 19721 [Source:MGI Symbol;Acc:MGI:5011906]"	2.123	9.65E-05
ENSMUSG00000097324	Carmn	cardiac mesoderm enhancer-associated non-coding RNA [Source:MGI Symbol;Acc:MGI:4439832]	2.122	6.31E-04
ENSMUSG00000025422	Agap2	"ArfGAP with GTPase domain, ankyrin repeat and PH domain 2 [Source:MGI Symbol;Acc:MGI:3580016]"	2.122	7.83E-03
ENSMUSG00000060950	Trmt61a	tRNA methyltransferase 61A [Source:MGI Symbol;Acc:MGI:2443487]	2.121	1.17E-03
ENSMUSG00000027864	Ptgfrn	prostaglandin F2 receptor negative regulator [Source:MGI Symbol;Acc:MGI:1277114]	2.121	2.35E-03
ENSMUSG00000001627	Ifrd1	interferon-related developmental regulator 1 [Source:MGI Symbol;Acc:MGI:1316717]	2.120	2.93E-10
ENSMUSG00000019899	Lama2	"laminin, alpha 2 [Source:MGI Symbol;Acc:MGI:99912]"	2.120	5.51E-04
ENSMUSG00000053907	Mat2a	"methionine adenosyltransferase II, alpha [Source:MGI Symbol;Acc:MGI:2443731]"	2.120	7.64E-18
ENSMUSG00000030103	Bhlhe40	"basic helix-loop-helix family, member e40 [Source:MGI Symbol;Acc:MGI:1097714]"	2.119	5.69E-08
ENSMUSG00000030772	Dkk3	dickkopf WNT signaling pathway inhibitor 3 [Source:MGI Symbol;Acc:MGI:1354952]	2.119	1.46E-02
ENSMUSG00000017550	Atad5	"ATPase family, AAA domain containing 5 [Source:MGI Symbol;Acc:MGI:2442925]"	2.119	1.51E-02
ENSMUSG00000038508	Gdf15	growth differentiation factor 15 [Source:MGI Symbol;Acc:MGI:1346047]	2.118	1.26E-02
ENSMUSG00000052572	Dlg2	discs large MAGUK scaffold protein 2 [Source:MGI Symbol;Acc:MGI:1344351]	2.118	1.36E-02

ENSMUSG00000006442	Srm	spermidine synthase [Source:MGI Symbol;Acc:MGI:102690]	2.117	1.94E-06
ENSMUSG00000109093	Gm19950	"predicted gene, 19950 [Source:MGI Symbol;Acc:MGI:5012135]"	2.117	3.70E-08
ENSMUSG00000023393	Slc17a9	"solute carrier family 17, member 9 [Source:MGI Symbol;Acc:MGI:1919107]"	2.116	1.07E-03
ENSMUSG00000020282	Rhbdf1	rhomboid 5 homolog 1 [Source:MGI Symbol;Acc:MGI:104328]	2.116	2.12E-05
ENSMUSG00000056131	Pgm3	phosphoglucomutase 3 [Source:MGI Symbol;Acc:MGI:97566]	2.115	2.93E-09
ENSMUSG00000112307	Gm48751	"predicted gene, 48751 [Source:MGI Symbol;Acc:MGI:6098427]"	2.115	1.41E-02
ENSMUSG00000114045	Gm34220	"predicted gene, 34220 [Source:MGI Symbol;Acc:MGI:5593379]"	2.113	3.18E-03
ENSMUSG00000029299	Abcg3	ATP binding cassette subfamily G member 3 [Source:MGI Symbol;Acc:MGI:1351624]	2.112	5.61E-07
ENSMUSG00000031214	Ophn1	oligophrenin 1 [Source:MGI Symbol;Acc:MGI:2151070]	2.112	2.87E-05
ENSMUSG00000045751	Mms22l	"MMS22-like, DNA repair protein [Source:MGI Symbol;Acc:MGI:2684980]"	2.111	1.85E-02
ENSMUSG00000092021	Gbp11	guanylate binding protein 11 [Source:MGI Symbol;Acc:MGI:3646307]	2.111	7.09E-03
ENSMUSG00000057058	Skap1	src family associated phosphoprotein 1 [Source:MGI Symbol;Acc:MGI:1925723]	2.111	1.27E-02
ENSMUSG00000070436	Serpinh1	"serine (or cysteine) peptidase inhibitor, clade H, member 1 [Source:MGI Symbol;Acc:MGI:88283]"	2.108	2.93E-03
ENSMUSG00000068114	Ccdc134	coiled-coil domain containing 134 [Source:MGI Symbol;Acc:MGI:1923707]	2.108	4.41E-04
ENSMUSG00000031827	Cotl1	coactosin-like 1 (Dictyostelium) [Source:MGI Symbol;Acc:MGI:1919292]	2.105	7.28E-05
ENSMUSG00000004609	Cd33	CD33 antigen [Source:MGI Symbol;Acc:MGI:99440]	2.105	1.86E-03
ENSMUSG00000030095	Tmem43	transmembrane protein 43 [Source:MGI Symbol;Acc:MGI:1921372]	2.102	8.09E-06
ENSMUSG00000029380	Cxcl1	chemokine (C-X-C motif) ligand 1 [Source:MGI Symbol;Acc:MGI:108068]	2.099	1.01E-07
ENSMUSG00000041607	Mbp	myelin basic protein [Source:MGI Symbol;Acc:MGI:96925]	2.098	2.32E-02
ENSMUSG00000007080	Pole	"polymerase (DNA directed), epsilon [Source:MGI Symbol;Acc:MGI:1196391]"	2.098	9.63E-03
ENSMUSG00000097415	AU020206	expressed sequence AU020206 [Source:MGI Symbol;Acc:MGI:2142134]	2.096	2.13E-12
ENSMUSG00000039208	Metrnl	"meteorin, glial cell differentiation regulator-like [Source:MGI Symbol;Acc:MGI:2384806]"	2.096	3.77E-03
ENSMUSG00000044072	Eml6	echinoderm microtubule associated protein like 6 [Source:MGI Symbol;Acc:MGI:2442895]	2.096	3.51E-03
ENSMUSG00000107456	Gm10400	predicted gene 10400 [Source:MGI Symbol;Acc:MGI:3708745]	2.096	1.49E-05
ENSMUSG00000027219	Slc28a2	"solute carrier family 28 (sodium-coupled nucleoside transporter), member 2 [Source:MGI Symbol;Acc:MGI:1913105]"	2.095	1.11E-02
ENSMUSG00000034854	Mfsd12	major facilitator superfamily domain containing 12 [Source:MGI Symbol;Acc:MGI:3604804]	2.094	2.27E-07
ENSMUSG00000051344	Plekhm3	"pleckstrin homology domain containing, family M, member 3 [Source:MGI Symbol;Acc:MGI:2443627]"	2.092	4.84E-09
ENSMUSG00000109325	Krt8-ps	"keratin 8, pseudogene [Source:MGI Symbol;Acc:MGI:3779503]"	2.091	3.12E-03
ENSMUSG00000074863	Platr25	pluripotency associated transcript 25 [Source:MGI Symbol;Acc:MGI:3645613]	2.089	1.75E-03
ENSMUSG00000039158	Akna	AT-hook transcription factor [Source:MGI Symbol;Acc:MGI:2140340]	2.089	1.09E-04
ENSMUSG00000000693	Loxl3	lysyl oxidase-like 3 [Source:MGI Symbol;Acc:MGI:1337004]	2.088	3.65E-02
ENSMUSG00000115381	Gm49266	"predicted gene, 49266 [Source:MGI Symbol;Acc:MGI:6118744]"	2.088	7.09E-05
ENSMUSG00000003355	Fkbp11	FK506 binding protein 11 [Source:MGI Symbol;Acc:MGI:1913370]	2.088	3.62E-04
ENSMUSG00000033705	Stard9	START domain containing 9 [Source:MGI Symbol;Acc:MGI:3045258]	2.088	2.85E-05
ENSMUSG00000024087	Cyp1b1	"cytochrome P450, family 1, subfamily b, polypeptide 1 [Source:MGI Symbol;Acc:MGI:88590]"	2.087	3.12E-02
ENSMUSG00000104391	Gm37960	"predicted gene, 37960 [Source:MGI Symbol;Acc:MGI:5611188]"	2.085	1.78E-03
ENSMUSG00000099413	Gm17767	"predicted gene, 17767 [Source:MGI Symbol;Acc:MGI:5009931]"	2.085	4.54E-03
ENSMUSG00000022306	Zfpm2	"zinc finger protein, multitype 2 [Source:MGI Symbol;Acc:MGI:1334444]"	2.084	1.93E-07
ENSMUSG00000044339	Alkbh2	"alkB homolog 2, alpha-ketoglutarate-dependent dioxygenase [Source:MGI Symbol;Acc:MGI:2141032]"	2.081	4.79E-02
ENSMUSG00000024538	Ppic	peptidylprolyl isomerase C [Source:MGI Symbol;Acc:MGI:97751]	2.081	5.25E-03
ENSMUSG00000029414	Kntc1	kinetochore associated 1 [Source:MGI Symbol;Acc:MGI:2673709]	2.081	2.76E-02
ENSMUSG00000108365	Gm44951	predicted gene 44951 [Source:MGI Symbol;Acc:MGI:5753527]	2.080	8.93E-08
ENSMUSG00000102352	Gm38346	"predicted gene, 38346 [Source:MGI Symbol;Acc:MGI:5611574]"	2.079	1.53E-04
ENSMUSG00000049037	Clec4a1	"C-type lectin domain family 4, member a1 [Source:MGI Symbol;Acc:MGI:3036291]"	2.079	6.76E-03
ENSMUSG00000110142	Gm45872	predicted gene 45872 [Source:MGI Symbol;Acc:MGI:5804987]	2.079	2.20E-02
ENSMUSG00000029910	Mad2l1	MAD2 mitotic arrest deficient-like 1 [Source:MGI Symbol;Acc:MGI:1860374]	2.078	2.53E-04
ENSMUSG00000049382	Krt8	keratin 8 [Source:MGI Symbol;Acc:MGI:96705]	2.077	4.25E-15
ENSMUSG00000024174	Pot1b	protection of telomeres 1B [Source:MGI Symbol;Acc:MGI:1920086]	2.076	2.20E-09
ENSMUSG00000074254	Cyp2a4	"cytochrome P450, family 2, subfamily a, polypeptide 4 [Source:MGI Symbol;Acc:MGI:88596]"	2.075	3.25E-05
ENSMUSG00000094405	Gm23143	"predicted gene, 23143 [Source:MGI Symbol;Acc:MGI:5452920]"	2.074	4.32E-03

ENSMUSG00000043015	Nemp2	nuclear envelope integral membrane protein 2 [Source:MGI Symbol;Acc:MGI:2444113]	2.073	1.75E-03
ENSMUSG00000060183	Cxcl11	chemokine (C-X-C motif) ligand 11 [Source:MGI Symbol;Acc:MGI:1860203]	2.073	2.30E-02
ENSMUSG00000103593	Gm37352	"predicted gene, 37352 [Source:MGI Symbol;Acc:MGI:5610580]"	2.072	2.21E-03
ENSMUSG00000005413	Hmox1	heme oxygenase 1 [Source:MGI Symbol;Acc:MGI:96163]	2.070	7.19E-07
ENSMUSG00000032093	Cd3e	"CD3 antigen, epsilon polypeptide [Source:MGI Symbol;Acc:MGI:88332]"	2.069	1.58E-02
ENSMUSG00000016494	Cd34	CD34 antigen [Source:MGI Symbol;Acc:MGI:88329]	2.069	4.57E-02
ENSMUSG00000102509	Gm37368	"predicted gene, 37368 [Source:MGI Symbol;Acc:MGI:5610596]"	2.068	2.50E-02
ENSMUSG00000028373	Astn2	astrotactin 2 [Source:MGI Symbol;Acc:MGI:1889277]	2.067	7.64E-03
ENSMUSG00000029322	Plac8	placenta-specific 8 [Source:MGI Symbol;Acc:MGI:2445289]	2.067	3.47E-04
ENSMUSG00000110894	Gm48335	"predicted gene, 48335 [Source:MGI Symbol;Acc:MGI:6097794]"	2.065	3.39E-03
ENSMUSG00000031328	Flna	"filamin, alpha [Source:MGI Symbol;Acc:MGI:95556]"	2.064	4.00E-06
ENSMUSG00000055675	Kbtbd11	kelch repeat and BTB (POZ) domain containing 11 [Source:MGI Symbol;Acc:MGI:1922151]	2.063	2.48E-02
ENSMUSG00000044231	Nhlrc1	NHL repeat containing 1 [Source:MGI Symbol;Acc:MGI:2145264]	2.062	1.20E-02
ENSMUSG00000049225	Pdp1	pyruvate dehyrogenase phosphatase catalytic subunit 1 [Source:MGI Symbol;Acc:MGI:2685870]	2.061	2.58E-03
ENSMUSG00000024548	Setbp1	SET binding protein 1 [Source:MGI Symbol;Acc:MGI:1933199]	2.061	1.02E-02
ENSMUSG00000034613	Ppm1h	protein phosphatase 1H (PP2C domain containing) [Source:MGI Symbol;Acc:MGI:2442087]	2.061	6.45E-07
ENSMUSG00000104925	Gm43061	predicted gene 43061 [Source:MGI Symbol;Acc:MGI:5663198]	2.059	4.45E-05
ENSMUSG00000102153	Gm37474	"predicted gene, 37474 [Source:MGI Symbol;Acc:MGI:5610702]"	2.058	1.55E-04
ENSMUSG00000028041	Adam15	a disintegrin and metallopeptidase domain 15 (metargidin) [Source:MGI Symbol;Acc:MGI:1333882]	2.058	7.36E-03
ENSMUSG00000046841	Ckap4	cytoskeleton-associated protein 4 [Source:MGI Symbol;Acc:MGI:2444926]	2.057	2.47E-04
ENSMUSG00000003534	Ddr1	"discoidin domain receptor family, member 1 [Source:MGI Symbol;Acc:MGI:99216]"	2.056	9.64E-04
ENSMUSG00000109873	Gm45407	predicted gene 45407 [Source:MGI Symbol;Acc:MGI:5791243]	2.056	3.64E-04
ENSMUSG00000027639	Samhd1	"SAM domain and HD domain, 1 [Source:MGI Symbol;Acc:MGI:1927468]"	2.055	9.71E-07
ENSMUSG00000032786	Alas1	aminolevulinic acid synthase 1 [Source:MGI Symbol;Acc:MGI:87989]	2.054	1.91E-05
ENSMUSG00000103233	Gm37159	"predicted gene, 37159 [Source:MGI Symbol;Acc:MGI:5610387]"	2.053	7.39E-05
ENSMUSG00000110282	B930086L07Rik	RIKEN cDNA B930086L07 gene [Source:MGI Symbol;Acc:MGI:2443202]	2.053	7.37E-06
ENSMUSG00000022673	Mcm4	minichromosome maintenance complex component 4 [Source:MGI Symbol;Acc:MGI:103199]	2.052	9.11E-05
ENSMUSG00000052384	Nrros	negative regulator of reactive oxygen species [Source:MGI Symbol;Acc:MGI:2445095]	2.052	3.14E-08
ENSMUSG00000059195	Gm12715	predicted gene 12715 [Source:MGI Symbol;Acc:MGI:3650759]	2.052	9.35E-11
ENSMUSG00000028838	Extl1	exostosin-like glycosyltransferase 1 [Source:MGI Symbol;Acc:MGI:1888742]	2.051	3.72E-02
ENSMUSG00000027306	Nusap1	nucleolar and spindle associated protein 1 [Source:MGI Symbol;Acc:MGI:2675669]	2.051	4.30E-02
ENSMUSG00000024063	Lbh	limb-bud and heart [Source:MGI Symbol;Acc:MGI:1925139]	2.050	4.08E-03
ENSMUSG00000064043	Trerf1	transcriptional regulating factor 1 [Source:MGI Symbol;Acc:MGI:2442086]	2.050	1.25E-02
ENSMUSG00000105378	Gm42515	predicted gene 42515 [Source:MGI Symbol;Acc:MGI:5662652]	2.050	1.21E-04
ENSMUSG00000111212	Gm47087	"predicted gene, 47087 [Source:MGI Symbol;Acc:MGI:6095814]"	2.047	3.09E-10
ENSMUSG00000015766	Eps8	epidermal growth factor receptor pathway substrate 8 [Source:MGI Symbol;Acc:MGI:104684]	2.045	2.67E-04
ENSMUSG00000023341	Mx2	MX dynamin-like GTPase 2 [Source:MGI Symbol;Acc:MGI:97244]	2.045	1.40E-03
ENSMUSG00000103772	Gm36933	"predicted gene, 36933 [Source:MGI Symbol;Acc:MGI:5610161]"	2.041	1.60E-05
ENSMUSG00000021196	Pfkp	"phosphofructokinase, platelet [Source:MGI Symbol;Acc:MGI:1891833]"	2.041	3.42E-05
ENSMUSG00000048163	Selplg	"selectin, platelet (p-selectin) ligand [Source:MGI Symbol;Acc:MGI:106689]"	2.040	8.70E-04
ENSMUSG00000059901	Adamts14	"a disintegrin-like and metallopeptidase (reprolysin type) with thrombospondin type 1 motif, 14 [Source:MGI Symbol;Acc:MGI:2179942]"	2.040	1.83E-02
ENSMUSG00000025758	Plk4	polo like kinase 4 [Source:MGI Symbol;Acc:MGI:101783]	2.040	3.11E-03
ENSMUSG00000043300	B3galnt1	"UDP-GalNAc:betaGlcNAc beta 1,3-galactosaminyltransferase, polypeptide 1 [Source:MGI Symbol;Acc:MGI:1349405]"	2.039	1.33E-02
ENSMUSG00000040204	Pclaf	PCNA clamp associated factor [Source:MGI Symbol;Acc:MGI:1915276]	2.039	1.32E-02
ENSMUSG00000049988	Lrrc25	leucine rich repeat containing 25 [Source:MGI Symbol;Acc:MGI:2445284]	2.036	1.30E-02
ENSMUSG00000072677	Sox5it	"SRY (sex determining region Y)-box 5, intronic transcript [Source:MGI Symbol;Acc:MGI:3642586]"	2.036	2.71E-03
ENSMUSG00000040247	Tbc1d10c	"TBC1 domain family, member 10c [Source:MGI Symbol;Acc:MGI:1922072]"	2.035	2.35E-02
ENSMUSG00000108042	B130021K23Rik	RIKEN cDNA B130021K23 gene [Source:MGI Symbol;Acc:MGI:2442518]	2.035	1.58E-03
ENSMUSG00000102714	Gm37618	"predicted gene, 37618 [Source:MGI Symbol;Acc:MGI:5610846]"	2.035	1.10E-03
ENSMUSG00000098934	Gm18853	"predicted gene, 18853 [Source:MGI Symbol;Acc:MGI:5011038]"	2.034	7.86E-03

ENSMUSG00000036381	P2ry14	"purinergic receptor P2Y, G-protein coupled, 14 [Source:MGI Symbol;Acc:MGI:2155705]"	2.034	1.30E-03
ENSMUSG00000070501	Ifi214	interferon activated gene 214 [Source:MGI Symbol;Acc:MGI:3584522]	2.032	4.65E-03
ENSMUSG00000068196	Col8a1	"collagen, type VIII, alpha 1 [Source:MGI Symbol;Acc:MGI:88463]"	2.032	1.93E-02
ENSMUSG00000098112	Bin2	bridging integrator 2 [Source:MGI Symbol;Acc:MGI:3611448]	2.031	4.95E-03
ENSMUSG00000004612	Nkg7	natural killer cell group 7 sequence [Source:MGI Symbol;Acc:MGI:1931250]	2.030	2.91E-02
ENSMUSG00000053158	Fes	feline sarcoma oncogene [Source:MGI Symbol;Acc:MGI:95514]	2.029	2.63E-02
ENSMUSG00000034220	Gpc1	glypican 1 [Source:MGI Symbol;Acc:MGI:1194891]	2.029	4.74E-04
ENSMUSG00000033031	Cip2a	cell proliferation regulating inhibitor of protein phosphatase 2A [Source:MGI Symbol;Acc:MGI:2146335]	2.029	2.17E-02
ENSMUSG00000024053	Emilin2	elastin microfibril interfacer 2 [Source:MGI Symbol;Acc:MGI:2389136]	2.028	1.04E-02
ENSMUSG00000041642	Kif21b	kinesin family member 21B [Source:MGI Symbol;Acc:MGI:109234]	2.028	9.02E-05
ENSMUSG00000025986	Slc39a10	"solute carrier family 39 (zinc transporter), member 10 [Source:MGI Symbol;Acc:MGI:1914515]"	2.027	4.06E-08
ENSMUSG00000037661	Gpr160	G protein-coupled receptor 160 [Source:MGI Symbol;Acc:MGI:1919112]	2.027	4.63E-02
ENSMUSG00000053192	Mllt11	"myeloid/lymphoid or mixed-lineage leukemia; translocated to, 11 [Source:MGI Symbol;Acc:MGI:1929671]"	2.024	2.30E-02
ENSMUSG00000019817	Plagl1	pleiomorphic adenoma gene-like 1 [Source:MGI Symbol;Acc:MGI:1100874]	2.023	7.48E-04
ENSMUSG00000011884	Gltp	glycolipid transfer protein [Source:MGI Symbol;Acc:MGI:1929253]	2.023	1.20E-03
ENSMUSG00000028633	Ctps	cytidine 5'-triphosphate synthase [Source:MGI Symbol;Acc:MGI:1858304]	2.023	3.39E-06
ENSMUSG00000020423	Btg2	BTG anti-proliferation factor 2 [Source:MGI Symbol;Acc:MGI:108384]	2.023	1.50E-07
ENSMUSG00000106024	A530083M17Rik	RIKEN cDNA A530083M17 gene [Source:MGI Symbol;Acc:MGI:2444172]	2.022	1.05E-02
ENSMUSG00000024937	Ehbp1l1	EH domain binding protein 1-like 1 [Source:MGI Symbol;Acc:MGI:3612340]	2.021	1.14E-12
ENSMUSG00000024851	Pitpnm1	"phosphatidylinositol transfer protein, membrane-associated 1 [Source:MGI Symbol;Acc:MGI:1197524]"	2.019	6.54E-05
ENSMUSG00000039985	Sinhcaf	SIN3-HDAC complex associated factor [Source:MGI Symbol;Acc:MGI:1929091]	2.018	1.12E-02
ENSMUSG00000026586	Prrx1	paired related homeobox 1 [Source:MGI Symbol;Acc:MGI:97712]	2.017	1.65E-02
ENSMUSG00000030798	Cd37	CD37 antigen [Source:MGI Symbol;Acc:MGI:88330]	2.015	7.52E-03
ENSMUSG00000042804	Gpr153	G protein-coupled receptor 153 [Source:MGI Symbol;Acc:MGI:1916157]	2.014	8.70E-03
ENSMUSG00000030156	Cd69	CD69 antigen [Source:MGI Symbol;Acc:MGI:88343]	2.014	3.04E-02
ENSMUSG00000097313	Gm26569	"predicted gene, 26569 [Source:MGI Symbol;Acc:MGI:5477063]"	2.007	1.57E-02
ENSMUSG00000026315	Serpinb8	"serine (or cysteine) peptidase inhibitor, clade B, member 8 [Source:MGI Symbol;Acc:MGI:894657]"	2.007	4.97E-04
ENSMUSG00000016918	Sulf1	sulfatase 1 [Source:MGI Symbol;Acc:MGI:2138563]	2.007	1.32E-02
ENSMUSG00000026360	Rgs2	regulator of G-protein signaling 2 [Source:MGI Symbol;Acc:MGI:1098271]	2.005	4.59E-04
ENSMUSG00000058163	Gm5431	predicted gene 5431 [Source:MGI Symbol;Acc:MGI:3645205]	2.004	1.68E-03
ENSMUSG00000099481	Xndc1	Xrcc1 N-terminal domain containing 1 [Source:MGI Symbol;Acc:MGI:5546359]	2.003	1.20E-02
ENSMUSG00000018459	Slc13a3	"solute carrier family 13 (sodium-dependent dicarboxylate transporter), member 3 [Source:MGI Symbol;Acc:MGI:2149635]"	2.001	3.03E-08
ENSMUSG00000030970	Ctbp2	C-terminal binding protein 2 [Source:MGI Symbol;Acc:MGI:1201686]	2.000	1.01E-02
ENSMUSG00000051579	Tceal8	transcription elongation factor A (SII)-like 8 [Source:MGI Symbol;Acc:MGI:1913934]	2.000	9.65E-07
ENSMUSG00000028159	Dapp1	dual adaptor for phosphotyrosine and 3-phosphoinositides 1 [Source:MGI Symbol;Acc:MGI:1347063]	2.000	3.03E-02
ENSMUSG00000025192	Entpd7	ectonucleoside triphosphate diphosphohydrolase 7 [Source:MGI Symbol;Acc:MGI:2135885]	1.999	2.21E-07
ENSMUSG00000086012	Gm15902	predicted gene 15902 [Source:MGI Symbol;Acc:MGI:3802133]	1.999	1.16E-05
ENSMUSG00000018068	Ints2	integrator complex subunit 2 [Source:MGI Symbol;Acc:MGI:1917672]	1.997	6.80E-08
ENSMUSG00000024411	Aqp4	aquaporin 4 [Source:MGI Symbol;Acc:MGI:107387]	1.996	6.52E-05
ENSMUSG00000105766	Gm19666	"predicted gene, 19666 [Source:MGI Symbol;Acc:MGI:5011851]"	1.996	4.22E-02
ENSMUSG00000028961	Pgd	phosphogluconate dehydrogenase [Source:MGI Symbol;Acc:MGI:97553]	1.994	4.39E-07
ENSMUSG00000014599	Csf1	colony stimulating factor 1 (macrophage) [Source:MGI Symbol;Acc:MGI:1339753]	1.991	1.55E-04
ENSMUSG00000038187	Btbd10	BTB (POZ) domain containing 10 [Source:MGI Symbol;Acc:MGI:1916065]	1.991	5.99E-05
ENSMUSG00000111361	Gm47445	"predicted gene, 47445 [Source:MGI Symbol;Acc:MGI:6096399]"	1.990	2.22E-02
ENSMUSG00000072620	Slfn2	schlafen 2 [Source:MGI Symbol;Acc:MGI:1313258]	1.990	6.25E-05
ENSMUSG00000112642	Gm17849	"predicted gene, 17849 [Source:MGI Symbol;Acc:MGI:5010034]"	1.990	1.02E-02
ENSMUSG00000039747	Orai2	ORAI calcium release-activated calcium modulator 2 [Source:MGI Symbol;Acc:MGI:2443195]	1.990	3.66E-03
ENSMUSG00000022831	Hcls1	hematopoietic cell specific Lyn substrate 1 [Source:MGI Symbol;Acc:MGI:104568]	1.989	3.49E-03
ENSMUSG00000073489	Ifi204	interferon activated gene 204 [Source:MGI Symbol;Acc:MGI:96429]	1.989	5.88E-07
ENSMUSG00000057191	AB124611	cDNA sequence AB124611 [Source:MGI Symbol;Acc:MGI:3043001]	1.989	7.05E-03

ENSMUSG00000102336	Gm37233	"predicted gene, 37233 [Source:MGI Symbol;Acc:MGI:5610461]"	1.988	2.49E-02
ENSMUSG00000106472	Gm43111	predicted gene 43111 [Source:MGI Symbol;Acc:MGI:5663248]	1.986	3.77E-02
ENSMUSG00000037341	Slc9a7	"solute carrier family 9 (sodium/hydrogen exchanger), member 7 [Source:MGI Symbol;Acc:MGI:2444530]"	1.986	4.11E-03
ENSMUSG00000001986	Gria3	"glutamate receptor, ionotropic, AMPA3 (alpha 3) [Source:MGI Symbol;Acc:MGI:95810]"	1.985	1.95E-02
ENSMUSG00000021322	Aoah	acyloxyacyl hydrolase [Source:MGI Symbol;Acc:MGI:1350928]	1.985	7.77E-03
ENSMUSG00000028268	Gbp3	guanylate binding protein 3 [Source:MGI Symbol;Acc:MGI:1926263]	1.984	4.88E-04
ENSMUSG00000043895	S1pr2	sphingosine-1-phosphate receptor 2 [Source:MGI Symbol;Acc:MGI:99569]	1.983	3.54E-06
ENSMUSG00000038205	Prkab2	"protein kinase, AMP-activated, beta 2 non-catalytic subunit [Source:MGI Symbol;Acc:MGI:1336185]"	1.983	7.54E-06
ENSMUSG00000045868	Gvin1	"GTPase, very large interferon inducible 1 [Source:MGI Symbol;Acc:MGI:1921808]"	1.983	5.00E-10
ENSMUSG00000014444	Piezo1	piezo-type mechanosensitive ion channel component 1 [Source:MGI Symbol;Acc:MGI:3603204]	1.977	2.03E-04
ENSMUSG00000020272	Stk10	serine/threonine kinase 10 [Source:MGI Symbol;Acc:MGI:1099439]	1.977	1.65E-04
ENSMUSG00000015312	Gadd45b	growth arrest and DNA-damage-inducible 45 beta [Source:MGI Symbol;Acc:MGI:107776]	1.976	8.61E-03
ENSMUSG00000022014	Epsti1	epithelial stromal interaction 1 (breast) [Source:MGI Symbol;Acc:MGI:1915168]	1.975	1.81E-02
ENSMUSG00000054752	Fsd1l	fibronectin type III and SPRY domain containing 1-like [Source:MGI Symbol;Acc:MGI:2442443]	1.974	3.08E-02
ENSMUSG00000060487	Samd5	sterile alpha motif domain containing 5 [Source:MGI Symbol;Acc:MGI:2444815]	1.974	2.20E-02
ENSMUSG00000111505	Gm48673	"predicted gene, 48673 [Source:MGI Symbol;Acc:MGI:6098292]"	1.973	7.79E-03
ENSMUSG00000098557	Kctd12	potassium channel tetramerisation domain containing 12 [Source:MGI Symbol;Acc:MGI:2145823]	1.973	1.66E-07
ENSMUSG00000096916	Zfp850	zinc finger protein 850 [Source:MGI Symbol;Acc:MGI:3036281]	1.972	2.46E-02
ENSMUSG00000020241	Col6a2	"collagen, type VI, alpha 2 [Source:MGI Symbol;Acc:MGI:88460]"	1.972	7.41E-03
ENSMUSG00000036246	Gmip	Gem-interacting protein [Source:MGI Symbol;Acc:MGI:1926066]	1.970	6.65E-04
ENSMUSG00000001750	Tcirg1	"T cell, immune regulator 1, ATPase, H+ transporting, lysosomal V0 protein A3 [Source:MGI Symbol;Acc:MGI:1350931]"	1.969	1.03E-03
ENSMUSG00000041219	Arhgap11a	Rho GTPase activating protein 11A [Source:MGI Symbol;Acc:MGI:2444300]	1.968	1.72E-04
ENSMUSG00000040268	Plekha1	"pleckstrin homology domain containing, family A (phosphoinositide binding specific) member 1 [Source:MGI Symbol;Acc:MGI:2442213]"	1.968	4.47E-06
ENSMUSG00000024885	Aldh3b1	"aldehyde dehydrogenase 3 family, member B1 [Source:MGI Symbol;Acc:MGI:1914939]"	1.967	2.13E-02
ENSMUSG00000078606	Gm4070	predicted gene 4070 [Source:MGI Symbol;Acc:MGI:3782245]	1.967	3.43E-09
ENSMUSG00000044702	Palb2	partner and localizer of BRCA2 [Source:MGI Symbol;Acc:MGI:3040695]	1.967	1.99E-02
ENSMUSG00000107448	Gm43947	"predicted gene, 43947 [Source:MGI Symbol;Acc:MGI:5690339]"	1.966	8.34E-03
ENSMUSG00000025268	Maged2	"melanoma antigen, family D, 2 [Source:MGI Symbol;Acc:MGI:1933391]"	1.965	2.98E-02
ENSMUSG00000026657	Frmd4a	FERM domain containing 4A [Source:MGI Symbol;Acc:MGI:1919850]	1.964	3.02E-04
ENSMUSG00000102145	Gm38056	"predicted gene, 38056 [Source:MGI Symbol;Acc:MGI:5611284]"	1.964	1.28E-05
ENSMUSG00000042249	Grk3	G protein-coupled receptor kinase 3 [Source:MGI Symbol;Acc:MGI:87941]	1.963	5.36E-07
ENSMUSG00000105071	Gm43336	predicted gene 43336 [Source:MGI Symbol;Acc:MGI:5663473]	1.962	5.83E-05
ENSMUSG00000105288	Gm25820	"predicted gene, 25820 [Source:MGI Symbol;Acc:MGI:5455597]"	1.961	3.52E-02
ENSMUSG00000030793	Pycard	PYD and CARD domain containing [Source:MGI Symbol;Acc:MGI:1931465]	1.960	2.70E-03
ENSMUSG00000043556	Fbxl7	F-box and leucine-rich repeat protein 7 [Source:MGI Symbol;Acc:MGI:3052506]	1.954	8.80E-04
ENSMUSG00000031101	Sash3	SAM and SH3 domain containing 3 [Source:MGI Symbol;Acc:MGI:1921381]	1.954	1.96E-02
ENSMUSG00000028339	Col15a1	"collagen, type XV, alpha 1 [Source:MGI Symbol;Acc:MGI:88449]"	1.953	2.23E-04
ENSMUSG00000022218	Tgm1	"transglutaminase 1, K polypeptide [Source:MGI Symbol;Acc:MGI:98730]"	1.953	7.39E-04
ENSMUSG00000023015	Racgap1	Rac GTPase-activating protein 1 [Source:MGI Symbol;Acc:MGI:1349423]	1.953	3.17E-02
ENSMUSG00000024059	Clip4	"CAP-GLY domain containing linker protein family, member 4 [Source:MGI Symbol;Acc:MGI:1919100]"	1.952	2.03E-02
ENSMUSG00000110405	Gm45534	predicted gene 45534 [Source:MGI Symbol;Acc:MGI:5791370]	1.952	1.60E-02
ENSMUSG00000039202	Abhd2	abhydrolase domain containing 2 [Source:MGI Symbol;Acc:MGI:1914344]	1.951	6.06E-22
ENSMUSG00000103497	Gm37407	"predicted gene, 37407 [Source:MGI Symbol;Acc:MGI:5610635]"	1.951	1.57E-03
ENSMUSG00000097431	Gm26782	"predicted gene, 26782 [Source:MGI Symbol;Acc:MGI:5477276]"	1.951	1.89E-02
ENSMUSG00000105985	Gm42993	predicted gene 42993 [Source:MGI Symbol;Acc:MGI:5663130]	1.950	5.24E-04
ENSMUSG00000022816	Fstl1	follistatin-like 1 [Source:MGI Symbol;Acc:MGI:102793]	1.949	1.01E-03
ENSMUSG00000103284	3110080O07Rik	RIKEN cDNA 3110080O07 gene [Source:MGI Symbol;Acc:MGI:1920491]	1.948	2.52E-02
ENSMUSG00000075284	Wipf1	"WAS/WASL interacting protein family, member 1 [Source:MGI Symbol;Acc:MGI:2178801]"	1.947	1.40E-03
ENSMUSG00000056665	Them6	thioesterase superfamily member 6 [Source:MGI Symbol;Acc:MGI:1925301]	1.947	3.21E-02
ENSMUSG00000104253	Gm37174	"predicted gene, 37174 [Source:MGI Symbol;Acc:MGI:5610402]"	1.947	4.64E-03

ENSMUSG00000113557	Gm47657	"predicted gene, 47657 [Source:MGI Symbol;Acc:MGI:6096742]"	1.946	9.24E-03
ENSMUSG00000040659	Efhd2	EF hand domain containing 2 [Source:MGI Symbol;Acc:MGI:106504]	1.946	2.64E-06
ENSMUSG00000104524	Gm37333	"predicted gene, 37333 [Source:MGI Symbol;Acc:MGI:5610561]"	1.946	4.29E-02
ENSMUSG00000061758	Akr1b10	"aldo-keto reductase family 1, member B10 (aldose reductase) [Source:MGI Symbol;Acc:MGI:1915111]"	1.945	4.41E-02
ENSMUSG00000037405	Icam1	intercellular adhesion molecule 1 [Source:MGI Symbol;Acc:MGI:96392]	1.945	1.93E-09
ENSMUSG00000056025	Clca3a1	chloride channel accessory 3A1 [Source:MGI Symbol;Acc:MGI:1316732]	1.945	5.81E-04
ENSMUSG00000027068	Dhrs9	dehydrogenase/reductase (SDR family) member 9 [Source:MGI Symbol;Acc:MGI:2442798]	1.944	5.03E-04
ENSMUSG00000036273	Lrrk2	leucine-rich repeat kinase 2 [Source:MGI Symbol;Acc:MGI:1913975]	1.942	1.25E-04
ENSMUSG00000014303	Glis2	GLIS family zinc finger 2 [Source:MGI Symbol;Acc:MGI:1932535]	1.942	2.64E-02
ENSMUSG00000021597	Slf1	SMC5-SMC6 complex localization factor 1 [Source:MGI Symbol;Acc:MGI:2145448]	1.941	1.00E-02
ENSMUSG00000028214	Gem	GTP binding protein (gene overexpressed in skeletal muscle) [Source:MGI Symbol;Acc:MGI:99844]	1.941	1.69E-02
ENSMUSG00000031133	Arhgef6	Rac/Cdc42 guanine nucleotide exchange factor (GEF) 6 [Source:MGI Symbol;Acc:MGI:1920591]	1.941	3.94E-03
ENSMUSG00000037685	Atp8a1	"ATPase, aminophospholipid transporter (APLT), class I, type 8A, member 1 [Source:MGI Symbol;Acc:MGI:1330848]"	1.940	9.78E-05
ENSMUSG00000022305	Lrp12	low density lipoprotein-related protein 12 [Source:MGI Symbol;Acc:MGI:2443132]	1.940	9.87E-04
ENSMUSG00000095676	Gm25099	"predicted gene, 25099 [Source:MGI Symbol;Acc:MGI:5454876]"	1.936	5.42E-04
ENSMUSG00000105553	Gm42514	predicted gene 42514 [Source:MGI Symbol;Acc:MGI:5662651]	1.935	4.61E-02
ENSMUSG00000021556	Golm1	golgi membrane protein 1 [Source:MGI Symbol;Acc:MGI:1917329]	1.935	6.37E-03
ENSMUSG00000103694	Gm37530	"predicted gene, 37530 [Source:MGI Symbol;Acc:MGI:5610758]"	1.933	2.74E-02
ENSMUSG00000117333	Gm16386	predicted gene 16386 [Source:MGI Symbol;Acc:MGI:3646848]	1.933	2.04E-04
ENSMUSG00000053541	Gm4759	predicted gene 4759 [Source:MGI Symbol;Acc:MGI:3647753]	1.932	3.86E-02
ENSMUSG00000051498	Tlr6	toll-like receptor 6 [Source:MGI Symbol;Acc:MGI:1341296]	1.929	3.73E-02
ENSMUSG00000032449	Slc25a36	"solute carrier family 25, member 36 [Source:MGI Symbol;Acc:MGI:1924909]"	1.929	1.54E-07
ENSMUSG00000043542	Zc2hc1a	"zinc finger, C2HC-type containing 1A [Source:MGI Symbol;Acc:MGI:1914556]"	1.928	9.97E-03
ENSMUSG00000093458	Gm20688	predicted gene 20688 [Source:MGI Symbol;Acc:MGI:5313135]	1.928	3.88E-02
ENSMUSG00000027293	Ehd4	EH-domain containing 4 [Source:MGI Symbol;Acc:MGI:1919619]	1.928	5.20E-03
ENSMUSG00000105083	Gm42699	predicted gene 42699 [Source:MGI Symbol;Acc:MGI:5662836]	1.928	1.79E-03
ENSMUSG00000027030	Stk39	serine/threonine kinase 39 [Source:MGI Symbol;Acc:MGI:1858416]	1.927	2.63E-03
ENSMUSG00000067916	Zfp991	zinc finger protein 991 [Source:MGI Symbol;Acc:MGI:3701604]	1.926	1.10E-03
ENSMUSG00000053175	Bcl3	B cell leukemia/lymphoma 3 [Source:MGI Symbol;Acc:MGI:88140]	1.926	4.47E-05
ENSMUSG00000027204	Fbn1	fibrillin 1 [Source:MGI Symbol;Acc:MGI:95489]	1.925	1.70E-04
ENSMUSG00000035891	Cerk	ceramide kinase [Source:MGI Symbol;Acc:MGI:2386052]	1.924	1.56E-04
ENSMUSG00000026784	Pdss1	"prenyl (solanesyl) diphosphate synthase, subunit 1 [Source:MGI Symbol;Acc:MGI:1889278]"	1.924	2.38E-02
ENSMUSG00000105748	Gm43088	predicted gene 43088 [Source:MGI Symbol;Acc:MGI:5663225]	1.924	4.65E-03
ENSMUSG00000044350	Lacc1	laccase domain containing 1 [Source:MGI Symbol;Acc:MGI:2445077]	1.923	1.32E-08
ENSMUSG00000102275	Gm37144	"predicted gene, 37144 [Source:MGI Symbol;Acc:MGI:5610372]"	1.923	3.04E-02
ENSMUSG00000099241	Gm18852	"predicted gene, 18852 [Source:MGI Symbol;Acc:MGI:5011037]"	1.923	1.59E-02
ENSMUSG00000086429	Gt(ROSA)26Sor	"gene trap ROSA 26, Philippe Soriano [Source:MGI Symbol;Acc:MGI:104735]"	1.922	1.69E-05
ENSMUSG00000022180	Slc7a8	"solute carrier family 7 (cationic amino acid transporter, y+ system), member 8 [Source:MGI Symbol;Acc:MGI:1355323]"	1.922	3.53E-05
ENSMUSG00000026605	Cenpf	centromere protein F [Source:MGI Symbol;Acc:MGI:1313302]	1.922	2.91E-04
ENSMUSG00000081651	Gm15530	predicted gene 15530 [Source:MGI Symbol;Acc:MGI:3782978]	1.921	1.57E-02
ENSMUSG00000074240	Cib3	calcium and integrin binding family member 3 [Source:MGI Symbol;Acc:MGI:2685953]	1.921	2.22E-03
ENSMUSG00000082570	Gm15711	predicted gene 15711 [Source:MGI Symbol;Acc:MGI:3783153]	1.920	2.14E-02
ENSMUSG00000078945	Naip2	"NLR family, apoptosis inhibitory protein 2 [Source:MGI Symbol;Acc:MGI:1298226]"	1.920	3.25E-06
ENSMUSG00000086920	Gm12207	predicted gene 12207 [Source:MGI Symbol;Acc:MGI:3702399]	1.918	3.77E-02
ENSMUSG00000034255	Arhgap27	Rho GTPase activating protein 27 [Source:MGI Symbol;Acc:MGI:1916903]	1.917	8.15E-03
ENSMUSG00000024235	Map3k8	mitogen-activated protein kinase kinase kinase 8 [Source:MGI Symbol;Acc:MGI:1346878]	1.917	4.82E-05
ENSMUSG00000052889	Prkcb	"protein kinase C, beta [Source:MGI Symbol;Acc:MGI:97596]"	1.917	9.03E-04
ENSMUSG00000106706	C530043K16Rik	RIKEN cDNA C530043K16 gene [Source:MGI Symbol;Acc:MGI:2444587]	1.917	2.71E-04
ENSMUSG00000002486	Tchp	"trichoplein, keratin filament binding [Source:MGI Symbol;Acc:MGI:1925082]"	1.916	3.90E-02
ENSMUSG00000025212	Sfxn3	sideroflexin 3 [Source:MGI Symbol;Acc:MGI:2137679]	1.915	2.32E-02

ENSMUSG00000082292	Gm12250	predicted gene 12250 [Source:MGI Symbol;Acc:MGI:3649299]	1.915	4.56E-02
ENSMUSG00000052160	Pld4	"phospholipase D family, member 4 [Source:MGI Symbol;Acc:MGI:2144765]"	1.912	1.55E-04
ENSMUSG00000016496	Cd274	CD274 antigen [Source:MGI Symbol;Acc:MGI:1926446]	1.912	2.37E-05
ENSMUSG00000005054	Cstb	cystatin B [Source:MGI Symbol;Acc:MGI:109514]	1.911	1.87E-06
ENSMUSG00000062488	Ifit3b	interferon-induced protein with tetratricopeptide repeats 3B [Source:MGI Symbol;Acc:MGI:3698419]	1.910	2.01E-04
ENSMUSG00000023805	Synj2	synaptojanin 2 [Source:MGI Symbol;Acc:MGI:1201671]	1.909	2.50E-04
ENSMUSG00000002602	Axl	AXL receptor tyrosine kinase [Source:MGI Symbol;Acc:MGI:1347244]	1.908	4.36E-05
ENSMUSG00000042331	Specc1	sperm antigen with calponin homology and coiled-coil domains 1 [Source:MGI Symbol;Acc:MGI:2442356]	1.908	2.41E-03
ENSMUSG00000058006	Mdn1	midasin AAA ATPase 1 [Source:MGI Symbol;Acc:MGI:1926159]	1.908	1.15E-04
ENSMUSG00000029553	Tfec	transcription factor EC [Source:MGI Symbol;Acc:MGI:1333760]	1.908	1.91E-03
ENSMUSG00000032366	Tpm1	"tropomyosin 1, alpha [Source:MGI Symbol;Acc:MGI:98809]"	1.907	1.87E-06
ENSMUSG00000029468	P2rx7	"purinergic receptor P2X, ligand-gated ion channel, 7 [Source:MGI Symbol;Acc:MGI:1339957]"	1.906	3.12E-03
ENSMUSG00000039126	Prune2	prune homolog 2 [Source:MGI Symbol;Acc:MGI:1925004]	1.906	4.93E-02
ENSMUSG00000091867	Cyp2a22	"cytochrome P450, family 2, subfamily a, polypeptide 22 [Source:MGI Symbol;Acc:MGI:3648316]"	1.906	1.20E-03
ENSMUSG00000031864	Ints10	integrator complex subunit 10 [Source:MGI Symbol;Acc:MGI:1918135]	1.905	4.20E-03
ENSMUSG00000012519	Mlkl	mixed lineage kinase domain-like [Source:MGI Symbol;Acc:MGI:1921818]	1.904	1.19E-04
ENSMUSG00000041836	Ptpre	"protein tyrosine phosphatase, receptor type, E [Source:MGI Symbol;Acc:MGI:97813]"	1.904	6.16E-05
ENSMUSG00000004698	Hdac9	histone deacetylase 9 [Source:MGI Symbol;Acc:MGI:1931221]	1.903	5.42E-05
ENSMUSG00000073490	Ifi207	interferon activated gene 207 [Source:MGI Symbol;Acc:MGI:2138302]	1.902	2.52E-05
ENSMUSG00000102698	Gm37777	"predicted gene, 37777 [Source:MGI Symbol;Acc:MGI:5611005]"	1.902	4.14E-02
ENSMUSG00000039959	Hip1	huntingtin interacting protein 1 [Source:MGI Symbol;Acc:MGI:1099804]	1.902	2.44E-05
ENSMUSG00000057596	Trim30d	tripartite motif-containing 30D [Source:MGI Symbol;Acc:MGI:3035181]	1.901	2.34E-06
ENSMUSG00000027459	Fam110a	"family with sequence similarity 110, member A [Source:MGI Symbol;Acc:MGI:1921097]"	1.900	1.97E-02
ENSMUSG00000086370	Ftx	"Ftx transcript, Xist regulator (non-protein coding) [Source:MGI Symbol;Acc:MGI:1926128]"	1.900	4.02E-06
ENSMUSG00000091649	Phf11b	PHD finger protein 11B [Source:MGI Symbol;Acc:MGI:3645789]	1.897	4.16E-02
ENSMUSG00000104795	Gm42783	predicted gene 42783 [Source:MGI Symbol;Acc:MGI:5662920]	1.897	3.87E-02
ENSMUSG00000032436	Cmtm7	CKLF-like MARVEL transmembrane domain containing 7 [Source:MGI Symbol;Acc:MGI:2447166]	1.895	2.00E-02
ENSMUSG00000042842	Serpinb6b	"serine (or cysteine) peptidase inhibitor, clade B, member 6b [Source:MGI Symbol;Acc:MGI:894688]"	1.895	2.61E-02
ENSMUSG00000116677	Gm49569	"predicted gene, 49569 [Source:MGI Symbol;Acc:MGI:6214960]"	1.894	3.94E-02
ENSMUSG00000030393	Zik1	zinc finger protein interacting with K protein 1 [Source:MGI Symbol;Acc:MGI:108070]	1.893	3.25E-02
ENSMUSG00000042745	Id1	"inhibitor of DNA binding 1, HLH protein [Source:MGI Symbol;Acc:MGI:96396]"	1.892	7.60E-03
ENSMUSG00000033676	Gabrb3	"gamma-aminobutyric acid (GABA) A receptor, subunit beta 3 [Source:MGI Symbol;Acc:MGI:95621]"	1.891	2.08E-02
ENSMUSG00000025321	Itgb8	integrin beta 8 [Source:MGI Symbol;Acc:MGI:1338035]	1.890	4.10E-02
ENSMUSG00000032334	Loxl1	lysyl oxidase-like 1 [Source:MGI Symbol;Acc:MGI:106096]	1.890	4.72E-02
ENSMUSG00000090582	Gm17024	predicted gene 17024 [Source:MGI Symbol;Acc:MGI:4937851]	1.890	3.94E-05
ENSMUSG00000001642	Akr1b3	"aldo-keto reductase family 1, member B3 (aldose reductase) [Source:MGI Symbol;Acc:MGI:1353494]"	1.889	1.66E-03
ENSMUSG00000102863	Gm37639	"predicted gene, 37639 [Source:MGI Symbol;Acc:MGI:5610867]"	1.889	3.46E-03
ENSMUSG00000064941	Gm23238	"predicted gene, 23238 [Source:MGI Symbol;Acc:MGI:5453015]"	1.888	1.49E-06
ENSMUSG00000103634	3110062G12Rik	RIKEN cDNA 3110062G12 gene [Source:MGI Symbol;Acc:MGI:1920438]	1.888	1.25E-02
ENSMUSG00000104687	Gm42899	predicted gene 42899 [Source:MGI Symbol;Acc:MGI:5663036]	1.885	7.85E-04
ENSMUSG00000042787	Exog	"endo/exonuclease (5'-3'), endonuclease G-like [Source:MGI Symbol;Acc:MGI:2143333]"	1.884	1.34E-02
ENSMUSG00000015947	Fcgr1	"Fc receptor, IgG, high affinity I [Source:MGI Symbol;Acc:MGI:95498]"	1.884	4.49E-03
ENSMUSG00000104724	Gm43162	predicted gene 43162 [Source:MGI Symbol;Acc:MGI:5663299]	1.883	5.65E-03
ENSMUSG00000115647	Gm49265	"predicted gene, 49265 [Source:MGI Symbol;Acc:MGI:6118742]"	1.883	1.01E-02
ENSMUSG00000028312	Smc2	structural maintenance of chromosomes 2 [Source:MGI Symbol;Acc:MGI:106067]	1.883	2.92E-03
ENSMUSG00000042129	Rassf4	Ras association (RalGDS/AF-6) domain family member 4 [Source:MGI Symbol;Acc:MGI:2386853]	1.882	1.82E-07
ENSMUSG00000039157	Fam102a	"family with sequence similarity 102, member A [Source:MGI Symbol;Acc:MGI:2138935]"	1.881	7.76E-08
ENSMUSG00000104052	Gm38125	"predicted gene, 38125 [Source:MGI Symbol;Acc:MGI:5611353]"	1.880	2.71E-04
ENSMUSG00000003849	Nqo1	"NAD(P)H dehydrogenase, quinone 1 [Source:MGI Symbol;Acc:MGI:103187]"	1.878	4.74E-04
ENSMUSG00000039646	Vasn	vasorin [Source:MGI Symbol;Acc:MGI:2177651]	1.876	5.81E-03

ENSMUSG00000037731	Themis2	thymocyte selection associated family member 2 [Source:MGI Symbol;Acc:MGI:2446213]	1.876	3.56E-04
ENSMUSG00000015133	Lrrk1	leucine-rich repeat kinase 1 [Source:MGI Symbol;Acc:MGI:2142227]	1.876	6.78E-04
ENSMUSG00000063286	Gm8995	predicted gene 8995 [Source:MGI Symbol;Acc:MGI:3644223]	1.874	2.94E-12
ENSMUSG00000026837	Col5a1	"collagen, type V, alpha 1 [Source:MGI Symbol;Acc:MGI:88457]"	1.871	5.59E-03
ENSMUSG00000044229	Nxpe4	"neurexophilin and PC-esterase domain family, member 4 [Source:MGI Symbol;Acc:MGI:1924792]"	1.871	2.26E-02
ENSMUSG00000093594	Gm20707	predicted gene 20707 [Source:MGI Symbol;Acc:MGI:5313154]	1.870	1.23E-03
ENSMUSG00000117775	Gm50462	"predicted gene, 50462 [Source:MGI Symbol;Acc:MGI:6324728]"	1.870	2.85E-03
ENSMUSG00000031271	Serpina7	"serine (or cysteine) peptidase inhibitor, clade A (alpha-1 antiproteinase, antitrypsin), member 7 [Source:MGI Symbol;Acc:MGI:3041197]"	1.868	2.39E-04
ENSMUSG00000074896	Ifit3	interferon-induced protein with tetratricopeptide repeats 3 [Source:MGI Symbol;Acc:MGI:1101055]	1.867	2.44E-06
ENSMUSG00000108067	Gm43953	"predicted gene, 43953 [Source:MGI Symbol;Acc:MGI:5690345]"	1.866	2.00E-03
ENSMUSG00000089746	Gm3513	predicted gene 3513 [Source:MGI Symbol;Acc:MGI:3781690]	1.866	1.46E-02
ENSMUSG00000006585	Cdt1	chromatin licensing and DNA replication factor 1 [Source:MGI Symbol;Acc:MGI:1914427]	1.866	3.08E-02
ENSMUSG00000059824	Dbp	D site albumin promoter binding protein [Source:MGI Symbol;Acc:MGI:94866]	1.864	9.44E-05
ENSMUSG00000040613	Apobec1	"apolipoprotein B mRNA editing enzyme, catalytic polypeptide 1 [Source:MGI Symbol;Acc:MGI:103298]"	1.864	6.97E-08
ENSMUSG00000030220	Arhgdib	"Rho, GDP dissociation inhibitor (GDI) beta [Source:MGI Symbol;Acc:MGI:101940]"	1.863	2.95E-03
ENSMUSG00000018920	Cxcl16	chemokine (C-X-C motif) ligand 16 [Source:MGI Symbol;Acc:MGI:1932682]	1.862	4.20E-03
ENSMUSG00000034330	Plcg2	"phospholipase C, gamma 2 [Source:MGI Symbol;Acc:MGI:97616]"	1.862	7.46E-04
ENSMUSG00000048120	Entpd1	ectonucleoside triphosphate diphosphohydrolase 1 [Source:MGI Symbol;Acc:MGI:102805]	1.861	3.03E-02
ENSMUSG00000040322	Slc25a24	"solute carrier family 25 (mitochondrial carrier, phosphate carrier), member 24 [Source:MGI Symbol;Acc:MGI:1917160]"	1.861	1.19E-02
ENSMUSG00000045136	Tubb2b	"tubulin, beta 2B class IIB [Source:MGI Symbol;Acc:MGI:1920960]"	1.861	1.84E-03
ENSMUSG00000030978	Rrm1	ribonucleotide reductase M1 [Source:MGI Symbol;Acc:MGI:98180]	1.860	2.97E-04
ENSMUSG00000105457	Gm43200	predicted gene 43200 [Source:MGI Symbol;Acc:MGI:5663337]	1.859	4.59E-04
ENSMUSG00000103780	Gm37524	"predicted gene, 37524 [Source:MGI Symbol;Acc:MGI:5610752]"	1.857	9.82E-05
ENSMUSG00000021665	Hexb	hexosaminidase B [Source:MGI Symbol;Acc:MGI:96074]	1.857	5.72E-04
ENSMUSG00000042622	Maff	"v-maf musculoaponeurotic fibrosarcoma oncogene family, protein F (avian) [Source:MGI Symbol;Acc:MGI:96910]"	1.857	2.85E-02
ENSMUSG00000019088	Dnase1l1	deoxyribonuclease 1-like 1 [Source:MGI Symbol;Acc:MGI:109628]	1.857	1.16E-03
ENSMUSG00000037318	Traf3ip3	TRAF3 interacting protein 3 [Source:MGI Symbol;Acc:MGI:2441706]	1.855	3.62E-04
ENSMUSG00000106092	Gm43072	predicted gene 43072 [Source:MGI Symbol;Acc:MGI:5663209]	1.854	3.62E-06
ENSMUSG00000078349	AW011738	expressed sequence AW011738 [Source:MGI Symbol;Acc:MGI:2140540]	1.854	3.91E-05
ENSMUSG00000024542	Cep192	centrosomal protein 192 [Source:MGI Symbol;Acc:MGI:1918049]	1.852	3.67E-05
ENSMUSG00000063146	Clip2	CAP-GLY domain containing linker protein 2 [Source:MGI Symbol;Acc:MGI:1313136]	1.851	5.08E-03
ENSMUSG00000103151	Gm38292	"predicted gene, 38292 [Source:MGI Symbol;Acc:MGI:5611520]"	1.851	2.62E-03
ENSMUSG00000079017	Ifi27l2a	"interferon, alpha-inducible protein 27 like 2A [Source:MGI Symbol;Acc:MGI:1924183]"	1.850	4.05E-03
ENSMUSG00000110986	Gm20276	"predicted gene, 20276 [Source:MGI Symbol;Acc:MGI:5012461]"	1.848	1.06E-02
ENSMUSG00000104528	Gm43314	predicted gene 43314 [Source:MGI Symbol;Acc:MGI:5663451]	1.848	9.15E-05
ENSMUSG00000066892	Fbxl12	F-box and leucine-rich repeat protein 12 [Source:MGI Symbol;Acc:MGI:1354738]	1.848	4.11E-02
ENSMUSG00000027624	Epb41l1	erythrocyte membrane protein band 4.1 like 1 [Source:MGI Symbol;Acc:MGI:103010]	1.847	6.74E-03
ENSMUSG00000078851	H2aw	H2A.W histone [Source:MGI Symbol;Acc:MGI:2448458]	1.847	2.10E-02
ENSMUSG00000037907	Ankrd13b	ankyrin repeat domain 13b [Source:MGI Symbol;Acc:MGI:2144501]	1.845	2.87E-02
ENSMUSG00000117628	Gm50012	"predicted gene, 50012 [Source:MGI Symbol;Acc:MGI:6275301]"	1.843	5.32E-03
ENSMUSG00000078546	Zfp995	zinc finger protein 995 [Source:MGI Symbol;Acc:MGI:1917331]	1.842	9.55E-03
ENSMUSG00000107567	4930480K02Rik	RIKEN cDNA 4930480K02 gene [Source:MGI Symbol;Acc:MGI:1922185]	1.841	4.66E-02
ENSMUSG00000022146	Osmr	oncostatin M receptor [Source:MGI Symbol;Acc:MGI:1330819]	1.841	2.30E-04
ENSMUSG00000074415	Mir100hg	Mir100 Mirlet7a-2 Mir125b-1 cluster host gene [Source:MGI Symbol;Acc:MGI:1920394]	1.841	1.13E-08
ENSMUSG00000016495	Plgrkt	"plasminogen receptor, C-terminal lysine transmembrane protein [Source:MGI Symbol;Acc:MGI:1915009]"	1.841	1.93E-03
ENSMUSG00000020101	Vsir	V-set immunoregulatory receptor [Source:MGI Symbol;Acc:MGI:1921298]	1.839	1.46E-05
ENSMUSG00000045932	Ifit2	interferon-induced protein with tetratricopeptide repeats 2 [Source:MGI Symbol;Acc:MGI:99449]	1.838	2.34E-05
ENSMUSG00000033355	Rtp4	receptor transporter protein 4 [Source:MGI Symbol;Acc:MGI:1915025]	1.838	7.81E-04
ENSMUSG00000009013	Dynll1	dynein light chain LC8-type 1 [Source:MGI Symbol;Acc:MGI:1861457]	1.837	1.05E-04
ENSMUSG00000039997	Ifi203	interferon activated gene 203 [Source:MGI Symbol;Acc:MGI:96428]	1.837	4.06E-08

ENSMUSG00000050379	Septin6	septin 6 [Source:MGI Symbol;Acc:MGI:1888939]	1.836	1.28E-02
ENSMUSG00000036473	Tbc1d24	"TBC1 domain family, member 24 [Source:MGI Symbol;Acc:MGI:2443456]"	1.836	1.88E-04
ENSMUSG00000102059	Gm20257	"predicted gene, 20257 [Source:MGI Symbol;Acc:MGI:5012442]"	1.835	4.44E-02
ENSMUSG00000046402	Rbp1	"retinol binding protein 1, cellular [Source:MGI Symbol;Acc:MGI:97876]"	1.835	8.46E-03
ENSMUSG00000027660	Skil	SKI-like [Source:MGI Symbol;Acc:MGI:106203]	1.835	4.26E-07
ENSMUSG00000030830	Itgal	integrin alpha L [Source:MGI Symbol;Acc:MGI:96606]	1.834	2.43E-07
ENSMUSG00000023505	Cdca3	cell division cycle associated 3 [Source:MGI Symbol;Acc:MGI:1315198]	1.834	3.16E-02
ENSMUSG00000013629	Cad	"carbamoyl-phosphate synthetase 2, aspartate transcarbamylase, and dihydroorotase [Source:MGI Symbol;Acc:MGI:1916969]"	1.833	3.02E-03
ENSMUSG00000104761	Gm43511	predicted gene 43511 [Source:MGI Symbol;Acc:MGI:5663648]	1.833	5.31E-03
ENSMUSG00000027959	Sass6	SAS-6 centriolar assembly protein [Source:MGI Symbol;Acc:MGI:1920026]	1.833	1.77E-02
ENSMUSG00000028599	Tnfrsf1b	"tumor necrosis factor receptor superfamily, member 1b [Source:MGI Symbol;Acc:MGI:1314883]"	1.833	3.65E-10
ENSMUSG00000111345	Gm48562	"predicted gene, 48562 [Source:MGI Symbol;Acc:MGI:6098119]"	1.832	6.87E-04
ENSMUSG00000103818	Gm38009	"predicted gene, 38009 [Source:MGI Symbol;Acc:MGI:5611237]"	1.832	4.09E-03
ENSMUSG00000093661	Eif4e3	eukaryotic translation initiation factor 4E member 3 [Source:MGI Symbol;Acc:MGI:1914142]	1.830	2.21E-03
ENSMUSG00000028933	Xrcc2	X-ray repair complementing defective repair in Chinese hamster cells 2 [Source:MGI Symbol;Acc:MGI:1927345]	1.829	1.77E-03
ENSMUSG00000090272	Mndal	myeloid nuclear differentiation antigen like [Source:MGI Symbol;Acc:MGI:3780953]	1.829	1.14E-06
ENSMUSG00000017491	Rarb	"retinoic acid receptor, beta [Source:MGI Symbol;Acc:MGI:97857]"	1.829	2.02E-02
ENSMUSG00000030263	Lrmp	lymphoid-restricted membrane protein [Source:MGI Symbol;Acc:MGI:108424]	1.828	1.40E-03
ENSMUSG00000030322	Mbd4	methyl-CpG binding domain protein 4 [Source:MGI Symbol;Acc:MGI:1333850]	1.828	1.99E-02
ENSMUSG00000029176	Anapc4	anaphase promoting complex subunit 4 [Source:MGI Symbol;Acc:MGI:1098673]	1.828	2.22E-03
ENSMUSG00000034647	Ankrd12	ankyrin repeat domain 12 [Source:MGI Symbol;Acc:MGI:1914357]	1.827	1.46E-06
ENSMUSG00000013236	Ptprs	"protein tyrosine phosphatase, receptor type, S [Source:MGI Symbol;Acc:MGI:97815]"	1.825	3.01E-03
ENSMUSG00000104571	Gm43010	predicted gene 43010 [Source:MGI Symbol;Acc:MGI:5663147]	1.824	9.39E-04
ENSMUSG00000108388	Gm44673	predicted gene 44673 [Source:MGI Symbol;Acc:MGI:5753249]	1.823	1.35E-02
ENSMUSG00000094377	Gm24407	"predicted gene, 24407 [Source:MGI Symbol;Acc:MGI:5454184]"	1.823	9.62E-09
ENSMUSG00000109852	Gm45360	predicted gene 45360 [Source:MGI Symbol;Acc:MGI:5791196]	1.823	2.32E-02
ENSMUSG00000058672	Tubb2a	"tubulin, beta 2A class IIA [Source:MGI Symbol;Acc:MGI:107861]"	1.822	2.61E-09
ENSMUSG00000027613	Eif6	eukaryotic translation initiation factor 6 [Source:MGI Symbol;Acc:MGI:1196288]	1.822	6.62E-08
ENSMUSG00000022894	Adamts5	"a disintegrin-like and metallopeptidase (reprolysin type) with thrombospondin type 1 motif, 5 (aggrecanase-2) [Source:MGI Symbol;Acc:MGI:1346321]"	1.822	1.65E-03
ENSMUSG00000106458	Gm42633	predicted gene 42633 [Source:MGI Symbol;Acc:MGI:5662770]	1.821	5.78E-04
ENSMUSG00000102854	C130023A14Rik	RIKEN cDNA C130023A14 gene [Source:MGI Symbol;Acc:MGI:2442654]	1.821	2.94E-05
ENSMUSG00000029798	Herc6	hect domain and RLD 6 [Source:MGI Symbol;Acc:MGI:1914388]	1.821	1.99E-10
ENSMUSG00000103132	Gm37978	"predicted gene, 37978 [Source:MGI Symbol;Acc:MGI:5611206]"	1.820	1.34E-06
ENSMUSG00000051735	Rinl	Ras and Rab interactor-like [Source:MGI Symbol;Acc:MGI:2444024]	1.820	1.66E-02
ENSMUSG00000022587	Ly6e	"lymphocyte antigen 6 complex, locus E [Source:MGI Symbol;Acc:MGI:106651]"	1.819	3.86E-05
ENSMUSG00000095595	Fam177a	"family with sequence similarity 177, member A [Source:MGI Symbol;Acc:MGI:1920635]"	1.819	5.00E-03
ENSMUSG00000029231	Pdgfra	"platelet derived growth factor receptor, alpha polypeptide [Source:MGI Symbol;Acc:MGI:97530]"	1.817	4.51E-04
ENSMUSG00000102642	A130048G24Rik	RIKEN cDNA A130048G24 gene [Source:MGI Symbol;Acc:MGI:2443520]	1.817	9.24E-06
ENSMUSG00000097310	A930038B10Rik	RIKEN cDNA A930038B10 gene [Source:MGI Symbol;Acc:MGI:2443813]	1.817	1.48E-03
ENSMUSG00000034342	Cbl	Casitas B-lineage lymphoma [Source:MGI Symbol;Acc:MGI:88279]	1.816	1.96E-05
ENSMUSG00000062995	Ica1	islet cell autoantigen 1 [Source:MGI Symbol;Acc:MGI:96391]	1.816	4.11E-02
ENSMUSG00000095649	Gm8979	predicted gene 8979 [Source:MGI Symbol;Acc:MGI:3703149]	1.815	1.55E-03
ENSMUSG00000031402	Mpp1	"membrane protein, palmitoylated [Source:MGI Symbol;Acc:MGI:105941]"	1.814	4.62E-07
ENSMUSG00000024620	Pdgfrb	"platelet derived growth factor receptor, beta polypeptide [Source:MGI Symbol;Acc:MGI:97531]"	1.814	2.37E-03
ENSMUSG00000017929	B4galt5	"UDP-Gal:betaGlcNAc beta 1,4-galactosyltransferase, polypeptide 5 [Source:MGI Symbol;Acc:MGI:1927169]"	1.814	7.14E-07
ENSMUSG00000050271	Prag1	PEAK1 related kinase activating pseudokinase 1 [Source:MGI Symbol;Acc:MGI:1196223]	1.813	3.63E-02
ENSMUSG00000019929	Dcn	decorin [Source:MGI Symbol;Acc:MGI:94872]	1.813	9.58E-04
ENSMUSG00000114169	Gm47075	"predicted gene, 47075 [Source:MGI Symbol;Acc:MGI:6095795]"	1.812	1.75E-03
ENSMUSG00000021423	Ly86	lymphocyte antigen 86 [Source:MGI Symbol;Acc:MGI:1321404]	1.812	5.66E-05
ENSMUSG00000002871	Tpra1	"transmembrane protein, adipocyte asscociated 1 [Source:MGI Symbol;Acc:MGI:1345190]"	1.812	1.80E-02

ENSMUSG00000099587	Gm28967	predicted gene 28967 [Source:MGI Symbol;Acc:MGI:5579673]	1.812	2.98E-04
ENSMUSG00000036594	H2-Aa	"histocompatibility 2, class II antigen A, alpha [Source:MGI Symbol;Acc:MGI:95895]"	1.811	8.99E-03
ENSMUSG00000030084	Plxna1	plexin A1 [Source:MGI Symbol;Acc:MGI:107685]	1.810	8.57E-09
ENSMUSG00000031838	Ifi30	interferon gamma inducible protein 30 [Source:MGI Symbol;Acc:MGI:2137648]	1.806	7.85E-04
ENSMUSG00000036469	Marchf1	membrane associated ring-CH-type finger 1 [Source:MGI Symbol;Acc:MGI:1920175]	1.806	8.64E-05
ENSMUSG00000024831	Ighmbp2	immunoglobulin mu binding protein 2 [Source:MGI Symbol;Acc:MGI:99954]	1.805	8.61E-03
ENSMUSG00000103624	Gm3081	predicted gene 3081 [Source:MGI Symbol;Acc:MGI:3781258]	1.804	1.53E-03
ENSMUSG00000079470	Utp14b	UTP14B small subunit processome component [Source:MGI Symbol;Acc:MGI:2445092]	1.803	3.01E-08
ENSMUSG00000102419	Gm36940	"predicted gene, 36940 [Source:MGI Symbol;Acc:MGI:5610168]"	1.802	1.04E-04
ENSMUSG00000086316	Nbdy	negative regulator of P-body association [Source:MGI Symbol;Acc:MGI:1917373]	1.801	1.38E-02
ENSMUSG00000024824	Rad9a	RAD9 checkpoint clamp component A [Source:MGI Symbol;Acc:MGI:1328356]	1.801	4.68E-02
ENSMUSG00000105230	Gm42433	predicted gene 42433 [Source:MGI Symbol;Acc:MGI:5662570]	1.801	3.95E-02
ENSMUSG00000105466	Gm42998	predicted gene 42998 [Source:MGI Symbol;Acc:MGI:5663135]	1.800	9.51E-03
ENSMUSG00000049775	Tmsb4x	"thymosin, beta 4, X chromosome [Source:MGI Symbol;Acc:MGI:99510]"	1.800	1.91E-03
ENSMUSG00000028278	Rragd	Ras-related GTP binding D [Source:MGI Symbol;Acc:MGI:1098604]	1.800	3.40E-02
ENSMUSG00000104018	4833412K13Rik	RIKEN cDNA 4833412K13 gene [Source:MGI Symbol;Acc:MGI:1921857]	1.800	2.30E-03
ENSMUSG00000031207	Msn	moesin [Source:MGI Symbol;Acc:MGI:97167]	1.797	6.87E-04
ENSMUSG00000108954	Gm44901	predicted gene 44901 [Source:MGI Symbol;Acc:MGI:5753477]	1.796	3.13E-04
ENSMUSG00000098066	Gm26944	"predicted gene, 26944 [Source:MGI Symbol;Acc:MGI:5504059]"	1.795	2.25E-02
ENSMUSG00000025366	Esyt1	extended synaptotagmin-like protein 1 [Source:MGI Symbol;Acc:MGI:1344426]	1.795	6.39E-06
ENSMUSG00000002845	Tmem39a	transmembrane protein 39a [Source:MGI Symbol;Acc:MGI:1915096]	1.795	2.71E-03
ENSMUSG00000043004	Gng2	"guanine nucleotide binding protein (G protein), gamma 2 [Source:MGI Symbol;Acc:MGI:102705]"	1.794	1.34E-02
ENSMUSG00000104107	Gm37879	"predicted gene, 37879 [Source:MGI Symbol;Acc:MGI:5611107]"	1.794	9.43E-04
ENSMUSG00000026020	Nop58	NOP58 ribonucleoprotein [Source:MGI Symbol;Acc:MGI:1933184]	1.792	4.15E-05
ENSMUSG00000026064	Ptp4a1	protein tyrosine phosphatase 4a1 [Source:MGI Symbol;Acc:MGI:1277096]	1.792	1.22E-03
ENSMUSG00000104947	Gm43721	predicted gene 43721 [Source:MGI Symbol;Acc:MGI:5663858]	1.791	2.03E-02
ENSMUSG00000029094	Afap1	actin filament associated protein 1 [Source:MGI Symbol;Acc:MGI:1917542]	1.790	1.10E-02
ENSMUSG00000070814	Zswim9	zinc finger SWIM-type containing 9 [Source:MGI Symbol;Acc:MGI:2447816]	1.789	4.56E-05
ENSMUSG00000056708	Ier5	immediate early response 5 [Source:MGI Symbol;Acc:MGI:1337072]	1.789	2.87E-05
ENSMUSG00000007891	Ctsd	cathepsin D [Source:MGI Symbol;Acc:MGI:88562]	1.789	8.21E-13
ENSMUSG00000052565	H1f3	"H1.3 linker histone, cluster member [Source:MGI Symbol;Acc:MGI:107502]"	1.787	1.11E-02
ENSMUSG00000038178	Slc43a2	"solute carrier family 43, member 2 [Source:MGI Symbol;Acc:MGI:2442746]"	1.787	3.29E-06
ENSMUSG00000031274	Col4a5	"collagen, type IV, alpha 5 [Source:MGI Symbol;Acc:MGI:88456]"	1.787	2.76E-02
ENSMUSG00000115662	Gm7232	predicted gene 7232 [Source:MGI Symbol;Acc:MGI:3645247]	1.787	7.58E-03
ENSMUSG00000103252	Gm37521	"predicted gene, 37521 [Source:MGI Symbol;Acc:MGI:5610749]"	1.786	4.34E-02
ENSMUSG00000094526	Gm21451	"predicted gene, 21451 [Source:MGI Symbol;Acc:MGI:5434806]"	1.786	3.02E-02
ENSMUSG00000025790	Slco3a1	"solute carrier organic anion transporter family, member 3a1 [Source:MGI Symbol;Acc:MGI:1351867]"	1.786	1.14E-02
ENSMUSG00000005397	Nid1	nidogen 1 [Source:MGI Symbol;Acc:MGI:97342]	1.785	1.58E-03
ENSMUSG00000069631	Strada	STE20-related kinase adaptor alpha [Source:MGI Symbol;Acc:MGI:1919399]	1.785	7.08E-05
ENSMUSG00000024251	Thada	thyroid adenoma associated [Source:MGI Symbol;Acc:MGI:3039623]	1.785	1.33E-03
ENSMUSG00000002897	Il17ra	interleukin 17 receptor A [Source:MGI Symbol;Acc:MGI:107399]	1.785	1.22E-04
ENSMUSG00000035493	Tgfbi	"transforming growth factor, beta induced [Source:MGI Symbol;Acc:MGI:99959]"	1.785	7.39E-04
ENSMUSG00000072235	Tuba1a	"tubulin, alpha 1A [Source:MGI Symbol;Acc:MGI:98869]"	1.783	9.46E-05
ENSMUSG00000056608	Chd9	chromodomain helicase DNA binding protein 9 [Source:MGI Symbol;Acc:MGI:1924001]	1.783	1.34E-03
ENSMUSG00000104378	Gm37510	"predicted gene, 37510 [Source:MGI Symbol;Acc:MGI:5610738]"	1.782	7.75E-04
ENSMUSG00000014329	Bicc1	BicC family RNA binding protein 1 [Source:MGI Symbol;Acc:MGI:1933388]	1.781	1.57E-02
ENSMUSG00000020649	Rrm2	ribonucleotide reductase M2 [Source:MGI Symbol;Acc:MGI:98181]	1.779	2.09E-03
ENSMUSG00000095590	Gm24305	"predicted gene, 24305 [Source:MGI Symbol;Acc:MGI:5454082]"	1.779	6.98E-11
ENSMUSG00000085156	Snhg15	small nucleolar RNA host gene 15 [Source:MGI Symbol;Acc:MGI:3650059]	1.779	5.17E-03
ENSMUSG00000007613	Tgfbr1	"transforming growth factor, beta receptor I [Source:MGI Symbol;Acc:MGI:98728]"	1.778	1.06E-09

ENSMUSG00000094103	Fam177a2	family with sequence similarity 177 member A2 [Source:MGI Symbol;Acc:MGI:3714351]	1.778	6.20E-03
ENSMUSG00000030726	Pold3	"polymerase (DNA-directed), delta 3, accessory subunit [Source:MGI Symbol;Acc:MGI:1915217]"	1.778	1.32E-03
ENSMUSG00000113076	Gm47088	"predicted gene, 47088 [Source:MGI Symbol;Acc:MGI:6095816]"	1.778	1.63E-02
ENSMUSG00000022964	Tmem50b	transmembrane protein 50B [Source:MGI Symbol;Acc:MGI:1925225]	1.777	4.27E-03
ENSMUSG00000027342	Pcna	proliferating cell nuclear antigen [Source:MGI Symbol;Acc:MGI:97503]	1.777	1.83E-04
ENSMUSG00000041025	Iffo2	intermediate filament family orphan 2 [Source:MGI Symbol;Acc:MGI:2140675]	1.776	4.97E-09
ENSMUSG00000022148	Fyb	FYN binding protein [Source:MGI Symbol;Acc:MGI:1346327]	1.776	3.38E-08
ENSMUSG00000039585	Myo9a	myosin IXa [Source:MGI Symbol;Acc:MGI:107735]	1.774	2.19E-05
ENSMUSG00000068245	Phf11d	PHD finger protein 11D [Source:MGI Symbol;Acc:MGI:1277133]	1.774	3.74E-04
ENSMUSG00000029622	Arpc1b	"actin related protein 2/3 complex, subunit 1B [Source:MGI Symbol;Acc:MGI:1343142]"	1.774	2.35E-05
ENSMUSG00000005583	Mef2c	myocyte enhancer factor 2C [Source:MGI Symbol;Acc:MGI:99458]	1.773	2.55E-04
ENSMUSG00000109784	Gm45493	predicted gene 45493 [Source:MGI Symbol;Acc:MGI:5791329]	1.772	1.07E-02
ENSMUSG00000109245	Gm44860	predicted gene 44860 [Source:MGI Symbol;Acc:MGI:5753436]	1.771	1.75E-03
ENSMUSG00000102504	Gm21955	"predicted gene, 21955 [Source:MGI Symbol;Acc:MGI:5439424]"	1.771	2.53E-04
ENSMUSG00000034401	Spata6	spermatogenesis associated 6 [Source:MGI Symbol;Acc:MGI:1915196]	1.770	2.70E-02
ENSMUSG00000033857	Engase	endo-beta-N-acetylglucosaminidase [Source:MGI Symbol;Acc:MGI:2443788]	1.770	2.19E-02
ENSMUSG00000028270	Gbp2	guanylate binding protein 2 [Source:MGI Symbol;Acc:MGI:102772]	1.770	6.51E-03
ENSMUSG00000109363	Gm44668	predicted gene 44668 [Source:MGI Symbol;Acc:MGI:5753244]	1.769	1.65E-08
ENSMUSG00000096838	Gm26232	"predicted gene, 26232 [Source:MGI Symbol;Acc:MGI:5456009]"	1.769	5.22E-06
ENSMUSG00000044768	D1Ertd622e	"DNA segment, Chr 1, ERATO Doi 622, expressed [Source:MGI Symbol;Acc:MGI:1277184]"	1.768	6.53E-11
ENSMUSG00000060441	Trim5	tripartite motif-containing 5 [Source:MGI Symbol;Acc:MGI:3646853]	1.768	6.90E-03
ENSMUSG00000064090	Vrk2	vaccinia related kinase 2 [Source:MGI Symbol;Acc:MGI:1917172]	1.767	7.62E-06
ENSMUSG00000024610	Cd74	"CD74 antigen (invariant polypeptide of major histocompatibility complex, class II antigen-associated) [Source:MGI Symbol;Acc:MGI:96534]"	1.767	1.06E-02
ENSMUSG00000095969	Rnu1a1	U1a1 small nuclear RNA [Source:MGI Symbol;Acc:MGI:97972]	1.766	2.33E-09
ENSMUSG00000041859	Mcm3	minichromosome maintenance complex component 3 [Source:MGI Symbol;Acc:MGI:101845]	1.766	8.98E-04
ENSMUSG00000055612	Cdca7	cell division cycle associated 7 [Source:MGI Symbol;Acc:MGI:1914203]	1.766	3.81E-02
ENSMUSG00000102782	Gm37625	"predicted gene, 37625 [Source:MGI Symbol;Acc:MGI:5610853]"	1.765	1.81E-02
ENSMUSG00000108443	Gm44510	predicted gene 44510 [Source:MGI Symbol;Acc:MGI:5753086]	1.763	1.94E-02
ENSMUSG00000106149	Gm43430	predicted gene 43430 [Source:MGI Symbol;Acc:MGI:5663567]	1.762	1.31E-04
ENSMUSG00000025077	Dclre1a	DNA cross-link repair 1A [Source:MGI Symbol;Acc:MGI:1930042]	1.761	4.97E-04
ENSMUSG00000103360	Gm38156	"predicted gene, 38156 [Source:MGI Symbol;Acc:MGI:5611384]"	1.761	2.63E-03
ENSMUSG00000026019	Wdr12	WD repeat domain 12 [Source:MGI Symbol;Acc:MGI:1927241]	1.761	4.04E-03
ENSMUSG00000078453	Abracl	ABRA C-terminal like [Source:MGI Symbol;Acc:MGI:1920362]	1.760	1.58E-02
ENSMUSG00000106229	Gm19409	"predicted gene, 19409 [Source:MGI Symbol;Acc:MGI:5011594]"	1.760	2.32E-04
ENSMUSG00000064061	Dzip3	"DAZ interacting protein 3, zinc finger [Source:MGI Symbol;Acc:MGI:1917433]"	1.760	1.71E-03
ENSMUSG00000026864	Hspa5	heat shock protein 5 [Source:MGI Symbol;Acc:MGI:95835]	1.759	9.25E-12
ENSMUSG00000063245	Zfp993	zinc finger protein 993 [Source:MGI Symbol;Acc:MGI:3713585]	1.759	1.21E-02
ENSMUSG00000106166	Gm43484	predicted gene 43484 [Source:MGI Symbol;Acc:MGI:5663621]	1.758	6.40E-04
ENSMUSG00000079671	2610203C22Rik	RIKEN cDNA 2610203C22 gene [Source:MGI Symbol;Acc:MGI:1919731]	1.756	3.45E-03
ENSMUSG00000034947	Tmem106a	transmembrane protein 106A [Source:MGI Symbol;Acc:MGI:1922056]	1.756	2.08E-04
ENSMUSG00000041354	Rgl2	ral guanine nucleotide dissociation stimulator-like 2 [Source:MGI Symbol;Acc:MGI:107483]	1.756	6.17E-03
ENSMUSG00000026082	Rev1	"REV1, DNA directed polymerase [Source:MGI Symbol;Acc:MGI:1929074]"	1.756	2.62E-02
ENSMUSG00000106717	Gm42798	predicted gene 42798 [Source:MGI Symbol;Acc:MGI:5662935]	1.756	3.41E-05
ENSMUSG00000056220	Pla2g4a	"phospholipase A2, group IVA (cytosolic, calcium-dependent) [Source:MGI Symbol;Acc:MGI:1195256]"	1.755	2.04E-04
ENSMUSG00000104940	Gm42908	predicted gene 42908 [Source:MGI Symbol;Acc:MGI:5663045]	1.754	1.39E-02
ENSMUSG00000021277	Traf3	TNF receptor-associated factor 3 [Source:MGI Symbol;Acc:MGI:108041]	1.754	2.25E-03
ENSMUSG00000029234	Tmem165	transmembrane protein 165 [Source:MGI Symbol;Acc:MGI:894407]	1.754	4.36E-04
ENSMUSG00000023915	Tnfrsf21	"tumor necrosis factor receptor superfamily, member 21 [Source:MGI Symbol;Acc:MGI:2151075]"	1.754	1.92E-02
ENSMUSG00000035356	Nfkbiz	"nuclear factor of kappa light polypeptide gene enhancer in B cells inhibitor, zeta [Source:MGI Symbol;Acc:MGI:1931595]"	1.753	2.84E-05
ENSMUSG00000030577	Cd22	CD22 antigen [Source:MGI Symbol;Acc:MGI:88322]	1.753	1.49E-02

ENSMUSG00000027490	E2f1	E2F transcription factor 1 [Source:MGI Symbol;Acc:MGI:101941]	1.753	3.36E-03
ENSMUSG00000027435	Cd93	CD93 antigen [Source:MGI Symbol;Acc:MGI:106664]	1.752	1.14E-03
ENSMUSG00000006342	Susd2	sushi domain containing 2 [Source:MGI Symbol;Acc:MGI:1918983]	1.751	2.87E-02
ENSMUSG00000022186	Oxct1	3-oxoacid CoA transferase 1 [Source:MGI Symbol;Acc:MGI:1914291]	1.750	2.80E-04
ENSMUSG00000074342	I830077J02Rik	RIKEN cDNA I830077J02 gene [Source:MGI Symbol;Acc:MGI:3588284]	1.749	3.73E-02
ENSMUSG00000011256	Adam19	a disintegrin and metallopeptidase domain 19 (meltrin beta) [Source:MGI Symbol;Acc:MGI:105377]	1.749	3.81E-02
ENSMUSG00000021670	Hmgcr	3-hydroxy-3-methylglutaryl-Coenzyme A reductase [Source:MGI Symbol;Acc:MGI:96159]	1.748	1.06E-05
ENSMUSG00000024170	Telo2	telomere maintenance 2 [Source:MGI Symbol;Acc:MGI:1918968]	1.746	2.59E-02
ENSMUSG00000102423	Gm37465	"predicted gene, 37465 [Source:MGI Symbol;Acc:MGI:5610693]"	1.746	2.49E-04
ENSMUSG00000020876	Snx11	sorting nexin 11 [Source:MGI Symbol;Acc:MGI:1921729]	1.746	4.11E-03
ENSMUSG00000100005	B130024G19Rik	RIKEN cDNA B130024G19 gene [Source:MGI Symbol;Acc:MGI:3604353]	1.745	1.18E-03
ENSMUSG00000064210	Ano6	anoctamin 6 [Source:MGI Symbol;Acc:MGI:2145890]	1.745	2.17E-08
ENSMUSG00000095115	Itpripl2	"inositol 1,4,5-triphosphate receptor interacting protein-like 2 [Source:MGI Symbol;Acc:MGI:2442416]"	1.745	4.56E-04
ENSMUSG00000014905	Dnajb9	DnaJ heat shock protein family (Hsp40) member B9 [Source:MGI Symbol;Acc:MGI:1351618]	1.742	1.28E-09
ENSMUSG00000020275	Rel	reticuloendotheliosis oncogene [Source:MGI Symbol;Acc:MGI:97897]	1.742	2.69E-08
ENSMUSG00000019796	Lrp11	low density lipoprotein receptor-related protein 11 [Source:MGI Symbol;Acc:MGI:2442989]	1.742	4.50E-02
ENSMUSG00000024030	Abcg1	ATP binding cassette subfamily G member 1 [Source:MGI Symbol;Acc:MGI:107704]	1.742	1.60E-04
ENSMUSG00000104713	Gbp6	guanylate binding protein 6 [Source:MGI Symbol;Acc:MGI:2140937]	1.740	1.11E-03
ENSMUSG00000020100	Slc29a3	"solute carrier family 29 (nucleoside transporters), member 3 [Source:MGI Symbol;Acc:MGI:1918529]"	1.739	3.86E-04
ENSMUSG00000021365	Nedd9	"neural precursor cell expressed, developmentally down-regulated gene 9 [Source:MGI Symbol;Acc:MGI:97302]"	1.739	1.58E-02
ENSMUSG00000037849	Ifi206	interferon activated gene 206 [Source:MGI Symbol;Acc:MGI:3646410]	1.738	1.74E-02
ENSMUSG00000084350	Znf41-ps	"ZNF41, pseudogene [Source:MGI Symbol;Acc:MGI:1917255]"	1.738	2.37E-03
ENSMUSG00000020810	Cygb	cytoglobin [Source:MGI Symbol;Acc:MGI:2149481]	1.738	2.18E-02
ENSMUSG00000003283	Hck	hemopoietic cell kinase [Source:MGI Symbol;Acc:MGI:96052]	1.736	6.61E-03
ENSMUSG00000045211	Nudt18	nudix (nucleoside diphosphate linked moiety X)-type motif 18 [Source:MGI Symbol;Acc:MGI:2385853]	1.736	1.68E-03
ENSMUSG00000055413	H2-Q5	"histocompatibility 2, Q region locus 5 [Source:MGI Symbol;Acc:MGI:95934]"	1.735	1.54E-02
ENSMUSG00000106115	Gm43420	predicted gene 43420 [Source:MGI Symbol;Acc:MGI:5663557]	1.734	3.96E-02
ENSMUSG00000005667	Mthfd2	"methylenetetrahydrofolate dehydrogenase (NAD+ dependent), methenyltetrahydrofolate cyclohydrolase [Source:MGI Symbol;Acc:MGI:1338850]"	1.734	3.50E-03
ENSMUSG00000026425	Srgap2	SLIT-ROBO Rho GTPase activating protein 2 [Source:MGI Symbol;Acc:MGI:109605]	1.733	8.90E-04
ENSMUSG00000030556	Lrrc28	leucine rich repeat containing 28 [Source:MGI Symbol;Acc:MGI:1915689]	1.733	1.76E-09
ENSMUSG00000034959	Rubcnl	RUN and cysteine rich domain containing beclin 1 interacting protein like [Source:MGI Symbol;Acc:MGI:2685590]	1.733	2.17E-02
ENSMUSG00000026773	Pfkfb3	"6-phosphofructo-2-kinase/fructose-2,6-biphosphatase 3 [Source:MGI Symbol;Acc:MGI:2181202]"	1.733	7.35E-07
ENSMUSG00000015745	Plekho1	"pleckstrin homology domain containing, family O member 1 [Source:MGI Symbol;Acc:MGI:1914470]"	1.732	1.37E-02
ENSMUSG00000086825	Gm15675	predicted gene 15675 [Source:MGI Symbol;Acc:MGI:3783117]	1.732	1.85E-05
ENSMUSG00000046826	Fam187b	"family with sequence similarity 187, member B [Source:MGI Symbol;Acc:MGI:1923665]"	1.732	2.33E-02
ENSMUSG00000108912	E230020D15Rik	RIKEN cDNA E230020D15 gene [Source:MGI Symbol;Acc:MGI:2442650]	1.732	1.74E-02
ENSMUSG00000027750	Postn	"periostin, osteoblast specific factor [Source:MGI Symbol;Acc:MGI:1926321]"	1.732	4.31E-03
ENSMUSG00000086859	Snhg20	small nucleolar RNA host gene 20 [Source:MGI Symbol;Acc:MGI:1924222]	1.730	1.51E-02
ENSMUSG00000064851	Gm24525	"predicted gene, 24525 [Source:MGI Symbol;Acc:MGI:5454302]"	1.730	3.11E-02
ENSMUSG00000025888	Casp1	caspase 1 [Source:MGI Symbol;Acc:MGI:96544]	1.728	1.13E-02
ENSMUSG00000026483	Fam129a	"family with sequence similarity 129, member A [Source:MGI Symbol;Acc:MGI:2137237]"	1.727	1.11E-02
ENSMUSG00000116581	Gm46565	"predicted gene, 46565 [Source:MGI Symbol;Acc:MGI:5826202]"	1.726	1.38E-02
ENSMUSG00000103747	Gm38236	"predicted gene, 38236 [Source:MGI Symbol;Acc:MGI:5611464]"	1.726	2.28E-02
ENSMUSG00000020160	Meis1	Meis homeobox 1 [Source:MGI Symbol;Acc:MGI:104717]	1.726	9.55E-03
ENSMUSG00000095701	Gm24830	"predicted gene, 24830 [Source:MGI Symbol;Acc:MGI:5454607]"	1.726	1.41E-05
ENSMUSG00000025225	Nfkb2	"nuclear factor of kappa light polypeptide gene enhancer in B cells 2, p49/p100 [Source:MGI Symbol;Acc:MGI:1099800]"	1.726	5.34E-04
ENSMUSG00000028864	Hgf	hepatocyte growth factor [Source:MGI Symbol;Acc:MGI:96079]	1.723	5.30E-05
ENSMUSG00000027360	Hdc	histidine decarboxylase [Source:MGI Symbol;Acc:MGI:96062]	1.723	2.96E-03
ENSMUSG00000090031	4732440D04Rik	RIKEN cDNA 4732440D04 gene [Source:MGI Symbol;Acc:MGI:3604103]	1.723	2.71E-04
ENSMUSG00000109539	Gm44667	predicted gene 44667 [Source:MGI Symbol;Acc:MGI:5753243]	1.722	4.46E-10

ENSMUSG00000069893	9930111J21Rik1	RIKEN cDNA 9930111J21 gene 1 [Source:MGI Symbol;Acc:MGI:3041173]	1.721	2.19E-02
ENSMUSG00000030795	Fus	fused in sarcoma [Source:MGI Symbol;Acc:MGI:1353633]	1.721	4.68E-08
ENSMUSG00000060586	H2-Eb1	"histocompatibility 2, class II antigen E beta [Source:MGI Symbol;Acc:MGI:95901]"	1.721	5.35E-03
ENSMUSG00000070034	Sp110	Sp110 nuclear body protein [Source:MGI Symbol;Acc:MGI:1923364]	1.720	1.18E-02
ENSMUSG00000102496	Gm36989	"predicted gene, 36989 [Source:MGI Symbol;Acc:MGI:5610217]"	1.720	1.20E-05
ENSMUSG00000030720	Cln3	"ceroid lipofuscinosis, neuronal 3, juvenile (Batten, Spielmeyer-Vogt disease) [Source:MGI Symbol;Acc:MGI:107537]"	1.720	3.48E-03
ENSMUSG00000070327	Rnf213	ring finger protein 213 [Source:MGI Symbol;Acc:MGI:1289196]	1.719	3.06E-08
ENSMUSG00000106186	Gm43627	predicted gene 43627 [Source:MGI Symbol;Acc:MGI:5663764]	1.719	1.67E-02
ENSMUSG00000105510	Gm43815	predicted gene 43815 [Source:MGI Symbol;Acc:MGI:5663952]	1.719	1.06E-02
ENSMUSG00000025044	Msr1	macrophage scavenger receptor 1 [Source:MGI Symbol;Acc:MGI:98257]	1.715	7.38E-05
ENSMUSG00000105987	AI506816	expressed sequence AI506816 [Source:MGI Symbol;Acc:MGI:2140929]	1.713	5.88E-05
ENSMUSG00000030787	Lyve1	lymphatic vessel endothelial hyaluronan receptor 1 [Source:MGI Symbol;Acc:MGI:2136348]	1.713	1.94E-03
ENSMUSG00000108526	Gm45828	predicted gene 45828 [Source:MGI Symbol;Acc:MGI:5804943]	1.713	6.98E-03
ENSMUSG00000097585	E230029C05Rik	RIKEN cDNA E230029C05 gene [Source:MGI Symbol;Acc:MGI:2442580]	1.713	2.00E-02
ENSMUSG00000051278	Zgrf1	"zinc finger, GRF-type containing 1 [Source:MGI Symbol;Acc:MGI:1918893]"	1.713	1.05E-04
ENSMUSG00000027669	Gnb4	"guanine nucleotide binding protein (G protein), beta 4 [Source:MGI Symbol;Acc:MGI:104581]"	1.712	3.29E-03
ENSMUSG00000026796	Fam129b	"family with sequence similarity 129, member B [Source:MGI Symbol;Acc:MGI:2442910]"	1.712	7.40E-03
ENSMUSG00000023147	Get1	guided entry of tail-anchored proteins factor 1 [Source:MGI Symbol;Acc:MGI:2136882]	1.712	4.85E-03
ENSMUSG00000022639	Dubr	Dppa2 upstream binding RNA [Source:MGI Symbol;Acc:MGI:1915440]	1.712	7.74E-03
ENSMUSG00000024349	Sting1	stimulator of interferon response cGAMP interactor 1 [Source:MGI Symbol;Acc:MGI:1919762]	1.711	2.16E-02
ENSMUSG00000023994	Nfya	nuclear transcription factor-Y alpha [Source:MGI Symbol;Acc:MGI:97316]	1.711	1.31E-05
ENSMUSG00000112317	Gm47580	"predicted gene, 47580 [Source:MGI Symbol;Acc:MGI:6096616]"	1.711	9.93E-04
ENSMUSG00000023367	Tmem176a	transmembrane protein 176A [Source:MGI Symbol;Acc:MGI:1913308]	1.710	1.37E-11
ENSMUSG00000108214	Gm43982	"predicted gene, 43982 [Source:MGI Symbol;Acc:MGI:5690374]"	1.710	9.66E-07
ENSMUSG00000106251	Gm42658	predicted gene 42658 [Source:MGI Symbol;Acc:MGI:5662795]	1.710	1.88E-03
ENSMUSG00000103046	Gm37309	"predicted gene, 37309 [Source:MGI Symbol;Acc:MGI:5610537]"	1.710	3.15E-02
ENSMUSG00000050989	Selenon	selenoprotein N [Source:MGI Symbol;Acc:MGI:2151208]	1.709	1.51E-03
ENSMUSG00000029782	Tmem209	transmembrane protein 209 [Source:MGI Symbol;Acc:MGI:1919899]	1.709	9.16E-04
ENSMUSG00000086513	9130208D14Rik	RIKEN cDNA 9130208D14 gene [Source:MGI Symbol;Acc:MGI:1924950]	1.707	2.15E-02
ENSMUSG00000004099	Dnmt1	DNA methyltransferase (cytosine-5) 1 [Source:MGI Symbol;Acc:MGI:94912]	1.707	6.10E-04
ENSMUSG00000107792	Gm43914	"predicted gene, 43914 [Source:MGI Symbol;Acc:MGI:5690306]"	1.706	4.51E-02
ENSMUSG00000074024	4632427E13Rik	RIKEN cDNA 4632427E13 gene [Source:MGI Symbol;Acc:MGI:1915436]	1.706	1.42E-02
ENSMUSG00000021994	Wnt5a	"wingless-type MMTV integration site family, member 5A [Source:MGI Symbol;Acc:MGI:98958]"	1.705	3.88E-02
ENSMUSG00000024691	Fam111a	"family with sequence similarity 111, member A [Source:MGI Symbol;Acc:MGI:1915508]"	1.705	1.96E-04
ENSMUSG00000069662	Marcks	myristoylated alanine rich protein kinase C substrate [Source:MGI Symbol;Acc:MGI:96907]	1.704	5.59E-03
ENSMUSG00000096214	Gm22634	"predicted gene, 22634 [Source:MGI Symbol;Acc:MGI:5452411]"	1.702	2.69E-08
ENSMUSG00000047810	Ccdc88b	coiled-coil domain containing 88B [Source:MGI Symbol;Acc:MGI:1925567]	1.702	2.94E-02
ENSMUSG00000032332	Col12a1	"collagen, type XII, alpha 1 [Source:MGI Symbol;Acc:MGI:88448]"	1.701	2.41E-03
ENSMUSG00000027514	Zbp1	Z-DNA binding protein 1 [Source:MGI Symbol;Acc:MGI:1927449]	1.700	1.80E-09
ENSMUSG00000095892	Rnu5g	"RNA, U5G small nuclear [Source:MGI Symbol;Acc:MGI:2157900]"	1.700	5.31E-04
ENSMUSG00000103094	Gm37558	"predicted gene, 37558 [Source:MGI Symbol;Acc:MGI:5610786]"	1.700	8.89E-10
ENSMUSG00000101778	Gm29488	predicted gene 29488 [Source:MGI Symbol;Acc:MGI:5580194]	1.699	8.99E-04
ENSMUSG00000042978	Sbk1	SH3-binding kinase 1 [Source:MGI Symbol;Acc:MGI:2135937]	1.699	4.39E-03
ENSMUSG00000021932	Rnaseh2b	"ribonuclease H2, subunit B [Source:MGI Symbol;Acc:MGI:1914403]"	1.699	1.47E-03
ENSMUSG00000096205	Gm22068	"predicted gene, 22068 [Source:MGI Symbol;Acc:MGI:5451845]"	1.697	2.88E-09
ENSMUSG00000038510	Rpf2	ribosome production factor 2 homolog [Source:MGI Symbol;Acc:MGI:1914489]	1.696	1.08E-02
ENSMUSG00000036905	C1qb	"complement component 1, q subcomponent, beta polypeptide [Source:MGI Symbol;Acc:MGI:88224]"	1.696	1.47E-06
ENSMUSG00000104184	Gm37818	"predicted gene, 37818 [Source:MGI Symbol;Acc:MGI:5611046]"	1.694	3.57E-02
ENSMUSG00000042410	Agps	alkylglycerone phosphate synthase [Source:MGI Symbol;Acc:MGI:2443065]	1.693	5.06E-04
ENSMUSG00000040584	Abcb1a	"ATP-binding cassette, sub-family B (MDR/TAP), member 1A [Source:MGI Symbol;Acc:MGI:97570]"	1.693	2.94E-03

ENSMUSG00000004207	Psap	prosaposin [Source:MGI Symbol;Acc:MGI:97783]	1.693	1.73E-09
ENSMUSG00000000958	Slc7a7	"solute carrier family 7 (cationic amino acid transporter, y+ system), member 7 [Source:MGI Symbol;Acc:MGI:1337120]"	1.691	2.18E-02
ENSMUSG00000046080	Clec9a	"C-type lectin domain family 9, member a [Source:MGI Symbol;Acc:MGI:2444608]"	1.690	3.06E-03
ENSMUSG00000096206	Gm22317	"predicted gene, 22317 [Source:MGI Symbol;Acc:MGI:5452094]"	1.690	5.62E-07
ENSMUSG00000026107	Nabp1	nucleic acid binding protein 1 [Source:MGI Symbol;Acc:MGI:1923258]	1.689	4.86E-10
ENSMUSG00000104548	Gm43857	predicted gene 43857 [Source:MGI Symbol;Acc:MGI:5663994]	1.687	6.22E-03
ENSMUSG00000024042	Sik1	salt inducible kinase 1 [Source:MGI Symbol;Acc:MGI:104754]	1.687	7.35E-06
ENSMUSG00000025646	Atrip	ATR interacting protein [Source:MGI Symbol;Acc:MGI:1925349]	1.686	4.70E-03
ENSMUSG00000029366	Dck	deoxycytidine kinase [Source:MGI Symbol;Acc:MGI:102726]	1.683	1.05E-02
ENSMUSG00000021938	Pspc1	paraspeckle protein 1 [Source:MGI Symbol;Acc:MGI:1913895]	1.682	8.69E-04
ENSMUSG00000104728	Gm42462	predicted gene 42462 [Source:MGI Symbol;Acc:MGI:5662599]	1.682	1.73E-02
ENSMUSG00000047246	H2bc6	H2B clustered histone 6 [Source:MGI Symbol;Acc:MGI:2448380]	1.682	4.36E-02
ENSMUSG00000020077	Srgn	serglycin [Source:MGI Symbol;Acc:MGI:97756]	1.681	3.27E-02
ENSMUSG00000083261	Gm7816	predicted pseudogene 7816 [Source:MGI Symbol;Acc:MGI:3646286]	1.681	1.39E-02
ENSMUSG00000115837	9130002K18Rik	RIKEN cDNA 9130002K18 gene [Source:MGI Symbol;Acc:MGI:1921804]	1.680	6.13E-03
ENSMUSG00000030844	Rgs10	regulator of G-protein signalling 10 [Source:MGI Symbol;Acc:MGI:1915115]	1.679	1.52E-02
ENSMUSG00000114469	C730002L08Rik	RIKEN cDNA C730002L08 gene [Source:MGI Symbol;Acc:MGI:2443493]	1.678	4.28E-03
ENSMUSG00000031453	Rasa3	RAS p21 protein activator 3 [Source:MGI Symbol;Acc:MGI:1197013]	1.678	2.67E-04
ENSMUSG00000057335	Cep170	centrosomal protein 170 [Source:MGI Symbol;Acc:MGI:1918348]	1.676	1.88E-07
ENSMUSG00000104445	Rhbg	Rhesus blood group-associated B glycoprotein [Source:MGI Symbol;Acc:MGI:1927379]	1.676	1.85E-03
ENSMUSG00000060147	Serpinb6a	"serine (or cysteine) peptidase inhibitor, clade B, member 6a [Source:MGI Symbol;Acc:MGI:103123]"	1.675	4.51E-03
ENSMUSG00000095260	Gm25890	"predicted gene, 25890 [Source:MGI Symbol;Acc:MGI:5455667]"	1.675	1.07E-04
ENSMUSG00000024742	Fen1	flap structure specific endonuclease 1 [Source:MGI Symbol;Acc:MGI:102779]	1.675	1.80E-02
ENSMUSG00000003848	Nob1	NIN1/RPN12 binding protein 1 homolog [Source:MGI Symbol;Acc:MGI:1914869]	1.674	7.78E-03
ENSMUSG00000026627	Pacc1	proton activated chloride channel 1 [Source:MGI Symbol;Acc:MGI:1914200]	1.674	2.20E-02
ENSMUSG00000103591	Gm38365	"predicted gene, 38365 [Source:MGI Symbol;Acc:MGI:5611593]"	1.673	4.33E-04
ENSMUSG00000029992	Gfpt1	glutamine fructose-6-phosphate transaminase 1 [Source:MGI Symbol;Acc:MGI:95698]	1.673	6.29E-11
ENSMUSG00000078921	Tgtp2	T cell specific GTPase 2 [Source:MGI Symbol;Acc:MGI:3710083]	1.672	7.36E-03
ENSMUSG00000002699	Lcp2	lymphocyte cytosolic protein 2 [Source:MGI Symbol;Acc:MGI:1321402]	1.672	9.58E-03
ENSMUSG00000039621	Prex1	"phosphatidylinositol-3,4,5-trisphosphate-dependent Rac exchange factor 1 [Source:MGI Symbol;Acc:MGI:3040696]"	1.672	1.33E-04
ENSMUSG00000031907	Zfp90	zinc finger protein 90 [Source:MGI Symbol;Acc:MGI:104786]	1.669	1.20E-03
ENSMUSG00000056602	Fry	FRY microtubule binding protein [Source:MGI Symbol;Acc:MGI:2443895]	1.668	1.32E-02
ENSMUSG00000028289	Epha7	Eph receptor A7 [Source:MGI Symbol;Acc:MGI:95276]	1.667	4.62E-02
ENSMUSG00000026737	Pip4k2a	"phosphatidylinositol-5-phosphate 4-kinase, type II, alpha [Source:MGI Symbol;Acc:MGI:1298206]"	1.666	3.31E-02
ENSMUSG00000066595	Flvcr1	feline leukemia virus subgroup C cellular receptor 1 [Source:MGI Symbol;Acc:MGI:2444881]	1.665	4.35E-04
ENSMUSG00000104262	Gm37747	"predicted gene, 37747 [Source:MGI Symbol;Acc:MGI:5610975]"	1.665	3.28E-02
ENSMUSG00000054293	P2ry10b	"purinergic receptor P2Y, G-protein coupled 10B [Source:MGI Symbol;Acc:MGI:2441814]"	1.665	4.98E-02
ENSMUSG00000040350	Trim7	tripartite motif-containing 7 [Source:MGI Symbol;Acc:MGI:2137353]	1.664	1.31E-03
ENSMUSG00000103122	Gm37391	"predicted gene, 37391 [Source:MGI Symbol;Acc:MGI:5610619]"	1.663	3.98E-03
ENSMUSG00000085875	Gm12905	predicted gene 12905 [Source:MGI Symbol;Acc:MGI:3702581]	1.663	8.14E-03
ENSMUSG00000034993	Vat1	vesicle amine transport 1 [Source:MGI Symbol;Acc:MGI:1349450]	1.663	1.02E-06
ENSMUSG00000104324	Gm37320	"predicted gene, 37320 [Source:MGI Symbol;Acc:MGI:5610548]"	1.663	1.17E-03
ENSMUSG00000101493	2810405F17Rik	RIKEN cDNA 2810405F17 gene [Source:MGI Symbol;Acc:MGI:1917225]	1.662	2.30E-02
ENSMUSG00000105842	Gm43329	predicted gene 43329 [Source:MGI Symbol;Acc:MGI:5663466]	1.662	3.63E-05
ENSMUSG00000089726	Mir17hg	Mir17 host gene (non-protein coding) [Source:MGI Symbol;Acc:MGI:1923207]	1.662	6.26E-03
ENSMUSG00000053581	Zfand2a	"zinc finger, AN1-type domain 2A [Source:MGI Symbol;Acc:MGI:2140729]"	1.661	2.51E-04
ENSMUSG00000026335	Pam	peptidylglycine alpha-amidating monooxygenase [Source:MGI Symbol;Acc:MGI:97475]	1.660	7.21E-03
ENSMUSG00000034254	Agpat1	"1-acylglycerol-3-phosphate O-acyltransferase 1 (lysophosphatidic acid acyltransferase, alpha) [Source:MGI Symbol;Acc:MGI:1932075]"	1.660	1.06E-02
ENSMUSG00000107476	Zfp862-ps	"zinc finger protein 862, pseudogene [Source:MGI Symbol;Acc:MGI:1889827]"	1.659	4.52E-02
ENSMUSG00000027035	Cers6	ceramide synthase 6 [Source:MGI Symbol;Acc:MGI:2442564]	1.658	3.31E-12

ENSMUSG00000023990	Tfeb	transcription factor EB [Source:MGI Symbol;Acc:MGI:103270]	1.658	2.46E-02
ENSMUSG00000030409	Dmpk	dystrophia myotonica-protein kinase [Source:MGI Symbol;Acc:MGI:94906]	1.658	6.44E-05
ENSMUSG00000000078	Klf6	Kruppel-like factor 6 [Source:MGI Symbol;Acc:MGI:1346318]	1.657	2.19E-05
ENSMUSG00000041920	Slc16a6	"solute carrier family 16 (monocarboxylic acid transporters), member 6 [Source:MGI Symbol;Acc:MGI:2144585]"	1.656	1.43E-05
ENSMUSG00000089417	Gm22009	"predicted gene, 22009 [Source:MGI Symbol;Acc:MGI:5451786]"	1.653	1.16E-05
ENSMUSG00000032596	Uba7	ubiquitin-like modifier activating enzyme 7 [Source:MGI Symbol;Acc:MGI:1349462]	1.653	9.27E-04
ENSMUSG00000104900	4930596I21Rik	RIKEN cDNA 4930596I21 gene [Source:MGI Symbol;Acc:MGI:1925479]	1.650	4.14E-02
ENSMUSG00000030659	Nucb2	nucleobindin 2 [Source:MGI Symbol;Acc:MGI:1858179]	1.649	2.67E-02
ENSMUSG00000079003	Samd1	sterile alpha motif domain containing 1 [Source:MGI Symbol;Acc:MGI:2142433]	1.647	7.56E-04
ENSMUSG00000034349	Smc4	structural maintenance of chromosomes 4 [Source:MGI Symbol;Acc:MGI:1917349]	1.646	1.49E-02
ENSMUSG00000105962	Gm42432	predicted gene 42432 [Source:MGI Symbol;Acc:MGI:5662569]	1.645	1.55E-02
ENSMUSG00000044906	4930503L19Rik	RIKEN cDNA 4930503L19 gene [Source:MGI Symbol;Acc:MGI:1922045]	1.644	6.63E-04
ENSMUSG00000105818	Gm43256	predicted gene 43256 [Source:MGI Symbol;Acc:MGI:5663393]	1.644	1.85E-04
ENSMUSG00000040722	Scamp5	secretory carrier membrane protein 5 [Source:MGI Symbol;Acc:MGI:1928948]	1.643	3.24E-04
ENSMUSG00000041773	Enc1	ectodermal-neural cortex 1 [Source:MGI Symbol;Acc:MGI:109610]	1.643	1.26E-03
ENSMUSG00000107756	Gm44164	"predicted gene, 44164 [Source:MGI Symbol;Acc:MGI:5690556]"	1.643	1.77E-06
ENSMUSG00000015501	Hivep2	human immunodeficiency virus type I enhancer binding protein 2 [Source:MGI Symbol;Acc:MGI:1338076]	1.643	1.13E-03
ENSMUSG00000022849	Hspbap1	Hspb associated protein 1 [Source:MGI Symbol;Acc:MGI:1913917]	1.643	2.90E-03
ENSMUSG00000020108	Ddit4	DNA-damage-inducible transcript 4 [Source:MGI Symbol;Acc:MGI:1921997]	1.642	3.61E-02
ENSMUSG00000028173	Wls	wntless WNT ligand secretion mediator [Source:MGI Symbol;Acc:MGI:1915401]	1.640	7.15E-03
ENSMUSG00000030232	Aebp2	AE binding protein 2 [Source:MGI Symbol;Acc:MGI:1338038]	1.640	6.37E-07
ENSMUSG00000032392	Parp16	"poly (ADP-ribose) polymerase family, member 16 [Source:MGI Symbol;Acc:MGI:2446133]"	1.639	7.81E-04
ENSMUSG00000105846	Gm43258	predicted gene 43258 [Source:MGI Symbol;Acc:MGI:5663395]	1.639	4.99E-05
ENSMUSG00000027712	Anxa5	annexin A5 [Source:MGI Symbol;Acc:MGI:106008]	1.639	1.21E-04
ENSMUSG00000042404	Dennd4b	DENN/MADD domain containing 4B [Source:MGI Symbol;Acc:MGI:2446201]	1.638	3.24E-03
ENSMUSG00000027322	Siglec1	"sialic acid binding Ig-like lectin 1, sialoadhesin [Source:MGI Symbol;Acc:MGI:99668]"	1.638	1.22E-04
ENSMUSG00000006732	Mettl1	methyltransferase like 1 [Source:MGI Symbol;Acc:MGI:1339986]	1.638	8.26E-03
ENSMUSG00000060519	Tor3a	"torsin family 3, member A [Source:MGI Symbol;Acc:MGI:1353652]"	1.638	1.56E-02
ENSMUSG00000102856	Gm37084	"predicted gene, 37084 [Source:MGI Symbol;Acc:MGI:5610312]"	1.638	6.21E-04
ENSMUSG00000018965	Ywhah	"tyrosine 3-monooxygenase/tryptophan 5-monooxygenase activation protein, eta polypeptide [Source:MGI Symbol;Acc:MGI:109194]"	1.637	1.32E-04
ENSMUSG00000027562	Car2	carbonic anhydrase 2 [Source:MGI Symbol;Acc:MGI:88269]	1.637	1.01E-02
ENSMUSG00000033192	Lpcat2	lysophosphatidylcholine acyltransferase 2 [Source:MGI Symbol;Acc:MGI:3606214]	1.636	2.08E-02
ENSMUSG00000037306	Man1c1	"mannosidase, alpha, class 1C, member 1 [Source:MGI Symbol;Acc:MGI:2446214]"	1.635	5.56E-03
ENSMUSG00000109498	Gm45222	predicted gene 45222 [Source:MGI Symbol;Acc:MGI:5753798]	1.634	4.52E-06
ENSMUSG00000048058	Ldlrad3	low density lipoprotein receptor class A domain containing 3 [Source:MGI Symbol;Acc:MGI:2138856]	1.634	3.91E-04
ENSMUSG00000018168	Ikzf3	IKAROS family zinc finger 3 [Source:MGI Symbol;Acc:MGI:1342542]	1.634	1.93E-02
ENSMUSG00000066392	Nrxn3	neurexin III [Source:MGI Symbol;Acc:MGI:1096389]	1.633	4.01E-03
ENSMUSG00000032353	Tmed3	transmembrane p24 trafficking protein 3 [Source:MGI Symbol;Acc:MGI:1913361]	1.632	4.65E-04
ENSMUSG00000065773	Rnu1b6	U1b6 small nuclear RNA [Source:MGI Symbol;Acc:MGI:104604]	1.632	2.14E-04
ENSMUSG00000106443	Gm42702	predicted gene 42702 [Source:MGI Symbol;Acc:MGI:5662839]	1.631	1.24E-03
ENSMUSG00000106341	Gm43330	predicted gene 43330 [Source:MGI Symbol;Acc:MGI:5663467]	1.631	4.04E-04
ENSMUSG00000045411	2410002F23Rik	RIKEN cDNA 2410002F23 gene [Source:MGI Symbol;Acc:MGI:1914226]	1.631	1.82E-06
ENSMUSG00000029657	Hsph1	heat shock 105kDa/110kDa protein 1 [Source:MGI Symbol;Acc:MGI:105053]	1.630	6.43E-05
ENSMUSG00000100826	Snhg14	small nucleolar RNA host gene 14 [Source:MGI Symbol;Acc:MGI:1289201]	1.630	3.16E-02
ENSMUSG00000021539	Lect2	leukocyte cell-derived chemotaxin 2 [Source:MGI Symbol;Acc:MGI:1278342]	1.629	2.81E-07
ENSMUSG00000109206	Gm45137	predicted gene 45137 [Source:MGI Symbol;Acc:MGI:5753713]	1.629	4.11E-04
ENSMUSG00000031387	Renbp	renin binding protein [Source:MGI Symbol;Acc:MGI:105940]	1.628	4.50E-02
ENSMUSG00000049866	Arl4c	ADP-ribosylation factor-like 4C [Source:MGI Symbol;Acc:MGI:2445172]	1.628	1.30E-02
ENSMUSG00000005686	Ampd3	adenosine monophosphate deaminase 3 [Source:MGI Symbol;Acc:MGI:1096344]	1.627	3.42E-02
ENSMUSG00000026848	Tor1b	"torsin family 1, member B [Source:MGI Symbol;Acc:MGI:1353605]"	1.627	1.10E-08

ENSMUSG00000039633	Lonrf1	LON peptidase N-terminal domain and ring finger 1 [Source:MGI Symbol;Acc:MGI:3609241]	1.627	1.03E-02
ENSMUSG00000102091	Olfr1034	olfactory receptor 1034 [Source:MGI Symbol;Acc:MGI:3030868]	1.626	1.58E-02
ENSMUSG00000042272	Sestd1	SEC14 and spectrin domains 1 [Source:MGI Symbol;Acc:MGI:1916262]	1.626	5.05E-03
ENSMUSG00000030279	C2cd5	C2 calcium-dependent domain containing 5 [Source:MGI Symbol;Acc:MGI:1921991]	1.626	8.62E-06
ENSMUSG00000039286	Fndc3b	fibronectin type III domain containing 3B [Source:MGI Symbol;Acc:MGI:1919257]	1.625	2.08E-07
ENSMUSG00000038930	Rccd1	RCC1 domain containing 1 [Source:MGI Symbol;Acc:MGI:2444156]	1.624	1.86E-02
ENSMUSG00000027087	Itgav	integrin alpha V [Source:MGI Symbol;Acc:MGI:96608]	1.624	5.57E-07
ENSMUSG00000052713	Zfp608	zinc finger protein 608 [Source:MGI Symbol;Acc:MGI:2442338]	1.624	3.89E-03
ENSMUSG00000001995	Sipa1l2	signal-induced proliferation-associated 1 like 2 [Source:MGI Symbol;Acc:MGI:2676970]	1.623	1.67E-04
ENSMUSG00000001774	Chordc1	"cysteine and histidine-rich domain (CHORD)-containing, zinc-binding protein 1 [Source:MGI Symbol;Acc:MGI:1914167]"	1.623	3.23E-04
ENSMUSG00000059173	Pde1a	"phosphodiesterase 1A, calmodulin-dependent [Source:MGI Symbol;Acc:MGI:1201792]"	1.622	8.81E-03
ENSMUSG00000057897	Camk2b	"calcium/calmodulin-dependent protein kinase II, beta [Source:MGI Symbol;Acc:MGI:88257]"	1.621	3.06E-02
ENSMUSG00000002109	Ddb2	damage specific DNA binding protein 2 [Source:MGI Symbol;Acc:MGI:1355314]	1.621	7.24E-03
ENSMUSG00000027799	Nbea	neurobeachin [Source:MGI Symbol;Acc:MGI:1347075]	1.619	4.54E-04
ENSMUSG00000036948	Map11	microtubule associated protein 11 [Source:MGI Symbol;Acc:MGI:2385896]	1.619	6.03E-04
ENSMUSG00000101587	Gm29036	predicted gene 29036 [Source:MGI Symbol;Acc:MGI:5579742]	1.618	3.09E-02
ENSMUSG00000025498	Irf7	interferon regulatory factor 7 [Source:MGI Symbol;Acc:MGI:1859212]	1.617	1.09E-03
ENSMUSG00000052248	Zeb2os	"zinc finger E-box binding homeobox 2, opposite strand [Source:MGI Symbol;Acc:MGI:3652108]"	1.616	2.46E-02
ENSMUSG00000026782	Abi2	abl-interactor 2 [Source:MGI Symbol;Acc:MGI:106913]	1.616	3.47E-03
ENSMUSG00000039738	Slx4	SLX4 structure-specific endonuclease subunit homolog (S. cerevisiae) [Source:MGI Symbol;Acc:MGI:106299]	1.615	3.62E-02
ENSMUSG00000096243	Gm24265	"predicted gene, 24265 [Source:MGI Symbol;Acc:MGI:5454042]"	1.615	2.04E-08
ENSMUSG00000029135	Fosl2	fos-like antigen 2 [Source:MGI Symbol;Acc:MGI:102858]	1.614	2.77E-04
ENSMUSG00000026499	Acbd3	acyl-Coenzyme A binding domain containing 3 [Source:MGI Symbol;Acc:MGI:2181074]	1.614	7.87E-08
ENSMUSG00000106993	Gm43417	predicted gene 43417 [Source:MGI Symbol;Acc:MGI:5663554]	1.614	4.71E-03
ENSMUSG00000103851	Gm37606	"predicted gene, 37606 [Source:MGI Symbol;Acc:MGI:5610834]"	1.614	2.03E-03
ENSMUSG00000021091	Serpina3n	"serine (or cysteine) peptidase inhibitor, clade A, member 3N [Source:MGI Symbol;Acc:MGI:105045]"	1.613	2.82E-05
ENSMUSG00000019960	Dusp6	dual specificity phosphatase 6 [Source:MGI Symbol;Acc:MGI:1914853]	1.612	6.60E-04
ENSMUSG00000032575	Manf	mesencephalic astrocyte-derived neurotrophic factor [Source:MGI Symbol;Acc:MGI:1922090]	1.612	7.00E-06
ENSMUSG00000023066	Rttn	rotatin [Source:MGI Symbol;Acc:MGI:2179288]	1.611	3.44E-02
ENSMUSG00000090877	Hspa1b	heat shock protein 1B [Source:MGI Symbol;Acc:MGI:99517]	1.611	7.03E-03
ENSMUSG00000076036	Gm22133	"predicted gene, 22133 [Source:MGI Symbol;Acc:MGI:5451910]"	1.611	1.69E-02
ENSMUSG00000073016	Uprt	uracil phosphoribosyltransferase [Source:MGI Symbol;Acc:MGI:2685620]	1.610	4.83E-02
ENSMUSG00000116796	Gm49784	"predicted gene, 49784 [Source:MGI Symbol;Acc:MGI:6215305]"	1.610	3.13E-02
ENSMUSG00000022178	Ajuba	ajuba LIM protein [Source:MGI Symbol;Acc:MGI:1341886]	1.610	1.07E-02
ENSMUSG00000026094	Stk17b	serine/threonine kinase 17b (apoptosis-inducing) [Source:MGI Symbol;Acc:MGI:2138162]	1.610	2.06E-03
ENSMUSG00000114749	Gm48442	"predicted gene, 48442 [Source:MGI Symbol;Acc:MGI:6097951]"	1.610	1.22E-03
ENSMUSG00000027506	Tpd52	tumor protein D52 [Source:MGI Symbol;Acc:MGI:107749]	1.609	2.77E-08
ENSMUSG00000036334	Igsf10	"immunoglobulin superfamily, member 10 [Source:MGI Symbol;Acc:MGI:1923481]"	1.608	2.50E-02
ENSMUSG00000094826	Gm23804	"predicted gene, 23804 [Source:MGI Symbol;Acc:MGI:5453581]"	1.608	2.98E-07
ENSMUSG00000039096	Rsad1	radical S-adenosyl methionine domain containing 1 [Source:MGI Symbol;Acc:MGI:3039628]	1.607	7.73E-03
ENSMUSG00000103614	Gm37949	"predicted gene, 37949 [Source:MGI Symbol;Acc:MGI:5611177]"	1.607	8.13E-03
ENSMUSG00000029298	Gbp9	guanylate-binding protein 9 [Source:MGI Symbol;Acc:MGI:3605620]	1.606	2.45E-04
ENSMUSG00000020400	Tnip1	TNFAIP3 interacting protein 1 [Source:MGI Symbol;Acc:MGI:1926194]	1.606	4.04E-03
ENSMUSG00000026074	Map4k4	mitogen-activated protein kinase kinase kinase kinase 4 [Source:MGI Symbol;Acc:MGI:1349394]	1.605	7.21E-03
ENSMUSG00000105148	Gm42700	predicted gene 42700 [Source:MGI Symbol;Acc:MGI:5662837]	1.604	1.15E-02
ENSMUSG00000105933	Gm43260	predicted gene 43260 [Source:MGI Symbol;Acc:MGI:5663397]	1.604	1.51E-03
ENSMUSG00000040177	2310057M21Rik	RIKEN cDNA 2310057M21 gene [Source:MGI Symbol;Acc:MGI:1915527]	1.603	6.97E-04
ENSMUSG00000102594	Gm38381	"predicted gene, 38381 [Source:MGI Symbol;Acc:MGI:5611609]"	1.602	6.40E-04
ENSMUSG00000027176	Cstf3	"cleavage stimulation factor, 3' pre-RNA, subunit 3 [Source:MGI Symbol;Acc:MGI:1351825]"	1.602	1.45E-04
ENSMUSG00000058873	Gm5582	predicted gene 5582 [Source:MGI Symbol;Acc:MGI:3643463]	1.602	4.07E-02

ENSMUSG00000015176	Nolc1	nucleolar and coiled-body phosphoprotein 1 [Source:MGI Symbol;Acc:MGI:1918019]	1.601	4.64E-03
ENSMUSG00000034774	Dsg1c	desmoglein 1 gamma [Source:MGI Symbol;Acc:MGI:2664358]	1.600	2.92E-04
ENSMUSG00000106063	C030032O16Rik	RIKEN cDNA C030032O16 gene [Source:MGI Symbol;Acc:MGI:1924718]	1.600	8.90E-04
ENSMUSG00000029860	Zyx	zyxin [Source:MGI Symbol;Acc:MGI:103072]	1.600	1.20E-03
ENSMUSG00000001248	Gramd1a	GRAM domain containing 1A [Source:MGI Symbol;Acc:MGI:105490]	1.600	1.39E-04
ENSMUSG00000020340	Cyfip2	cytoplasmic FMR1 interacting protein 2 [Source:MGI Symbol;Acc:MGI:1924134]	1.599	1.66E-02
ENSMUSG00000106636	Gm43813	predicted gene 43813 [Source:MGI Symbol;Acc:MGI:5663950]	1.599	3.77E-04
ENSMUSG00000054364	Rhob	ras homolog family member B [Source:MGI Symbol;Acc:MGI:107949]	1.599	4.83E-03
ENSMUSG00000040339	Fam102b	"family with sequence similarity 102, member B [Source:MGI Symbol;Acc:MGI:3036259]"	1.598	1.68E-03
ENSMUSG00000018983	E2f2	E2F transcription factor 2 [Source:MGI Symbol;Acc:MGI:1096341]	1.598	1.48E-02
ENSMUSG00000071337	Tia1	cytotoxic granule-associated RNA binding protein 1 [Source:MGI Symbol;Acc:MGI:107914]	1.596	3.98E-07
ENSMUSG00000057914	Cacnb2	"calcium channel, voltage-dependent, beta 2 subunit [Source:MGI Symbol;Acc:MGI:894644]"	1.596	3.32E-02
ENSMUSG00000038495	Otud7b	OTU domain containing 7B [Source:MGI Symbol;Acc:MGI:2654703]	1.595	5.16E-06
ENSMUSG00000022636	Alcam	activated leukocyte cell adhesion molecule [Source:MGI Symbol;Acc:MGI:1313266]	1.593	2.49E-05
ENSMUSG00000032555	Topbp1	topoisomerase (DNA) II binding protein 1 [Source:MGI Symbol;Acc:MGI:1920018]	1.592	4.27E-03
ENSMUSG00000058620	Adra2b	"adrenergic receptor, alpha 2b [Source:MGI Symbol;Acc:MGI:87935]"	1.591	1.93E-02
ENSMUSG00000033880	Lgals3bp	"lectin, galactoside-binding, soluble, 3 binding protein [Source:MGI Symbol;Acc:MGI:99554]"	1.591	1.00E-03
ENSMUSG00000025964	Adam23	a disintegrin and metallopeptidase domain 23 [Source:MGI Symbol;Acc:MGI:1345162]	1.590	9.52E-03
ENSMUSG00000027965	Olfm3	olfactomedin 3 [Source:MGI Symbol;Acc:MGI:2387329]	1.590	2.58E-02
ENSMUSG00000048897	Zfp710	zinc finger protein 710 [Source:MGI Symbol;Acc:MGI:1921747]	1.589	7.48E-03
ENSMUSG00000030505	Prmt3	protein arginine N-methyltransferase 3 [Source:MGI Symbol;Acc:MGI:1919224]	1.589	1.39E-03
ENSMUSG00000097277	2900076A07Rik	RIKEN cDNA 2900076A07 gene [Source:MGI Symbol;Acc:MGI:1920242]	1.588	1.70E-02
ENSMUSG00000030605	Mfge8	milk fat globule-EGF factor 8 protein [Source:MGI Symbol;Acc:MGI:102768]	1.587	2.94E-02
ENSMUSG00000033502	Cdc14a	CDC14 cell division cycle 14A [Source:MGI Symbol;Acc:MGI:2442676]	1.587	2.24E-02
ENSMUSG00000045328	Cenpe	centromere protein E [Source:MGI Symbol;Acc:MGI:1098230]	1.585	2.14E-02
ENSMUSG00000039456	Morc3	microrchidia 3 [Source:MGI Symbol;Acc:MGI:2136841]	1.585	5.18E-04
ENSMUSG00000060771	Tsga10	testis specific 10 [Source:MGI Symbol;Acc:MGI:2685063]	1.584	1.45E-02
ENSMUSG00000031015	Swap70	SWA-70 protein [Source:MGI Symbol;Acc:MGI:1298390]	1.580	2.06E-04
ENSMUSG00000096659	Gm25679	"predicted gene, 25679 [Source:MGI Symbol;Acc:MGI:5455456]"	1.580	7.24E-08
ENSMUSG00000009418	Nav1	neuron navigator 1 [Source:MGI Symbol;Acc:MGI:2183683]	1.580	2.33E-03
ENSMUSG00000024498	Tcerg1	transcription elongation regulator 1 (CA150) [Source:MGI Symbol;Acc:MGI:1926421]	1.579	1.85E-04
ENSMUSG00000003062	Stard3nl	STARD3 N-terminal like [Source:MGI Symbol;Acc:MGI:1923455]	1.578	3.32E-04
ENSMUSG00000046157	Tmem229b	transmembrane protein 229B [Source:MGI Symbol;Acc:MGI:2444389]	1.577	1.60E-02
ENSMUSG00000110077	Gm45632	predicted gene 45632 [Source:MGI Symbol;Acc:MGI:5791468]	1.576	4.11E-02
ENSMUSG00000075602	Ly6a	"lymphocyte antigen 6 complex, locus A [Source:MGI Symbol;Acc:MGI:107527]"	1.576	3.58E-02
ENSMUSG00000103572	Gm37115	"predicted gene, 37115 [Source:MGI Symbol;Acc:MGI:5610343]"	1.575	3.84E-03
ENSMUSG00000029190	D5Ertd579e	"DNA segment, Chr 5, ERATO Doi 579, expressed [Source:MGI Symbol;Acc:MGI:1261849]"	1.575	8.34E-06
ENSMUSG00000008373	Prpf31	pre-mRNA processing factor 31 [Source:MGI Symbol;Acc:MGI:1916238]	1.575	9.99E-03
ENSMUSG00000031608	Galnt7	polypeptide N-acetylgalactosaminyltransferase 7 [Source:MGI Symbol;Acc:MGI:1349449]	1.575	1.03E-02
ENSMUSG00000034617	Mtrr	5-methyltetrahydrofolate-homocysteine methyltransferase reductase [Source:MGI Symbol;Acc:MGI:1891037]	1.575	5.88E-03
ENSMUSG00000027423	Snx5	sorting nexin 5 [Source:MGI Symbol;Acc:MGI:1916428]	1.575	6.93E-05
ENSMUSG00000030729	Pgm2l1	phosphoglucomutase 2-like 1 [Source:MGI Symbol;Acc:MGI:1918224]	1.574	1.08E-03
ENSMUSG00000073982	Rhog	ras homolog family member G [Source:MGI Symbol;Acc:MGI:1928370]	1.574	2.83E-02
ENSMUSG00000054074	Skida1	SKI/DACH domain containing 1 [Source:MGI Symbol;Acc:MGI:1919918]	1.574	4.00E-02
ENSMUSG00000062373	Tmem65	transmembrane protein 65 [Source:MGI Symbol;Acc:MGI:1922118]	1.574	6.31E-05
ENSMUSG00000118434	Gm13301	predicted gene 13301 [Source:MGI Symbol;Acc:MGI:3707230]	1.574	3.61E-02
ENSMUSG00000014039	Prdm15	PR domain containing 15 [Source:MGI Symbol;Acc:MGI:1930121]	1.573	7.63E-03
ENSMUSG00000032409	Atr	ataxia telangiectasia and Rad3 related [Source:MGI Symbol;Acc:MGI:108028]	1.573	1.14E-03
ENSMUSG00000002233	Rhoc	ras homolog family member C [Source:MGI Symbol;Acc:MGI:106028]	1.573	1.07E-03
ENSMUSG00000030766	Arhgap17	Rho GTPase activating protein 17 [Source:MGI Symbol;Acc:MGI:1917747]	1.573	7.73E-06

ENSMUSG00000034641	Cd300ld	CD300 molecule like family member d [Source:MGI Symbol;Acc:MGI:2442358]	1.571	4.52E-03
ENSMUSG00000026222	Sp100	nuclear antigen Sp100 [Source:MGI Symbol;Acc:MGI:109561]	1.569	5.53E-03
ENSMUSG00000062944	9130023H24Rik	RIKEN cDNA 9130023H24 gene [Source:MGI Symbol;Acc:MGI:2442738]	1.569	7.28E-03
ENSMUSG00000033706	Smyd5	SET and MYND domain containing 5 [Source:MGI Symbol;Acc:MGI:108048]	1.569	1.57E-02
ENSMUSG00000114547	Gm3226	predicted gene 3226 [Source:MGI Symbol;Acc:MGI:3804968]	1.568	4.79E-02
ENSMUSG00000031502	Col4a1	"collagen, type IV, alpha 1 [Source:MGI Symbol;Acc:MGI:88454]"	1.567	5.46E-03
ENSMUSG00000052331	Ankrd44	ankyrin repeat domain 44 [Source:MGI Symbol;Acc:MGI:3045243]	1.567	1.37E-02
ENSMUSG00000109576	Gm44704	predicted gene 44704 [Source:MGI Symbol;Acc:MGI:5753280]	1.566	3.49E-02
ENSMUSG00000056515	Rab31	"RAB31, member RAS oncogene family [Source:MGI Symbol;Acc:MGI:1914603]"	1.565	2.82E-02
ENSMUSG00000086944	Gm15859	predicted gene 15859 [Source:MGI Symbol;Acc:MGI:3801904]	1.565	4.12E-02
ENSMUSG00000091337	Eid1	EP300 interacting inhibitor of differentiation 1 [Source:MGI Symbol;Acc:MGI:1889651]	1.565	3.63E-02
ENSMUSG00000078922	Tgtp1	T cell specific GTPase 1 [Source:MGI Symbol;Acc:MGI:98734]	1.564	1.98E-02
ENSMUSG00000007850	Hnrnph1	heterogeneous nuclear ribonucleoprotein H1 [Source:MGI Symbol;Acc:MGI:1891925]	1.564	1.76E-07
ENSMUSG00000106022	Gm42929	predicted gene 42929 [Source:MGI Symbol;Acc:MGI:5663066]	1.564	9.41E-03
ENSMUSG00000058152	Chsy3	chondroitin sulfate synthase 3 [Source:MGI Symbol;Acc:MGI:1926173]	1.563	1.32E-05
ENSMUSG00000109429	Gm45223	predicted gene 45223 [Source:MGI Symbol;Acc:MGI:5753799]	1.562	4.76E-05
ENSMUSG00000041734	Kirrel	kirre like nephrin family adhesion molecule 1 [Source:MGI Symbol;Acc:MGI:1891396]	1.562	6.84E-03
ENSMUSG00000041264	Uspl1	ubiquitin specific peptidase like 1 [Source:MGI Symbol;Acc:MGI:2442342]	1.562	5.68E-05
ENSMUSG00000093815	Gm26444	"predicted gene, 26444 [Source:MGI Symbol;Acc:MGI:5456221]"	1.561	7.57E-07
ENSMUSG00000102780	Gm38253	"predicted gene, 38253 [Source:MGI Symbol;Acc:MGI:5611481]"	1.561	1.37E-04
ENSMUSG00000031799	Tpm4	tropomyosin 4 [Source:MGI Symbol;Acc:MGI:2449202]	1.561	2.09E-03
ENSMUSG00000100394	Gm28791	predicted gene 28791 [Source:MGI Symbol;Acc:MGI:5579497]	1.560	8.75E-03
ENSMUSG00000106432	Gm43259	predicted gene 43259 [Source:MGI Symbol;Acc:MGI:5663396]	1.560	6.48E-04
ENSMUSG00000057346	Apol9a	apolipoprotein L 9a [Source:MGI Symbol;Acc:MGI:3606001]	1.559	1.86E-06
ENSMUSG00000109555	Gm44891	predicted gene 44891 [Source:MGI Symbol;Acc:MGI:5753467]	1.559	1.09E-03
ENSMUSG00000021108	Prkch	"protein kinase C, eta [Source:MGI Symbol;Acc:MGI:97600]"	1.557	2.04E-03
ENSMUSG00000020572	Nampt	nicotinamide phosphoribosyltransferase [Source:MGI Symbol;Acc:MGI:1929865]	1.556	1.49E-05
ENSMUSG00000005374	Tbl2	transducin (beta)-like 2 [Source:MGI Symbol;Acc:MGI:1351652]	1.556	5.36E-04
ENSMUSG00000063455	D630045J12Rik	RIKEN cDNA D630045J12 gene [Source:MGI Symbol;Acc:MGI:2669829]	1.556	2.42E-02
ENSMUSG00000056148	Rdh9	retinol dehydrogenase 9 [Source:MGI Symbol;Acc:MGI:2143528]	1.556	4.46E-09
ENSMUSG00000039531	Zup1	zinc finger containing ubiquitin peptidase 1 [Source:MGI Symbol;Acc:MGI:1919830]	1.556	8.79E-07
ENSMUSG00000103146	Gm37745	"predicted gene, 37745 [Source:MGI Symbol;Acc:MGI:5610973]"	1.555	9.52E-03
ENSMUSG00000113776	Gm19327	"predicted gene, 19327 [Source:MGI Symbol;Acc:MGI:5011512]"	1.555	8.76E-03
ENSMUSG00000032253	Phip	pleckstrin homology domain interacting protein [Source:MGI Symbol;Acc:MGI:1932404]	1.555	6.30E-06
ENSMUSG00000036667	Tcaf1	TRPM8 channel-associated factor 1 [Source:MGI Symbol;Acc:MGI:1914665]	1.555	2.26E-02
ENSMUSG00000038729	Pakap	paralemmin A kinase anchor protein [Source:MGI Symbol;Acc:MGI:5141924]	1.553	1.45E-05
ENSMUSG00000095738	Gm25313	"predicted gene, 25313 [Source:MGI Symbol;Acc:MGI:5455090]"	1.553	2.10E-03
ENSMUSG00000027018	Hat1	histone aminotransferase 1 [Source:MGI Symbol;Acc:MGI:96013]	1.551	3.72E-02
ENSMUSG00000109279	Gm45220	predicted gene 45220 [Source:MGI Symbol;Acc:MGI:5753796]	1.551	1.80E-04
ENSMUSG00000029304	Spp1	secreted phosphoprotein 1 [Source:MGI Symbol;Acc:MGI:98389]	1.551	3.06E-02
ENSMUSG00000025981	Coq10b	coenzyme Q10B [Source:MGI Symbol;Acc:MGI:1915126]	1.551	1.36E-03
ENSMUSG00000096349	Gm22513	"predicted gene, 22513 [Source:MGI Symbol;Acc:MGI:5452290]"	1.550	1.74E-07
ENSMUSG00000028496	Mllt3	"myeloid/lymphoid or mixed-lineage leukemia; translocated to, 3 [Source:MGI Symbol;Acc:MGI:1917372]"	1.550	5.31E-03
ENSMUSG00000104852	Gm43201	predicted gene 43201 [Source:MGI Symbol;Acc:MGI:5663338]	1.549	1.60E-03
ENSMUSG00000092341	Malat1	metastasis associated lung adenocarcinoma transcript 1 (non-coding RNA) [Source:MGI Symbol;Acc:MGI:1919539]	1.549	4.86E-07
ENSMUSG00000109812	Gm45640	predicted gene 45640 [Source:MGI Symbol;Acc:MGI:5791476]	1.549	1.66E-04
ENSMUSG00000097039	Pvt1	Pvt1 oncogene [Source:MGI Symbol;Acc:MGI:97824]	1.548	1.34E-02
ENSMUSG00000040441	Slc26a10	"solute carrier family 26, member 10 [Source:MGI Symbol;Acc:MGI:2143920]"	1.548	1.40E-02
ENSMUSG00000024807	Syvn1	"synovial apoptosis inhibitor 1, synoviolin [Source:MGI Symbol;Acc:MGI:1921376]"	1.548	2.41E-05
ENSMUSG00000019863	Qrsl1	glutaminyl-tRNA synthase (glutamine-hydrolyzing)-like 1 [Source:MGI Symbol;Acc:MGI:1923813]	1.548	2.32E-02

ENSMUSG00000020737	Jpt1	Jupiter microtubule associated homolog 1 [Source:MGI Symbol;Acc:MGI:1096361]	1.547	4.22E-02
ENSMUSG00000105412	Gm43768	predicted gene 43768 [Source:MGI Symbol;Acc:MGI:5663905]	1.546	1.42E-02
ENSMUSG00000025591	Tma16	translation machinery associated 16 [Source:MGI Symbol;Acc:MGI:1913532]	1.546	6.17E-03
ENSMUSG00000102205	9430092D12Rik	RIKEN cDNA 9430092D12 gene [Source:MGI Symbol;Acc:MGI:1924677]	1.546	4.18E-02
ENSMUSG00000045980	Tmem104	transmembrane protein 104 [Source:MGI Symbol;Acc:MGI:2444222]	1.544	1.80E-02
ENSMUSG00000046994	Mars2	methionine-tRNA synthetase 2 (mitochondrial) [Source:MGI Symbol;Acc:MGI:2444136]	1.543	1.02E-03
ENSMUSG00000108878	Gm49493	"predicted gene, 49493 [Source:MGI Symbol;Acc:MGI:6155171]"	1.543	3.87E-02
ENSMUSG00000105801	C430019N01Rik	RIKEN cDNA C430019N01 gene [Source:MGI Symbol;Acc:MGI:3028058]	1.543	9.49E-04
ENSMUSG00000028018	Gstcd	"glutathione S-transferase, C-terminal domain containing [Source:MGI Symbol;Acc:MGI:1914803]"	1.543	1.33E-02
ENSMUSG00000105199	Gm43581	predicted gene 43581 [Source:MGI Symbol;Acc:MGI:5663718]	1.542	1.69E-02
ENSMUSG00000004730	Adgre1	adhesion G protein-coupled receptor E1 [Source:MGI Symbol;Acc:MGI:106912]	1.542	7.08E-04
ENSMUSG00000039637	Coro7	coronin 7 [Source:MGI Symbol;Acc:MGI:1926135]	1.542	2.87E-03
ENSMUSG00000079036	Alkbh1	"alkB homolog 1, histone H2A dioxygenase [Source:MGI Symbol;Acc:MGI:2384034]"	1.541	4.54E-05
ENSMUSG00000106751	Gm42467	predicted gene 42467 [Source:MGI Symbol;Acc:MGI:5662604]	1.541	1.30E-02
ENSMUSG00000054099	Slc25a40	"solute carrier family 25, member 40 [Source:MGI Symbol;Acc:MGI:2442486]"	1.541	1.35E-02
ENSMUSG00000043467	Zbtb37	zinc finger and BTB domain containing 37 [Source:MGI Symbol;Acc:MGI:2444467]	1.540	5.10E-06
ENSMUSG00000043336	Filip1l	filamin A interacting protein 1-like [Source:MGI Symbol;Acc:MGI:1925999]	1.539	2.41E-02
ENSMUSG00000019699	Akt3	thymoma viral proto-oncogene 3 [Source:MGI Symbol;Acc:MGI:1345147]	1.538	1.39E-02
ENSMUSG00000019978	Epb41l2	erythrocyte membrane protein band 4.1 like 2 [Source:MGI Symbol;Acc:MGI:103009]	1.538	2.97E-04
ENSMUSG00000032553	Srprb	"signal recognition particle receptor, B subunit [Source:MGI Symbol;Acc:MGI:102964]"	1.538	8.46E-05
ENSMUSG00000108910	Gm44625	predicted gene 44625 [Source:MGI Symbol;Acc:MGI:5753201]	1.537	2.58E-02
ENSMUSG00000109154	Gm44822	predicted gene 44822 [Source:MGI Symbol;Acc:MGI:5753398]	1.536	1.71E-02
ENSMUSG00000055491	Pprc1	"peroxisome proliferative activated receptor, gamma, coactivator-related 1 [Source:MGI Symbol;Acc:MGI:2385096]"	1.536	8.26E-05
ENSMUSG00000103222	Gm37729	"predicted gene, 37729 [Source:MGI Symbol;Acc:MGI:5610957]"	1.536	4.31E-02
ENSMUSG00000031266	Gla	"galactosidase, alpha [Source:MGI Symbol;Acc:MGI:1347344]"	1.535	1.41E-02
ENSMUSG00000115044	Gm48978	"predicted gene, 48978 [Source:MGI Symbol;Acc:MGI:6118322]"	1.535	7.25E-03
ENSMUSG00000041324	Inhba	inhibin beta-A [Source:MGI Symbol;Acc:MGI:96570]	1.534	2.21E-06
ENSMUSG00000033004	Mycbp2	"MYC binding protein 2, E3 ubiquitin protein ligase [Source:MGI Symbol;Acc:MGI:2179432]"	1.534	2.59E-04
ENSMUSG00000029490	Mfsd7a	major facilitator superfamily domain containing 7A [Source:MGI Symbol;Acc:MGI:2442629]	1.533	3.45E-02
ENSMUSG00000040111	Gramd1b	GRAM domain containing 1B [Source:MGI Symbol;Acc:MGI:1925037]	1.533	8.69E-04
ENSMUSG00000040612	Ildr2	immunoglobulin-like domain containing receptor 2 [Source:MGI Symbol;Acc:MGI:1196370]	1.531	1.08E-07
ENSMUSG00000032300	1700017B05Rik	RIKEN cDNA 1700017B05 gene [Source:MGI Symbol;Acc:MGI:1921461]	1.531	6.44E-05
ENSMUSG00000025408	Ddit3	DNA-damage inducible transcript 3 [Source:MGI Symbol;Acc:MGI:109247]	1.531	1.51E-02
ENSMUSG00000103805	Gm37063	"predicted gene, 37063 [Source:MGI Symbol;Acc:MGI:5610291]"	1.530	9.60E-03
ENSMUSG00000032369	Plscr1	phospholipid scramblase 1 [Source:MGI Symbol;Acc:MGI:893575]	1.530	4.44E-03
ENSMUSG00000105293	Gm42843	predicted gene 42843 [Source:MGI Symbol;Acc:MGI:5662980]	1.529	2.27E-02
ENSMUSG00000038301	Snx10	sorting nexin 10 [Source:MGI Symbol;Acc:MGI:1919232]	1.529	3.72E-07
ENSMUSG00000113328	Gm47260	"predicted gene, 47260 [Source:MGI Symbol;Acc:MGI:6096093]"	1.528	4.30E-04
ENSMUSG00000024665	Fads2	fatty acid desaturase 2 [Source:MGI Symbol;Acc:MGI:1930079]	1.528	7.65E-11
ENSMUSG00000102732	Gm37342	"predicted gene, 37342 [Source:MGI Symbol;Acc:MGI:5610570]"	1.528	1.05E-02
ENSMUSG00000103373	Gm37238	"predicted gene, 37238 [Source:MGI Symbol;Acc:MGI:5610466]"	1.527	1.93E-04
ENSMUSG00000019916	P4ha1	"procollagen-proline, 2-oxoglutarate 4-dioxygenase (proline 4-hydroxylase), alpha 1 polypeptide [Source:MGI Symbol;Acc:MGI:97463]"	1.527	6.01E-03
ENSMUSG00000034744	Nagk	N-acetylglucosamine kinase [Source:MGI Symbol;Acc:MGI:1860418]	1.527	8.63E-03
ENSMUSG00000025132	Arhgdia	Rho GDP dissociation inhibitor (GDI) alpha [Source:MGI Symbol;Acc:MGI:2178103]	1.527	4.29E-04
ENSMUSG00000041798	Gck	glucokinase [Source:MGI Symbol;Acc:MGI:1270854]	1.526	4.69E-03
ENSMUSG00000074733	Zfp950	zinc finger protein 950 [Source:MGI Symbol;Acc:MGI:2652824]	1.526	3.88E-04
ENSMUSG00000002297	Dbf4	DBF4 zinc finger [Source:MGI Symbol;Acc:MGI:1351328]	1.525	1.58E-02
ENSMUSG00000024079	Eif2ak2	eukaryotic translation initiation factor 2-alpha kinase 2 [Source:MGI Symbol;Acc:MGI:1353449]	1.525	2.57E-05
ENSMUSG00000052534	Pbx1	pre B cell leukemia homeobox 1 [Source:MGI Symbol;Acc:MGI:97495]	1.525	3.00E-05
ENSMUSG00000031749	St3gal2	"ST3 beta-galactoside alpha-2,3-sialyltransferase 2 [Source:MGI Symbol;Acc:MGI:99427]"	1.524	2.62E-02

ENSMUSG00000031381	Piga	"phosphatidylinositol glycan anchor biosynthesis, class A [Source:MGI Symbol;Acc:MGI:99461]"	1.524	2.57E-04
ENSMUSG00000045392	Olfr1033	olfactory receptor 1033 [Source:MGI Symbol;Acc:MGI:3030867]	1.523	5.72E-05
ENSMUSG00000087396	4933407K13Rik	RIKEN cDNA 4933407K13 gene [Source:MGI Symbol;Acc:MGI:1921646]	1.523	2.11E-02
ENSMUSG00000022863	Btg3	BTG anti-proliferation factor 3 [Source:MGI Symbol;Acc:MGI:109532]	1.522	3.92E-02
ENSMUSG00000019943	Atp2b1	"ATPase, Ca++ transporting, plasma membrane 1 [Source:MGI Symbol;Acc:MGI:104653]"	1.522	3.89E-05
ENSMUSG00000106099	Gm42664	predicted gene 42664 [Source:MGI Symbol;Acc:MGI:5662801]	1.522	1.60E-05
ENSMUSG00000031557	Plekha2	"pleckstrin homology domain-containing, family A (phosphoinositide binding specific) member 2 [Source:MGI Symbol;Acc:MGI:1928144]"	1.520	1.15E-02
ENSMUSG00000094306	Gm24924	"predicted gene, 24924 [Source:MGI Symbol;Acc:MGI:5454701]"	1.519	1.92E-06
ENSMUSG00000030681	Mvp	major vault protein [Source:MGI Symbol;Acc:MGI:1925638]	1.519	1.18E-05
ENSMUSG00000102691	Gm37780	"predicted gene, 37780 [Source:MGI Symbol;Acc:MGI:5611008]"	1.518	2.66E-02
ENSMUSG00000029810	Tmem176b	transmembrane protein 176B [Source:MGI Symbol;Acc:MGI:1916348]	1.518	1.18E-06
ENSMUSG00000028249	Sdcbp	syndecan binding protein [Source:MGI Symbol;Acc:MGI:1337026]	1.518	1.71E-03
ENSMUSG00000074743	Thbd	thrombomodulin [Source:MGI Symbol;Acc:MGI:98736]	1.517	1.85E-02
ENSMUSG00000103657	Gm37204	"predicted gene, 37204 [Source:MGI Symbol;Acc:MGI:5610432]"	1.517	6.50E-05
ENSMUSG00000020899	Pfas	phosphoribosylformylglycinamidine synthase (FGAR amidotransferase) [Source:MGI Symbol;Acc:MGI:2684864]	1.515	3.31E-03
ENSMUSG00000104325	Gm37321	"predicted gene, 37321 [Source:MGI Symbol;Acc:MGI:5610549]"	1.514	1.61E-03
ENSMUSG00000109652	Gm45555	predicted gene 45555 [Source:MGI Symbol;Acc:MGI:5791391]	1.514	1.32E-04
ENSMUSG00000042292	Mrtfa	myocardin related transcription factor A [Source:MGI Symbol;Acc:MGI:2384495]	1.514	4.72E-02
ENSMUSG00000095616	Gm26244	"predicted gene, 26244 [Source:MGI Symbol;Acc:MGI:5456021]"	1.513	3.45E-06
ENSMUSG00000036896	C1qc	"complement component 1, q subcomponent, C chain [Source:MGI Symbol;Acc:MGI:88225]"	1.511	2.20E-03
ENSMUSG00000039318	Rab3gap2	RAB3 GTPase activating protein subunit 2 [Source:MGI Symbol;Acc:MGI:1916043]	1.511	4.25E-03
ENSMUSG00000042453	Reln	reelin [Source:MGI Symbol;Acc:MGI:103022]	1.510	2.17E-02
ENSMUSG00000021706	Zfyve16	"zinc finger, FYVE domain containing 16 [Source:MGI Symbol;Acc:MGI:2145181]"	1.510	1.59E-03
ENSMUSG00000006281	Tep1	telomerase associated protein 1 [Source:MGI Symbol;Acc:MGI:109573]	1.510	9.62E-04
ENSMUSG00000036552	Ermard	ER membrane associated RNA degradation [Source:MGI Symbol;Acc:MGI:1917317]	1.510	1.81E-02
ENSMUSG00000063727	Tnfrsf11b	"tumor necrosis factor receptor superfamily, member 11b (osteoprotegerin) [Source:MGI Symbol;Acc:MGI:109587]"	1.508	4.66E-02
ENSMUSG00000026135	Zfp142	zinc finger protein 142 [Source:MGI Symbol;Acc:MGI:1924514]	1.506	4.46E-04
ENSMUSG00000102573	Gm7265	predicted gene 7265 [Source:MGI Symbol;Acc:MGI:3648991]	1.505	1.53E-02
ENSMUSG00000025816	Sec61a2	"Sec61, alpha subunit 2 (S. cerevisiae) [Source:MGI Symbol;Acc:MGI:1931071]"	1.504	8.52E-03
ENSMUSG00000024097	Srsf7	serine and arginine-rich splicing factor 7 [Source:MGI Symbol;Acc:MGI:1926232]	1.503	3.35E-04
ENSMUSG00000020580	Rock2	Rho-associated coiled-coil containing protein kinase 2 [Source:MGI Symbol;Acc:MGI:107926]	1.503	8.90E-07
ENSMUSG00000101823	Gm29438	predicted gene 29438 [Source:MGI Symbol;Acc:MGI:5580144]	1.503	2.60E-02
ENSMUSG00000091542	Gm17167	predicted gene 17167 [Source:MGI Symbol;Acc:MGI:4937994]	1.503	7.70E-03
ENSMUSG00000021068	Nin	ninein [Source:MGI Symbol;Acc:MGI:105108]	1.503	9.59E-03
ENSMUSG00000052917	Senp7	SUMO1/sentrin specific peptidase 7 [Source:MGI Symbol;Acc:MGI:1913565]	1.502	1.81E-03
ENSMUSG00000026581	Sell	"selectin, lymphocyte [Source:MGI Symbol;Acc:MGI:98279]"	1.502	9.93E-03
ENSMUSG00000004508	Gab2	growth factor receptor bound protein 2-associated protein 2 [Source:MGI Symbol;Acc:MGI:1333854]	1.502	7.99E-03
ENSMUSG00000103697	Gm38020	"predicted gene, 38020 [Source:MGI Symbol;Acc:MGI:5611248]"	1.501	6.04E-04
ENSMUSG00000025647	Shisa5	shisa family member 5 [Source:MGI Symbol;Acc:MGI:1915044]	1.501	1.79E-02
ENSMUSG00000046111	Cep295	centrosomal protein 295 [Source:MGI Symbol;Acc:MGI:2442521]	1.500	6.15E-04
ENSMUSG00000062545	Tlr12	toll-like receptor 12 [Source:MGI Symbol;Acc:MGI:3045221]	1.500	2.32E-02
ENSMUSG00000009585	Apobec3	"apolipoprotein B mRNA editing enzyme, catalytic polypeptide 3 [Source:MGI Symbol;Acc:MGI:1933111]"	1.499	1.97E-02
ENSMUSG00000103821	D430013B06Rik	RIKEN cDNA D430013B06 gene [Source:MGI Symbol;Acc:MGI:3026994]	1.498	1.73E-02
ENSMUSG00000104469	Gm37663	"predicted gene, 37663 [Source:MGI Symbol;Acc:MGI:5610891]"	1.497	1.12E-03
ENSMUSG00000100313	Gm28323	predicted gene 28323 [Source:MGI Symbol;Acc:MGI:5579029]	1.496	2.83E-03
ENSMUSG00000026896	Ifih1	interferon induced with helicase C domain 1 [Source:MGI Symbol;Acc:MGI:1918836]	1.495	8.88E-04
ENSMUSG00000030403	Vasp	vasodilator-stimulated phosphoprotein [Source:MGI Symbol;Acc:MGI:109268]	1.493	1.39E-02
ENSMUSG00000020806	Rhbdf2	rhomboid 5 homolog 2 [Source:MGI Symbol;Acc:MGI:2442473]	1.492	7.52E-04
ENSMUSG00000029104	Htt	huntingtin [Source:MGI Symbol;Acc:MGI:96067]	1.492	2.32E-03
ENSMUSG00000115252	Gm48996	"predicted gene, 48996 [Source:MGI Symbol;Acc:MGI:6118344]"	1.490	5.74E-03

ENSMUSG00000050555	Hyls1	"HYLS1, centriolar and ciliogenesis associated [Source:MGI Symbol;Acc:MGI:1924082]"	1.489	3.31E-03
ENSMUSG00000046722	Cdc42se1	CDC42 small effector 1 [Source:MGI Symbol;Acc:MGI:1889510]	1.488	6.25E-05
ENSMUSG00000024772	Ehd1	EH-domain containing 1 [Source:MGI Symbol;Acc:MGI:1341878]	1.487	2.09E-05
ENSMUSG00000029415	Sdad1	SDA1 domain containing 1 [Source:MGI Symbol;Acc:MGI:2140779]	1.486	5.28E-03
ENSMUSG00000008384	Sertad1	SERTA domain containing 1 [Source:MGI Symbol;Acc:MGI:1913438]	1.486	4.08E-02
ENSMUSG00000021838	Samd4	sterile alpha motif domain containing 4 [Source:MGI Symbol;Acc:MGI:1921730]	1.485	5.33E-08
ENSMUSG00000025511	Tspan4	tetraspanin 4 [Source:MGI Symbol;Acc:MGI:1928097]	1.485	1.44E-04
ENSMUSG00000105137	Gm42869	predicted gene 42869 [Source:MGI Symbol;Acc:MGI:5663006]	1.484	9.93E-03
ENSMUSG00000069892	9930111J21Rik2	RIKEN cDNA 9930111J21 gene 2 [Source:MGI Symbol;Acc:MGI:3711310]	1.484	3.95E-02
ENSMUSG00000034321	Exosc1	exosome component 1 [Source:MGI Symbol;Acc:MGI:1913833]	1.483	3.47E-02
ENSMUSG00000074165	Zfp788	zinc finger protein 788 [Source:MGI Symbol;Acc:MGI:1914857]	1.482	2.47E-02
ENSMUSG00000082896	Gm5844	predicted gene 5844 [Source:MGI Symbol;Acc:MGI:3645252]	1.481	3.58E-02
ENSMUSG00000116624	Gm49706	"predicted gene, 49706 [Source:MGI Symbol;Acc:MGI:6215169]"	1.481	3.99E-02
ENSMUSG00000072889	Nfxl1	"nuclear transcription factor, X-box binding-like 1 [Source:MGI Symbol;Acc:MGI:1923646]"	1.480	1.12E-03
ENSMUSG00000025571	Tnrc6c	trinucleotide repeat containing 6C [Source:MGI Symbol;Acc:MGI:2443265]	1.480	4.27E-03
ENSMUSG00000104235	Gm37589	"predicted gene, 37589 [Source:MGI Symbol;Acc:MGI:5610817]"	1.480	7.02E-08
ENSMUSG00000108447	Gm44567	predicted gene 44567 [Source:MGI Symbol;Acc:MGI:5753143]	1.480	1.19E-02
ENSMUSG00000105970	Gm43360	predicted gene 43360 [Source:MGI Symbol;Acc:MGI:5663497]	1.479	1.70E-03
ENSMUSG00000026104	Stat1	signal transducer and activator of transcription 1 [Source:MGI Symbol;Acc:MGI:103063]	1.478	7.41E-05
ENSMUSG00000024590	Lmnb1	lamin B1 [Source:MGI Symbol;Acc:MGI:96795]	1.478	2.26E-02
ENSMUSG00000068011	Mkrn2os	"makorin, ring finger protein 2, opposite strand [Source:MGI Symbol;Acc:MGI:1917541]"	1.477	2.39E-02
ENSMUSG00000025041	Nt5c2	"5'-nucleotidase, cytosolic II [Source:MGI Symbol;Acc:MGI:2178563]"	1.476	1.86E-04
ENSMUSG00000003810	Mast2	microtubule associated serine/threonine kinase 2 [Source:MGI Symbol;Acc:MGI:894676]	1.476	5.02E-03
ENSMUSG00000002797	Ggct	gamma-glutamyl cyclotransferase [Source:MGI Symbol;Acc:MGI:95700]	1.475	1.49E-02
ENSMUSG00000107624	Gm44005	"predicted gene, 44005 [Source:MGI Symbol;Acc:MGI:5690397]"	1.475	2.61E-02
ENSMUSG00000039294	Cybc1	cytochrome b 245 chaperone 1 [Source:MGI Symbol;Acc:MGI:2384959]	1.475	1.22E-02
ENSMUSG00000028497	Hacd4	3-hydroxyacyl-CoA dehydratase 4 [Source:MGI Symbol;Acc:MGI:1914025]	1.474	6.35E-03
ENSMUSG00000021190	Lgmn	legumain [Source:MGI Symbol;Acc:MGI:1330838]	1.474	1.29E-05
ENSMUSG00000104164	Gm38248	"predicted gene, 38248 [Source:MGI Symbol;Acc:MGI:5611476]"	1.474	9.00E-03
ENSMUSG00000080717	B230307C23Rik	RIKEN cDNA B230307C23 gene [Source:MGI Symbol;Acc:MGI:3643396]	1.474	1.56E-03
ENSMUSG00000095098	Ccdc85b	coiled-coil domain containing 85B [Source:MGI Symbol;Acc:MGI:2147607]	1.474	2.51E-02
ENSMUSG00000027132	Katnbl1	katanin p80 subunit B like 1 [Source:MGI Symbol;Acc:MGI:1919675]	1.473	1.45E-02
ENSMUSG00000113326	Gm47586	"predicted gene, 47586 [Source:MGI Symbol;Acc:MGI:6096627]"	1.473	1.67E-03
ENSMUSG00000106904	Gm43029	predicted gene 43029 [Source:MGI Symbol;Acc:MGI:5663166]	1.472	1.06E-02
ENSMUSG00000065176	Rnu12	"RNA U12, small nuclear [Source:MGI Symbol;Acc:MGI:1336893]"	1.470	3.11E-04
ENSMUSG00000038648	Creb3l2	cAMP responsive element binding protein 3-like 2 [Source:MGI Symbol;Acc:MGI:2442695]	1.470	2.64E-04
ENSMUSG00000022560	Slc52a2	"solute carrier protein 52, member 2 [Source:MGI Symbol;Acc:MGI:1289288]"	1.470	7.50E-03
ENSMUSG00000082319	Actr3-ps	"actin related protein 3, pseudogene [Source:MGI Symbol;Acc:MGI:3648640]"	1.468	4.68E-03
ENSMUSG00000022893	Adamts1	"a disintegrin-like and metallopeptidase (reprolysin type) with thrombospondin type 1 motif, 1 [Source:MGI Symbol;Acc:MGI:109249]"	1.468	4.25E-03
ENSMUSG00000110751	C230053D17Rik	RIKEN cDNA C230053D17 gene [Source:MGI Symbol;Acc:MGI:2441895]	1.467	3.76E-02
ENSMUSG00000027381	Bcl2l11	BCL2-like 11 (apoptosis facilitator) [Source:MGI Symbol;Acc:MGI:1197519]	1.466	1.13E-03
ENSMUSG00000117679	Apbb3	"amyloid beta (A4) precursor protein-binding, family B, member 3 [Source:MGI Symbol;Acc:MGI:108404]"	1.464	2.06E-02
ENSMUSG00000102143	Gm38057	"predicted gene, 38057 [Source:MGI Symbol;Acc:MGI:5611285]"	1.464	5.96E-03
ENSMUSG00000002107	Celf2	"CUGBP, Elav-like family member 2 [Source:MGI Symbol;Acc:MGI:1338822]"	1.464	4.33E-03
ENSMUSG00000105655	Gm42659	predicted gene 42659 [Source:MGI Symbol;Acc:MGI:5662796]	1.462	3.63E-03
ENSMUSG00000101609	Kcnq1ot1	KCNQ1 overlapping transcript 1 [Source:MGI Symbol;Acc:MGI:1926855]	1.462	1.59E-04
ENSMUSG00000053477	Tcf4	transcription factor 4 [Source:MGI Symbol;Acc:MGI:98506]	1.461	8.12E-04
ENSMUSG00000108494	Gm45203	predicted gene 45203 [Source:MGI Symbol;Acc:MGI:5753779]	1.460	1.49E-02
ENSMUSG00000097207	6030443J06Rik	RIKEN cDNA 6030443J06 gene [Source:MGI Symbol;Acc:MGI:2444595]	1.460	3.93E-02
ENSMUSG00000032698	Lmo2	LIM domain only 2 [Source:MGI Symbol;Acc:MGI:102811]	1.459	1.42E-02

ENSMUSG00000020652	Cenpo	centromere protein O [Source:MGI Symbol;Acc:MGI:1923800]	1.459	2.05E-02
ENSMUSG00000015839	Nfe2l2	"nuclear factor, erythroid derived 2, like 2 [Source:MGI Symbol;Acc:MGI:108420]"	1.458	1.75E-04
ENSMUSG00000035258	Abi3bp	"ABI gene family, member 3 (NESH) binding protein [Source:MGI Symbol;Acc:MGI:2444583]"	1.458	1.41E-02
ENSMUSG00000001521	Tulp3	tubby-like protein 3 [Source:MGI Symbol;Acc:MGI:1329045]	1.455	3.23E-02
ENSMUSG00000104910	Gm43331	predicted gene 43331 [Source:MGI Symbol;Acc:MGI:5663468]	1.454	7.17E-03
ENSMUSG00000098439	Hm629797	cDNA sequence HM629797 [Source:MGI Symbol;Acc:MGI:5440479]	1.454	2.74E-02
ENSMUSG00000048537	Phldb1	"pleckstrin homology like domain, family B, member 1 [Source:MGI Symbol;Acc:MGI:2143230]"	1.452	2.46E-02
ENSMUSG00000054404	Slfn5	schlafen 5 [Source:MGI Symbol;Acc:MGI:1329004]	1.451	1.23E-02
ENSMUSG00000029389	Ddx55	DEAD (Asp-Glu-Ala-Asp) box polypeptide 55 [Source:MGI Symbol;Acc:MGI:1915098]	1.451	4.53E-02
ENSMUSG00000087177	E130307A14Rik	RIKEN cDNA E130307A14 gene [Source:MGI Symbol;Acc:MGI:3036287]	1.450	3.50E-03
ENSMUSG00000102939	Gm38111	"predicted gene, 38111 [Source:MGI Symbol;Acc:MGI:5611339]"	1.448	2.84E-02
ENSMUSG00000017132	Cyth1	cytohesin 1 [Source:MGI Symbol;Acc:MGI:1334257]	1.446	1.61E-02
ENSMUSG00000040282	BC052040	cDNA sequence BC052040 [Source:MGI Symbol;Acc:MGI:3026886]	1.446	3.58E-02
ENSMUSG00000038811	Gngt2	"guanine nucleotide binding protein (G protein), gamma transducing activity polypeptide 2 [Source:MGI Symbol;Acc:MGI:893584]"	1.446	1.63E-02
ENSMUSG00000032609	Klhdc8b	kelch domain containing 8B [Source:MGI Symbol;Acc:MGI:1925517]	1.445	4.06E-02
ENSMUSG00000031167	Rbm3	"RNA binding motif (RNP1, RRM) protein 3 [Source:MGI Symbol;Acc:MGI:1099460]"	1.445	6.05E-06
ENSMUSG00000103672	Gm37621	"predicted gene, 37621 [Source:MGI Symbol;Acc:MGI:5610849]"	1.443	2.07E-02
ENSMUSG00000029267	Mtf2	metal response element binding transcription factor 2 [Source:MGI Symbol;Acc:MGI:105050]	1.443	3.58E-02
ENSMUSG00000083899	Gm12346	predicted gene 12346 [Source:MGI Symbol;Acc:MGI:3649810]	1.443	2.45E-02
ENSMUSG00000035049	Rrp12	ribosomal RNA processing 12 homolog (S. cerevisiae) [Source:MGI Symbol;Acc:MGI:2147437]	1.442	3.97E-02
ENSMUSG00000034853	Acot11	acyl-CoA thioesterase 11 [Source:MGI Symbol;Acc:MGI:1913736]	1.442	1.48E-02
ENSMUSG00000105677	Gm43328	predicted gene 43328 [Source:MGI Symbol;Acc:MGI:5663465]	1.442	9.94E-03
ENSMUSG00000034422	Parp14	"poly (ADP-ribose) polymerase family, member 14 [Source:MGI Symbol;Acc:MGI:1919489]"	1.442	8.34E-05
ENSMUSG00000077323	Rnu11	U11 small nuclear RNA [Source:MGI Symbol;Acc:MGI:2148804]	1.441	2.74E-03
ENSMUSG00000103049	Gm37311	"predicted gene, 37311 [Source:MGI Symbol;Acc:MGI:5610539]"	1.441	5.53E-03
ENSMUSG00000039842	Mcph1	"microcephaly, primary autosomal recessive 1 [Source:MGI Symbol;Acc:MGI:2443308]"	1.438	8.33E-03
ENSMUSG00000030557	Mef2a	myocyte enhancer factor 2A [Source:MGI Symbol;Acc:MGI:99532]	1.438	4.60E-07
ENSMUSG00000061689	Dlgap4	DLG associated protein 4 [Source:MGI Symbol;Acc:MGI:2138865]	1.438	2.78E-03
ENSMUSG00000028995	Fam126a	"family with sequence similarity 126, member A [Source:MGI Symbol;Acc:MGI:2149839]"	1.437	3.47E-03
ENSMUSG00000024143	Rhoq	ras homolog family member Q [Source:MGI Symbol;Acc:MGI:1931553]	1.436	4.50E-04
ENSMUSG00000074071	Fam169b	"family with sequence similarity 169, member B [Source:MGI Symbol;Acc:MGI:3644026]"	1.435	5.92E-05
ENSMUSG00000038058	Nod1	nucleotide-binding oligomerization domain containing 1 [Source:MGI Symbol;Acc:MGI:1341839]	1.435	1.43E-02
ENSMUSG00000069089	Cdk7	cyclin-dependent kinase 7 [Source:MGI Symbol;Acc:MGI:102956]	1.435	1.84E-02
ENSMUSG00000105824	Gm43466	predicted gene 43466 [Source:MGI Symbol;Acc:MGI:5663603]	1.434	3.95E-02
ENSMUSG00000027536	Chmp4c	charged multivesicular body protein 4C [Source:MGI Symbol;Acc:MGI:1913621]	1.432	2.16E-02
ENSMUSG00000018800	Abca5	"ATP-binding cassette, sub-family A (ABC1), member 5 [Source:MGI Symbol;Acc:MGI:2386607]"	1.432	4.89E-03
ENSMUSG00000098374	Gm28043	"predicted gene, 28043 [Source:MGI Symbol;Acc:MGI:5547779]"	1.431	5.00E-03
ENSMUSG00000004317	Clcn5	"chloride channel, voltage-sensitive 5 [Source:MGI Symbol;Acc:MGI:99486]"	1.431	1.76E-04
ENSMUSG00000035898	Uba6	ubiquitin-like modifier activating enzyme 6 [Source:MGI Symbol;Acc:MGI:1913894]	1.431	6.89E-05
ENSMUSG00000004677	Myo9b	myosin IXb [Source:MGI Symbol;Acc:MGI:106624]	1.430	5.03E-08
ENSMUSG00000035725	Prkx	"protein kinase, X-linked [Source:MGI Symbol;Acc:MGI:1309999]"	1.430	2.50E-02
ENSMUSG00000069833	Ahnak	AHNAK nucleoprotein (desmoyokin) [Source:MGI Symbol;Acc:MGI:1316648]	1.430	5.10E-03
ENSMUSG00000030187	Klra2	"killer cell lectin-like receptor, subfamily A, member 2 [Source:MGI Symbol;Acc:MGI:101906]"	1.429	4.88E-02
ENSMUSG00000028195	Ccn1	cellular communication network factor 1 [Source:MGI Symbol;Acc:MGI:88613]	1.429	9.56E-03
ENSMUSG00000035021	Baz1a	bromodomain adjacent to zinc finger domain 1A [Source:MGI Symbol;Acc:MGI:1309478]	1.428	4.84E-03
ENSMUSG00000079108	Srp54c	signal recognition particle 54C [Source:MGI Symbol;Acc:MGI:3714359]	1.428	5.55E-05
ENSMUSG00000020869	Lrrc59	leucine rich repeat containing 59 [Source:MGI Symbol;Acc:MGI:2138133]	1.427	7.31E-07
ENSMUSG00000104569	Gm43054	predicted gene 43054 [Source:MGI Symbol;Acc:MGI:5663191]	1.427	4.74E-02
ENSMUSG00000007041	Clic1	chloride intracellular channel 1 [Source:MGI Symbol;Acc:MGI:2148924]	1.426	1.13E-02
ENSMUSG00000088185	Scarna2	small Cajal body-specific RNA 2 [Source:MGI Symbol;Acc:MGI:3819484]	1.426	1.10E-03

ENSMUSG00000053819	Camk2d	"calcium/calmodulin-dependent protein kinase II, delta [Source:MGI Symbol;Acc:MGI:1341265]"	1.425	5.26E-05
ENSMUSG00000040296	Ddx58	DEAD (Asp-Glu-Ala-Asp) box polypeptide 58 [Source:MGI Symbol;Acc:MGI:2442858]	1.424	3.27E-04
ENSMUSG00000054520	Sh3bp2	SH3-domain binding protein 2 [Source:MGI Symbol;Acc:MGI:1346349]	1.424	8.02E-03
ENSMUSG00000037922	Bank1	B cell scaffold protein with ankyrin repeats 1 [Source:MGI Symbol;Acc:MGI:2442120]	1.424	6.77E-03
ENSMUSG00000061607	Mdc1	mediator of DNA damage checkpoint 1 [Source:MGI Symbol;Acc:MGI:3525201]	1.423	1.30E-03
ENSMUSG00000031503	Col4a2	"collagen, type IV, alpha 2 [Source:MGI Symbol;Acc:MGI:88455]"	1.422	2.00E-02
ENSMUSG00000087174	5530601H04Rik	RIKEN cDNA 5530601H04 gene [Source:MGI Symbol;Acc:MGI:1918695]	1.422	4.38E-02
ENSMUSG00000112449	Srp54b	signal recognition particle 54B [Source:MGI Symbol;Acc:MGI:3714357]	1.422	1.63E-04
ENSMUSG00000033021	Gmppa	GDP-mannose pyrophosphorylase A [Source:MGI Symbol;Acc:MGI:1916330]	1.421	4.18E-02
ENSMUSG00000078495	Zfp984	zinc finger protein 984 [Source:MGI Symbol;Acc:MGI:3651978]	1.421	2.03E-02
ENSMUSG00000105761	Gm43787	predicted gene 43787 [Source:MGI Symbol;Acc:MGI:5663924]	1.420	1.66E-02
ENSMUSG00000104676	Gm42777	predicted gene 42777 [Source:MGI Symbol;Acc:MGI:5662914]	1.419	4.90E-02
ENSMUSG00000108238	Gm43984	"predicted gene, 43984 [Source:MGI Symbol;Acc:MGI:5690376]"	1.419	5.85E-04
ENSMUSG00000022858	Tra2b	transformer 2 beta [Source:MGI Symbol;Acc:MGI:106016]	1.419	1.04E-06
ENSMUSG00000037820	Tgm2	"transglutaminase 2, C polypeptide [Source:MGI Symbol;Acc:MGI:98731]"	1.418	3.06E-05
ENSMUSG00000103525	Gm37262	"predicted gene, 37262 [Source:MGI Symbol;Acc:MGI:5610490]"	1.417	1.36E-02
ENSMUSG00000024556	Me2	"malic enzyme 2, NAD(+)-dependent, mitochondrial [Source:MGI Symbol;Acc:MGI:2147351]"	1.417	4.88E-02
ENSMUSG00000073274	Gm14636	predicted gene 14636 [Source:MGI Symbol;Acc:MGI:3641976]	1.416	5.65E-04
ENSMUSG00000113432	8430406P12Rik	RIKEN cDNA 8430406P12 gene [Source:MGI Symbol;Acc:MGI:1918744]	1.415	4.94E-02
ENSMUSG00000018381	Abi3	"ABI gene family, member 3 [Source:MGI Symbol;Acc:MGI:1913860]"	1.415	2.43E-02
ENSMUSG00000073079	Srp54a	signal recognition particle 54A [Source:MGI Symbol;Acc:MGI:1346087]	1.415	1.03E-04
ENSMUSG00000033991	Ttc37	tetratricopeptide repeat domain 37 [Source:MGI Symbol;Acc:MGI:2679923]	1.415	6.64E-03
ENSMUSG00000105703	Gm43305	predicted gene 43305 [Source:MGI Symbol;Acc:MGI:5663442]	1.414	1.60E-02
ENSMUSG00000030847	Bag3	BCL2-associated athanogene 3 [Source:MGI Symbol;Acc:MGI:1352493]	1.414	1.46E-03
ENSMUSG00000039753	Fbxl5	F-box and leucine-rich repeat protein 5 [Source:MGI Symbol;Acc:MGI:2152883]	1.414	1.05E-04
ENSMUSG00000106940	Gm42930	predicted gene 42930 [Source:MGI Symbol;Acc:MGI:5663067]	1.413	3.83E-02
ENSMUSG00000002658	Gtf2f1	"general transcription factor IIF, polypeptide 1 [Source:MGI Symbol;Acc:MGI:1923848]"	1.413	2.69E-02
ENSMUSG00000029817	Tra2a	transformer 2 alpha [Source:MGI Symbol;Acc:MGI:1933972]	1.413	3.64E-06
ENSMUSG00000000957	Mmp14	matrix metallopeptidase 14 (membrane-inserted) [Source:MGI Symbol;Acc:MGI:101900]	1.412	1.30E-04
ENSMUSG00000103976	Gm37677	"predicted gene, 37677 [Source:MGI Symbol;Acc:MGI:5610905]"	1.412	1.08E-03
ENSMUSG00000066800	Rnasel	"ribonuclease L (2', 5'-oligoisoadenylate synthetase-dependent) [Source:MGI Symbol;Acc:MGI:1098272]"	1.412	1.34E-02
ENSMUSG00000109096	Gm44888	predicted gene 44888 [Source:MGI Symbol;Acc:MGI:5753464]	1.411	3.04E-03
ENSMUSG00000009035	Tmem184b	transmembrane protein 184b [Source:MGI Symbol;Acc:MGI:2445179]	1.411	1.15E-03
ENSMUSG00000037440	Vnn1	vanin 1 [Source:MGI Symbol;Acc:MGI:108395]	1.411	1.36E-02
ENSMUSG00000037251	Pomk	protein-O-mannose kinase [Source:MGI Symbol;Acc:MGI:1921903]	1.411	9.24E-03
ENSMUSG00000031422	Morf4l2	mortality factor 4 like 2 [Source:MGI Symbol;Acc:MGI:1927167]	1.410	1.78E-04
ENSMUSG00000019961	Tmpo	thymopoietin [Source:MGI Symbol;Acc:MGI:106920]	1.410	2.19E-06
ENSMUSG00000030609	Aen	apoptosis enhancing nuclease [Source:MGI Symbol;Acc:MGI:1915298]	1.410	1.40E-02
ENSMUSG00000104583	Gm42701	predicted gene 42701 [Source:MGI Symbol;Acc:MGI:5662838]	1.410	1.63E-02
ENSMUSG00000114605	C130051F05Rik	RIKEN cDNA C130051F05 gene [Source:MGI Symbol;Acc:MGI:2443856]	1.409	2.17E-02
ENSMUSG00000072568	Lratd2	LRAT domain containing 1 [Source:MGI Symbol;Acc:MGI:3026924]	1.409	1.04E-02
ENSMUSG00000074649	BC029722	cDNA sequence BC029722 [Source:MGI Symbol;Acc:MGI:3584273]	1.409	5.59E-03
ENSMUSG00000023249	Parp3	"poly (ADP-ribose) polymerase family, member 3 [Source:MGI Symbol;Acc:MGI:1891258]"	1.409	7.96E-04
ENSMUSG00000068566	Myadm	myeloid-associated differentiation marker [Source:MGI Symbol;Acc:MGI:1355332]	1.408	2.80E-02
ENSMUSG00000048832	Vps37c	vacuolar protein sorting 37C [Source:MGI Symbol;Acc:MGI:2147661]	1.408	1.11E-02
ENSMUSG00000003363	Pld3	"phospholipase D family, member 3 [Source:MGI Symbol;Acc:MGI:1333782]"	1.408	4.70E-04
ENSMUSG00000105861	Gm43508	predicted gene 43508 [Source:MGI Symbol;Acc:MGI:5663645]	1.407	2.37E-02
ENSMUSG00000023277	Twf2	twinfilin actin binding protein 2 [Source:MGI Symbol;Acc:MGI:1346078]	1.407	2.17E-02
ENSMUSG00000024109	Nrxn1	neurexin I [Source:MGI Symbol;Acc:MGI:1096391]	1.407	4.30E-03
ENSMUSG00000027405	Nop56	NOP56 ribonucleoprotein [Source:MGI Symbol;Acc:MGI:1914384]	1.406	1.13E-02

ENSMUSG00000103937	Gm37186	"predicted gene, 37186 [Source:MGI Symbol;Acc:MGI:5610414]"	1.405	1.97E-05
ENSMUSG00000033364	Usp37	ubiquitin specific peptidase 37 [Source:MGI Symbol;Acc:MGI:2442483]	1.404	7.51E-04
ENSMUSG00000032279	Idh3a	isocitrate dehydrogenase 3 (NAD+) alpha [Source:MGI Symbol;Acc:MGI:1915084]	1.404	4.40E-04
ENSMUSG00000097375	6720427I07Rik	RIKEN cDNA 6720427I07 gene [Source:MGI Symbol;Acc:MGI:1924398]	1.404	3.12E-03
ENSMUSG00000103507	Gm38375	"predicted gene, 38375 [Source:MGI Symbol;Acc:MGI:5611603]"	1.403	2.55E-02
ENSMUSG00000115624	Gm49204	"predicted gene, 49204 [Source:MGI Symbol;Acc:MGI:6118653]"	1.403	2.69E-05
ENSMUSG00000049047	Armcx3	"armadillo repeat containing, X-linked 3 [Source:MGI Symbol;Acc:MGI:1918953]"	1.403	1.17E-03
ENSMUSG00000027610	Gss	glutathione synthetase [Source:MGI Symbol;Acc:MGI:95852]	1.402	2.07E-04
ENSMUSG00000025283	Sat1	spermidine/spermine N1-acetyl transferase 1 [Source:MGI Symbol;Acc:MGI:98233]	1.402	2.69E-06
ENSMUSG00000059182	Skap2	src family associated phosphoprotein 2 [Source:MGI Symbol;Acc:MGI:1889206]	1.402	3.14E-07
ENSMUSG00000035868	Zfp983	zinc finger protein 983 [Source:MGI Symbol;Acc:MGI:1920479]	1.402	3.21E-02
ENSMUSG00000016087	Fli1	Friend leukemia integration 1 [Source:MGI Symbol;Acc:MGI:95554]	1.402	3.14E-02
ENSMUSG00000019891	Dcbld1	"discoidin, CUB and LCCL domain containing 1 [Source:MGI Symbol;Acc:MGI:1913936]"	1.401	2.06E-03
ENSMUSG00000104125	Gm37488	"predicted gene, 37488 [Source:MGI Symbol;Acc:MGI:5610716]"	1.401	7.20E-04
ENSMUSG00000027281	Slx4ip	SLX4 interacting protein [Source:MGI Symbol;Acc:MGI:1921493]	1.400	1.78E-02
ENSMUSG00000101970	1810026B05Rik	RIKEN cDNA 1810026B05 gene [Source:MGI Symbol;Acc:MGI:1916420]	1.400	6.82E-03
ENSMUSG00000021036	Sptlc2	"serine palmitoyltransferase, long chain base subunit 2 [Source:MGI Symbol;Acc:MGI:108074]"	1.400	6.77E-04
ENSMUSG00000020601	Trib2	tribbles pseudokinase 2 [Source:MGI Symbol;Acc:MGI:2145021]	1.399	2.36E-02
ENSMUSG00000049932	H2ax	H2A.X variant histone [Source:MGI Symbol;Acc:MGI:102688]	1.399	3.40E-02
ENSMUSG00000042492	Tbc1d10b	"TBC1 domain family, member 10b [Source:MGI Symbol;Acc:MGI:1915699]"	1.399	3.89E-02
ENSMUSG00000033166	Dis3	"DIS3 homolog, exosome endoribonuclease and 3'-5' exoribonuclease [Source:MGI Symbol;Acc:MGI:1919912]"	1.398	3.05E-02
ENSMUSG00000022364	Tbc1d31	"TBC1 domain family, member 31 [Source:MGI Symbol;Acc:MGI:2684931]"	1.397	2.66E-02
ENSMUSG00000041057	Wdr43	WD repeat domain 43 [Source:MGI Symbol;Acc:MGI:1919765]	1.397	1.27E-02
ENSMUSG00000000440	Pparg	peroxisome proliferator activated receptor gamma [Source:MGI Symbol;Acc:MGI:97747]	1.396	1.93E-02
ENSMUSG00000023927	Satb1	special AT-rich sequence binding protein 1 [Source:MGI Symbol;Acc:MGI:105084]	1.396	4.08E-02
ENSMUSG00000106073	Gm42892	predicted gene 42892 [Source:MGI Symbol;Acc:MGI:5663029]	1.395	8.28E-04
ENSMUSG00000033799	Tasor2	transcription activation suppressor family member 2 [Source:MGI Symbol;Acc:MGI:2145274]	1.395	3.11E-03
ENSMUSG00000055485	Soga1	"suppressor of glucose, autophagy associated 1 [Source:MGI Symbol;Acc:MGI:2444575]"	1.395	3.92E-02
ENSMUSG00000021811	Dnajc9	DnaJ heat shock protein family (Hsp40) member C9 [Source:MGI Symbol;Acc:MGI:1915326]	1.393	3.58E-02
ENSMUSG00000029920	Smarcad1	"SWI/SNF-related, matrix-associated actin-dependent regulator of chromatin, subfamily a, containing DEAD/H box 1 [Source:MGI Symbol;Acc:MGI:95453]"	1.393	2.39E-02
ENSMUSG00000052406	Rexo4	"REX4, 3'-5' exonuclease [Source:MGI Symbol;Acc:MGI:2684957]"	1.393	4.22E-02
ENSMUSG00000040586	Ofd1	"OFD1, centriole and centriolar satellite protein [Source:MGI Symbol;Acc:MGI:1350328]"	1.393	3.99E-02
ENSMUSG00000029763	Exoc4	exocyst complex component 4 [Source:MGI Symbol;Acc:MGI:1096376]	1.392	7.73E-05
ENSMUSG00000108268	Gm44187	"predicted gene, 44187 [Source:MGI Symbol;Acc:MGI:5690579]"	1.390	1.17E-03
ENSMUSG00000073902	Gm1966	predicted gene 1966 [Source:MGI Symbol;Acc:MGI:3584360]	1.388	3.88E-03
ENSMUSG00000002885	Adgre5	adhesion G protein-coupled receptor E5 [Source:MGI Symbol;Acc:MGI:1347095]	1.388	3.35E-02
ENSMUSG00000103696	Gm37531	"predicted gene, 37531 [Source:MGI Symbol;Acc:MGI:5610759]"	1.387	1.38E-02
ENSMUSG00000047180	Neurl3	neuralized E3 ubiquitin protein ligase 3 [Source:MGI Symbol;Acc:MGI:2429944]	1.386	4.00E-02
ENSMUSG00000037376	Trmt6	tRNA methyltransferase 6 [Source:MGI Symbol;Acc:MGI:1914176]	1.386	4.67E-02
ENSMUSG00000105572	Gm43300	predicted gene 43300 [Source:MGI Symbol;Acc:MGI:5663437]	1.385	1.25E-03
ENSMUSG00000034610	Tut4	terminal uridylyl transferase 4 [Source:MGI Symbol;Acc:MGI:2445126]	1.385	1.53E-02
ENSMUSG00000107362	Gm40309	"predicted gene, 40309 [Source:MGI Symbol;Acc:MGI:5623194]"	1.385	1.53E-04
ENSMUSG00000040297	Suco	SUN domain containing ossification factor [Source:MGI Symbol;Acc:MGI:2138346]	1.385	1.93E-02
ENSMUSG00000037847	Nmrk1	nicotinamide riboside kinase 1 [Source:MGI Symbol;Acc:MGI:2147434]	1.384	2.06E-02
ENSMUSG00000105804	Gm43654	predicted gene 43654 [Source:MGI Symbol;Acc:MGI:5663791]	1.383	1.71E-03
ENSMUSG00000027663	Zmat3	zinc finger matrin type 3 [Source:MGI Symbol;Acc:MGI:1195270]	1.383	4.25E-03
ENSMUSG00000022505	Emp2	epithelial membrane protein 2 [Source:MGI Symbol;Acc:MGI:1098726]	1.383	2.80E-02
ENSMUSG00000100455	Gm29170	predicted gene 29170 [Source:MGI Symbol;Acc:MGI:5579876]	1.383	3.18E-02
ENSMUSG00000028163	Nfkb1	"nuclear factor of kappa light polypeptide gene enhancer in B cells 1, p105 [Source:MGI Symbol;Acc:MGI:97312]"	1.381	1.24E-05
ENSMUSG00000060935	Tmem263	transmembrane protein 263 [Source:MGI Symbol;Acc:MGI:2143652]	1.381	3.14E-03

ENSMUSG00000114598	D130062J10Rik	RIKEN cDNA D130062J10 gene [Source:MGI Symbol;Acc:MGI:2441949]	1.381	1.97E-02
ENSMUSG00000028063	Lmna	lamin A [Source:MGI Symbol;Acc:MGI:96794]	1.380	1.47E-03
ENSMUSG00000032688	Malt1	MALT1 paracaspase [Source:MGI Symbol;Acc:MGI:2445027]	1.379	1.94E-03
ENSMUSG00000025269	Apex2	apurinic/apyrimidinic endonuclease 2 [Source:MGI Symbol;Acc:MGI:1924872]	1.378	1.38E-02
ENSMUSG00000107118	Gm42986	predicted gene 42986 [Source:MGI Symbol;Acc:MGI:5663123]	1.378	3.66E-04
ENSMUSG00000071076	Jund	jun D proto-oncogene [Source:MGI Symbol;Acc:MGI:96648]	1.378	6.38E-03
ENSMUSG00000031570	Plpp5	phospholipid phosphatase 5 [Source:MGI Symbol;Acc:MGI:1919160]	1.377	6.17E-05
ENSMUSG00000078853	Igtp	interferon gamma induced GTPase [Source:MGI Symbol;Acc:MGI:107729]	1.377	1.59E-02
ENSMUSG00000020189	Osbpl8	oxysterol binding protein-like 8 [Source:MGI Symbol;Acc:MGI:2443807]	1.377	2.79E-06
ENSMUSG00000037965	Zc3h7a	zinc finger CCCH type containing 7 A [Source:MGI Symbol;Acc:MGI:2445044]	1.375	7.43E-08
ENSMUSG00000028034	Fubp1	far upstream element (FUSE) binding protein 1 [Source:MGI Symbol;Acc:MGI:1196294]	1.375	4.72E-05
ENSMUSG00000098188	Sowahc	sosondowah ankyrin repeat domain family member C [Source:MGI Symbol;Acc:MGI:3606051]	1.374	7.40E-03
ENSMUSG00000032525	Nktr	natural killer tumor recognition sequence [Source:MGI Symbol;Acc:MGI:97346]	1.374	7.77E-08
ENSMUSG00000073557	Ppp1r12b	"protein phosphatase 1, regulatory subunit 12B [Source:MGI Symbol;Acc:MGI:1916417]"	1.373	7.69E-04
ENSMUSG00000033909	Usp36	ubiquitin specific peptidase 36 [Source:MGI Symbol;Acc:MGI:1919594]	1.373	1.64E-02
ENSMUSG00000031360	Ctps2	cytidine 5'-triphosphate synthase 2 [Source:MGI Symbol;Acc:MGI:1933185]	1.373	3.20E-02
ENSMUSG00000113650	Gm47826	"predicted gene, 47826 [Source:MGI Symbol;Acc:MGI:6097020]"	1.372	9.31E-03
ENSMUSG00000035561	Aldh1b1	"aldehyde dehydrogenase 1 family, member B1 [Source:MGI Symbol;Acc:MGI:1919785]"	1.371	3.10E-03
ENSMUSG00000008035	Mid1ip1	Mid1 interacting protein 1 (gastrulation specific G12-like (zebrafish)) [Source:MGI Symbol;Acc:MGI:1915291]	1.371	8.60E-03
ENSMUSG00000022443	Myh9	"myosin, heavy polypeptide 9, non-muscle [Source:MGI Symbol;Acc:MGI:107717]"	1.371	1.11E-05
ENSMUSG00000023043	Krt18	keratin 18 [Source:MGI Symbol;Acc:MGI:96692]	1.370	4.05E-04
ENSMUSG00000024955	Esrra	"estrogen related receptor, alpha [Source:MGI Symbol;Acc:MGI:1346831]"	1.370	4.92E-04
ENSMUSG00000115276	9930017N22Rik	RIKEN cDNA 9930017N22 gene [Source:MGI Symbol;Acc:MGI:2443961]	1.370	2.52E-03
ENSMUSG00000024423	Impact	"impact, RWD domain protein [Source:MGI Symbol;Acc:MGI:1098233]"	1.369	1.53E-02
ENSMUSG00000037225	Fgf2	fibroblast growth factor 2 [Source:MGI Symbol;Acc:MGI:95516]	1.369	4.69E-02
ENSMUSG00000026014	Raph1	Ras association (RalGDS/AF-6) and pleckstrin homology domains 1 [Source:MGI Symbol;Acc:MGI:1924550]	1.368	7.06E-04
ENSMUSG00000105597	4633401B06Rik	RIKEN cDNA 4633401B06 gene [Source:MGI Symbol;Acc:MGI:1918078]	1.367	3.46E-02
ENSMUSG00000017561	Crlf3	cytokine receptor-like factor 3 [Source:MGI Symbol;Acc:MGI:1860086]	1.366	3.55E-02
ENSMUSG00000054693	Adam10	a disintegrin and metallopeptidase domain 10 [Source:MGI Symbol;Acc:MGI:109548]	1.366	1.31E-03
ENSMUSG00000049553	Polr1a	polymerase (RNA) I polypeptide A [Source:MGI Symbol;Acc:MGI:1096397]	1.366	3.50E-02
ENSMUSG00000048668	Rhno1	RAD9-HUS1-RAD1 interacting nuclear orphan 1 [Source:MGI Symbol;Acc:MGI:1915315]	1.366	3.69E-02
ENSMUSG00000024054	Smchd1	SMC hinge domain containing 1 [Source:MGI Symbol;Acc:MGI:1921605]	1.364	3.05E-04
ENSMUSG00000033790	Tubgcp5	"tubulin, gamma complex associated protein 5 [Source:MGI Symbol;Acc:MGI:2178836]"	1.364	4.12E-02
ENSMUSG00000031790	Mmp15	matrix metallopeptidase 15 [Source:MGI Symbol;Acc:MGI:109320]	1.363	9.62E-03
ENSMUSG00000079084	Ccdc82	coiled-coil domain containing 82 [Source:MGI Symbol;Acc:MGI:1913646]	1.362	5.16E-03
ENSMUSG00000022718	Dgcr8	"DGCR8, microprocessor complex subunit [Source:MGI Symbol;Acc:MGI:2151114]"	1.361	2.83E-02
ENSMUSG00000104094	Gm37314	"predicted gene, 37314 [Source:MGI Symbol;Acc:MGI:5610542]"	1.360	1.58E-02
ENSMUSG00000054737	Zfp182	zinc finger protein 182 [Source:MGI Symbol;Acc:MGI:2442220]	1.360	7.27E-03
ENSMUSG00000025040	Fundc1	FUN14 domain containing 1 [Source:MGI Symbol;Acc:MGI:1919268]	1.360	2.71E-02
ENSMUSG00000032840	2410131K14Rik	RIKEN cDNA 2410131K14 gene [Source:MGI Symbol;Acc:MGI:1924042]	1.360	2.55E-02
ENSMUSG00000024462	Gabbr1	"gamma-aminobutyric acid (GABA) B receptor, 1 [Source:MGI Symbol;Acc:MGI:1860139]"	1.360	4.58E-02
ENSMUSG00000112090	Gm48231	"predicted gene, 48231 [Source:MGI Symbol;Acc:MGI:6097636]"	1.359	3.41E-02
ENSMUSG00000034371	Tkfc	"triokinase, FMN cyclase [Source:MGI Symbol;Acc:MGI:2385084]"	1.359	7.56E-04
ENSMUSG00000020641	Rsad2	radical S-adenosyl methionine domain containing 2 [Source:MGI Symbol;Acc:MGI:1929628]	1.359	1.96E-02
ENSMUSG00000038299	Wdr36	WD repeat domain 36 [Source:MGI Symbol;Acc:MGI:1917819]	1.358	7.37E-03
ENSMUSG00000029833	Trim24	tripartite motif-containing 24 [Source:MGI Symbol;Acc:MGI:109275]	1.356	6.87E-04
ENSMUSG00000064682	Gm25813	"predicted gene, 25813 [Source:MGI Symbol;Acc:MGI:5455590]"	1.355	2.28E-02
ENSMUSG00000078185	Chml	choroideremia-like [Source:MGI Symbol;Acc:MGI:101913]	1.355	1.08E-02
ENSMUSG00000090674	Gm17082	predicted gene 17082 [Source:MGI Symbol;Acc:MGI:4937909]	1.354	2.40E-02
ENSMUSG00000106822	Gm43533	predicted gene 43533 [Source:MGI Symbol;Acc:MGI:5663670]	1.354	2.52E-02

ENSMUSG00000104114	Gm37297	"predicted gene, 37297 [Source:MGI Symbol;Acc:MGI:5610525]"	1.354	2.83E-02
ENSMUSG00000025134	Alyref	Aly/REF export factor [Source:MGI Symbol;Acc:MGI:1341044]	1.352	1.22E-02
ENSMUSG00000106720	Gm43795	predicted gene 43795 [Source:MGI Symbol;Acc:MGI:5663932]	1.351	2.71E-02
ENSMUSG00000102411	Gm36936	"predicted gene, 36936 [Source:MGI Symbol;Acc:MGI:5610164]"	1.349	6.32E-03
ENSMUSG00000023186	Vwa5a	von Willebrand factor A domain containing 5A [Source:MGI Symbol;Acc:MGI:1915026]	1.349	1.86E-02
ENSMUSG00000104525	Gm37334	"predicted gene, 37334 [Source:MGI Symbol;Acc:MGI:5610562]"	1.348	1.74E-02
ENSMUSG00000047407	Tgif1	TGFB-induced factor homeobox 1 [Source:MGI Symbol;Acc:MGI:1194497]	1.347	1.36E-03
ENSMUSG00000020422	Tns3	tensin 3 [Source:MGI Symbol;Acc:MGI:2443012]	1.346	2.34E-03
ENSMUSG00000035293	G2e3	G2/M-phase specific E3 ubiquitin ligase [Source:MGI Symbol;Acc:MGI:2444298]	1.346	2.18E-02
ENSMUSG00000042476	Abcb4	"ATP-binding cassette, sub-family B (MDR/TAP), member 4 [Source:MGI Symbol;Acc:MGI:97569]"	1.346	7.91E-03
ENSMUSG00000010608	Rbm25	RNA binding motif protein 25 [Source:MGI Symbol;Acc:MGI:1914289]	1.346	3.89E-04
ENSMUSG00000064302	Clasp1	CLIP associating protein 1 [Source:MGI Symbol;Acc:MGI:1923957]	1.345	2.79E-02
ENSMUSG00000104706	Gm43421	predicted gene 43421 [Source:MGI Symbol;Acc:MGI:5663558]	1.345	1.67E-03
ENSMUSG00000074151	Nlrc5	"NLR family, CARD domain containing 5 [Source:MGI Symbol;Acc:MGI:3612191]"	1.344	1.11E-02
ENSMUSG00000030159	Clec1b	"C-type lectin domain family 1, member b [Source:MGI Symbol;Acc:MGI:1913287]"	1.342	1.48E-04
ENSMUSG00000090386	Mir99ahg	Mir99a and Mirlet7c-1 host gene (non-protein coding) [Source:MGI Symbol;Acc:MGI:1919929]	1.341	3.08E-03
ENSMUSG00000021003	Galc	galactosylceramidase [Source:MGI Symbol;Acc:MGI:95636]	1.341	4.71E-03
ENSMUSG00000031400	G6pdx	glucose-6-phosphate dehydrogenase X-linked [Source:MGI Symbol;Acc:MGI:105979]	1.341	1.77E-03
ENSMUSG00000040463	Mybbp1a	MYB binding protein (P160) 1a [Source:MGI Symbol;Acc:MGI:106181]	1.340	7.19E-03
ENSMUSG00000017670	Elmo2	engulfment and cell motility 2 [Source:MGI Symbol;Acc:MGI:2153045]	1.340	2.81E-02
ENSMUSG00000021143	Pacs2	phosphofurin acidic cluster sorting protein 2 [Source:MGI Symbol;Acc:MGI:1924399]	1.337	8.30E-03
ENSMUSG00000037111	Setd7	SET domain containing (lysine methyltransferase) 7 [Source:MGI Symbol;Acc:MGI:1920501]	1.336	1.19E-02
ENSMUSG00000106757	Gm43482	predicted gene 43482 [Source:MGI Symbol;Acc:MGI:5663619]	1.334	6.20E-03
ENSMUSG00000106205	C230096K16Rik	RIKEN cDNA C230096K16 gene [Source:MGI Symbol;Acc:MGI:3041194]	1.333	1.45E-02
ENSMUSG00000026979	Psd4	pleckstrin and Sec7 domain containing 4 [Source:MGI Symbol;Acc:MGI:2674093]	1.333	1.01E-05
ENSMUSG00000037679	Inf2	"inverted formin, FH2 and WH2 domain containing [Source:MGI Symbol;Acc:MGI:1917685]"	1.333	4.74E-02
ENSMUSG00000102157	Gm37470	"predicted gene, 37470 [Source:MGI Symbol;Acc:MGI:5610698]"	1.333	3.12E-02
ENSMUSG00000027951	Adar	"adenosine deaminase, RNA-specific [Source:MGI Symbol;Acc:MGI:1889575]"	1.332	6.83E-03
ENSMUSG00000079507	H2-Q1	"histocompatibility 2, Q region locus 1 [Source:MGI Symbol;Acc:MGI:95928]"	1.332	8.80E-03
ENSMUSG00000034320	Slc26a2	"solute carrier family 26 (sulfate transporter), member 2 [Source:MGI Symbol;Acc:MGI:892977]"	1.331	2.86E-04
ENSMUSG00000022969	Il10rb	"interleukin 10 receptor, beta [Source:MGI Symbol;Acc:MGI:109380]"	1.329	6.68E-05
ENSMUSG00000024048	Myl12a	"myosin, light chain 12A, regulatory, non-sarcomeric [Source:MGI Symbol;Acc:MGI:1914518]"	1.329	2.08E-02
ENSMUSG00000105950	Gm43679	predicted gene 43679 [Source:MGI Symbol;Acc:MGI:5663816]	1.328	6.27E-03
ENSMUSG00000041974	Spidr	scaffolding protein involved in DNA repair [Source:MGI Symbol;Acc:MGI:1924834]	1.328	1.96E-02
ENSMUSG00000002603	Tgfb1	"transforming growth factor, beta 1 [Source:MGI Symbol;Acc:MGI:98725]"	1.327	2.02E-02
ENSMUSG00000021270	Hsp90aa1	"heat shock protein 90, alpha (cytosolic), class A member 1 [Source:MGI Symbol;Acc:MGI:96250]"	1.326	1.44E-02
ENSMUSG00000000782	Tcf7	"transcription factor 7, T cell specific [Source:MGI Symbol;Acc:MGI:98507]"	1.326	3.06E-02
ENSMUSG00000066258	Trim12a	tripartite motif-containing 12A [Source:MGI Symbol;Acc:MGI:1923931]	1.325	9.99E-04
ENSMUSG00000102555	6430511E19Rik	RIKEN cDNA 6430511E19 gene [Source:MGI Symbol;Acc:MGI:2443259]	1.324	6.52E-04
ENSMUSG00000024816	Frmd8	FERM domain containing 8 [Source:MGI Symbol;Acc:MGI:1914707]	1.323	1.06E-02
ENSMUSG00000030309	Caprin2	caprin family member 2 [Source:MGI Symbol;Acc:MGI:2448541]	1.323	1.35E-02
ENSMUSG00000050244	Heatr1	HEAT repeat containing 1 [Source:MGI Symbol;Acc:MGI:2442524]	1.322	1.60E-02
ENSMUSG00000003200	Sh3gl1	SH3-domain GRB2-like 1 [Source:MGI Symbol;Acc:MGI:700010]	1.322	4.65E-03
ENSMUSG00000070544	Top1	topoisomerase (DNA) I [Source:MGI Symbol;Acc:MGI:98788]	1.321	2.18E-04
ENSMUSG00000030272	Camk1	calcium/calmodulin-dependent protein kinase I [Source:MGI Symbol;Acc:MGI:1098535]	1.320	2.91E-03
ENSMUSG00000102302	Gm38190	"predicted gene, 38190 [Source:MGI Symbol;Acc:MGI:5611418]"	1.320	8.89E-03
ENSMUSG00000057894	Zfp329	zinc finger protein 329 [Source:MGI Symbol;Acc:MGI:1921283]	1.319	4.01E-03
ENSMUSG00000032178	Ilf3	interleukin enhancer binding factor 3 [Source:MGI Symbol;Acc:MGI:1339973]	1.319	2.37E-03
ENSMUSG00000112822	A130012E19Rik	RIKEN cDNA A130012E19 gene [Source:MGI Symbol;Acc:MGI:2443640]	1.316	8.04E-03
ENSMUSG00000024163	Mapk8ip3	mitogen-activated protein kinase 8 interacting protein 3 [Source:MGI Symbol;Acc:MGI:1353598]	1.315	3.04E-02

ENSMUSG00000044147	Arf6	ADP-ribosylation factor 6 [Source:MGI Symbol;Acc:MGI:99435]	1.314	3.80E-03
ENSMUSG00000104383	Gm37553	"predicted gene, 37553 [Source:MGI Symbol;Acc:MGI:5610781]"	1.314	3.12E-02
ENSMUSG00000028999	Rint1	RAD50 interactor 1 [Source:MGI Symbol;Acc:MGI:1916233]	1.313	1.09E-03
ENSMUSG00000020009	Ifngr1	interferon gamma receptor 1 [Source:MGI Symbol;Acc:MGI:107655]	1.313	1.80E-03
ENSMUSG00000104436	Gm37423	"predicted gene, 37423 [Source:MGI Symbol;Acc:MGI:5610651]"	1.312	4.70E-03
ENSMUSG00000069094	Pde7a	phosphodiesterase 7A [Source:MGI Symbol;Acc:MGI:1202402]	1.312	2.93E-02
ENSMUSG00000021699	Pde4d	"phosphodiesterase 4D, cAMP specific [Source:MGI Symbol;Acc:MGI:99555]"	1.311	1.14E-02
ENSMUSG00000107586	Gm44283	"predicted gene, 44283 [Source:MGI Symbol;Acc:MGI:5690675]"	1.311	4.42E-02
ENSMUSG00000041238	Rbbp8	"retinoblastoma binding protein 8, endonuclease [Source:MGI Symbol;Acc:MGI:2442995]"	1.310	4.87E-02
ENSMUSG00000003134	Tbc1d8	"TBC1 domain family, member 8 [Source:MGI Symbol;Acc:MGI:1927225]"	1.308	3.49E-02
ENSMUSG00000041797	Abca9	"ATP-binding cassette, sub-family A (ABC1), member 9 [Source:MGI Symbol;Acc:MGI:2386796]"	1.307	1.18E-02
ENSMUSG00000022769	Sdf2l1	stromal cell-derived factor 2-like 1 [Source:MGI Symbol;Acc:MGI:2149842]	1.307	3.35E-03
ENSMUSG00000009378	Slc16a12	"solute carrier family 16 (monocarboxylic acid transporters), member 12 [Source:MGI Symbol;Acc:MGI:2147716]"	1.307	7.02E-05
ENSMUSG00000029163	Emilin1	elastin microfibril interfacer 1 [Source:MGI Symbol;Acc:MGI:1926189]	1.307	2.31E-02
ENSMUSG00000002748	Baz1b	"bromodomain adjacent to zinc finger domain, 1B [Source:MGI Symbol;Acc:MGI:1353499]"	1.306	8.19E-04
ENSMUSG00000085334	Gm12940	predicted gene 12940 [Source:MGI Symbol;Acc:MGI:3702626]	1.306	8.91E-04
ENSMUSG00000028842	Ago3	argonaute RISC catalytic subunit 3 [Source:MGI Symbol;Acc:MGI:2446634]	1.306	4.37E-02
ENSMUSG00000062198	2700097O09Rik	RIKEN cDNA 2700097O09 gene [Source:MGI Symbol;Acc:MGI:1919908]	1.306	6.94E-03
ENSMUSG00000027540	Ptpn1	"protein tyrosine phosphatase, non-receptor type 1 [Source:MGI Symbol;Acc:MGI:97805]"	1.304	4.09E-03
ENSMUSG00000105304	Gm43696	predicted gene 43696 [Source:MGI Symbol;Acc:MGI:5663833]	1.304	1.21E-02
ENSMUSG00000022272	Myo10	myosin X [Source:MGI Symbol;Acc:MGI:107716]	1.304	3.04E-02
ENSMUSG00000035392	Dennd1a	DENN/MADD domain containing 1A [Source:MGI Symbol;Acc:MGI:2442794]	1.303	2.44E-02
ENSMUSG00000028088	Fmo5	flavin containing monooxygenase 5 [Source:MGI Symbol;Acc:MGI:1310004]	1.302	8.17E-07
ENSMUSG00000039474	Wfs1	wolframin ER transmembrane glycoprotein [Source:MGI Symbol;Acc:MGI:1328355]	1.301	1.41E-02
ENSMUSG00000035673	Sbno2	strawberry notch 2 [Source:MGI Symbol;Acc:MGI:2448490]	1.301	1.52E-02
ENSMUSG00000033377	Palmd	palmdelphin [Source:MGI Symbol;Acc:MGI:2148896]	1.299	5.51E-04
ENSMUSG00000038116	Phf20	PHD finger protein 20 [Source:MGI Symbol;Acc:MGI:2444148]	1.298	4.81E-02
ENSMUSG00000040209	Zfp704	zinc finger protein 704 [Source:MGI Symbol;Acc:MGI:2180715]	1.298	2.93E-02
ENSMUSG00000027006	Dnajc10	DnaJ heat shock protein family (Hsp40) member C10 [Source:MGI Symbol;Acc:MGI:1914111]	1.298	1.66E-03
ENSMUSG00000030254	Rad18	RAD18 E3 ubiquitin protein ligase [Source:MGI Symbol;Acc:MGI:1890476]	1.297	4.38E-02
ENSMUSG00000028820	Sfpq	splicing factor proline/glutamine rich (polypyrimidine tract binding protein associated) [Source:MGI Symbol;Acc:MGI:1918764]	1.297	1.25E-04
ENSMUSG00000026608	Kctd3	potassium channel tetramerisation domain containing 3 [Source:MGI Symbol;Acc:MGI:2444629]	1.297	2.90E-02
ENSMUSG00000028693	Nasp	nuclear autoantigenic sperm protein (histone-binding) [Source:MGI Symbol;Acc:MGI:1355328]	1.297	1.36E-02
ENSMUSG00000032577	Mapkapk3	mitogen-activated protein kinase-activated protein kinase 3 [Source:MGI Symbol;Acc:MGI:2143163]	1.296	1.15E-02
ENSMUSG00000038507	Parp12	"poly (ADP-ribose) polymerase family, member 12 [Source:MGI Symbol;Acc:MGI:2143990]"	1.296	1.30E-02
ENSMUSG00000108780	5430434F05Rik	RIKEN cDNA 5430434F05 gene [Source:MGI Symbol;Acc:MGI:1925345]	1.296	3.13E-03
ENSMUSG00000113067	Gm47166	"predicted gene, 47166 [Source:MGI Symbol;Acc:MGI:6095944]"	1.294	2.94E-02
ENSMUSG00000097589	Dleu2	"deleted in lymphocytic leukemia, 2 [Source:MGI Symbol;Acc:MGI:1934030]"	1.294	2.94E-05
ENSMUSG00000033792	Atp7a	"ATPase, Cu++ transporting, alpha polypeptide [Source:MGI Symbol;Acc:MGI:99400]"	1.294	1.22E-02
ENSMUSG00000026542	Apcs	serum amyloid P-component [Source:MGI Symbol;Acc:MGI:98229]	1.294	2.45E-02
ENSMUSG00000051391	Ywhag	"tyrosine 3-monooxygenase/tryptophan 5-monooxygenase activation protein, gamma polypeptide [Source:MGI Symbol;Acc:MGI:108109]"	1.293	1.53E-05
ENSMUSG00000110424	1700012D14Rik	RIKEN cDNA 1700012D14 gene [Source:MGI Symbol;Acc:MGI:1922729]	1.293	3.04E-02
ENSMUSG00000035107	Dcbld2	"discoidin, CUB and LCCL domain containing 2 [Source:MGI Symbol;Acc:MGI:1920629]"	1.293	2.43E-02
ENSMUSG00000106087	Gm43609	predicted gene 43609 [Source:MGI Symbol;Acc:MGI:5663746]	1.293	1.35E-02
ENSMUSG00000115148	Gm49125	"predicted gene, 49125 [Source:MGI Symbol;Acc:MGI:6118530]"	1.292	3.36E-02
ENSMUSG00000060090	Rp2	retinitis pigmentosa 2 homolog [Source:MGI Symbol;Acc:MGI:1277953]	1.292	6.46E-03
ENSMUSG00000003778	Brd8	bromodomain containing 8 [Source:MGI Symbol;Acc:MGI:1925906]	1.290	1.42E-03
ENSMUSG00000028645	Slc2a1	"solute carrier family 2 (facilitated glucose transporter), member 1 [Source:MGI Symbol;Acc:MGI:95755]"	1.290	3.36E-02
ENSMUSG00000056612	Ppp1r14b	"protein phosphatase 1, regulatory inhibitor subunit 14B [Source:MGI Symbol;Acc:MGI:107682]"	1.289	2.03E-02
ENSMUSG00000052298	Cdc42se2	CDC42 small effector 2 [Source:MGI Symbol;Acc:MGI:1919979]	1.288	9.85E-03

ENSMUSG00000030655	Smg1	"SMG1 homolog, phosphatidylinositol 3-kinase-related kinase (C. elegans) [Source:MGI Symbol;Acc:MGI:1919742]"	1.287	1.46E-02
ENSMUSG00000031722	Hp	haptoglobin [Source:MGI Symbol;Acc:MGI:96211]	1.287	4.47E-04
ENSMUSG00000104116	Gm37296	"predicted gene, 37296 [Source:MGI Symbol;Acc:MGI:5610524]"	1.286	2.61E-03
ENSMUSG00000025854	Fam20c	"family with sequence similarity 20, member C [Source:MGI Symbol;Acc:MGI:2136853]"	1.286	5.14E-03
ENSMUSG00000035397	Klf16	Kruppel-like factor 16 [Source:MGI Symbol;Acc:MGI:2153049]	1.285	3.57E-02
ENSMUSG00000027751	Supt20	SPT20 SAGA complex component [Source:MGI Symbol;Acc:MGI:1929651]	1.285	3.35E-05
ENSMUSG00000073147	5031425E22Rik	RIKEN cDNA 5031425E22 gene [Source:MGI Symbol;Acc:MGI:1923227]	1.285	6.18E-04
ENSMUSG00000017715	Pgs1	phosphatidylglycerophosphate synthase 1 [Source:MGI Symbol;Acc:MGI:1921701]	1.285	2.03E-02
ENSMUSG00000020290	Xpo1	exportin 1 [Source:MGI Symbol;Acc:MGI:2144013]	1.284	6.46E-03
ENSMUSG00000025278	Flnb	"filamin, beta [Source:MGI Symbol;Acc:MGI:2446089]"	1.284	1.46E-04
ENSMUSG00000103505	Gm38374	"predicted gene, 38374 [Source:MGI Symbol;Acc:MGI:5611602]"	1.283	4.13E-03
ENSMUSG00000026819	Slc25a25	"solute carrier family 25 (mitochondrial carrier, phosphate carrier), member 25 [Source:MGI Symbol;Acc:MGI:1915913]"	1.282	4.21E-03
ENSMUSG00000034160	Ogt	O-linked N-acetylglucosamine (GlcNAc) transferase (UDP-N-acetylglucosamine:polypeptide-N-acetylglucosaminyl transferase) [Source:MGI Symbol;Acc:MGI:1339639]	1.282	1.91E-03
ENSMUSG00000024621	Csf1r	colony stimulating factor 1 receptor [Source:MGI Symbol;Acc:MGI:1339758]	1.281	8.00E-03
ENSMUSG00000105561	Gm43462	predicted gene 43462 [Source:MGI Symbol;Acc:MGI:5663599]	1.281	2.38E-02
ENSMUSG00000036698	Ago2	argonaute RISC catalytic subunit 2 [Source:MGI Symbol;Acc:MGI:2446632]	1.281	7.31E-06
ENSMUSG00000086040	Wipf3	"WAS/WASL interacting protein family, member 3 [Source:MGI Symbol;Acc:MGI:3044681]"	1.280	2.26E-02
ENSMUSG00000032475	Nck1	non-catalytic region of tyrosine kinase adaptor protein 1 [Source:MGI Symbol;Acc:MGI:109601]	1.280	9.44E-03
ENSMUSG00000002728	Naa20	"N(alpha)-acetyltransferase 20, NatB catalytic subunit [Source:MGI Symbol;Acc:MGI:1915127]"	1.279	4.80E-02
ENSMUSG00000029580	Actb	"actin, beta [Source:MGI Symbol;Acc:MGI:87904]"	1.279	3.06E-02
ENSMUSG00000058503	Fam133b	"family with sequence similarity 133, member B [Source:MGI Symbol;Acc:MGI:1915402]"	1.278	1.19E-02
ENSMUSG00000039740	Alg2	"asparagine-linked glycosylation 2 (alpha-1,3-mannosyltransferase) [Source:MGI Symbol;Acc:MGI:1914731]"	1.277	7.35E-03
ENSMUSG00000073411	H2-D1	"histocompatibility 2, D region locus 1 [Source:MGI Symbol;Acc:MGI:95896]"	1.276	1.81E-03
ENSMUSG00000056144	Trim34a	tripartite motif-containing 34A [Source:MGI Symbol;Acc:MGI:2137359]	1.276	1.33E-02
ENSMUSG00000110635	Gm45853	predicted gene 45853 [Source:MGI Symbol;Acc:MGI:5804968]	1.276	2.19E-02
ENSMUSG00000026341	Actr3	ARP3 actin-related protein 3 [Source:MGI Symbol;Acc:MGI:1921367]	1.275	6.13E-04
ENSMUSG00000040459	Arglu1	arginine and glutamate rich 1 [Source:MGI Symbol;Acc:MGI:2442985]	1.275	7.88E-05
ENSMUSG00000034998	Foxn2	forkhead box N2 [Source:MGI Symbol;Acc:MGI:1347478]	1.275	3.19E-03
ENSMUSG00000005103	Wdr1	WD repeat domain 1 [Source:MGI Symbol;Acc:MGI:1337100]	1.274	1.36E-04
ENSMUSG00000057406	Nsd2	nuclear receptor binding SET domain protein 2 [Source:MGI Symbol;Acc:MGI:1276574]	1.274	1.73E-03
ENSMUSG00000030555	Ttc23	tetratricopeptide repeat domain 23 [Source:MGI Symbol;Acc:MGI:1914259]	1.273	1.05E-02
ENSMUSG00000004266	Ptpn6	"protein tyrosine phosphatase, non-receptor type 6 [Source:MGI Symbol;Acc:MGI:96055]"	1.273	7.63E-03
ENSMUSG00000043091	Tuba1c	"tubulin, alpha 1C [Source:MGI Symbol;Acc:MGI:1095409]"	1.272	1.05E-02
ENSMUSG00000033904	Ccp110	centriolar coiled coil protein 110 [Source:MGI Symbol;Acc:MGI:2141942]	1.271	2.82E-02
ENSMUSG00000014763	Fam120b	"family with sequence similarity 120, member B [Source:MGI Symbol;Acc:MGI:1914794]"	1.270	9.16E-04
ENSMUSG00000000838	Fmr1	fragile X mental retardation 1 [Source:MGI Symbol;Acc:MGI:95564]	1.268	7.59E-03
ENSMUSG00000031983	2310022B05Rik	RIKEN cDNA 2310022B05 gene [Source:MGI Symbol;Acc:MGI:1916801]	1.267	4.71E-02
ENSMUSG00000103720	Gm37094	"predicted gene, 37094 [Source:MGI Symbol;Acc:MGI:5610322]"	1.265	1.93E-02
ENSMUSG00000112391	Gm48230	"predicted gene, 48230 [Source:MGI Symbol;Acc:MGI:6097634]"	1.263	2.32E-02
ENSMUSG00000022389	Tef	thyrotroph embryonic factor [Source:MGI Symbol;Acc:MGI:98663]	1.263	1.74E-02
ENSMUSG00000020661	Dnmt3a	DNA methyltransferase 3A [Source:MGI Symbol;Acc:MGI:1261827]	1.262	1.93E-02
ENSMUSG00000113752	Gm47817	"predicted gene, 47817 [Source:MGI Symbol;Acc:MGI:6097004]"	1.261	2.59E-02
ENSMUSG00000025915	Sgk3	serum/glucocorticoid regulated kinase 3 [Source:MGI Symbol;Acc:MGI:2182368]	1.261	3.17E-03
ENSMUSG00000054580	Pla2r1	phospholipase A2 receptor 1 [Source:MGI Symbol;Acc:MGI:102468]	1.261	1.66E-02
ENSMUSG00000042520	Ubap2l	ubiquitin-associated protein 2-like [Source:MGI Symbol;Acc:MGI:1921633]	1.261	4.05E-03
ENSMUSG00000036769	Wdr44	WD repeat domain 44 [Source:MGI Symbol;Acc:MGI:1919654]	1.261	1.89E-02
ENSMUSG00000038855	Itpkb	"inositol 1,4,5-trisphosphate 3-kinase B [Source:MGI Symbol;Acc:MGI:109235]"	1.260	4.80E-02
ENSMUSG00000089281	Scarna6	small Cajal body-specific RNA 6 [Source:MGI Symbol;Acc:MGI:3819487]	1.259	1.67E-02
ENSMUSG00000027778	Ift80	intraflagellar transport 80 [Source:MGI Symbol;Acc:MGI:1915509]	1.259	2.22E-02
ENSMUSG00000025534	Gusb	"glucuronidase, beta [Source:MGI Symbol;Acc:MGI:95872]"	1.259	9.42E-03

ENSMUSG00000029863	Casp2	caspase 2 [Source:MGI Symbol;Acc:MGI:97295]	1.259	1.86E-02
ENSMUSG00000023988	Bysl	bystin-like [Source:MGI Symbol;Acc:MGI:1858419]	1.259	1.56E-02
ENSMUSG00000025232	Hexa	hexosaminidase A [Source:MGI Symbol;Acc:MGI:96073]	1.257	5.47E-04
ENSMUSG00000020790	Ankfy1	ankyrin repeat and FYVE domain containing 1 [Source:MGI Symbol;Acc:MGI:1337008]	1.257	2.02E-02
ENSMUSG00000104293	Gm38043	"predicted gene, 38043 [Source:MGI Symbol;Acc:MGI:5611271]"	1.257	3.18E-02
ENSMUSG00000038072	Galnt11	polypeptide N-acetylgalactosaminyltransferase 11 [Source:MGI Symbol;Acc:MGI:2444392]	1.256	5.76E-04
ENSMUSG00000015143	Actn1	"actinin, alpha 1 [Source:MGI Symbol;Acc:MGI:2137706]"	1.256	8.33E-03
ENSMUSG00000024095	Hnrnpll	heterogeneous nuclear ribonucleoprotein L-like [Source:MGI Symbol;Acc:MGI:1919942]	1.255	1.40E-03
ENSMUSG00000031930	Wwp2	WW domain containing E3 ubiquitin protein ligase 2 [Source:MGI Symbol;Acc:MGI:1914144]	1.255	3.92E-02
ENSMUSG00000035181	Heatr5a	HEAT repeat containing 5A [Source:MGI Symbol;Acc:MGI:2444133]	1.255	8.12E-04
ENSMUSG00000073409	H2-Q6	"histocompatibility 2, Q region locus 6 [Source:MGI Symbol;Acc:MGI:95935]"	1.254	3.32E-02
ENSMUSG00000029175	Slc35f6	"solute carrier family 35, member F6 [Source:MGI Symbol;Acc:MGI:1922169]"	1.254	1.31E-02
ENSMUSG00000000600	Krit1	"KRIT1, ankyrin repeat containing [Source:MGI Symbol;Acc:MGI:1930618]"	1.254	1.20E-02
ENSMUSG00000017774	Myo1c	myosin IC [Source:MGI Symbol;Acc:MGI:106612]	1.254	1.08E-03
ENSMUSG00000110556	Gm45762	predicted gene 45762 [Source:MGI Symbol;Acc:MGI:5804877]	1.253	4.65E-02
ENSMUSG00000034640	Tiparp	TCDD-inducible poly(ADP-ribose) polymerase [Source:MGI Symbol;Acc:MGI:2159210]	1.253	3.62E-02
ENSMUSG00000021027	Ralgapa1	"Ral GTPase activating protein, alpha subunit 1 [Source:MGI Symbol;Acc:MGI:1931050]"	1.253	1.56E-02
ENSMUSG00000027429	Sec23b	"SEC23 homolog B, COPII coat complex component [Source:MGI Symbol;Acc:MGI:1350925]"	1.251	2.79E-03
ENSMUSG00000032557	Uba5	ubiquitin-like modifier activating enzyme 5 [Source:MGI Symbol;Acc:MGI:1913913]	1.251	2.57E-05
ENSMUSG00000041959	S100a10	S100 calcium binding protein A10 (calpactin) [Source:MGI Symbol;Acc:MGI:1339468]	1.250	4.54E-02
ENSMUSG00000107529	Gm44291	"predicted gene, 44291 [Source:MGI Symbol;Acc:MGI:5690683]"	1.250	8.45E-03
ENSMUSG00000028708	Mknk1	MAP kinase-interacting serine/threonine kinase 1 [Source:MGI Symbol;Acc:MGI:894316]	1.249	3.01E-02
ENSMUSG00000103285	Gm37274	"predicted gene, 37274 [Source:MGI Symbol;Acc:MGI:5610502]"	1.249	4.09E-02
ENSMUSG00000004980	Hnrnpa2b1	heterogeneous nuclear ribonucleoprotein A2/B1 [Source:MGI Symbol;Acc:MGI:104819]	1.249	9.16E-04
ENSMUSG00000026893	Gca	grancalcin [Source:MGI Symbol;Acc:MGI:1918521]	1.246	1.68E-02
ENSMUSG00000025034	Trim8	tripartite motif-containing 8 [Source:MGI Symbol;Acc:MGI:1933302]	1.246	2.03E-03
ENSMUSG00000086968	4933431E20Rik	RIKEN cDNA 4933431E20 gene [Source:MGI Symbol;Acc:MGI:3584041]	1.245	3.13E-02
ENSMUSG00000032497	Lrrfip2	leucine rich repeat (in FLII) interacting protein 2 [Source:MGI Symbol;Acc:MGI:1918518]	1.243	5.44E-03
ENSMUSG00000103869	Gm37420	"predicted gene, 37420 [Source:MGI Symbol;Acc:MGI:5610648]"	1.243	2.43E-02
ENSMUSG00000047123	Ticam1	toll-like receptor adaptor molecule 1 [Source:MGI Symbol;Acc:MGI:2147032]	1.243	3.57E-02
ENSMUSG00000055013	Agap1	"ArfGAP with GTPase domain, ankyrin repeat and PH domain 1 [Source:MGI Symbol;Acc:MGI:2653690]"	1.243	3.06E-03
ENSMUSG00000030091	Nup210	nucleoporin 210 [Source:MGI Symbol;Acc:MGI:1859555]	1.243	5.50E-03
ENSMUSG00000033769	Exoc6b	exocyst complex component 6B [Source:MGI Symbol;Acc:MGI:1923164]	1.242	1.98E-02
ENSMUSG00000036890	Gtdc1	glycosyltransferase-like domain containing 1 [Source:MGI Symbol;Acc:MGI:2444269]	1.242	3.68E-02
ENSMUSG00000057691	Zfp746	zinc finger protein 746 [Source:MGI Symbol;Acc:MGI:1916478]	1.240	1.81E-02
ENSMUSG00000104394	Gm37254	"predicted gene, 37254 [Source:MGI Symbol;Acc:MGI:5610482]"	1.239	6.94E-03
ENSMUSG00000105728	Gm42819	predicted gene 42819 [Source:MGI Symbol;Acc:MGI:5662956]	1.239	1.39E-02
ENSMUSG00000022498	Txndc11	thioredoxin domain containing 11 [Source:MGI Symbol;Acc:MGI:1923620]	1.238	2.79E-03
ENSMUSG00000032621	Srek1	splicing regulatory glutamine/lysine-rich protein 1 [Source:MGI Symbol;Acc:MGI:2145245]	1.237	3.87E-03
ENSMUSG00000093843	Gm25939	"predicted gene, 25939 [Source:MGI Symbol;Acc:MGI:5455716]"	1.236	4.18E-03
ENSMUSG00000026317	Cln8	CLN8 transmembrane ER and ERGIC protein [Source:MGI Symbol;Acc:MGI:1349447]	1.236	1.10E-04
ENSMUSG00000113766	Gm47374	"predicted gene, 47374 [Source:MGI Symbol;Acc:MGI:6096288]"	1.234	4.64E-02
ENSMUSG00000053646	Plxnb1	plexin B1 [Source:MGI Symbol;Acc:MGI:2154238]	1.234	1.36E-02
ENSMUSG00000066037	Hnrnpr	heterogeneous nuclear ribonucleoprotein R [Source:MGI Symbol;Acc:MGI:1891692]	1.234	2.21E-03
ENSMUSG00000070738	Dgkd	"diacylglycerol kinase, delta [Source:MGI Symbol;Acc:MGI:2138334]"	1.233	2.77E-02
ENSMUSG00000039191	Rbpj	recombination signal binding protein for immunoglobulin kappa J region [Source:MGI Symbol;Acc:MGI:96522]	1.233	2.78E-02
ENSMUSG00000103065	Gm20236	"predicted gene, 20236 [Source:MGI Symbol;Acc:MGI:5012421]"	1.231	2.88E-02
ENSMUSG00000001785	Pwp1	"PWP1 homolog, endonuclein [Source:MGI Symbol;Acc:MGI:1914735]"	1.230	4.89E-02
ENSMUSG00000027763	Mbnl1	muscleblind like splicing factor 1 [Source:MGI Symbol;Acc:MGI:1928482]	1.230	1.13E-02
ENSMUSG00000036435	Exoc1	exocyst complex component 1 [Source:MGI Symbol;Acc:MGI:2445020]	1.230	1.38E-02

ENSMUSG00000039994	Timeless	timeless circadian clock 1 [Source:MGI Symbol;Acc:MGI:1321393]	1.230	2.85E-02
ENSMUSG00000038473	Nos1ap	nitric oxide synthase 1 (neuronal) adaptor protein [Source:MGI Symbol;Acc:MGI:1917979]	1.229	4.22E-02
ENSMUSG00000015488	Cacfd1	calcium channel flower domain containing 1 [Source:MGI Symbol;Acc:MGI:1924317]	1.229	3.88E-03
ENSMUSG00000107286	Gm43788	predicted gene 43788 [Source:MGI Symbol;Acc:MGI:5663925]	1.227	1.92E-02
ENSMUSG00000021939	Ctsb	cathepsin B [Source:MGI Symbol;Acc:MGI:88561]	1.227	1.41E-04
ENSMUSG00000020532	Acaca	acetyl-Coenzyme A carboxylase alpha [Source:MGI Symbol;Acc:MGI:108451]	1.227	1.56E-02
ENSMUSG00000049521	Cdc42ep1	CDC42 effector protein (Rho GTPase binding) 1 [Source:MGI Symbol;Acc:MGI:1929763]	1.225	3.27E-02
ENSMUSG00000021877	Arf4	ADP-ribosylation factor 4 [Source:MGI Symbol;Acc:MGI:99433]	1.222	2.93E-02
ENSMUSG00000018001	Cyth3	cytohesin 3 [Source:MGI Symbol;Acc:MGI:1335107]	1.221	3.32E-02
ENSMUSG00000023004	Tuba1b	"tubulin, alpha 1B [Source:MGI Symbol;Acc:MGI:107804]"	1.220	1.55E-02
ENSMUSG00000028790	Khdrbs1	"KH domain containing, RNA binding, signal transduction associated 1 [Source:MGI Symbol;Acc:MGI:893579]"	1.220	1.89E-02
ENSMUSG00000034663	Bmp2k	BMP2 inducible kinase [Source:MGI Symbol;Acc:MGI:2155456]	1.218	1.32E-03
ENSMUSG00000109045	Gm45084	predicted gene 45084 [Source:MGI Symbol;Acc:MGI:5753660]	1.216	1.83E-02
ENSMUSG00000029221	Slc30a9	"solute carrier family 30 (zinc transporter), member 9 [Source:MGI Symbol;Acc:MGI:1923690]"	1.215	2.12E-02
ENSMUSG00000117448	Gm50055	"predicted gene, 50055 [Source:MGI Symbol;Acc:MGI:6275370]"	1.215	3.92E-02
ENSMUSG00000085385	Snhg17	small nucleolar RNA host gene 17 [Source:MGI Symbol;Acc:MGI:1915358]	1.215	1.37E-02
ENSMUSG00000039463	Slc9a8	"solute carrier family 9 (sodium/hydrogen exchanger), member 8 [Source:MGI Symbol;Acc:MGI:1924281]"	1.215	2.22E-02
ENSMUSG00000057672	Pkn1	protein kinase N1 [Source:MGI Symbol;Acc:MGI:108022]	1.215	3.02E-02
ENSMUSG00000030264	Thumpd3	THUMP domain containing 3 [Source:MGI Symbol;Acc:MGI:1277973]	1.213	3.75E-03
ENSMUSG00000059552	Trp53	transformation related protein 53 [Source:MGI Symbol;Acc:MGI:98834]	1.213	4.06E-02
ENSMUSG00000018583	G3bp1	GTPase activating protein (SH3 domain) binding protein 1 [Source:MGI Symbol;Acc:MGI:1351465]	1.213	1.81E-04
ENSMUSG00000023923	Tbc1d5	"TBC1 domain family, member 5 [Source:MGI Symbol;Acc:MGI:1919488]"	1.212	3.14E-02
ENSMUSG00000030435	U2af2	U2 small nuclear ribonucleoprotein auxiliary factor (U2AF) 2 [Source:MGI Symbol;Acc:MGI:98886]	1.212	6.88E-03
ENSMUSG00000024603	Dctn4	dynactin 4 [Source:MGI Symbol;Acc:MGI:1914915]	1.212	1.80E-02
ENSMUSG00000114104	Gm47585	"predicted gene, 47585 [Source:MGI Symbol;Acc:MGI:6096625]"	1.212	2.50E-02
ENSMUSG00000109324	Prmt1	protein arginine N-methyltransferase 1 [Source:MGI Symbol;Acc:MGI:107846]	1.211	3.17E-02
ENSMUSG00000027582	Zgpat	"zinc finger, CCCH-type with G patch domain [Source:MGI Symbol;Acc:MGI:2449939]"	1.211	2.90E-04
ENSMUSG00000021392	Nol8	nucleolar protein 8 [Source:MGI Symbol;Acc:MGI:1918180]	1.209	2.02E-02
ENSMUSG00000021713	Ppwd1	peptidylprolyl isomerase domain and WD repeat containing 1 [Source:MGI Symbol;Acc:MGI:2443069]	1.209	9.22E-03
ENSMUSG00000038005	Hpf1	histone PARylation factor 1 [Source:MGI Symbol;Acc:MGI:1919862]	1.208	2.25E-02
ENSMUSG00000021639	Gtf2h2	"general transcription factor II H, polypeptide 2 [Source:MGI Symbol;Acc:MGI:1345669]"	1.206	2.03E-02
ENSMUSG00000035125	Gcfc2	GC-rich sequence DNA binding factor 2 [Source:MGI Symbol;Acc:MGI:2141656]	1.205	1.22E-02
ENSMUSG00000056260	Lrif1	ligand dependent nuclear receptor interacting factor 1 [Source:MGI Symbol;Acc:MGI:2445214]	1.204	6.48E-03
ENSMUSG00000046794	Ppp1r3b	"protein phosphatase 1, regulatory subunit 3B [Source:MGI Symbol;Acc:MGI:2177268]"	1.204	2.63E-02
ENSMUSG00000026942	Traf2	TNF receptor-associated factor 2 [Source:MGI Symbol;Acc:MGI:101835]	1.204	1.95E-02
ENSMUSG00000024644	Cndp2	CNDP dipeptidase 2 (metallopeptidase M20 family) [Source:MGI Symbol;Acc:MGI:1913304]	1.204	2.91E-03
ENSMUSG00000027175	Tcp11l1	t-complex 11 like 1 [Source:MGI Symbol;Acc:MGI:2444263]	1.204	6.53E-04
ENSMUSG00000039501	Znfx1	"zinc finger, NFX1-type containing 1 [Source:MGI Symbol;Acc:MGI:2138982]"	1.202	2.57E-03
ENSMUSG00000022973	Synj1	synaptojanin 1 [Source:MGI Symbol;Acc:MGI:1354961]	1.198	9.80E-04
ENSMUSG00000046879	Irgm1	immunity-related GTPase family M member 1 [Source:MGI Symbol;Acc:MGI:107567]	1.197	3.95E-02
ENSMUSG00000028256	Odf2l	outer dense fiber of sperm tails 2-like [Source:MGI Symbol;Acc:MGI:1098600]	1.193	7.85E-03
ENSMUSG00000032479	Map4	microtubule-associated protein 4 [Source:MGI Symbol;Acc:MGI:97178]	1.192	4.29E-02
ENSMUSG00000102151	Gm37472	"predicted gene, 37472 [Source:MGI Symbol;Acc:MGI:5610700]"	1.192	2.72E-02
ENSMUSG00000090946	Ccdc71l	coiled-coil domain containing 71 like [Source:MGI Symbol;Acc:MGI:1919373]	1.192	4.60E-02
ENSMUSG00000049516	Spty2d1	SPT2 chromatin protein domain containing 1 [Source:MGI Symbol;Acc:MGI:2142062]	1.191	6.94E-03
ENSMUSG00000020638	Cmpk2	"cytidine monophosphate (UMP-CMP) kinase 2, mitochondrial [Source:MGI Symbol;Acc:MGI:99830]"	1.190	4.92E-02
ENSMUSG00000052539	Magi3	"membrane associated guanylate kinase, WW and PDZ domain containing 3 [Source:MGI Symbol;Acc:MGI:1923484]"	1.187	2.53E-02
ENSMUSG00000034120	Srsf2	serine and arginine-rich splicing factor 2 [Source:MGI Symbol;Acc:MGI:98284]	1.187	9.10E-04
ENSMUSG00000102719	Gm37760	"predicted gene, 37760 [Source:MGI Symbol;Acc:MGI:5610988]"	1.187	1.40E-02
ENSMUSG00000102964	9430034N14Rik	RIKEN cDNA 9430034N14 gene [Source:MGI Symbol;Acc:MGI:2444791]	1.185	1.99E-02

ENSMUSG00000065087	Snord22	"small nucleolar RNA, C/D box 22 [Source:MGI Symbol;Acc:MGI:1933385]"	1.185	3.32E-02
ENSMUSG00000033955	Tnks1bp1	tankyrase 1 binding protein 1 [Source:MGI Symbol;Acc:MGI:2446193]	1.184	2.18E-02
ENSMUSG00000028676	Srsf10	serine and arginine-rich splicing factor 10 [Source:MGI Symbol;Acc:MGI:1333805]	1.184	2.91E-02
ENSMUSG00000031700	Gpt2	glutamic pyruvate transaminase (alanine aminotransferase) 2 [Source:MGI Symbol;Acc:MGI:1915391]	1.184	3.77E-04
ENSMUSG00000055436	Srsf11	serine and arginine-rich splicing factor 11 [Source:MGI Symbol;Acc:MGI:1916457]	1.183	1.84E-03
ENSMUSG00000026754	Golga1	"golgi autoantigen, golgin subfamily a, 1 [Source:MGI Symbol;Acc:MGI:1924149]"	1.183	8.13E-03
ENSMUSG00000044757	Gm6430	predicted gene 6430 [Source:MGI Symbol;Acc:MGI:3648857]	1.182	3.04E-02
ENSMUSG00000058317	Ube2e2	ubiquitin-conjugating enzyme E2E 2 [Source:MGI Symbol;Acc:MGI:2384997]	1.182	1.99E-02
ENSMUSG00000022139	Mbnl2	muscleblind like splicing factor 2 [Source:MGI Symbol;Acc:MGI:2145597]	1.182	9.17E-03
ENSMUSG00000028134	Ptbp2	polypyrimidine tract binding protein 2 [Source:MGI Symbol;Acc:MGI:1860489]	1.181	2.66E-02
ENSMUSG00000083396	Gm15542	predicted gene 15542 [Source:MGI Symbol;Acc:MGI:3782990]	1.181	4.00E-02
ENSMUSG00000055044	Pdlim1	PDZ and LIM domain 1 (elfin) [Source:MGI Symbol;Acc:MGI:1860611]	1.181	1.21E-02
ENSMUSG00000052593	Adam17	a disintegrin and metallopeptidase domain 17 [Source:MGI Symbol;Acc:MGI:1096335]	1.181	1.90E-03
ENSMUSG00000044345	Marveld1	MARVEL (membrane-associating) domain containing 1 [Source:MGI Symbol;Acc:MGI:2147570]	1.180	1.81E-03
ENSMUSG00000104503	Gm37738	"predicted gene, 37738 [Source:MGI Symbol;Acc:MGI:5610966]"	1.180	1.45E-02
ENSMUSG00000041417	Pik3r1	phosphoinositide-3-kinase regulatory subunit 1 [Source:MGI Symbol;Acc:MGI:97583]	1.179	1.38E-03
ENSMUSG00000058126	Tpm3-rs7	"tropomyosin 3, related sequence 7 [Source:MGI Symbol;Acc:MGI:99705]"	1.179	3.20E-02
ENSMUSG00000100801	Gm15459	predicted gene 15459 [Source:MGI Symbol;Acc:MGI:3705702]	1.179	3.37E-03
ENSMUSG00000053604	Rpia	ribose 5-phosphate isomerase A [Source:MGI Symbol;Acc:MGI:103254]	1.178	4.30E-02
ENSMUSG00000031668	Eif2ak3	eukaryotic translation initiation factor 2 alpha kinase 3 [Source:MGI Symbol;Acc:MGI:1341830]	1.178	1.20E-02
ENSMUSG00000029178	Klf3	Kruppel-like factor 3 (basic) [Source:MGI Symbol;Acc:MGI:1342773]	1.177	1.69E-02
ENSMUSG00000027200	Sema6d	"sema domain, transmembrane domain (TM), and cytoplasmic domain, (semaphorin) 6D [Source:MGI Symbol;Acc:MGI:2387661]"	1.176	2.20E-02
ENSMUSG00000107951	Gm6210	predicted gene 6210 [Source:MGI Symbol;Acc:MGI:3643375]	1.174	2.82E-02
ENSMUSG00000040033	Stat2	signal transducer and activator of transcription 2 [Source:MGI Symbol;Acc:MGI:103039]	1.174	3.18E-02
ENSMUSG00000089774	Slc5a3	"solute carrier family 5 (inositol transporters), member 3 [Source:MGI Symbol;Acc:MGI:1858226]"	1.173	1.88E-03
ENSMUSG00000061665	Cd2ap	CD2-associated protein [Source:MGI Symbol;Acc:MGI:1330281]	1.173	8.78E-03
ENSMUSG00000085396	Firre	functional intergenic repeating RNA element [Source:MGI Symbol;Acc:MGI:2147989]	1.172	4.87E-02
ENSMUSG00000062232	Rapgef2	Rap guanine nucleotide exchange factor (GEF) 2 [Source:MGI Symbol;Acc:MGI:2659071]	1.170	5.46E-03
ENSMUSG00000047719	Ubiad1	UbiA prenyltransferase domain containing 1 [Source:MGI Symbol;Acc:MGI:1918957]	1.169	4.27E-02
ENSMUSG00000029916	Agk	acylglycerol kinase [Source:MGI Symbol;Acc:MGI:1917173]	1.168	3.11E-02
ENSMUSG00000026727	Rsu1	Ras suppressor protein 1 [Source:MGI Symbol;Acc:MGI:103040]	1.168	1.33E-03
ENSMUSG00000022865	Cxadr	coxsackie virus and adenovirus receptor [Source:MGI Symbol;Acc:MGI:1201679]	1.168	1.87E-02
ENSMUSG00000018736	Ndel1	nudE neurodevelopment protein 1 like 1 [Source:MGI Symbol;Acc:MGI:1932915]	1.167	4.44E-02
ENSMUSG00000032580	Rbm5	RNA binding motif protein 5 [Source:MGI Symbol;Acc:MGI:1933204]	1.166	1.55E-02
ENSMUSG00000041360	Pum3	pumilio RNA-binding family member 3 [Source:MGI Symbol;Acc:MGI:106253]	1.163	5.77E-03
ENSMUSG00000015290	Ubl4a	ubiquitin-like 4A [Source:MGI Symbol;Acc:MGI:95049]	1.162	2.12E-02
ENSMUSG00000106959	Gm42548	predicted gene 42548 [Source:MGI Symbol;Acc:MGI:5662685]	1.160	4.74E-02
ENSMUSG00000020102	Slc16a7	"solute carrier family 16 (monocarboxylic acid transporters), member 7 [Source:MGI Symbol;Acc:MGI:1330284]"	1.160	2.42E-02
ENSMUSG00000059436	Max	Max protein [Source:MGI Symbol;Acc:MGI:96921]	1.160	1.25E-02
ENSMUSG00000061759	Armt1	acidic residue methyltransferase 1 [Source:MGI Symbol;Acc:MGI:1920669]	1.160	4.96E-02
ENSMUSG00000034807	Colgalt1	collagen beta(1-O)galactosyltransferase 1 [Source:MGI Symbol;Acc:MGI:1924348]	1.160	5.27E-03
ENSMUSG00000116564	Riok2	RIO kinase 2 [Source:MGI Symbol;Acc:MGI:1914295]	1.159	3.64E-02
ENSMUSG00000006273	Atp6v1b2	"ATPase, H+ transporting, lysosomal V1 subunit B2 [Source:MGI Symbol;Acc:MGI:109618]"	1.156	2.39E-02
ENSMUSG00000028656	Cap1	"CAP, adenylate cyclase-associated protein 1 (yeast) [Source:MGI Symbol;Acc:MGI:88262]"	1.155	1.44E-02
ENSMUSG00000022159	Rab2b	"RAB2B, member RAS oncogene family [Source:MGI Symbol;Acc:MGI:1923588]"	1.154	4.98E-02
ENSMUSG00000107116	Gm43274	predicted gene 43274 [Source:MGI Symbol;Acc:MGI:5663411]	1.152	1.99E-02
ENSMUSG00000114797	Gm49336	"predicted gene, 49336 [Source:MGI Symbol;Acc:MGI:6121524]"	1.152	8.55E-03
ENSMUSG00000031781	Ciapin1	cytokine induced apoptosis inhibitor 1 [Source:MGI Symbol;Acc:MGI:1922083]	1.151	1.90E-02
ENSMUSG00000029534	St7	suppression of tumorigenicity 7 [Source:MGI Symbol;Acc:MGI:1927450]	1.151	2.37E-02
ENSMUSG00000074030	Exoc8	exocyst complex component 8 [Source:MGI Symbol;Acc:MGI:2142527]	1.150	3.97E-02

ENSMUSG00000027881	Prpf38b	PRP38 pre-mRNA processing factor 38 (yeast) domain containing B [Source:MGI Symbol;Acc:MGI:1914171]	1.150	2.14E-05
ENSMUSG00000020250	Txnrd1	thioredoxin reductase 1 [Source:MGI Symbol;Acc:MGI:1354175]	1.148	2.46E-03
ENSMUSG00000012114	Med15	mediator complex subunit 15 [Source:MGI Symbol;Acc:MGI:2137379]	1.147	2.87E-02
ENSMUSG00000019189	Rnf145	ring finger protein 145 [Source:MGI Symbol;Acc:MGI:1921565]	1.146	7.79E-03
ENSMUSG00000032228	Tcf12	transcription factor 12 [Source:MGI Symbol;Acc:MGI:101877]	1.145	1.02E-02
ENSMUSG00000004530	Coro1c	"coronin, actin binding protein 1C [Source:MGI Symbol;Acc:MGI:1345964]"	1.144	2.65E-02
ENSMUSG00000028410	Dnaja1	DnaJ heat shock protein family (Hsp40) member A1 [Source:MGI Symbol;Acc:MGI:1270129]	1.144	3.97E-02
ENSMUSG00000024594	Prrc1	proline-rich coiled-coil 1 [Source:MGI Symbol;Acc:MGI:1916106]	1.144	3.64E-04
ENSMUSG00000020576	Nbas	neuroblastoma amplified sequence [Source:MGI Symbol;Acc:MGI:1918419]	1.143	3.00E-02
ENSMUSG00000000561	Wdr77	WD repeat domain 77 [Source:MGI Symbol;Acc:MGI:1917715]	1.143	4.18E-02
ENSMUSG00000025982	Sf3b1	"splicing factor 3b, subunit 1 [Source:MGI Symbol;Acc:MGI:1932339]"	1.140	2.50E-02
ENSMUSG00000045409	Trim39	tripartite motif-containing 39 [Source:MGI Symbol;Acc:MGI:1890659]	1.138	4.02E-02
ENSMUSG00000036155	Mgat5	mannoside acetylglucosaminyltransferase 5 [Source:MGI Symbol;Acc:MGI:894701]	1.138	1.77E-02
ENSMUSG00000028634	Hivep3	human immunodeficiency virus type I enhancer binding protein 3 [Source:MGI Symbol;Acc:MGI:106589]	1.138	2.77E-04
ENSMUSG00000020538	Srebf1	sterol regulatory element binding transcription factor 1 [Source:MGI Symbol;Acc:MGI:107606]	1.137	4.38E-02
ENSMUSG00000067017	Capza1-ps1	"capping protein (actin filament) muscle Z-line, alpha 1, pseudogene 1 [Source:MGI Symbol;Acc:MGI:106236]"	1.137	4.19E-02
ENSMUSG00000117113	Gm49883	"predicted gene, 49883 [Source:MGI Symbol;Acc:MGI:6270565]"	1.137	3.76E-02
ENSMUSG00000048578	Mlec	malectin [Source:MGI Symbol;Acc:MGI:1924015]	1.137	4.67E-03
ENSMUSG00000040723	Rcsd1	RCSD domain containing 1 [Source:MGI Symbol;Acc:MGI:2676394]	1.136	2.75E-02
ENSMUSG00000104339	C130089K02Rik	RIKEN cDNA C130089K02 gene [Source:MGI Symbol;Acc:MGI:2444420]	1.136	4.66E-02
ENSMUSG00000039234	Sec24d	"Sec24 related gene family, member D (S. cerevisiae) [Source:MGI Symbol;Acc:MGI:1916858]"	1.134	5.04E-03
ENSMUSG00000032212	Sltm	"SAFB-like, transcription modulator [Source:MGI Symbol;Acc:MGI:1913910]"	1.132	1.22E-02
ENSMUSG00000028683	Eif2b3	"eukaryotic translation initiation factor 2B, subunit 3 [Source:MGI Symbol;Acc:MGI:1313286]"	1.132	2.64E-02
ENSMUSG00000039735	Fnbp1l	formin binding protein 1-like [Source:MGI Symbol;Acc:MGI:1925642]	1.131	5.39E-03
ENSMUSG00000045962	Wnk1	WNK lysine deficient protein kinase 1 [Source:MGI Symbol;Acc:MGI:2442092]	1.127	1.80E-03
ENSMUSG00000022895	Ets2	"E26 avian leukemia oncogene 2, 3' domain [Source:MGI Symbol;Acc:MGI:95456]"	1.125	3.59E-02
ENSMUSG00000030064	Frmd4b	FERM domain containing 4B [Source:MGI Symbol;Acc:MGI:2141794]	1.124	1.97E-02
ENSMUSG00000025236	Adpgk	ADP-dependent glucokinase [Source:MGI Symbol;Acc:MGI:1919391]	1.124	6.44E-03
ENSMUSG00000027801	Tm4sf4	transmembrane 4 superfamily member 4 [Source:MGI Symbol;Acc:MGI:2385173]	1.122	4.28E-02
ENSMUSG00000032115	Hyou1	hypoxia up-regulated 1 [Source:MGI Symbol;Acc:MGI:108030]	1.121	8.59E-03
ENSMUSG00000030695	Aldoa	"aldolase A, fructose-bisphosphate [Source:MGI Symbol;Acc:MGI:87994]"	1.119	1.19E-02
ENSMUSG00000001833	Septin7	septin 7 [Source:MGI Symbol;Acc:MGI:1335094]	1.119	4.54E-02
ENSMUSG00000025017	Pik3ap1	phosphoinositide-3-kinase adaptor protein 1 [Source:MGI Symbol;Acc:MGI:1933177]	1.118	7.93E-03
ENSMUSG00000043998	Mgat2	mannoside acetylglucosaminyltransferase 2 [Source:MGI Symbol;Acc:MGI:2384966]	1.117	7.63E-03
ENSMUSG00000028483	Snapc3	"small nuclear RNA activating complex, polypeptide 3 [Source:MGI Symbol;Acc:MGI:1916338]"	1.117	3.06E-02
ENSMUSG00000042901	Aida	"axin interactor, dorsalization associated [Source:MGI Symbol;Acc:MGI:1919737]"	1.117	2.92E-02
ENSMUSG00000031711	Zfp330	zinc finger protein 330 [Source:MGI Symbol;Acc:MGI:1353574]	1.116	1.35E-02
ENSMUSG00000034708	Grn	granulin [Source:MGI Symbol;Acc:MGI:95832]	1.116	2.92E-02
ENSMUSG00000018750	Zbtb4	zinc finger and BTB domain containing 4 [Source:MGI Symbol;Acc:MGI:1922830]	1.116	1.87E-02
ENSMUSG00000020719	Ddx5	DEAD (Asp-Glu-Ala-Asp) box polypeptide 5 [Source:MGI Symbol;Acc:MGI:105037]	1.114	2.02E-02
ENSMUSG00000029328	Hnrnpdl	heterogeneous nuclear ribonucleoprotein D-like [Source:MGI Symbol;Acc:MGI:1355299]	1.113	2.82E-02
ENSMUSG00000020088	Sar1a	secretion associated Ras related GTPase 1A [Source:MGI Symbol;Acc:MGI:98230]	1.113	3.47E-03
ENSMUSG00000034893	Cog3	component of oligomeric golgi complex 3 [Source:MGI Symbol;Acc:MGI:2450151]	1.112	1.58E-02
ENSMUSG00000029185	Fam114a1	"family with sequence similarity 114, member A1 [Source:MGI Symbol;Acc:MGI:1915553]"	1.112	4.05E-02
ENSMUSG00000116185	Gm49411	"predicted gene, 49411 [Source:MGI Symbol;Acc:MGI:6155037]"	1.109	4.27E-02
ENSMUSG00000024969	Mark2	MAP/microtubule affinity regulating kinase 2 [Source:MGI Symbol;Acc:MGI:99638]	1.109	1.98E-02
ENSMUSG00000035929	H2-Q4	"histocompatibility 2, Q region locus 4 [Source:MGI Symbol;Acc:MGI:95933]"	1.108	8.37E-03
ENSMUSG00000047789	Slc38a9	"solute carrier family 38, member 9 [Source:MGI Symbol;Acc:MGI:1918839]"	1.106	3.95E-02
ENSMUSG00000020458	Rtn4	reticulon 4 [Source:MGI Symbol;Acc:MGI:1915835]	1.106	4.19E-03
ENSMUSG00000031537	Ikbkb	inhibitor of kappaB kinase beta [Source:MGI Symbol;Acc:MGI:1338071]	1.106	1.04E-02

ENSMUSG00000033732	Sf3b3	"splicing factor 3b, subunit 3 [Source:MGI Symbol;Acc:MGI:1289341]"	1.105	4.23E-02
ENSMUSG00000027829	Ccnl1	cyclin L1 [Source:MGI Symbol;Acc:MGI:1922664]	1.102	1.79E-03
ENSMUSG00000038828	Tmem214	transmembrane protein 214 [Source:MGI Symbol;Acc:MGI:1916046]	1.102	4.73E-02
ENSMUSG00000043760	Pkhd1	polycystic kidney and hepatic disease 1 [Source:MGI Symbol;Acc:MGI:2155808]	1.101	3.12E-02
ENSMUSG00000041890	Git2	GIT ArfGAP 2 [Source:MGI Symbol;Acc:MGI:1347053]	1.097	1.12E-02
ENSMUSG00000031652	N4bp1	NEDD4 binding protein 1 [Source:MGI Symbol;Acc:MGI:2136825]	1.097	4.16E-02
ENSMUSG00000020255	D10Wsu102e	"DNA segment, Chr 10, Wayne State University 102, expressed [Source:MGI Symbol;Acc:MGI:106381]"	1.095	6.87E-03
ENSMUSG00000047963	Stbd1	starch binding domain 1 [Source:MGI Symbol;Acc:MGI:1261768]	1.094	1.10E-02
ENSMUSG00000059208	Hnrnpm	heterogeneous nuclear ribonucleoprotein M [Source:MGI Symbol;Acc:MGI:1926465]	1.094	2.42E-02
ENSMUSG00000075232	Amd1	S-adenosylmethionine decarboxylase 1 [Source:MGI Symbol;Acc:MGI:88004]	1.092	1.85E-02
ENSMUSG00000009772	Nuak2	"NUAK family, SNF1-like kinase, 2 [Source:MGI Symbol;Acc:MGI:1921387]"	1.092	2.20E-02
ENSMUSG00000027522	Stx16	syntaxin 16 [Source:MGI Symbol;Acc:MGI:1923396]	1.091	2.84E-02
ENSMUSG00000029823	Luc7l2	LUC7-like 2 (S. cerevisiae) [Source:MGI Symbol;Acc:MGI:2183260]	1.091	8.98E-03
ENSMUSG00000042506	Usp22	ubiquitin specific peptidase 22 [Source:MGI Symbol;Acc:MGI:2144157]	1.091	1.42E-02
ENSMUSG00000063317	Usp31	ubiquitin specific peptidase 31 [Source:MGI Symbol;Acc:MGI:1923429]	1.091	2.57E-02
ENSMUSG00000029647	Pan3	PAN3 poly(A) specific ribonuclease subunit [Source:MGI Symbol;Acc:MGI:1919837]	1.089	3.25E-02
ENSMUSG00000030895	Hpx	hemopexin [Source:MGI Symbol;Acc:MGI:105112]	1.087	1.11E-02
ENSMUSG00000032194	Kank2	KN motif and ankyrin repeat domains 2 [Source:MGI Symbol;Acc:MGI:2384568]	1.087	3.45E-02
ENSMUSG00000049327	Kmt5a	lysine methyltransferase 5A [Source:MGI Symbol;Acc:MGI:1915206]	1.087	2.76E-03
ENSMUSG00000034926	Dhcr24	24-dehydrocholesterol reductase [Source:MGI Symbol;Acc:MGI:1922004]	1.085	1.83E-02
ENSMUSG00000097536	2610037D02Rik	RIKEN cDNA 2610037D02 gene [Source:MGI Symbol;Acc:MGI:1917290]	1.084	3.24E-02
ENSMUSG00000064289	Tank	TRAF family member-associated Nf-kappa B activator [Source:MGI Symbol;Acc:MGI:107676]	1.084	1.48E-02
ENSMUSG00000009293	Ube2g2	ubiquitin-conjugating enzyme E2G 2 [Source:MGI Symbol;Acc:MGI:1343188]	1.083	1.10E-02
ENSMUSG00000021823	Vcl	vinculin [Source:MGI Symbol;Acc:MGI:98927]	1.074	3.92E-02
ENSMUSG00000063953	Amd2	S-adenosylmethionine decarboxylase 2 [Source:MGI Symbol;Acc:MGI:1333111]	1.072	4.26E-02
ENSMUSG00000053841	Txlna	taxilin alpha [Source:MGI Symbol;Acc:MGI:105968]	1.072	4.13E-02
ENSMUSG00000061751	Kalrn	"kalirin, RhoGEF kinase [Source:MGI Symbol;Acc:MGI:2685385]"	1.069	2.53E-04
ENSMUSG00000026305	Lrrfip1	leucine rich repeat (in FLII) interacting protein 1 [Source:MGI Symbol;Acc:MGI:1342770]	1.069	2.64E-02
ENSMUSG00000046434	Hnrnpa1	heterogeneous nuclear ribonucleoprotein A1 [Source:MGI Symbol;Acc:MGI:104820]	1.068	2.05E-03
ENSMUSG00000038280	Ostm1	osteopetrosis associated transmembrane protein 1 [Source:MGI Symbol;Acc:MGI:2655574]	1.067	4.92E-02
ENSMUSG00000004642	Slbp	stem-loop binding protein [Source:MGI Symbol;Acc:MGI:108402]	1.066	2.63E-02
ENSMUSG00000022814	Umps	uridine monophosphate synthetase [Source:MGI Symbol;Acc:MGI:1298388]	1.064	4.54E-02
ENSMUSG00000032244	Fem1b	fem 1 homolog b [Source:MGI Symbol;Acc:MGI:1335087]	1.063	4.40E-02
ENSMUSG00000022781	Pak2	p21 (RAC1) activated kinase 2 [Source:MGI Symbol;Acc:MGI:1339984]	1.061	3.98E-02
ENSMUSG00000026017	Carf	calcium response factor [Source:MGI Symbol;Acc:MGI:2182269]	1.061	4.29E-02
ENSMUSG00000017664	Slc35c2	"solute carrier family 35, member C2 [Source:MGI Symbol;Acc:MGI:2385166]"	1.060	2.84E-02
ENSMUSG00000016481	Cr1l	complement component (3b/4b) receptor 1-like [Source:MGI Symbol;Acc:MGI:88513]	1.059	6.91E-03
ENSMUSG00000024472	Dcp2	decapping mRNA 2 [Source:MGI Symbol;Acc:MGI:1917890]	1.058	4.46E-02
ENSMUSG00000062202	Btbd9	BTB (POZ) domain containing 9 [Source:MGI Symbol;Acc:MGI:1916625]	1.057	1.09E-02
ENSMUSG00000005547	Cyp2a5	"cytochrome P450, family 2, subfamily a, polypeptide 5 [Source:MGI Symbol;Acc:MGI:88597]"	1.057	1.79E-02
ENSMUSG00000055531	Cpsf6	cleavage and polyadenylation specific factor 6 [Source:MGI Symbol;Acc:MGI:1913948]	1.056	4.41E-02
ENSMUSG00000039361	Picalm	phosphatidylinositol binding clathrin assembly protein [Source:MGI Symbol;Acc:MGI:2385902]	1.052	1.35E-03
ENSMUSG00000039220	Ppp1r10	"protein phosphatase 1, regulatory subunit 10 [Source:MGI Symbol;Acc:MGI:1289273]"	1.051	2.93E-02
ENSMUSG00000069539	Scyl2	SCY1-like 2 (S. cerevisiae) [Source:MGI Symbol;Acc:MGI:1289172]	1.051	4.89E-02
ENSMUSG00000020190	Mknk2	MAP kinase-interacting serine/threonine kinase 2 [Source:MGI Symbol;Acc:MGI:894279]	1.051	2.62E-02
ENSMUSG00000030779	Rbbp6	"retinoblastoma binding protein 6, ubiquitin ligase [Source:MGI Symbol;Acc:MGI:894835]"	1.050	7.04E-03
ENSMUSG00000056211	R3hdm1	R3H domain containing 1 [Source:MGI Symbol;Acc:MGI:2448514]	1.048	5.43E-03
ENSMUSG00000057367	Birc2	baculoviral IAP repeat-containing 2 [Source:MGI Symbol;Acc:MGI:1197009]	1.044	1.36E-02
ENSMUSG00000048109	Rbm15	RNA binding motif protein 15 [Source:MGI Symbol;Acc:MGI:2443205]	1.043	4.24E-02
ENSMUSG00000020863	Luc7l3	LUC7-like 3 (S. cerevisiae) [Source:MGI Symbol;Acc:MGI:1914934]	1.043	4.31E-02

ENSMUSG00000015087	Rabl6	"RAB, member RAS oncogene family-like 6 [Source:MGI Symbol;Acc:MGI:2442633]"	1.041	2.19E-02
ENSMUSG00000001525	Tubb5	"tubulin, beta 5 class I [Source:MGI Symbol;Acc:MGI:107812]"	1.039	3.18E-02
ENSMUSG00000026131	Dst	dystonin [Source:MGI Symbol;Acc:MGI:104627]	1.036	4.90E-03
ENSMUSG00000000194	Gpr107	G protein-coupled receptor 107 [Source:MGI Symbol;Acc:MGI:2139054]	1.036	3.83E-02
ENSMUSG00000035851	Ythdc1	YTH domain containing 1 [Source:MGI Symbol;Acc:MGI:2443713]	1.033	2.76E-02
ENSMUSG00000027615	Hps3	"HPS3, biogenesis of lysosomal organelles complex 2 subunit 1 [Source:MGI Symbol;Acc:MGI:2153839]"	1.033	2.60E-02
ENSMUSG00000033808	Tmem87a	transmembrane protein 87A [Source:MGI Symbol;Acc:MGI:2441844]	1.026	3.72E-02
ENSMUSG00000052459	Atp6v1a	"ATPase, H+ transporting, lysosomal V1 subunit A [Source:MGI Symbol;Acc:MGI:1201780]"	1.022	4.98E-02
ENSMUSG00000020952	Scfd1	Sec1 family domain containing 1 [Source:MGI Symbol;Acc:MGI:1924233]	1.022	5.27E-03
ENSMUSG00000035401	Emsy	"EMSY, BRCA2-interacting transcriptional repressor [Source:MGI Symbol;Acc:MGI:1924203]"	1.019	3.92E-02
ENSMUSG00000022707	Gbe1	"glucan (1,4-alpha-), branching enzyme 1 [Source:MGI Symbol;Acc:MGI:1921435]"	1.017	3.74E-02
ENSMUSG00000046352	Gjb2	"gap junction protein, beta 2 [Source:MGI Symbol;Acc:MGI:95720]"	1.014	1.32E-02
ENSMUSG00000030870	Ubfd1	ubiquitin family domain containing 1 [Source:MGI Symbol;Acc:MGI:107301]	1.012	1.25E-02
ENSMUSG00000060550	H2-Q7	"histocompatibility 2, Q region locus 7 [Source:MGI Symbol;Acc:MGI:95936]"	1.011	1.49E-03
ENSMUSG00000038784	Cnot4	"CCR4-NOT transcription complex, subunit 4 [Source:MGI Symbol;Acc:MGI:1859026]"	1.007	4.36E-02
ENSMUSG00000046598	Bdh1	"3-hydroxybutyrate dehydrogenase, type 1 [Source:MGI Symbol;Acc:MGI:1919161]"	1.003	3.79E-02
ENSMUSG00000063108	Zfp26	zinc finger protein 26 [Source:MGI Symbol;Acc:MGI:99173]	1.001	1.61E-02
ENSMUSG00000015806	Qdpr	quinoid dihydropteridine reductase [Source:MGI Symbol;Acc:MGI:97836]	-1.002	2.83E-25
ENSMUSG00000116097	Gm36738	"predicted gene, 36738 [Source:MGI Symbol;Acc:MGI:5595897]"	-1.002	3.09E-02
ENSMUSG00000028716	Pdzk1ip1	PDZK1 interacting protein 1 [Source:MGI Symbol;Acc:MGI:1914432]	-1.003	1.69E-02
ENSMUSG00000083061	Gm12191	predicted gene 12191 [Source:MGI Symbol;Acc:MGI:3651293]	-1.005	4.82E-09
ENSMUSG00000089960	Ugt1a1	"UDP glucuronosyltransferase 1 family, polypeptide A1 [Source:MGI Symbol;Acc:MGI:98898]"	-1.005	2.55E-12
ENSMUSG00000020017	Hal	histidine ammonia lyase [Source:MGI Symbol;Acc:MGI:96010]	-1.011	1.17E-20
ENSMUSG00000064350	mt-Ty	mitochondrially encoded tRNA tyrosine [Source:MGI Symbol;Acc:MGI:102470]	-1.022	1.03E-09
ENSMUSG00000017868	Sgk2	serum/glucocorticoid regulated kinase 2 [Source:MGI Symbol;Acc:MGI:1351318]	-1.023	9.14E-14
ENSMUSG00000018796	Acsl1	acyl-CoA synthetase long-chain family member 1 [Source:MGI Symbol;Acc:MGI:102797]	-1.025	2.78E-29
ENSMUSG00000015357	Clpx	caseinolytic mitochondrial matrix peptidase chaperone subunit [Source:MGI Symbol;Acc:MGI:1346017]	-1.026	4.60E-27
ENSMUSG00000039620	Trmt9b	tRNA methyltransferase 9B [Source:MGI Symbol;Acc:MGI:2442328]	-1.028	1.80E-04
ENSMUSG00000064254	Ethe1	ethylmalonic encephalopathy 1 [Source:MGI Symbol;Acc:MGI:1913321]	-1.029	4.49E-17
ENSMUSG00000037686	Aspg	asparaginase [Source:MGI Symbol;Acc:MGI:2144822]	-1.040	7.85E-09
ENSMUSG00000062582	Rpl30-ps8	"ribosomal protein L30, pseudogene 8 [Source:MGI Symbol;Acc:MGI:3643509]"	-1.042	7.80E-05
ENSMUSG00000020182	Ddc	dopa decarboxylase [Source:MGI Symbol;Acc:MGI:94876]	-1.048	1.96E-22
ENSMUSG00000063929	Cyp4a32	"cytochrome P450, family 4, subfamily a, polypeptide 32 [Source:MGI Symbol;Acc:MGI:3717148]"	-1.048	4.70E-06
ENSMUSG00000113637	Gm7049	predicted gene 7049 [Source:MGI Symbol;Acc:MGI:3646400]	-1.055	3.04E-04
ENSMUSG00000029195	Klb	klotho beta [Source:MGI Symbol;Acc:MGI:1932466]	-1.060	2.66E-08
ENSMUSG00000031767	Nudt7	nudix (nucleoside diphosphate linked moiety X)-type motif 7 [Source:MGI Symbol;Acc:MGI:1914778]	-1.061	2.41E-12
ENSMUSG00000097673	Gm26608	"predicted gene, 26608 [Source:MGI Symbol;Acc:MGI:5477102]"	-1.068	4.52E-02
ENSMUSG00000028354	Fmn2	formin 2 [Source:MGI Symbol;Acc:MGI:1859252]	-1.072	1.68E-02
ENSMUSG00000029664	Tfpi2	tissue factor pathway inhibitor 2 [Source:MGI Symbol;Acc:MGI:108543]	-1.077	3.15E-09
ENSMUSG00000001763	Tspan33	tetraspanin 33 [Source:MGI Symbol;Acc:MGI:1919012]	-1.079	1.14E-06
ENSMUSG00000043681	Fam25c	"family with sequence similarity 25, member C [Source:MGI Symbol;Acc:MGI:1916384]"	-1.080	1.07E-06
ENSMUSG00000108599	Gm10616	predicted gene 10616 [Source:MGI Symbol;Acc:MGI:3642103]	-1.092	1.41E-02
ENSMUSG00000090038	Gm16573	predicted gene 16573 [Source:MGI Symbol;Acc:MGI:4414993]	-1.093	2.29E-02
ENSMUSG00000063590	Slc22a28	"solute carrier family 22, member 28 [Source:MGI Symbol;Acc:MGI:3645714]"	-1.094	6.77E-09
ENSMUSG00000109291	Gm2814	predicted gene 2814 [Source:MGI Symbol;Acc:MGI:3780984]	-1.105	6.02E-05
ENSMUSG00000023800	Tiam2	T cell lymphoma invasion and metastasis 2 [Source:MGI Symbol;Acc:MGI:1344338]	-1.105	4.73E-06
ENSMUSG00000079164	Tlr5	toll-like receptor 5 [Source:MGI Symbol;Acc:MGI:1858171]	-1.107	1.84E-05
ENSMUSG00000059064	Gm10059	predicted pseudogene 10059 [Source:MGI Symbol;Acc:MGI:3642158]	-1.111	4.96E-03
ENSMUSG00000061718	Ppp1r1b	"protein phosphatase 1, regulatory inhibitor subunit 1B [Source:MGI Symbol;Acc:MGI:94860]"	-1.117	2.50E-03
ENSMUSG00000112774	Gm36041	"predicted gene, 36041 [Source:MGI Symbol;Acc:MGI:5595200]"	-1.117	3.74E-07

ENSMUSG00000106560	Gm43119	predicted gene 43119 [Source:MGI Symbol;Acc:MGI:5663256]	-1.117	1.28E-04
ENSMUSG00000048538	Gm9826	predicted gene 9826 [Source:MGI Symbol;Acc:MGI:3642725]	-1.125	4.68E-18
ENSMUSG00000056749	Nfil3	"nuclear factor, interleukin 3, regulated [Source:MGI Symbol;Acc:MGI:109495]"	-1.131	2.02E-04
ENSMUSG00000115756	Gm49519	"predicted gene, 49519 [Source:MGI Symbol;Acc:MGI:6155213]"	-1.133	4.07E-02
ENSMUSG00000033585	Ndn	necdin [Source:MGI Symbol;Acc:MGI:97290]	-1.133	4.05E-02
ENSMUSG00000020027	Socs2	suppressor of cytokine signaling 2 [Source:MGI Symbol;Acc:MGI:1201787]	-1.134	4.86E-08
ENSMUSG00000108436	Gm44851	predicted gene 44851 [Source:MGI Symbol;Acc:MGI:5753427]	-1.138	2.05E-03
ENSMUSG00000096891	Mup-ps8	"major urinary protein, pseudogene 8 [Source:MGI Symbol;Acc:MGI:3780197]"	-1.139	8.67E-05
ENSMUSG00000044349	Snhg11	small nucleolar RNA host gene 11 [Source:MGI Symbol;Acc:MGI:2441845]	-1.140	5.15E-04
ENSMUSG00000043439	Epop	elongin BC and polycomb repressive complex 2 associated protein [Source:MGI Symbol;Acc:MGI:2143991]	-1.151	3.61E-02
ENSMUSG00000083409	Gm11340	predicted gene 11340 [Source:MGI Symbol;Acc:MGI:3652096]	-1.155	4.26E-03
ENSMUSG00000019989	Enpp3	ectonucleotide pyrophosphatase/phosphodiesterase 3 [Source:MGI Symbol;Acc:MGI:2143702]	-1.157	7.01E-19
ENSMUSG00000071178	Serpina1b	"serine (or cysteine) preptidase inhibitor, clade A, member 1B [Source:MGI Symbol;Acc:MGI:891970]"	-1.158	3.55E-22
ENSMUSG00000066629	Rpl36-ps3	"ribosomal protein L36, pseudogene 3 [Source:MGI Symbol;Acc:MGI:3642480]"	-1.160	2.04E-03
ENSMUSG00000030378	Sult2a8	"sulfotransferase family 2A, dehydroepiandrosterone (DHEA)-preferring, member 8 [Source:MGI Symbol;Acc:MGI:1924221]"	-1.161	5.22E-13
ENSMUSG00000112796	Gm40770	"predicted gene, 40770 [Source:MGI Symbol;Acc:MGI:5623655]"	-1.165	4.43E-05
ENSMUSG00000058260	Serpina9	"serine (or cysteine) peptidase inhibitor, clade A (alpha-1 antiproteinase, antitrypsin), member 9 [Source:MGI Symbol;Acc:MGI:1919157]"	-1.167	4.51E-02
ENSMUSG00000057863	Rpl36	ribosomal protein L36 [Source:MGI Symbol;Acc:MGI:1860603]	-1.174	1.71E-12
ENSMUSG00000042248	Cyp2c37	"cytochrome P450, family 2. subfamily c, polypeptide 37 [Source:MGI Symbol;Acc:MGI:1306806]"	-1.178	4.66E-12
ENSMUSG00000068686	Cd59b	CD59b antigen [Source:MGI Symbol;Acc:MGI:1888996]	-1.185	1.18E-02
ENSMUSG00000032482	Cspg5	chondroitin sulfate proteoglycan 5 [Source:MGI Symbol;Acc:MGI:1352747]	-1.208	8.04E-03
ENSMUSG00000062647	Rpl7a	ribosomal protein L7A [Source:MGI Symbol;Acc:MGI:1353472]	-1.227	4.66E-06
ENSMUSG00000027950	Chrnb2	"cholinergic receptor, nicotinic, beta polypeptide 2 (neuronal) [Source:MGI Symbol;Acc:MGI:87891]"	-1.227	2.00E-02
ENSMUSG00000064349	mt-Tc	mitochondrially encoded tRNA cysteine [Source:MGI Symbol;Acc:MGI:102490]	-1.229	1.25E-06
ENSMUSG00000101517	4732465J04Rik	RIKEN cDNA 4732465J04 gene [Source:MGI Symbol;Acc:MGI:3041208]	-1.237	1.83E-09
ENSMUSG00000026077	Npas2	neuronal PAS domain protein 2 [Source:MGI Symbol;Acc:MGI:109232]	-1.245	6.68E-05
ENSMUSG00000025936	Gm4956	predicted gene 4956 [Source:MGI Symbol;Acc:MGI:3647976]	-1.248	4.67E-10
ENSMUSG00000034429	Zfp707	zinc finger protein 707 [Source:MGI Symbol;Acc:MGI:1916270]	-1.249	2.90E-05
ENSMUSG00000086054	Hnf1aos1	"HNF1 homeobox A, opposite strand 1 [Source:MGI Symbol;Acc:MGI:3652225]"	-1.253	9.69E-04
ENSMUSG00000060636	Rpl35a	ribosomal protein L35A [Source:MGI Symbol;Acc:MGI:1928894]	-1.254	1.29E-08
ENSMUSG00000085995	Gm2788	predicted gene 2788 [Source:MGI Symbol;Acc:MGI:3780956]	-1.262	1.24E-11
ENSMUSG00000022679	Mpv17l	"Mpv17 transgene, kidney disease mutant-like [Source:MGI Symbol;Acc:MGI:2135951]"	-1.263	1.74E-12
ENSMUSG00000062181	Ces3b	carboxylesterase 3B [Source:MGI Symbol;Acc:MGI:3644960]	-1.267	3.50E-21
ENSMUSG00000090306	Adh6-ps1	"alcohol dehydrogenase 6 (class V), pseudogene 1 [Source:MGI Symbol;Acc:MGI:1918999]"	-1.272	9.29E-17
ENSMUSG00000022615	Tymp	thymidine phosphorylase [Source:MGI Symbol;Acc:MGI:1920212]	-1.272	1.25E-12
ENSMUSG00000056973	Ces1d	carboxylesterase 1D [Source:MGI Symbol;Acc:MGI:2148202]	-1.273	2.58E-11
ENSMUSG00000037826	Ppm1k	protein phosphatase 1K (PP2C domain containing) [Source:MGI Symbol;Acc:MGI:2442111]	-1.281	1.11E-26
ENSMUSG00000035836	Ugt2b1	"UDP glucuronosyltransferase 2 family, polypeptide B1 [Source:MGI Symbol;Acc:MGI:1919023]"	-1.289	2.22E-23
ENSMUSG00000078963	Hsbp1l1	heat shock factor binding protein 1-like 1 [Source:MGI Symbol;Acc:MGI:1913505]	-1.290	2.41E-02
ENSMUSG00000105192	Gm42796	predicted gene 42796 [Source:MGI Symbol;Acc:MGI:5662933]	-1.291	2.01E-02
ENSMUSG00000043687	1190005I06Rik	RIKEN cDNA 1190005I06 gene [Source:MGI Symbol;Acc:MGI:1916168]	-1.293	8.30E-04
ENSMUSG00000117501	Gm30117	"predicted gene, 30117 [Source:MGI Symbol;Acc:MGI:5589276]"	-1.295	1.79E-02
ENSMUSG00000024799	Tm7sf2	transmembrane 7 superfamily member 2 [Source:MGI Symbol;Acc:MGI:1920416]	-1.300	5.68E-15
ENSMUSG00000099242	Obox4-ps3	"oocyte specific homeobox 4, pseudogene 3 [Source:MGI Symbol;Acc:MGI:5521023]"	-1.303	8.52E-06
ENSMUSG00000040264	Gbp2b	guanylate binding protein 2b [Source:MGI Symbol;Acc:MGI:95666]	-1.304	2.04E-02
ENSMUSG00000110035	Gm30931	"predicted gene, 30931 [Source:MGI Symbol;Acc:MGI:5590090]"	-1.314	1.52E-02
ENSMUSG00000058207	Serpina3k	"serine (or cysteine) peptidase inhibitor, clade A, member 3K [Source:MGI Symbol;Acc:MGI:98377]"	-1.318	3.78E-20
ENSMUSG00000028167	Bdh2	"3-hydroxybutyrate dehydrogenase, type 2 [Source:MGI Symbol;Acc:MGI:1917022]"	-1.318	1.17E-08
ENSMUSG00000091780	Sco2	SCO2 cytochrome c oxidase assembly protein [Source:MGI Symbol;Acc:MGI:3818630]	-1.328	1.97E-11
ENSMUSG00000067225	Cyp2c54	"cytochrome P450, family 2, subfamily c, polypeptide 54 [Source:MGI Symbol;Acc:MGI:3642960]"	-1.345	4.57E-16

ENSMUSG00000026839	Upp2	uridine phosphorylase 2 [Source:MGI Symbol;Acc:MGI:1923904]	-1.351	6.36E-13
ENSMUSG00000038217	Tlcd2	TLC domain containing 2 [Source:MGI Symbol;Acc:MGI:1917141]	-1.365	2.33E-11
ENSMUSG00000022550	Adck5	aarF domain containing kinase 5 [Source:MGI Symbol;Acc:MGI:2679274]	-1.373	1.50E-06
ENSMUSG00000060314	Zfp941	zinc finger protein 941 [Source:MGI Symbol;Acc:MGI:3039601]	-1.374	7.37E-03
ENSMUSG00000100094	1810008I18Rik	RIKEN cDNA 1810008I18 gene [Source:MGI Symbol;Acc:MGI:1920875]	-1.375	7.60E-23
ENSMUSG00000042686	Jph1	junctophilin 1 [Source:MGI Symbol;Acc:MGI:1891495]	-1.379	5.73E-04
ENSMUSG00000110439	Mup22	major urinary protein 22 [Source:MGI Symbol;Acc:MGI:5434675]	-1.380	4.81E-16
ENSMUSG00000113543	Gm36264	"predicted gene, 36264 [Source:MGI Symbol;Acc:MGI:5595423]"	-1.380	9.65E-04
ENSMUSG00000078817	Nlrp12	"NLR family, pyrin domain containing 12 [Source:MGI Symbol;Acc:MGI:2676630]"	-1.382	7.35E-16
ENSMUSG00000064371	mt-Tt	mitochondrially encoded tRNA threonine [Source:MGI Symbol;Acc:MGI:102473]	-1.384	1.26E-03
ENSMUSG00000022236	Ropn1l	ropporin 1-like [Source:MGI Symbol;Acc:MGI:2182357]	-1.399	1.17E-04
ENSMUSG00000032418	Me1	"malic enzyme 1, NADP(+)-dependent, cytosolic [Source:MGI Symbol;Acc:MGI:97043]"	-1.400	4.55E-41
ENSMUSG00000109599	Gm31812	"predicted gene, 31812 [Source:MGI Symbol;Acc:MGI:5590971]"	-1.408	2.09E-02
ENSMUSG00000030731	Syt3	synaptotagmin III [Source:MGI Symbol;Acc:MGI:99665]	-1.408	4.75E-04
ENSMUSG00000030340	Scnn1a	"sodium channel, nonvoltage-gated 1 alpha [Source:MGI Symbol;Acc:MGI:101782]"	-1.411	7.50E-14
ENSMUSG00000041698	Slco1a1	"solute carrier organic anion transporter family, member 1a1 [Source:MGI Symbol;Acc:MGI:1351891]"	-1.416	6.22E-13
ENSMUSG00000074219	Gm10644	predicted gene 10644 [Source:MGI Symbol;Acc:MGI:3704314]	-1.441	2.57E-02
ENSMUSG00000030470	Csrp3	cysteine and glycine-rich protein 3 [Source:MGI Symbol;Acc:MGI:1330824]	-1.444	5.27E-07
ENSMUSG00000109089	4833411C07Rik	RIKEN cDNA 4833411C07 gene [Source:MGI Symbol;Acc:MGI:1918874]	-1.448	9.38E-09
ENSMUSG00000090555	Gm8893	predicted gene 8893 [Source:MGI Symbol;Acc:MGI:3779818]	-1.449	9.59E-25
ENSMUSG00000024694	Keg1	kidney expressed gene 1 [Source:MGI Symbol;Acc:MGI:1928492]	-1.451	1.40E-11
ENSMUSG00000064372	mt-Tp	mitochondrially encoded tRNA proline [Source:MGI Symbol;Acc:MGI:102478]	-1.465	6.49E-04
ENSMUSG00000070271	Gm13268	predicted gene 13268 [Source:MGI Symbol;Acc:MGI:3651947]	-1.469	4.10E-02
ENSMUSG00000065016	Snora3	"small nucleolar RNA, H/ACA box 3 [Source:MGI Symbol;Acc:MGI:3819498]"	-1.480	3.53E-02
ENSMUSG00000052632	Asap2	"ArfGAP with SH3 domain, ankyrin repeat and PH domain 2 [Source:MGI Symbol;Acc:MGI:2685438]"	-1.503	1.52E-19
ENSMUSG00000086938	4930481A15Rik	RIKEN cDNA 4930481A15 gene [Source:MGI Symbol;Acc:MGI:1922181]	-1.508	1.68E-06
ENSMUSG00000103653	Gstp-ps	"glutathione S-transferase, pi, pseudogene [Source:MGI Symbol;Acc:MGI:3782108]"	-1.508	4.59E-25
ENSMUSG00000023078	Cxcl13	chemokine (C-X-C motif) ligand 13 [Source:MGI Symbol;Acc:MGI:1888499]	-1.512	1.59E-02
ENSMUSG00000027863	Cd2	CD2 antigen [Source:MGI Symbol;Acc:MGI:88320]	-1.522	2.74E-02
ENSMUSG00000031637	Lrp2bp	Lrp2 binding protein [Source:MGI Symbol;Acc:MGI:1914870]	-1.535	1.88E-02
ENSMUSG00000107997	Gm44243	"predicted gene, 44243 [Source:MGI Symbol;Acc:MGI:5690635]"	-1.538	3.39E-03
ENSMUSG00000078674	Mup18	major urinary protein 18 [Source:MGI Symbol;Acc:MGI:3705220]	-1.547	1.00E-16
ENSMUSG00000038599	Capn8	calpain 8 [Source:MGI Symbol;Acc:MGI:2181366]	-1.551	1.64E-02
ENSMUSG00000048489	Depp1	DEPP1 autophagy regulator [Source:MGI Symbol;Acc:MGI:1918730]	-1.551	4.01E-12
ENSMUSG00000038768	9130409I23Rik	RIKEN cDNA 9130409I23 gene [Source:MGI Symbol;Acc:MGI:3588271]	-1.556	3.57E-15
ENSMUSG00000042010	Acacb	acetyl-Coenzyme A carboxylase beta [Source:MGI Symbol;Acc:MGI:2140940]	-1.563	2.74E-19
ENSMUSG00000066366	Serpina1a	"serine (or cysteine) peptidase inhibitor, clade A, member 1A [Source:MGI Symbol;Acc:MGI:891971]"	-1.565	1.11E-28
ENSMUSG00000110234	Gm45799	predicted gene 45799 [Source:MGI Symbol;Acc:MGI:5804914]	-1.568	2.20E-02
ENSMUSG00000066477	Gm16551	predicted gene 16551 [Source:MGI Symbol;Acc:MGI:4414971]	-1.571	1.91E-04
ENSMUSG00000029630	Cyp3a25	"cytochrome P450, family 3, subfamily a, polypeptide 25 [Source:MGI Symbol;Acc:MGI:1930638]"	-1.579	4.35E-13
ENSMUSG00000061959	Ces1e	carboxylesterase 1E [Source:MGI Symbol;Acc:MGI:95432]	-1.589	2.14E-16
ENSMUSG00000031725	Ces1f	carboxylesterase 1F [Source:MGI Symbol;Acc:MGI:2142687]	-1.597	1.95E-24
ENSMUSG00000105025	Rnu3b1	U3B small nuclear RNA 1 [Source:MGI Symbol;Acc:MGI:97985]	-1.600	5.43E-03
ENSMUSG00000060803	Gstp1	"glutathione S-transferase, pi 1 [Source:MGI Symbol;Acc:MGI:95865]"	-1.604	1.60E-32
ENSMUSG00000071177	Serpina1d	"serine (or cysteine) peptidase inhibitor, clade A, member 1D [Source:MGI Symbol;Acc:MGI:891968]"	-1.609	1.87E-30
ENSMUSG00000030364	Clec2h	"C-type lectin domain family 2, member h [Source:MGI Symbol;Acc:MGI:2136934]"	-1.610	1.05E-13
ENSMUSG00000073830	Mup14	major urinary protein 14 [Source:MGI Symbol;Acc:MGI:3702005]	-1.610	1.31E-23
ENSMUSG00000089873	Mup13	major urinary protein 13 [Source:MGI Symbol;Acc:MGI:3702003]	-1.613	3.61E-22
ENSMUSG00000068086	Cyp2d9	"cytochrome P450, family 2, subfamily d, polypeptide 9 [Source:MGI Symbol;Acc:MGI:88606]"	-1.628	3.14E-31
ENSMUSG00000078680	Mup10	major urinary protein 10 [Source:MGI Symbol;Acc:MGI:1924164]	-1.635	1.37E-04

ENSMUSG00000053141	Ptprt	"protein tyrosine phosphatase, receptor type, T [Source:MGI Symbol;Acc:MGI:1321152]"	-1.635	7.75E-03
ENSMUSG00000030701	Plekhb1	"pleckstrin homology domain containing, family B (evectins) member 1 [Source:MGI Symbol;Acc:MGI:1351469]"	-1.640	2.99E-13
ENSMUSG00000100075	1700018L02Rik	RIKEN cDNA 1700018L02 gene [Source:MGI Symbol;Acc:MGI:1914579]	-1.645	1.65E-04
ENSMUSG00000022508	Bcl6	B cell leukemia/lymphoma 6 [Source:MGI Symbol;Acc:MGI:107187]	-1.652	1.25E-02
ENSMUSG00000042851	Zc3h6	zinc finger CCCH type containing 6 [Source:MGI Symbol;Acc:MGI:1926001]	-1.652	5.64E-06
ENSMUSG00000022982	Sod1	"superoxide dismutase 1, soluble [Source:MGI Symbol;Acc:MGI:98351]"	-1.660	6.31E-30
ENSMUSG00000044122	Proca1	protein interacting with cyclin A1 [Source:MGI Symbol;Acc:MGI:1918274]	-1.672	4.78E-03
ENSMUSG00000096726	Gm5558	predicted gene 5558 [Source:MGI Symbol;Acc:MGI:3644616]	-1.672	4.41E-02
ENSMUSG00000027577	Chrna4	"cholinergic receptor, nicotinic, alpha polypeptide 4 [Source:MGI Symbol;Acc:MGI:87888]"	-1.679	2.68E-02
ENSMUSG00000024211	Grm8	"glutamate receptor, metabotropic 8 [Source:MGI Symbol;Acc:MGI:1351345]"	-1.680	8.45E-05
ENSMUSG00000022763	Aifm3	"apoptosis-inducing factor, mitochondrion-associated 3 [Source:MGI Symbol;Acc:MGI:1919418]"	-1.681	4.71E-05
ENSMUSG00000049685	Cyp2g1	"cytochrome P450, family 2, subfamily g, polypeptide 1 [Source:MGI Symbol;Acc:MGI:109612]"	-1.682	3.61E-02
ENSMUSG00000066153	Mup21	major urinary protein 21 [Source:MGI Symbol;Acc:MGI:3650630]	-1.692	1.09E-13
ENSMUSG00000083649	Rasl2-9	"RAS-like, family 2, locus 9 [Source:MGI Symbol;Acc:MGI:104605]"	-1.700	2.52E-02
ENSMUSG00000038155	Gstp2	"glutathione S-transferase, pi 2 [Source:MGI Symbol;Acc:MGI:95864]"	-1.701	2.34E-48
ENSMUSG00000023044	Csad	cysteine sulfinic acid decarboxylase [Source:MGI Symbol;Acc:MGI:2180098]	-1.705	1.58E-25
ENSMUSG00000038415	Foxq1	forkhead box Q1 [Source:MGI Symbol;Acc:MGI:1298228]	-1.719	4.19E-04
ENSMUSG00000111055	D030034A15Rik	RIKEN cDNA D030034A15 gene [Source:MGI Symbol;Acc:MGI:2442288]	-1.719	2.84E-02
ENSMUSG00000038576	Susd4	sushi domain containing 4 [Source:MGI Symbol;Acc:MGI:2138351]	-1.722	7.40E-08
ENSMUSG00000079015	Serpina1c	"serine (or cysteine) peptidase inhibitor, clade A, member 1C [Source:MGI Symbol;Acc:MGI:891969]"	-1.738	1.06E-26
ENSMUSG00000020435	Osbp2	oxysterol binding protein 2 [Source:MGI Symbol;Acc:MGI:1921559]	-1.753	3.70E-02
ENSMUSG00000100774	Gm7329	predicted gene 7329 [Source:MGI Symbol;Acc:MGI:3646271]	-1.773	2.35E-08
ENSMUSG00000118631	AL590864.1	"novel transcript, antisense to Cmah"	-1.778	8.96E-08
ENSMUSG00000107552	Gm44096	"predicted gene, 44096 [Source:MGI Symbol;Acc:MGI:5690488]"	-1.787	2.31E-02
ENSMUSG00000102101	Zbtb11os1	"zinc finger and BTB domain containing 11, opposite strand 1 [Source:MGI Symbol;Acc:MGI:1913641]"	-1.794	1.18E-03
ENSMUSG00000015224	Cyp2j9	"cytochrome P450, family 2, subfamily j, polypeptide 9 [Source:MGI Symbol;Acc:MGI:1921769]"	-1.804	1.70E-06
ENSMUSG00000054793	Cadm4	cell adhesion molecule 4 [Source:MGI Symbol;Acc:MGI:2449088]	-1.822	4.32E-05
ENSMUSG00000033107	Rnf125	ring finger protein 125 [Source:MGI Symbol;Acc:MGI:1914914]	-1.825	8.90E-32
ENSMUSG00000017718	Afmid	arylformamidase [Source:MGI Symbol;Acc:MGI:2448704]	-1.832	1.68E-11
ENSMUSG00000021884	Hacl1	2-hydroxyacyl-CoA lyase 1 [Source:MGI Symbol;Acc:MGI:1929657]	-1.840	8.25E-20
ENSMUSG00000043648	Pld6	"phospholipase D family, member 6 [Source:MGI Symbol;Acc:MGI:2687283]"	-1.853	1.62E-02
ENSMUSG00000040035	Disp2	dispatched RND tramsporter family member 2 [Source:MGI Symbol;Acc:MGI:2388733]	-1.860	1.73E-07
ENSMUSG00000003477	Inmt	indolethylamine N-methyltransferase [Source:MGI Symbol;Acc:MGI:102963]	-1.867	5.61E-32
ENSMUSG00000087579	Hectd2os	"Hectd2, opposite strand [Source:MGI Symbol;Acc:MGI:1919243]"	-1.867	2.24E-17
ENSMUSG00000044378	Slc15a5	"solute carrier family 15, member 5 [Source:MGI Symbol;Acc:MGI:3607714]"	-1.903	1.03E-06
ENSMUSG00000026489	Coq8a	coenzyme Q8A [Source:MGI Symbol;Acc:MGI:1914676]	-1.910	1.48E-43
ENSMUSG00000054191	Klf1	Kruppel-like factor 1 (erythroid) [Source:MGI Symbol;Acc:MGI:1342771]	-1.913	6.61E-03
ENSMUSG00000089943	Ugt1a5	"UDP glucuronosyltransferase 1 family, polypeptide A5 [Source:MGI Symbol;Acc:MGI:3032634]"	-1.926	4.49E-11
ENSMUSG00000086253	Gm13773	predicted gene 13773 [Source:MGI Symbol;Acc:MGI:3712316]	-1.965	1.03E-04
ENSMUSG00000078688	Mup2	major urinary protein 2 [Source:MGI Symbol;Acc:MGI:97234]	-1.968	2.88E-23
ENSMUSG00000112880	Gm20337	"predicted gene, 20337 [Source:MGI Symbol;Acc:MGI:5012522]"	-1.970	4.60E-02
ENSMUSG00000001420	Tmem79	transmembrane protein 79 [Source:MGI Symbol;Acc:MGI:1919163]	-1.977	1.02E-02
ENSMUSG00000092274	Neat1	nuclear paraspeckle assembly transcript 1 (non-protein coding) [Source:MGI Symbol;Acc:MGI:1914211]	-1.995	1.72E-05
ENSMUSG00000030945	Acsm2	acyl-CoA synthetase medium-chain family member 2 [Source:MGI Symbol;Acc:MGI:2385289]	-2.005	3.99E-03
ENSMUSG00000066154	Mup3	major urinary protein 3 [Source:MGI Symbol;Acc:MGI:97235]	-2.029	1.00E-39
ENSMUSG00000107516	Gm30784	"predicted gene, 30784 [Source:MGI Symbol;Acc:MGI:5589943]"	-2.029	5.10E-05
ENSMUSG00000056035	Cyp3a11	"cytochrome P450, family 3, subfamily a, polypeptide 11 [Source:MGI Symbol;Acc:MGI:88609]"	-2.052	2.14E-48
ENSMUSG00000113063	Gm34667	"predicted gene, 34667 [Source:MGI Symbol;Acc:MGI:5593826]"	-2.069	1.99E-08
ENSMUSG00000027556	Car1	carbonic anhydrase 1 [Source:MGI Symbol;Acc:MGI:88268]	-2.069	1.56E-07
ENSMUSG00000039519	Cyp7b1	"cytochrome P450, family 7, subfamily b, polypeptide 1 [Source:MGI Symbol;Acc:MGI:104978]"	-2.132	1.93E-26

ENSMUSG00000105837	Gm35986	"predicted gene, 35986 [Source:MGI Symbol;Acc:MGI:5595145]"	-2.145	2.03E-02
ENSMUSG00000049353	Rd3	retinal degeneration 3 [Source:MGI Symbol;Acc:MGI:1921273]	-2.153	3.17E-04
ENSMUSG00000046840	Hnf4aos	"hepatic nuclear factor 4 alpha, opposite strand [Source:MGI Symbol;Acc:MGI:1915564]"	-2.160	9.30E-05
ENSMUSG00000047150	1700001C19Rik	RIKEN cDNA 1700001C19 gene [Source:MGI Symbol;Acc:MGI:1922712]	-2.168	5.06E-13
ENSMUSG00000065952	Rps23rg1	"ribosomal protein S23, retrogene 1 [Source:MGI Symbol;Acc:MGI:3612471]"	-2.191	7.69E-04
ENSMUSG00000022129	Dct	dopachrome tautomerase [Source:MGI Symbol;Acc:MGI:102563]	-2.192	9.14E-04
ENSMUSG00000089712	Gm15889	predicted gene 15889 [Source:MGI Symbol;Acc:MGI:3802159]	-2.204	9.87E-03
ENSMUSG00000025175	Fn3k	fructosamine 3 kinase [Source:MGI Symbol;Acc:MGI:1926834]	-2.219	2.32E-07
ENSMUSG00000085923	Gm12781	predicted gene 12781 [Source:MGI Symbol;Acc:MGI:3649569]	-2.225	2.05E-03
ENSMUSG00000050195	Scd4	stearoyl-coenzyme A desaturase 4 [Source:MGI Symbol;Acc:MGI:2670997]	-2.364	2.53E-04
ENSMUSG00000061292	Cyp3a59	"cytochrome P450, family 3, subfamily a, polypeptide 59 [Source:MGI Symbol;Acc:MGI:3769707]"	-2.375	1.01E-13
ENSMUSG00000099032	Tcf24	transcription factor 24 [Source:MGI Symbol;Acc:MGI:3780500]	-2.391	1.33E-07
ENSMUSG00000112278	Gm30025	"predicted gene, 30025 [Source:MGI Symbol;Acc:MGI:5589184]"	-2.397	1.13E-02
ENSMUSG00000018727	Cpsf4l	cleavage and polyadenylation specific factor 4-like [Source:MGI Symbol;Acc:MGI:1277182]	-2.436	7.81E-03
ENSMUSG00000041044	Lrit1	"leucine-rich repeat, immunoglobulin-like and transmembrane domains 1 [Source:MGI Symbol;Acc:MGI:2385320]"	-2.446	6.68E-22
ENSMUSG00000072949	Acot1	acyl-CoA thioesterase 1 [Source:MGI Symbol;Acc:MGI:1349396]	-2.453	2.04E-09
ENSMUSG00000058613	Cyp2d41-ps	"cytochrome P450, family 2, subfamily d, member 41, pseudogene [Source:MGI Symbol;Acc:MGI:3645691]"	-2.462	2.83E-03
ENSMUSG00000085642	3110053B16Rik	RIKEN cDNA 3110053B16 gene [Source:MGI Symbol;Acc:MGI:1920435]	-2.499	4.51E-02
ENSMUSG00000081084	Gm5638	predicted gene 5638 [Source:MGI Symbol;Acc:MGI:3648121]	-2.503	2.62E-02
ENSMUSG00000053219	Raet1e	retinoic acid early transcript 1E [Source:MGI Symbol;Acc:MGI:2675273]	-2.563	7.64E-04
ENSMUSG00000025002	Cyp2c55	"cytochrome P450, family 2, subfamily c, polypeptide 55 [Source:MGI Symbol;Acc:MGI:1919332]"	-2.668	2.41E-13
ENSMUSG00000096688	Mup17	major urinary protein 17 [Source:MGI Symbol;Acc:MGI:3705217]	-2.695	4.77E-08
ENSMUSG00000073834	Mup11	major urinary protein 11 [Source:MGI Symbol;Acc:MGI:3709617]	-2.702	7.41E-06
ENSMUSG00000110568	Gm7208	predicted gene 7208 [Source:MGI Symbol;Acc:MGI:3648500]	-2.710	2.90E-06
ENSMUSG00000114818	Gm35164	"predicted gene, 35164 [Source:MGI Symbol;Acc:MGI:5594323]"	-2.710	8.69E-04
ENSMUSG00000078687	Mup8	major urinary protein 8 [Source:MGI Symbol;Acc:MGI:3709619]	-2.734	2.91E-09
ENSMUSG00000078675	Mup16	major urinary protein 16 [Source:MGI Symbol;Acc:MGI:3780250]	-2.741	3.57E-07
ENSMUSG00000032561	Acpp	"acid phosphatase, prostate [Source:MGI Symbol;Acc:MGI:1928480]"	-2.758	4.74E-13
ENSMUSG00000078686	Mup9	major urinary protein 9 [Source:MGI Symbol;Acc:MGI:3782918]	-2.759	6.46E-13
ENSMUSG00000115948	Gm49419	"predicted gene, 49419 [Source:MGI Symbol;Acc:MGI:6155052]"	-2.788	3.50E-02
ENSMUSG00000060560	Ces4a	carboxylesterase 4A [Source:MGI Symbol;Acc:MGI:2384581]	-2.821	1.23E-03
ENSMUSG00000118155	Gm50136	"predicted gene, 50136 [Source:MGI Symbol;Acc:MGI:6302882]"	-2.825	1.47E-07
ENSMUSG00000078673	Mup19	major urinary protein 19 [Source:MGI Symbol;Acc:MGI:3705235]	-2.890	4.38E-09
ENSMUSG00000108030	9530062K07Rik	RIKEN cDNA 9530062K07 gene [Source:MGI Symbol;Acc:MGI:1925989]	-2.904	1.10E-02
ENSMUSG00000115423	AL731706.1	novel protein	-2.928	1.63E-10
ENSMUSG00000035112	Wnk4	WNK lysine deficient protein kinase 4 [Source:MGI Symbol;Acc:MGI:1917097]	-2.929	8.06E-04
ENSMUSG00000066071	Cyp4a12a	"cytochrome P450, family 4, subfamily a, polypeptide 12a [Source:MGI Symbol;Acc:MGI:88612]"	-2.980	4.93E-43
ENSMUSG00000078683	Mup1	major urinary protein 1 [Source:MGI Symbol;Acc:MGI:97233]	-2.990	2.94E-10
ENSMUSG00000096674	Mup15	major urinary protein 15 [Source:MGI Symbol;Acc:MGI:3780235]	-3.016	2.29E-07
ENSMUSG00000105790	Gm24105	"predicted gene, 24105 [Source:MGI Symbol;Acc:MGI:5453882]"	-3.023	6.78E-03
ENSMUSG00000086136	Gm12718	predicted gene 12718 [Source:MGI Symbol;Acc:MGI:3651516]	-3.050	1.13E-03
ENSMUSG00000117856	Gm6937	predicted pseudogene 6937 [Source:MGI Symbol;Acc:MGI:3647230]	-3.094	3.28E-03
ENSMUSG00000078597	Cyp4a12b	"cytochrome P450, family 4, subfamily a, polypeptide 12B [Source:MGI Symbol;Acc:MGI:3611747]"	-3.097	1.70E-33
ENSMUSG00000116718	Gm49668	"predicted gene, 49668 [Source:MGI Symbol;Acc:MGI:6215110]"	-3.108	5.71E-04
ENSMUSG00000030483	Cyp2b10	"cytochrome P450, family 2, subfamily b, polypeptide 10 [Source:MGI Symbol;Acc:MGI:88598]"	-3.163	4.12E-06
ENSMUSG00000075551	Cyp3a41a	"cytochrome P450, family 3, subfamily a, polypeptide 41A [Source:MGI Symbol;Acc:MGI:1858451]"	-3.183	3.00E-03
ENSMUSG00000043418	Lrit2	"leucine-rich repeat, immunoglobulin-like and transmembrane domains 2 [Source:MGI Symbol;Acc:MGI:2444885]"	-3.208	1.70E-09
ENSMUSG00000115919	Gm31583	"predicted gene, 31583 [Source:MGI Symbol;Acc:MGI:5590742]"	-3.232	4.47E-136
ENSMUSG00000073435	Nme3	NME/NM23 nucleoside diphosphate kinase 3 [Source:MGI Symbol;Acc:MGI:1930182]	-3.234	2.16E-06
ENSMUSG00000020381	Mrnip	MRN complex interacting protein [Source:MGI Symbol;Acc:MGI:1915317]	-3.242	6.97E-04

ENSMUSG00000075552	Cyp3a41b	"cytochrome P450, family 3, subfamily a, polypeptide 41B [Source:MGI Symbol;Acc:MGI:3714859]"	-3.268	1.67E-02
ENSMUSG00000109612	Gm45253	predicted gene 45253 [Source:MGI Symbol;Acc:MGI:5791089]	-3.304	1.49E-03
ENSMUSG00000079110	Capn3	calpain 3 [Source:MGI Symbol;Acc:MGI:107437]	-3.316	4.62E-03
ENSMUSG00000104520	Gm37336	"predicted gene, 37336 [Source:MGI Symbol;Acc:MGI:5610564]"	-3.410	8.27E-03
ENSMUSG00000041293	Adgrf1	adhesion G protein-coupled receptor F1 [Source:MGI Symbol;Acc:MGI:1924846]	-3.473	2.18E-30
ENSMUSG00000075025	Gm10804	predicted gene 10804 [Source:MGI Symbol;Acc:MGI:3641755]	-3.524	3.30E-03
ENSMUSG00000104063	Pcdhgb7	"protocadherin gamma subfamily B, 7 [Source:MGI Symbol;Acc:MGI:1935199]"	-3.541	3.26E-02
ENSMUSG00000055730	Ces2a	carboxylesterase 2A [Source:MGI Symbol;Acc:MGI:2142491]	-3.601	4.19E-76
ENSMUSG00000110827	Gm32281	"predicted gene, 32281 [Source:MGI Symbol;Acc:MGI:5591440]"	-3.624	1.01E-04
ENSMUSG00000072849	Serpina1e	"serine (or cysteine) peptidase inhibitor, clade A, member 1E [Source:MGI Symbol;Acc:MGI:891967]"	-3.658	1.00E-41
ENSMUSG00000117780	Gm3734	predicted gene 3734 [Source:MGI Symbol;Acc:MGI:3781909]	-3.689	6.23E-33
ENSMUSG00000038656	Cyp3a16	"cytochrome P450, family 3, subfamily a, polypeptide 16 [Source:MGI Symbol;Acc:MGI:106099]"	-3.750	1.42E-02
ENSMUSG00000016356	Col20a1	"collagen, type XX, alpha 1 [Source:MGI Symbol;Acc:MGI:1920618]"	-3.821	4.45E-02
ENSMUSG00000037071	Scd1	stearoyl-Coenzyme A desaturase 1 [Source:MGI Symbol;Acc:MGI:98239]	-3.849	5.14E-44
ENSMUSG00000111329	A830035O19Rik	RIKEN cDNA A830035O19 gene [Source:MGI Symbol;Acc:MGI:3704334]	-3.948	1.32E-02
ENSMUSG00000086389	Gm15998	predicted gene 15998 [Source:MGI Symbol;Acc:MGI:3802094]	-3.981	2.63E-07
ENSMUSG00000090165	Ugt1a10	"UDP glycosyltransferase 1 family, polypeptide A10 [Source:MGI Symbol;Acc:MGI:3580642]"	-3.981	4.93E-03
ENSMUSG00000003053	Cyp2c29	"cytochrome P450, family 2, subfamily c, polypeptide 29 [Source:MGI Symbol;Acc:MGI:103238]"	-3.993	3.83E-56
ENSMUSG00000116437	Gm46545	"predicted gene, 46545 [Source:MGI Symbol;Acc:MGI:5826182]"	-4.031	2.76E-02
ENSMUSG00000022025	Cnmd	chondromodulin [Source:MGI Symbol;Acc:MGI:1341171]	-4.097	1.85E-03
ENSMUSG00000038092	Hsd3b5	"hydroxy-delta-5-steroid dehydrogenase, 3 beta- and steroid delta-isomerase 5 [Source:MGI Symbol;Acc:MGI:104645]"	-4.099	3.99E-07
ENSMUSG00000073842	Mup7	major urinary protein 7 [Source:MGI Symbol;Acc:MGI:3709615]	-4.126	6.52E-21
ENSMUSG00000048424	Ranbp3l	RAN binding protein 3-like [Source:MGI Symbol;Acc:MGI:2444654]	-4.278	3.96E-04
ENSMUSG00000092075	Serpina4-ps1	"serine (or cysteine) peptidase inhibitor, clade A, member 4, pseudogene 1 [Source:MGI Symbol;Acc:MGI:2448363]"	-4.618	4.87E-05
ENSMUSG00000094793	Mup12	major urinary protein 12 [Source:MGI Symbol;Acc:MGI:3780193]	-4.735	4.31E-215
ENSMUSG00000038754	Elovl3	"elongation of very long chain fatty acids (FEN1/Elo2, SUR4/Elo3, yeast)-like 3 [Source:MGI Symbol;Acc:MGI:1195976]"	-4.903	2.86E-142
ENSMUSG00000074607	Tox2	TOX high mobility group box family member 2 [Source:MGI Symbol;Acc:MGI:3611233]	-10.143	2.36E-02
ENSMUSG00000085408	C530005A16Rik	RIKEN cDNA C530005A16 gene [Source:MGI Symbol;Acc:MGI:3612454]	-10.143	3.81E-02
ENSMUSG00000035275	Raver2	"ribonucleoprotein, PTB-binding 2 [Source:MGI Symbol;Acc:MGI:2443623]"	-10.227	1.69E-03
ENSMUSG00000029151	Slc30a3	"solute carrier family 30 (zinc transporter), member 3 [Source:MGI Symbol;Acc:MGI:1345280]"	-10.532	9.93E-03
ENSMUSG00000040860	Crocc	"ciliary rootlet coiled-coil, rootletin [Source:MGI Symbol;Acc:MGI:3529431]"	-10.559	1.22E-05
ENSMUSG00000058914	C1qtnf3	C1q and tumor necrosis factor related protein 3 [Source:MGI Symbol;Acc:MGI:1932136]	-10.564	1.42E-02
ENSMUSG00000112491	Gm19801	"predicted gene, 19801 [Source:MGI Symbol;Acc:MGI:5011986]"	-10.594	1.16E-02
ENSMUSG00000087466	A330041J22Rik	RIKEN cDNA A330041J22 gene [Source:MGI Symbol;Acc:MGI:2442269]	-10.652	1.79E-02
ENSMUSG00000103030	E330011M16Rik	RIKEN cDNA E330011M16 gene [Source:MGI Symbol;Acc:MGI:3704186]	-10.704	1.76E-02
ENSMUSG00000114558	Gm9570	predicted gene 9570 [Source:MGI Symbol;Acc:MGI:3779980]	-10.769	3.09E-02
ENSMUSG00000085708	Gm16063	predicted gene 16063 [Source:MGI Symbol;Acc:MGI:3801955]	-10.794	1.30E-02
ENSMUSG00000042474	Fcmr	Fc fragment of IgM receptor [Source:MGI Symbol;Acc:MGI:1916419]	-10.905	3.38E-02
ENSMUSG00000027296	Itpka	"inositol 1,4,5-trisphosphate 3-kinase A [Source:MGI Symbol;Acc:MGI:1333822]"	-11.028	4.27E-03
ENSMUSG00000039137	Whrn	whirlin [Source:MGI Symbol;Acc:MGI:2682003]	-11.063	6.74E-03
ENSMUSG00000071573	Rnls	"renalase, FAD-dependent amine oxidase [Source:MGI Symbol;Acc:MGI:1915045]"	-11.237	3.32E-02
ENSMUSG00000097092	Gm26725	"predicted gene, 26725 [Source:MGI Symbol;Acc:MGI:5477219]"	-11.269	1.83E-02
ENSMUSG00000105512	Gm43714	predicted gene 43714 [Source:MGI Symbol;Acc:MGI:5663851]	-11.384	2.71E-02
ENSMUSG00000091119	Ccdc152	coiled-coil domain containing 152 [Source:MGI Symbol;Acc:MGI:3641617]	-11.541	1.67E-02
ENSMUSG00000113149	Gm49383	"predicted gene, 49383 [Source:MGI Symbol;Acc:MGI:6121605]"	-11.566	2.99E-02
ENSMUSG00000049721	Gal3st1	galactose-3-O-sulfotransferase 1 [Source:MGI Symbol;Acc:MGI:1858277]	-11.658	6.57E-04
ENSMUSG00000025916	Ppp1r42	"protein phosphatase 1, regulatory subunit 42 [Source:MGI Symbol;Acc:MGI:1921138]"	-11.665	2.07E-02
ENSMUSG00000067049	Unc93a	unc-93 homolog A [Source:MGI Symbol;Acc:MGI:1933250]	-11.756	2.00E-05
ENSMUSG00000072944	Nup62cl	nucleoporin 62 C-terminal like [Source:MGI Symbol;Acc:MGI:2685565]	-11.767	1.35E-04
ENSMUSG00000027713	1810062G17Rik	RIKEN cDNA 1810062G17 gene [Source:MGI Symbol;Acc:MGI:1919532]	-11.768	9.93E-03

ENSMUSG00000095388	Gm10681	predicted gene 10681 [Source:MGI Symbol;Acc:MGI:3711284]	-11.788	1.09E-03
ENSMUSG00000114355	Gm40916	"predicted gene, 40916 [Source:MGI Symbol;Acc:MGI:5623801]"	-11.820	2.77E-02
ENSMUSG00000050097	Ces2b	carboxyesterase 2B [Source:MGI Symbol;Acc:MGI:2448547]	-11.934	4.81E-06
ENSMUSG00000092499	1700092C10Rik	RIKEN cDNA 1700092C10 gene [Source:MGI Symbol;Acc:MGI:1920804]	-11.978	2.95E-02
ENSMUSG00000106040	Cyp3a63-ps	"cytochrome P450, family 3, subfamily a, member 63, pseudogene [Source:MGI Symbol;Acc:MGI:3717142]"	-12.020	1.01E-03
ENSMUSG00000050663	Trhde	TRH-degrading enzyme [Source:MGI Symbol;Acc:MGI:2384311]	-12.122	1.48E-09
ENSMUSG00000095143	Hsd3b4	"hydroxy-delta-5-steroid dehydrogenase, 3 beta- and steroid delta-isomerase 4 [Source:MGI Symbol;Acc:MGI:96236]"	-12.162	1.38E-04
ENSMUSG00000000320	Alox12	arachidonate 12-lipoxygenase [Source:MGI Symbol;Acc:MGI:87998]	-12.230	1.22E-06
ENSMUSG00000032892	Rangrf	RAN guanine nucleotide release factor [Source:MGI Symbol;Acc:MGI:1889073]	-12.248	2.64E-03
ENSMUSG00000093610	Cyp2c53-ps	"cytochrome P450, family 2, subfamily c, polypeptide 53-ps [Source:MGI Symbol;Acc:MGI:3646306]"	-12.257	3.16E-04
ENSMUSG00000097876	Gm16892	"predicted gene, 16892 [Source:MGI Symbol;Acc:MGI:4439816]"	-12.383	1.78E-05
ENSMUSG00000108624	Gm45091	predicted gene 45091 [Source:MGI Symbol;Acc:MGI:5753667]	-12.489	1.72E-02
ENSMUSG00000050914	Ankrd37	ankyrin repeat domain 37 [Source:MGI Symbol;Acc:MGI:3603344]	-12.584	7.62E-03
ENSMUSG00000001095	Slc13a2	"solute carrier family 13 (sodium-dependent dicarboxylate transporter), member 2 [Source:MGI Symbol;Acc:MGI:1276558]"	-12.638	2.43E-07
ENSMUSG00000099055	Zfp-ps	"zinc finger protein, pseudogene [Source:MGI Symbol;Acc:MGI:5521072]"	-12.705	1.46E-02
ENSMUSG00000111409	Gm49380	"predicted gene, 49380 [Source:MGI Symbol;Acc:MGI:6121601]"	-12.837	5.58E-08
ENSMUSG00000086103	Gm11832	predicted gene 11832 [Source:MGI Symbol;Acc:MGI:3650310]	-12.902	2.12E-02
ENSMUSG00000074218	Cox7a1	cytochrome c oxidase subunit 7A1 [Source:MGI Symbol;Acc:MGI:1316714]	-12.989	8.03E-03
ENSMUSG00000081888	Spcs2-ps	"signal peptidase complex subunit 2, pseudogene [Source:MGI Symbol;Acc:MGI:3650451]"	-12.992	4.20E-02
ENSMUSG00000104011	Gm32391	"predicted gene, 32391 [Source:MGI Symbol;Acc:MGI:5591550]"	-13.070	1.76E-03
ENSMUSG00000110649	Gm40466	"predicted gene, 40466 [Source:MGI Symbol;Acc:MGI:5623351]"	-13.477	2.39E-03
ENSMUSG00000087361	0610043K17Rik	RIKEN cDNA 0610043K17 gene [Source:MGI Symbol;Acc:MGI:1915650]	-13.696	1.81E-04
ENSMUSG00000111253	Gm34654	"predicted gene, 34654 [Source:MGI Symbol;Acc:MGI:5593813]"	-14.240	2.81E-05
ENSMUSG00000112793	Gm32872	"predicted gene, 32872 [Source:MGI Symbol;Acc:MGI:5592031]"	-14.649	8.76E-04
ENSMUSG00000092345	Gm20503	predicted gene 20503 [Source:MGI Symbol;Acc:MGI:5141968]	-14.707	4.20E-02
ENSMUSG00000112527	Gm35696	"predicted gene, 35696 [Source:MGI Symbol;Acc:MGI:5594855]"	-15.835	1.62E-09
ENSMUSG00000093752	Gm20716	predicted gene 20716 [Source:MGI Symbol;Acc:MGI:5313163]	-16.061	2.80E-03
ENSMUSG00000083524	Mup-ps21	"major urinary protein, pseudogene 21 [Source:MGI Symbol;Acc:MGI:3652151]"	-18.292	1.39E-16
ENSMUSG00000084309	Mup-ps20	"major urinary protein, pseudogene 20 [Source:MGI Symbol;Acc:MGI:3651976]"	-18.715	1.50E-10
