# Supplementary material for: Transcriptomic Profiling Reveals Inflammatory, Fibrotic, and Apoptotic Signatures in a Methionine–Choline-Deficient Diet-Induced Murine Model of Metabolism-Dysfunction-Associated Steatohepatitis
Source: Int J Mol Sci. 2026 Jul 5;27(13):6033. doi: 10.3390/ijms27136033 (PMC13362325; doi:10.3390/ijms27136033)
Supplement: Supplementary file 1 [file ijms-27-06033-s001.zip › Supplementary Table S2.pdf]

Supplementary Table S2: Gene-cluster annotation of fibrosis-related toxicogenomic signatures and hepatic stellate cell (HSC) activation pathways identified in MCD-induced NASH. Differentially expressed genes are grouped into functional clusters involved in extracellular matrix remodeling, inflammatory signaling, and fibrogenic transcriptional regulation. Gene expression is color-coded, with red indicating up-regulation and blue indicating down-regulation, highlighting stage-specific transcriptional reprogramming during HSC activation under toxic stress conditions.

(A) Fatty acid metabolic process

Gene name	MCD_8	MCD_6	MCD_5	Mean (MCD)	SD (MCD)	Control_1	Control_3	Control_2	Mean (Control)	SD (Control)	Ratio(MCD/Control)	Log2 ratio (MCD/Control)	p value
Apoa4	69.711456	114.850876	151.066635	111.8763223	40.75907586	7.857032	7.972633	3.698847	6.509504	2.434786536	17.18661243	4.10	1.58E-70
Lpl	52.770508	77.053078	99.531425	76.45167033	23.38625896	11.679478	11.064767	7.303865	10.01603667	2.368833841	7.632926364	2.93	4.45E-37
Per2	4.997325	9.521624	6.900687	7.139878667	2.271613931	0.531128	0.857255	0.233351	0.540578	0.312059333	13.20786023	3.72	5.50E-28
Myo5a	3.907474	5.954926	10.058237	6.640212333	3.132121388	0.281527	0.226223	0.212697	0.240149	0.036467003	27.65038511	4.79	1.01E-25
Mfsd2a	12.713952	13.847137	16.067825	14.209638	1.706068948	0.791997	0.552232	0.672024	0.672084333	0.119882511	21.14264133	4.40	3.96E-24
Abhd2	52.686745	82.922012	85.246765	73.61850733	18.16466694	22.607946	21.514482	12.984616	19.03568133	5.268819567	3.867395448	1.95	6.06E-22
Hpgds	3.353066	5.545398	8.31027	5.736244667	2.484106421	0.559293	0.566928	0.201693	0.442638	0.208699408	12.95922326	3.70	3.95E-19
Cd36	67.39222	111.712753	130.990677	103.3652167	32.61061108	16.748146	13.663623	13.823341	14.74503667	1.736580753	7.010170202	2.81	8.94E-18
Tbxas1	4.904555	5.760005	7.04975	5.90477	1.079899568	0.550828	1.135893	0.413869	0.700196667	0.383487827	8.433016438	3.08	2.62E-12
Fads2	135.37822	239.139359	231.93927	202.152283	57.93998565	80.804932	86.142258	43.339821	70.09567033	23.32441569	2.88394821	1.53	7.65E-11
Acnat2	26.010363	28.849596	15.752564	23.53750767	6.889798395	4.247018	5.500902	1.959562	3.902494	1.795632156	6.031401372	2.59	8.02E-10
Anxa1	5.723522	13.890663	13.694172	11.10278567	4.659614831	1.190355	0.960709	1.490654	1.213906	0.265756304	9.14633066	3.19	2.95E-08
Cyp2b9	16.287804	29.833811	5.670237	17.26395067	12.11132626	0.278676	0.113557	0.0001	0.130777667	0.140084119	132.0099303	7.04	4.38E-08
Eif6	18.003166	26.619144	27.040535	23.887615	5.100436022	7.052791	9.383412	3.829021	6.755074667	2.789138058	3.536247366	1.82	6.62E-08
Lipg	3.196416	6.293936	8.066666	5.852329333	2.464969823	1.694607	1.500468	0.505925	1.233666667	0.637674241	4.74384977	2.25	1.18E-06
Prkab2	3.72368	4.091122	5.324923	4.379908333	0.838774669	1.264424	1.077265	0.98227	1.107986333	0.143563819	3.953034619	1.98	7.54E-06
Aacs	12.687875	23.118095	18.292408	18.03279267	5.219954253	1.667948	5.450531	3.697219	3.605232667	1.892968476	5.001838809	2.32	1.17E-05
Cyp4f18	0.996121	0.841431	0.806739	0.881430333	0.100828297	0.0001	0.0001	0.0001	0.0001	0	8814.303333	13.11	1.33E-05
Cyp2a4	4.94988	9.600442	10.344826	8.298382667	2.923675677	1.696293	3.269762	0.941793	1.969282667	1.187750999	4.213911394	2.08	3.25E-05
Brca1	0.768466	1.006926	2.057681	1.277691	0.685933009	0.173116	0.139677	0.134788	0.149193667	0.020861067	8.563976129	3.10	4.54E-05
Ptgs2	0.190687	0.404272	0.161617	0.252192	0.132504758	0.0001	0.0001	0.0001	0.0001	0	2521.92	11.30	1.84E-04
Nr4a3	0.796773	0.486185	0.560618	0.614525333	0.162159572	0.068048	0.096744	0.057729	0.074173667	0.020215969	8.284952881	3.05	1.96E-04
Pla2g4a	2.360547	3.585857	3.328912	3.091772	0.646159952	1.255963	0.944842	0.546342	0.915715667	0.355705986	3.376344986	1.76	2.04E-04
Acot9	2.871638	5.382372	9.212769	5.822259667	3.193369922	0.651563	1.309994	0.935705	0.965754	0.330242414	6.028719184	2.59	2.08E-04
Il1b	3.087222	3.857042	3.995502	3.646588667	0.489347621	0.618111	0.993867	0.429184	0.680387333	0.28744648	5.359577535	2.42	2.37E-04
Elovl7	1.19666	1.32424	2.557905	1.692935	0.751797173	0.323719	0.094531	0.264969	0.227739667	0.119043279	7.433641336	2.89	4.25E-04
Cyp2a22	1.72055	2.695739	2.681057	2.365782	0.558835522	1.009037	0.568987	0.316362	0.631462	0.350538169	3.74651523	1.91	1.20E-03
Ces2h	0.16326	0.364686	0.526261	0.351402333	0.181864712	0.0001	0.0001	0.0001	0.0001	0	3514.023333	11.78	1.24E-03
Cnr1	0.201717	0.133053	0.146317	0.160362333	0.036423066	0.0001	0.0001	0.0001	0.0001	0	1603.623333	10.65	1.41E-03
Aldh1l2	0.187171	0.274243	0.277151	0.246188333	0.051131188	0.042445	0.0001	0.0001	0.014215	0.024447897	17.31891195	4.11	3.54E-03
Alox5ap	5.372902	8.206828	7.755566	7.111765333	1.522709302	1.629578	0.890022	1.89776	1.472453333	0.52191962	4.829874857	2.27	4.79E-03
Hacd4	3.056956	3.891281	5.380105	4.109447333	1.176840147	1.91447	1.41923	1.103479	1.479059667	0.408792481	2.778418901	1.47	6.35E-03
Pam	26.245167	38.805729	47.664001	37.57163233	10.76261392	11.711007	11.785416	12.171385	11.88926933	0.247135817	3.160129633	1.66	7.21E-03
Elovl3	1.980313	5.114073	3.421667	3.505351	1.568555129	140.321976	113.25267	60.952709	104.8424517	40.34747781	0.033434462	-4.90	2.86E-142
Ces2a	4.819698	7.057574	6.976204	6.284492	1.269201074	83.508804	112.214661	33.113518	76.27899433	40.04310573	0.082388239	-3.60	4.19E-76
Cyp2c29	14.335561	28.883343	10.54457	17.921158	9.680910503	350.147858	366.157837	139.509079	285.2715913	126.4875976	0.06282139	-3.99	3.83E-56
Gstp2	71.022636	101.568977	98.84024	90.47728433	16.90337267	314.414886	375.806915	192.140091	294.1206307	93.50009711	0.307619646	-1.70	2.34E-48
Scd1	195.281067	156.986023	93.364708	148.5439327	51.47997402	2791.444092	2405.362793	1226.722046	2141.17631	815.1286055	0.069374919	-3.85	5.14E-44
Cyp4a12a	7.916093	28.101963	24.420113	20.14605633	10.75025615	185.633133	169.496002	121.620911	158.916682	33.29163033	0.126771187	-2.98	4.93E-43
Cyp4a12b	4.27054	7.167961	16.395197	9.277899333	6.331722251	95.348457	74.896782	67.96022	79.40181967	14.23904508	0.11684744	-3.10	1.70E-33
Gstp1	98.116745	167.794693	143.894638	136.6020253	35.40678973	425.866394	614.861938	205.028778	415.25237	205.1226411	0.328961459	-1.60	1.60E-32
Cyp2d9	47.690319	68.996605	46.342739	54.343221	12.72807774	186.289749	226.636841	91.135559	168.0207163	69.57346852	0.323431671	-1.63	3.14E-31
Acsl1	87.225014	158.849152	119.153648	121.7426047	35.88218651	274.578796	293.989746	174.772171	247.7802377	63.9674104	0.491332989	-1.03	2.78E-29
Ces1f	23.203407	53.94231	35.648926	37.59821433	15.46188323	137.486435	158.169083	45.606232	113.7539167	59.91680212	0.330522372	-1.60	1.95E-24
Hacl1	9.994715	15.70973	9.448815	11.71775333	3.467911473	45.153202	62.949551	17.735422	41.94605833	22.77704312	0.279352907	-1.84	8.25E-20
Acacb	2.449609	3.444985	2.643685	2.846093	0.52765528	9.002766	12.55062	3.669805	8.407730333	4.470209095	0.338509073	-1.56	2.74E-19
Ces1e	5.846127	17.13818	9.079555	10.687954	5.8153096	37.232475	43.12064	16.076237	32.14311733	14.22238041	0.332511433	-1.59	2.14E-16
Cyp2c54	13.050272	35.601749	18.887766	22.51326233	11.70471898	70.636276	67.819809	33.090279	57.18212133	20.91161811	0.393711562	-1.34	4.57E-16
Cyp2c55	0.464714	0.96659	0.673826	0.70171	0.252097239	5.469236	6.326211	1.581377	4.458941333	2.52861304	0.157371436	-2.67	2.41E-13
Nudt7	31.445768	86.092949	77.563705	65.03414067	29.39933817	105.864738	209.268051	92.037392	135.7233937	64.0656799	0.479166774	-1.06	2.41E-12
Cyp2c37	26.023289	48.508869	22.931316	32.48782467	13.96049678	99.463501	81.768677	39.245533	73.49257033	30.9503049	0.442055905	-1.18	4.66E-12
Ces1d	29.50577	76.77874	31.83036	46.03829	26.64737096	135.117203	154.098969	44.666683	111.294285	58.47653265	0.41366266	-1.27	2.58E-11
Acot1	0.249951	0.488254	1.712435	0.81688	0.784672638	7.046691	4.812057	1.559489	4.472745667	2.759292587	0.182635021	-2.45	2.04E-09
Bdh2	1.409559	2.724692	3.126332	2.420194333	0.897979123	6.740455	7.930279	3.433563	6.034765667	2.329938148	0.401041974	-1.32	1.17E-08
Alox12	0.0001	0.0001	0.0001	0.0001	0	0.270973	0.488166	0.682345	0.480494667	0.205793264	0.000208119	-12.23	1.22E-06
Cyp2j9	0.858992	1.483821	1.165318	1.169377	0.312434275	4.78116	6.564829	0.905335	4.083774667	2.893480225	0.286347092	-1.80	1.70E-06
Cyp2b10	0.0001	0.485025	0.0001	0.161741667	0.279971579	2.633929	1.179238	0.532643	1.448603333	1.076229053	0.111653524	-3.16	4.12E-06
Cyp4a32	3.387257	6.368027	5.800553	5.185279	1.582772574	16.327248	12.444488	3.400376	10.72403733	6.632945635	0.483519298	-1.05	4.70E-06
Ces2b	0.0001	0.0001	0.0001	0.0001	0	0.147644	0.770903	0.255158	0.391235	0.333167612	0.000255601	-11.93	4.81E-06
Scd4	0.153734	0.124369	0.0001	0.092734333	0.081556211	0.634637	0.630922	0.167049	0.477536	0.268896045	0.194193387	-2.36	2.53E-04
Acsm2	0.0001	0.035906	0.155101	0.063702333	0.08115297	0.309952	0.222137	0.234952	0.255680333	0.047435392	0.249148351	-2.00	3.99E-03

(B) Lipid metabolic pathways

Gene name	MCD_8	MCD_6	MCD_5	Mean (MCD)	SD (MCD)	Control_1	Control_3	Control_2	Mean (Control)	SD (Control)	Ratio(MCD/Control)	Log2 ratio (MCD/Control)	p value
Apoa4	69.711456	114.850876	151.066635	111.8763223	40.75907586	7.857032	7.972633	3.698847	6.509504	2.434786536	17.18661243	4.10	1.58E-70
Cybb	26.786518	33.456963	40.556454	33.59997833	6.886081934	6.298179	5.481755	4.121415	5.300449667	1.099649533	6.339080728	2.66	2.92E-23
Cd36	67.39222	111.712753	130.990677	103.3652167	32.61061108	16.748146	13.663623	13.823341	14.74503667	1.736580753	7.010170202	2.81	8.94E-18
Ikbke	14.680729	19.675493	20.932983	18.429735	3.307053789	2.806845	4.314041	1.55882	2.893235333	1.37964059	6.369939834	2.67	1.61E-13
Jun	13.320441	13.153684	18.944857	15.13966067	3.296451323	3.222338	3.283725	2.228927	2.911663333	0.592063144	5.199660446	2.38	8.72E-13
Vcam1	17.029045	20.617908	21.130627	19.59252667	2.234792806	3.034424	4.064379	3.178267	3.42569	0.557777224	5.719293534	2.52	9.58E-13
Hspa5	157.957611	318.254486	288.018188	254.7434283	85.17146974	81.750336	103.000328	41.1124	75.287688	31.44603747	3.383600096	1.76	9.25E-12
Tlr2	3.357719	5.097874	5.58225	4.679281	1.169850254	0.559075	0.870826	0.554571	0.661490667	0.181303703	7.073842816	2.82	1.21E-10
Ncf1	4.912299	8.731557	8.684598	7.442818	2.191619514	1.2097	1.524921	0.883181	1.205934	0.320886575	6.171828641	2.63	1.84E-10
Apaf1	4.767279	8.200268	9.79176	7.586435667	2.567867779	1.432788	2.123816	1.157867	1.571490333	0.497687774	4.827542051	2.27	4.75E-10
Icam1	8.848032	13.159272	14.102825	12.03670967	2.801485679	3.995374	3.429471	1.951251	3.125365333	1.055447726	3.851296851	1.95	1.93E-09
Ncf2	3.301573	3.915877	2.926745	3.381398333	0.4993742	0.398038	0.351941	0.39643	0.382136333	0.026162283	8.848670065	3.15	3.87E-09

Cd14	3.442406	5.253843	4.148178	4.281475667	0.913045564	0.740475	0.421976	0.309418	0.490623	0.223577374	8.726610181	3.13	6.47E-08
Fos	2.880655	3.22555	4.265087	3.457097333	0.720675805	0.466272	0.513185	0.291599	0.423685333	0.116770317	8.159586989	3.03	7.08E-08
Cxcl1	9.992468	18.591221	15.771691	14.78512667	4.383448434	3.501148	4.993383	1.856167	3.450232667	1.569227624	4.285254965	2.10	1.01E-07
Bcl2	3.181268	6.032191	5.564151	4.92587	1.528886021	1.174392	1.144397	0.908299	1.075696	0.145743754	4.57923986	2.20	1.66E-07
Tlr4	4.066502	4.826442	5.415774	4.769572667	0.676431315	0.890929	1.186947	0.860842	0.979572667	0.1802204	4.869034048	2.28	2.41E-07
Rock2	16.005972	29.101212	31.662199	25.58979433	8.39802962	10.749439	11.071096	5.267833	9.029456	3.261628658	2.834035	1.50	8.90E-07
Cyba	10.98901	17.040386	23.117582	17.04899267	6.064290581	3.700262	4.789875	2.995602	3.828579667	0.903992792	4.453085518	2.15	1.01E-05
Nfkb1	16.833752	21.847376	21.969707	20.216945	2.930569463	8.199962	10.064748	5.015374	7.760028	2.553272585	2.605267017	1.38	1.24E-05
Ticam2	1.628714	1.887846	1.357173	1.624577667	0.265360679	0.15702	0.35287	0.052257	0.187382333	0.152589146	8.669855038	3.12	2.34E-05
Pik3cd	3.242561	3.255134	3.712559	3.403418	0.267797757	0.977231	0.588623	0.510005	0.691953	0.250165646	4.918568169	2.30	2.65E-05
Vav3	3.662432	5.274219	3.782652	4.239767667	0.897875483	0.672254	1.275313	0.649654	0.865740333	0.354880285	4.897274048	2.29	3.38E-05
Pou2f2	1.217442	1.759499	1.548772	1.508571	0.273255445	0.323111	0.385661	0.269578	0.326116667	0.058099839	4.625862932	2.21	3.78E-05
Camk2d	17.473324	23.783125	23.017984	21.424811	3.443406354	9.075263	10.495311	4.373301	7.981291667	3.204267712	2.684378907	1.42	5.26E-05
Vav1	3.346296	4.901774	6.967661	5.071910333	1.816667525	0.663082	1.160354	0.819353	0.880929667	0.254290434	5.757452071	2.53	6.21E-05
Ccl3	2.980034	3.846183	2.176305	3.000840667	0.835133416	0.0001	0.282802	0.0001	0.094334	0.163218076	31.81080699	4.99	1.05E-04
Abcg1	7.312823	10.026082	10.272225	9.20371	1.642174421	2.725366	3.309596	2.220148	2.751703333	0.545201318	3.34473193	1.74	1.60E-04
Nfe2l2	35.233459	57.887398	66.339294	53.15338367	16.08419757	26.368959	22.668737	9.005912	19.34786933	9.145489152	2.747247397	1.46	1.75E-04
Il1b	3.087222	3.857042	3.995502	3.646588667	0.489347621	0.618111	0.993867	0.429184	0.680387333	0.28744648	5.359577535	2.42	2.37E-04
Ccl2	2.694071	6.763049	3.522325	4.326481667	2.150382861	0.707446	0.0001	0.254816	0.320787333	0.358257943	13.48707139	3.75	2.81E-04
Olr1	0.593194	0.708634	0.728011	0.676613	0.07288974	0.106832	0.0001	0.0001	0.035677333	0.061621749	18.96478623	4.25	5.81E-04
Plcb2	0.681293	1.451258	1.952208	1.361586333	0.640185122	0.188285	0.467138	0.152278	0.269233667	0.172333165	5.057266241	2.34	6.40E-04
Irf7	25.143126	28.023054	25.486742	26.21764067	1.572944998	10.230639	8.802448	6.607296	8.546794333	1.825150055	3.06754084	1.62	1.09E-03
Pik3r1	42.224159	53.745712	54.00943	49.99310033	6.729392534	21.906631	28.769907	15.548531	22.075023	6.612296328	2.264690747	1.18	1.38E-03
Traf3	4.075067	6.107934	7.910964	6.031321667	1.91909576	2.202983	2.130257	1.03179	1.788343333	0.656202695	3.372574804	1.75	2.25E-03
Pycard	1.497864	1.693408	1.958261	1.716511	0.231066356	0.4599	0.467403	0.396043	0.441115333	0.039213648	3.891297514	1.96	2.70E-03
Tnf	1.588277	0.836077	0.897892	1.107415333	0.4175838	0.0001	0.0001	0.138356	0.046185333	0.079822139	23.97764081	4.58	3.61E-03
Cxcl2	0.663378	0.872314	0.797186	0.777626	0.105832454	0.031664	0.17666	0.02688	0.078401333	0.085128114	9.918530297	3.31	4.94E-03
Ccl5	16.810623	29.33164	8.743227	18.29516333	10.37417864	2.201626	1.681839	4.694987	2.859484	1.61069801	6.398064593	2.68	6.44E-03
Hspa1b	1.884131	4.765643	4.430022	3.693265333	1.5757175	1.484779	1.50662	0.635649	1.209016	0.496670459	3.054769609	1.61	7.03E-03
Il12b	0.643969	0.466897	0.0001	0.370322	0.332621211	0.0001	0.0001	0.0001	0.0001	0	3703.22	11.85	8.48E-03
Ikbkb	18.713951	29.843973	28.672121	25.74334833	6.115768916	14.333868	16.936432	4.61938	11.96322667	6.491717626	2.151873324	1.11	1.04E-02
Casp1	2.266979	3.169014	3.066336	2.834109667	0.493825463	1.285067	0.59331	0.689028	0.855801667	0.374822663	3.311643079	1.73	1.13E-02
Eif2ak3	6.643215	7.888504	7.386446	7.306055	0.626524707	3.771482	4.099844	1.817307	3.229544333	1.234004098	2.262255676	1.18	1.20E-02
Akt3	5.068993	7.018343	9.252949	7.113428333	2.093598067	2.262787	2.613745	2.470306	2.448946	0.176451315	2.904689745	1.54	1.39E-02
Tnfrsf10b	0.383611	0.562916	0.69835	0.548292333	0.15787827	0.115923	0.101407	0.042099	0.086476333	0.039111243	6.340374438	2.66	1.43E-02
Hsp90aa1	24.020979	71.830948	60.031525	51.96115067	24.90575215	22.615074	27.271923	12.272723	20.71990667	7.677092195	2.507788838	1.33	1.44E-02
Tank	12.98425	22.394497	16.304771	17.22783933	4.772549477	9.642978	9.197989	5.547401	8.129456	2.247167065	2.119187229	1.08	1.48E-02
Ddit3	7.776196	7.18765	8.723456	7.895767333	0.774853531	2.986702	3.453145	1.758164	2.732670333	0.875579359	2.889396221	1.53	1.51E-02
Pparg	4.464601	6.867633	8.173172	6.501802	1.881156282	2.661562	3.396115	1.352939	2.470205333	1.034942062	2.632089694	1.40	1.93E-02
Traf2	3.144925	4.441696	5.519935	4.368852	1.18917947	2.362694	2.302498	1.02316	1.896117333	0.756602121	2.304104247	1.20	1.95E-02
Camk2b	2.30271	2.314077	3.067194	2.561327	0.438130538	1.002788	0.663022	0.831701	0.832503667	0.169884422	3.076655518	1.62	3.06E-02
Nfatc2	1.199017	0.813621	1.393201	1.135279667	0.295000135	0.20991	0.285069	0.24093	0.245303	0.037769845	4.628070862	2.21	3.25E-02
Nlrp3	0.496356	0.883443	1.519317	0.966372	0.516498031	0.295418	0.174132	0.095391	0.188313667	0.100764775	5.131714639	2.36	3.33E-02
Ticam1	4.500633	4.374565	5.434366	4.769854667	0.57892553	1.953384	2.760877	1.332114	2.015458333	0.716401315	2.366635215	1.24	3.57E-02
Tlr6	0.838543	0.939977	1.017893	0.932137667	0.089931624	0.190315	0.31914	0.224845	0.244766667	0.066683017	3.808270462	1.93	3.73E-02
Trp53	4.287155	6.338456	7.904696	6.176769	1.814182388	2.479186	3.645586	1.866174	2.663648667	0.90393397	2.318912805	1.21	4.06E-02
Cyp2j9	0.858992	1.483821	1.165318	1.169377	0.312434275	4.78116	6.564829	0.905335	4.083774667	2.893480225	0.286347092	-1.80	1.70E-06

(C) Regulation of inflammatory response

Gene name	MCD_8	MCD_6	MCD_5	Mean (MCD)	SD (MCD)	Control_1	Control_3	Control_2	Mean (Control)	SD (Control)	Ratio(MCD/Control)	Log2 ratio (MCD/Control)	p value
Lpl	52.770508	77.053078	99.531425	76.45167033	23.38625896	11.679478	11.064767	7.303865	10.01603667	2.368833841	7.632926364	2.93	4.45E-37
Stap1	7.24446	7.892395	8.645535	7.927463333	0.7011955	0.364755	0.235671	0.273573	0.291333	0.066349325	27.21100367	4.77	2.09E-27
Ccr5	8.776878	9.665445	11.077787	9.840036667	1.160347898	1.393449	1.264768	1.003937	1.220718	0.198457052	8.060859811	3.01	1.24E-25
Abcc1	5.439364	9.845737	8.414314	7.899805	2.247792336	1.013951	1.089174	0.517071	0.873398667	0.310872449	9.044901603	3.18	3.83E-22
Trem2	3.313693	5.677338	8.707722	5.899584333	2.703873571	0.0001	0.044638	0.0001	0.014946	0.025714026	394.7266381	8.62	1.25E-16
Ctss	93.409073	144.904114	157.826706	132.046631	34.07923139	19.312706	14.47446	16.792931	16.86003233	2.419820868	7.831932252	2.97	1.56E-16
Ccr2	7.090495	11.523571	13.944882	10.85298267	3.476049744	0.817567	0.777805	1.109865	0.901745667	0.181329801	12.03552517	3.59	2.09E-16
Tnfaip3	3.590337	5.953007	5.389837	4.977727	1.234069841	0.623732	0.77908	0.342952	0.581921333	0.221049784	8.553951737	3.10	2.33E-15
Serpine1	3.835907	5.917427	5.497101	5.083478333	1.100678954	0.379806	0.0001	0.106057	0.161987667	0.195934533	31.38188504	4.97	1.51E-14
Cx3cr1	4.311779	3.838964	7.587977	5.24624	2.041736427	0.194496	0.292297	0.517835	0.334876	0.165821457	15.66621675	3.97	7.39E-11
Tlr2	3.357719	5.097874	5.58225	4.679281	1.169850254	0.559075	0.870826	0.554571	0.661490667	0.181303703	7.073842816	2.82	1.21E-10
Ncf1	4.912299	8.731557	8.684598	7.442818	2.191619514	1.2097	1.524921	0.883181	1.205934	0.320886575	6.171828641	2.63	1.84E-10
Tnfrsf1b	11.714591	13.829306	17.965767	14.50322133	3.179610315	4.717139	4.828391	2.668017	4.071182333	1.216449328	3.562410166	1.83	3.65E-10
Cd44	23.437643	26.690445	32.843838	27.65730867	4.77705402	3.773986	3.857448	5.991861	4.541098333	1.257090177	6.090444786	2.61	7.17E-10
Cd200r1	2.351574	3.288312	2.704568	2.781484667	0.473082076	0.243229	0.306553	0.156964	0.235582	0.075087114	11.80686414	3.56	1.23E-09
Zbp1	3.805883	9.559266	10.588037	7.984395333	3.655074067	3.441186	2.665846	1.264411	2.457147667	1.103292163	3.249456857	1.70	1.80E-09
Tnfrsf11a	5.17039	4.467984	9.43121	6.356528	2.685813768	0.796195	1.047383	0.730147	0.857908333	0.167380021	7.409332388	2.89	1.83E-09
Abr	5.899267	7.549968	8.204133	7.217789333	1.187795782	1.271677	1.006733	0.999307	1.092572333	0.155153626	6.606234766	2.72	3.79E-09
Lacc1	8.225266	14.10885	14.67161	12.335242	3.570448395	4.142697	3.637555	1.97619	3.252147333	1.133508909	3.792953005	1.92	1.32E-08
Adamts12	2.071282	2.388525	3.10199	2.520599	0.527894309	0.279933	0.281169	0.311597	0.290899667	0.017935067	8.664839767	3.12	2.14E-08
Anxa1	5.723522	13.890663	13.694172	11.10278567	4.659614831	1.190355	0.960709	1.490654	1.213906	0.265756304	9.14633066	3.19	2.95E-08
Tlr4	4.066502	4.826442	5.415774	4.769572667	0.676431315	0.890929	1.186947	0.860842	0.979572667	0.1802204	4.869034048	2.28	2.41E-07
Pik3cg	1.720092	3.068679	2.840368	2.543046333	0.7217838	0.462724	0.711665	0.392373	0.522254	0.167763855	4.869366885	2.28	4.28E-07
Acod1	0.612813	1.348842	0.544758	0.835471	0.445892597	0.0001	0.0001	0.0001	0.0001	0	8354.71	13.03	1.13E-06
S100a8	1.474508	7.543886	5.025886	4.681426667	3.049315743	0.0001	0.0001	0.0001	0.0001	0	46814.26667	15.51	1.19E-06
Tnc	1.456822	2.667684	2.153531	2.092679	0.607720265	0.052704	0.145345	0.263378	0.153809	0.105591728	13.60569928	3.77	1.81E-06
S100a9	3.013048	8.546268	1.499342	4.352886	3.709604549	0.0001	0.0001	0.0001	0.0001	0	43528.86	15.41	2.80E-06
Nfkb1	16.833752	21.847376	21.969707	20.216945	2.930569463	8.199962	10.064748	5.015374	7.760028	2.553272585	2.605267017	1.38	1.24E-05
Fcgr3	10.748247	12.872694	16.957624	13.52618833	3.155848826	2.570576	2.678614	2.958003	2.735731	0.199929197	4.9442684	2.31	2.55E-05
Nfkbiz	6.406047	9.517214	8.678941	8.200734	1.609767523	3.602537	2.566353	1.130238	2.433042667	1.241529025	3.370567279	1.75	2.84E-05
Nlrp1b	0.929004	1.678178	1.52748	1.378220667	0.396262769	0.108929	0.358854	0.145562	0.204448333	0.134967872	6.74116851	2.75	3.74E-05

Hgf	17.333931	21.255636	22.728661	20.43940933	2.788448928	6.723717	6.812789	5.035504	6.19067	1.001393939	3.301647372	1.72	5.30E-05
Fcer1g	54.785313	52.94714	81.129715	62.954056	15.76739215	13.963125	12.259938	15.892587	14.03855	1.817498662	4.484370252	2.16	9.21E-05
Ccl3	2.980034	3.846183	2.176305	3.000840667	0.835133416	0.0001	0.282802	0.0001	0.094334	0.163218076	31.81080699	4.99	1.05E-04
Akna	2.490627	3.185332	2.344496	2.673485	0.449253916	0.654744	0.666751	0.564265	0.628586667	0.056026776	4.253168484	2.09	1.09E-04
Slamf8	2.418212	3.724198	2.691711	2.944707	0.688770709	0.460722	0.31097	0.421551	0.397747667	0.077661859	7.403455122	2.89	1.09E-04
Il17ra	3.298273	6.238379	5.028948	4.8552	1.477733771	1.929725	1.530479	0.767061	1.409088333	0.590761089	3.445632105	1.78	1.22E-04
Lrrk2	2.541719	4.631986	3.799882	3.657862333	1.052345581	0.928211	1.155372	0.772116	0.951899667	0.192723001	3.842697357	1.94	1.25E-04
Ptgs2	0.190687	0.404272	0.161617	0.252192	0.132504758	0.0001	0.0001	0.0001	0.0001	0	2521.92	11.30	1.84E-04
Pla2g4a	2.360547	3.585857	3.328912	3.091772	0.646159952	1.255963	0.944842	0.546342	0.915715667	0.355705986	3.376344986	1.76	2.04E-04
Il1b	3.087222	3.857042	3.995502	3.646588667	0.489347621	0.618111	0.993867	0.429184	0.680387333	0.28744648	5.359577535	2.42	2.37E-04
Fut7	0.221797	0.337089	1.031602	0.530162667	0.438068619	0.0001	0.0001	0.0001	0.0001	0	5301.626667	12.37	3.76E-04
Tlr9	0.682654	0.922074	1.475561	1.026763	0.406688109	0.179626	0.072321	0.122808	0.124918333	0.053683618	8.219474056	3.04	6.36E-04
Plcg2	1.479316	2.74024	2.885962	2.368506	0.773500399	0.587638	0.868174	0.498662	0.651491333	0.192854131	3.63551421	1.86	7.46E-04
Casp4	2.398894	5.968969	4.457874	4.275245667	1.79203061	0.746511	1.25659	0.790885	0.931328667	0.282557011	4.590480053	2.20	8.15E-04
Adam8	1.851774	4.099059	5.853451	3.934761333	2.005891322	0.0001	0.0001	0.084213	0.028137667	0.048562663	139.8396456	7.13	8.34E-04
Ptger4	1.464841	1.755331	2.431157	1.883776333	0.495797646	0.176778	0.0001	0.275844	0.150907333	0.139680553	12.48300061	3.64	1.00E-03
Clcf1	0.935722	1.628085	2.390022	1.651276333	0.727427317	0.328131	0.368587	0.245587	0.314101667	0.062688648	5.257139673	2.39	1.01E-03
Lgals1	12.441858	21.176861	22.430651	18.68312333	5.441327233	2.934938	4.508886	4.959064	4.134296	1.06278397	4.519057981	2.18	1.04E-03
Fancd2	0.202849	0.481663	0.289621	0.324711	0.142680737	0.0001	0.0001	0.064054	0.021418	0.036923859	15.16065926	3.92	1.18E-03
Cnr1	0.201717	0.133053	0.146317	0.160362333	0.036423066	0.0001	0.0001	0.0001	0.0001	0	1603.623333	10.65	1.41E-03
Aim2	2.687448	3.868723	1.997005	2.851058667	0.946524382	0.43896	0.731736	0.342131	0.504275667	0.202848752	5.653770061	2.50	2.28E-03
Stk39	2.404419	4.108631	5.784153	4.099067667	1.689887295	0.888197	1.624945	0.72024	1.077794	0.481230767	3.803201416	1.93	2.63E-03
Pycard	1.497864	1.693408	1.958261	1.716511	0.231066356	0.4599	0.467403	0.396043	0.441115333	0.039213648	3.891297514	1.96	2.70E-03
Nupr1	3.111801	0.838071	4.016768	2.655546667	1.637728508	0.190545	0.122202	0.411531	0.241426	0.151226567	10.99942287	3.46	2.74E-03
Pbk	0.469142	0.791409	0.945378	0.735309667	0.24302373	0.0001	0.113112	0.0001	0.037770667	0.065247509	19.46774393	4.28	3.54E-03
Tnf	1.588277	0.836077	0.897892	1.107415333	0.4175838	0.0001	0.0001	0.138356	0.046185333	0.079822139	23.97764081	4.58	3.61E-03
Metrnl	2.84458	2.930379	3.864217	3.213058667	0.565549068	0.925241	0.664291	0.66551	0.751680667	0.150308894	4.274499544	2.10	3.77E-03
Tnip1	4.082422	4.592513	7.876553	5.517162667	2.059147947	2.066252	2.333972	1.036901	1.812375	0.684790802	3.044161758	1.61	4.04E-03
Fcgr1	2.186137	2.900917	2.857946	2.648333333	0.40084999	0.485769	1.079803	0.58662	0.717397333	0.317877542	3.691585137	1.88	4.49E-03
Ttbk1	0.41008	0.355782	0.429136	0.398332667	0.038061821	0.058936	0.138599	0.058682	0.085405667	0.046066953	4.664007462	2.22	4.61E-03
Alox5ap	5.372902	8.206828	7.755566	7.111765333	1.522709302	1.629578	0.890022	1.89776	1.472453333	0.52191962	4.829874857	2.27	4.79E-03
Ccr7	1.154409	1.499007	0.505854	1.05309	0.50426915	0.066697	0.0001	0.140243	0.069013333	0.070100208	15.25922527	3.93	6.03E-03
Ccl5	16.810623	29.33164	8.743227	18.29516333	10.37417864	2.201626	1.681839	4.694987	2.859484	1.61069801	6.398064593	2.68	6.44E-03
Gstp1	98.116745	167.794693	143.894638	136.6020253	35.40678973	425.866394	614.861938	205.028778	415.25237	205.1226411	0.328961459	-1.60	1.60E-32
Sod1	93.598885	156.386749	153.048264	134.3446327	35.32631222	482.522766	538.212646	252.801468	424.5122933	151.290437	0.31646818	-1.66	6.31E-30
Enpp3	6.12862	12.804184	6.603952	8.512252	3.724512766	22.003433	25.318233	9.624135	18.98193367	8.271836052	0.448439666	-1.16	7.01E-19
**(D) Antioxidant activity**													
**Gene name**	**MCD_8**	**MCD_6**	**MCD_5**	**Mean (MCD)**	**SD (MCD)**	**Control_1**	**Control_3**	**Control_2**	**Mean (Control)**	**SD (Control)**	**Ratio(MCD/Control)**	**Log2 ratio (MCD/Control)**	**p value**
Apoa4	69.711456	114.850876	151.066635	111.8763223	40.75907586	7.857032	7.972633	3.698847	6.509504	2.434786536	17.18661243	4.10	1.58E-70
Srxn1	35.777042	36.469738	46.025841	39.424207	5.727664056	8.722608	10.806087	4.400829	7.976508	3.267159534	4.942539643	2.31	6.72E-18
Gsta1	9.722733	19.227453	25.203932	18.05137267	7.80732056	4.038228	1.704451	0.754615	2.165764667	1.68971486	8.334872641	3.06	5.11E-08
S100a8	1.474508	7.543886	5.025886	4.681426667	3.049315743	0.0001	0.0001	0.0001	0.0001	0	46814.26667	15.51	1.19E-06
S100a9	3.013048	8.546268	1.499342	4.352886	3.709604549	0.0001	0.0001	0.0001	0.0001	0	43528.86	15.41	2.80E-06
Ptgs2	0.190687	0.404272	0.161617	0.252192	0.132504758	0.0001	0.0001	0.0001	0.0001	0	2521.92	11.30	1.84E-04
Hp	538.16449	788.194824	571.991516	632.78361	135.6486307	267.724609	368.478241	141.969864	259.3909047	113.483916	2.439498065	1.29	4.47E-04
Nqo1	4.965587	5.060993	7.700558	5.909046	1.55222808	1.801479	1.93731	1.083477	1.607422	0.458804193	3.676101235	1.88	4.74E-04
Txnrd1	53.90041	70.224388	76.909615	67.011471	11.83630033	32.655994	41.708221	16.346544	30.23691967	12.85272755	2.216213548	1.15	2.46E-03
Alox5ap	5.372902	8.206828	7.755566	7.111765333	1.522709302	1.629578	0.890022	1.89776	1.472453333	0.52191962	4.829874857	2.27	4.79E-03
Cygb	9.478873	8.900109	14.833219	11.07073367	3.271232773	2.845244	3.088143	4.026424	3.319937	0.623773109	3.334621611	1.74	2.18E-02
Ubiad1	3.501953	5.192955	5.256497	4.650468333	0.995150742	2.522036	2.588794	1.094279	2.068369667	0.844247374	2.24837388	1.17	4.27E-02
Gstp2	71.022636	101.568977	98.84024	90.47728433	16.90337267	314.414886	375.806915	192.140091	294.1206307	93.50009711	0.307619646	-1.70	2.34E-48
Gstp1	98.116745	167.794693	143.894638	136.6020253	35.40678973	425.866394	614.861938	205.028778	415.25237	205.1226411	0.328961459	-1.60	1.60E-32
Sod1	93.598885	156.386749	153.048264	134.3446327	35.32631222	482.522766	538.212646	252.801468	424.5122933	151.290437	0.31646818	-1.66	6.31E-30
**(E) MAPK signaling pathway**													
**Gene name**	**MCD_8**	**MCD_6**	**MCD_5**	**Mean (MCD)**	**SD (MCD)**	**Control_1**	**Control_3**	**Control_2**	**Mean (Control)**	**SD (Control)**	**Ratio(MCD/Control)**	**Log2 ratio (MCD/Control)**	**p value**
Jun	13.320441	13.153684	18.944857	15.13966067	3.296451323	3.222338	3.283725	2.228927	2.911663333	0.592063144	5.199660446	2.38	8.72E-13
Tgfbr1	28.123123	44.017212	55.69162	42.61065167	13.83796646	15.568585	14.762262	6.942194	12.424347	4.764770753	3.42960895	1.78	1.06E-09
Ntrk2	2.926632	3.18214	4.622149	3.576973667	0.914119627	0.334422	0.390934	0.483456	0.402937333	0.075238576	8.877245593	3.15	6.27E-08
Cd14	3.442406	5.253843	4.148178	4.281475667	0.913045564	0.740475	0.421976	0.309418	0.490623	0.223577374	8.726610181	3.13	6.47E-08
Fos	2.880655	3.22555	4.265087	3.457097333	0.720675805	0.466272	0.513185	0.291599	0.423685333	0.116770317	8.159586989	3.03	7.08E-08
Pak1	3.688318	3.85305	5.073203	4.204857	0.756506933	1.02502	0.98044	0.623948	0.876469333	0.21982291	4.797494721	2.26	2.26E-06
Flna	17.660484	28.352297	28.100143	24.704308	6.101433259	5.427261	6.340096	5.951792	5.906383	0.458108521	4.182645792	2.06	4.00E-06
Relb	1.915902	2.263598	2.517196	2.232232	0.301871642	0.504674	0.777929	0.196373	0.492992	0.290953943	4.527927431	2.18	6.64E-06
Nfkb1	16.833752	21.847376	21.969707	20.216945	2.930569463	8.199962	10.064748	5.015374	7.760028	2.553272585	2.605267017	1.38	1.24E-05
Epha2	5.487962	10.03554	10.484872	8.669458	2.764400913	2.313144	1.410647	1.377757	1.700516	0.530806215	5.098133743	2.35	1.98E-05
Dusp8	1.133642	0.895951	1.092428	1.040673667	0.127016283	0.203579	0.083561	0.101037	0.129392333	0.064839022	8.042776878	3.01	2.60E-05
Pdgfa	6.958641	10.216499	13.344239	10.17312633	3.193019941	1.372568	2.174555	1.985016	1.844046333	0.419165984	5.516741174	2.46	3.60E-05
Ptpn7	0.570752	0.933058	1.591969	1.031926333	0.517737627	0.074559	0.055588	0.0758	0.068649	0.011328165	15.03192083	3.91	4.31E-05
Map3k8	4.644087	8.102399	8.356988	7.034491333	2.074060883	1.99585	2.687032	0.903784	1.862222	0.899102713	3.77747193	1.92	4.82E-05
Hgf	17.333931	21.255636	22.728661	20.43940933	2.788448928	6.723717	6.812789	5.035504	6.19067	1.001393939	3.301647372	1.72	5.30E-05
Flnb	12.33709	18.498989	21.05652	17.297533	4.482157658	7.690653	9.952458	3.669225	7.104112	3.182416848	2.434862091	1.28	1.46E-04
Csf1	7.173495	9.56284	10.742694	9.159676333	1.818433689	2.645988	2.301589	1.963312	2.303629667	0.341342575	3.976193077	1.99	1.55E-04
Pla2g4a	2.360547	3.585857	3.328912	3.091772	0.646159952	1.255963	0.944842	0.546342	0.915715667	0.355705986	3.376344986	1.76	2.04E-04
Dusp5	1.627152	1.915344	2.432193	1.991563	0.407896745	0.279958	0.400829	0.312297	0.331028	0.062574655	6.016297715	2.59	2.05E-04
Il1b	3.087222	3.857042	3.995502	3.646588667	0.489347621	0.618111	0.993867	0.429184	0.680387333	0.28744648	5.359577535	2.42	2.37E-04
Mef2c	5.867791	9.521666	10.915373	8.768276667	2.606763823	2.878496	2.482372	2.333901	2.564923	0.281526072	3.418534072	1.77	2.55E-04
Map3k14	1.576148	1.217942	1.93398	1.576023333	0.358019016	0.236028	0.452531	0.223785	0.304114667	0.128678004	5.182332541	2.37	2.87E-04
Pdgfra	7.470135	6.188165	12.186909	8.615069667	3.159017084	1.979849	2.904773	2.450783	2.445135	0.462487866	3.523351335	1.82	4.51E-04
Nfkb2	2.49395	3.813434	3.99385	3.433744667	0.81886995	1.120676	1.217147	0.777202	1.038341667	0.231240368	3.306950667	1.73	5.34E-04
Dusp6	30.884878	36.665268	47.049454	38.19986667	8.190825606	20.232609	9.747673	7.503527	12.494603	6.794600831	3.057309357	1.61	6.60E-04

Fgf21	3.229264	1.577712	1.585997	2.130991	0.951141339	0.0001	0.308216	0.0001	0.102805333	0.177890856	20.72840903	4.37	8.55E-04
Prkcb	5.808091	8.861041	8.357742	7.675624667	1.63679228	1.566424	1.862392	2.667281	2.032032333	0.569697214	3.777314239	1.92	9.03E-04
Cacna2d1	1.923955	3.591355	3.575439	3.030249667	0.958112335	0.662719	0.635259	0.501961	0.599979667	0.085989867	5.05058727	2.34	2.04E-03
Ereg	0.628018	0.874722	1.621275	1.041338333	0.517165949	0.174981	0.06827	0.171263	0.138171333	0.060564868	7.53657295	2.91	2.15E-03
Pdgfrb	4.784432	7.691014	8.69035	7.055265333	2.029083816	1.581906	2.241593	2.197558	2.007019	0.368816441	3.515295736	1.81	2.37E-03
Cacna1e	0.416421	1.083896	0.426244	0.642187	0.382562744	0.145993	0.163472	0.045047	0.118170667	0.06392716	5.434402785	2.44	3.58E-03
Tnf	1.588277	0.836077	0.897892	1.107415333	0.4175838	0.0001	0.0001	0.138356	0.046185333	0.079822139	23.97764081	4.58	3.61E-03
Map4k1	0.0001	0.679077	1.15858	0.612585667	0.582095182	0.0001	0.0001	0.0001	0.0001	0	6125.856667	12.58	3.66E-03
Nr4a1	3.054695	7.230901	1.372242	3.885946	3.016488851	0.190898	0.841147	0.756272	0.596105667	0.353476847	6.518887871	2.70	4.77E-03
Rapgef2	12.44952	17.993814	19.51161	16.651648	3.717436747	8.94883	9.214381	4.03062	7.397943667	2.919208941	2.250848175	1.17	5.46E-03
Jund	34.213085	29.01672	41.868526	35.032777	6.464994246	17.142405	13.116905	10.188709	13.482673	3.49124787	2.59835546	1.38	6.38E-03
Stmn1	7.182033	9.093618	11.714077	9.329909333	2.275243042	0.808983	2.264077	2.50809	1.860383333	0.918677082	5.01504672	2.33	6.90E-03
Ngf	5.876041	8.775425	9.130369	7.927278333	1.785266719	1.183093	1.882611	1.487185	1.517629667	0.35075136	5.223460313	2.39	6.98E-03
Hspa1b	1.884131	4.765643	4.430022	3.693265333	1.5757175	1.484779	1.50662	0.635649	1.209016	0.496670459	3.054769609	1.61	7.03E-03
Map4k4	14.22518	16.991571	17.204727	16.14049267	1.662129907	6.059124	6.633815	3.230377	5.307772	1.821879504	3.040916729	1.60	7.21E-03
Csf1r	19.79039	20.638063	21.775183	20.73454533	0.995907849	8.140139	10.832973	6.622302	8.531804667	2.132484247	2.43026489	1.28	8.00E-03
Gadd45b	2.081961	2.892338	2.61706	2.530453	0.41207196	0.932205	0.652185	0.345797	0.643395667	0.293302787	3.932965562	1.98	8.61E-03
Ikbkb	18.713951	29.843973	28.672121	25.74334833	6.115768916	14.333868	16.936432	4.61938	11.96322667	6.491717626	2.151873324	1.11	1.04E-02
Pdgfb	1.5534	1.516991	2.611618	1.894003	0.621739391	0.307016	0.443586	0.493877	0.414826333	0.096693315	4.565773308	2.19	1.04E-02
Mapkapk3	3.918055	4.0662	5.590255	4.524836667	0.925647829	1.394168	2.810476	1.321965	1.842203	0.839325783	2.456209585	1.30	1.15E-02
Max	22.989607	27.142931	32.754063	27.628867	4.900331682	15.747347	13.43331	7.906469	12.36237533	4.028649618	2.234915723	1.16	1.25E-02
Akt3	5.068993	7.018343	9.252949	7.113428333	2.093598067	2.262787	2.613745	2.470306	2.448946	0.176451315	2.904689745	1.54	1.39E-02
Areg	0.391533	0.0001	1.480472	0.624035	0.767084219	0.0001	0.0001	0.0001	0.0001	0	6240.35	12.61	1.41E-02
Ddit3	7.776196	7.18765	8.723456	7.895767333	0.774853531	2.986702	3.453145	1.758164	2.732670333	0.875579359	2.889396221	1.53	1.51E-02
Traf2	3.144925	4.441696	5.519935	4.368852	1.18917947	2.362694	2.302498	1.02316	1.896117333	0.756602121	2.304104247	1.20	1.95E-02
Tgfb1	8.612462	8.625794	8.650573	8.629609667	0.019339896	4.25825	3.551817	2.511274	3.440447	0.878796759	2.50828153	1.33	2.02E-02
Cacnb3	0.560786	0.943916	0.878407	0.794369667	0.204924021	0.0001	0.0001	0.161083	0.053761	0.092943578	14.77594663	3.89	2.31E-02
Mknk2	20.276863	24.095249	35.194977	26.522363	7.749560833	14.885331	16.292988	7.236211	12.80484333	4.873666392	2.071275869	1.05	2.62E-02
Mknk1	6.279087	7.575273	8.708579	7.520979667	1.215655654	2.905203	4.924805	1.662168	3.164058667	1.64664955	2.377003861	1.25	3.01E-02
Mapk8ip3	9.110793	12.140924	14.163512	11.80507633	2.543046906	3.348737	8.99677	1.89292	4.746142333	3.75242954	2.487299264	1.31	3.04E-02
Cacnb2	1.12598	1.276731	1.005537	1.136082667	0.135878969	0.182312	0.698983	0.246305	0.375866667	0.281650315	3.022568287	1.60	3.32E-02
Pak2	17.107498	22.979727	25.33938	21.80886833	4.239003754	12.236254	11.637875	7.48056	10.451563	2.590300847	2.086660946	1.06	3.98E-02
Trp53	4.287155	6.338456	7.904696	6.176769	1.814182388	2.479186	3.645586	1.866174	2.663648667	0.90393397	2.318912805	1.21	4.06E-02
Efna2	0.0001	0.412928	0.331581	0.248203	0.218679349	0.0001	0.0001	0.0001	0.0001	0	2482.03	11.28	4.40E-02
Fgf2	1.344556	1.683571	2.208333	1.745486667	0.435204364	0.569544	0.747205	0.711103	0.675950667	0.093902197	2.582269317	1.37	4.69E-02
**(F) Kinase regulator activity**													
**Gene name**	**MCD_8**	**MCD_6**	**MCD_5**	**Mean (MCD)**	**SD (MCD)**	**Control_1**	**Control_3**	**Control_2**	**Mean (Control)**	**SD (Control)**	**Ratio(MCD/Control)**	**Log2 ratio (MCD/Control)**	**p value**
Lilrb4a	29.170012	45.971813	40.350559	38.49746133	8.552812837	2.269317	2.19014	1.226464	1.895307	0.580586314	20.31199238	4.34	1.47E-72
Ccnd1	108.486961	143.475174	151.218903	134.3936793	22.767518	13.224729	10.919368	10.108171	11.41742267	1.616872685	11.77092968	3.56	2.09E-40
Stap1	7.24446	7.892395	8.645535	7.927463333	0.7011955	0.364755	0.235671	0.273573	0.291333	0.066349325	27.21100367	4.77	2.09E-27
Nrg1	5.027548	8.024668	16.538511	9.863575667	5.971746256	0.510232	0.938075	0.564129	0.670812	0.233020074	14.70393444	3.88	3.61E-17
Nckap1l	9.101505	12.216705	13.537497	11.618569	2.277681115	1.659892	2.38296	1.531306	1.858052667	0.459107146	6.253089166	2.64	4.46E-17
Trem2	3.313693	5.677338	8.707722	5.899584333	2.703873571	0.0001	0.044638	0.0001	0.014946	0.025714026	394.7266381	8.62	1.25E-16
Iqgap1	21.027636	27.062792	30.140291	26.07690633	4.635633527	4.668788	5.113707	3.589745	4.457413333	0.783660948	5.850232945	2.55	9.22E-16
Ncapg2	3.682717	6.460612	6.902937	5.682088667	1.745573873	0.921281	0.999799	0.634344	0.851808	0.192377786	6.670621392	2.74	5.27E-13
Pkib	2.33251	2.759153	3.857165	2.982942667	0.786577764	0.615875	0.174554	0.132146	0.307525	0.267879453	9.699837954	3.28	2.05E-11
Pik3r5	1.275751	1.95329	3.389161	2.206067333	1.079142133	0.14845	0.152942	0.063727	0.121706333	0.050261783	18.12615065	4.18	6.35E-11
Rhoh	2.333792	2.764383	3.253043	2.783739333	0.459931083	0.500933	0.528859	0.206687	0.412159667	0.178491535	6.754031407	2.76	2.08E-10
Btc	2.106282	4.628687	5.411007	4.048658667	1.727028223	0.689997	1.334736	0.593754	0.872829	0.40290723	4.638547375	2.21	3.47E-07
Cdkn1a	3.524973	4.855404	6.935457	5.105278	1.718917695	0.319488	0.52666	0.667747	0.504631667	0.175171397	10.11684034	3.34	7.21E-06
Mob3a	2.992995	7.406395	6.949201	5.782863667	2.426887301	1.14233	1.11911	0.739913	1.000451	0.225931027	5.780256771	2.53	2.02E-05
Strada	3.107275	4.792727	4.994759	4.298253667	1.036352677	1.12967	1.674491	0.937498	1.247219667	0.382299769	3.446268353	1.79	7.08E-05
Incenp	2.147689	2.687885	2.101286	2.312286667	0.32610411	0.2981	0.61364	0.195035	0.368925	0.218104727	6.267633439	2.65	6.80E-04
Parp16	6.804553	14.047231	13.24236	11.36471467	3.969667511	3.372605	5.456484	2.114109	3.647732667	1.688087404	3.1155558	1.64	7.81E-04
Pik3r1	42.224159	53.745712	54.00943	49.99310033	6.729392534	21.906631	28.769907	15.548531	22.075023	6.612296328	2.264690747	1.18	1.38E-03
Wnk1	57.629589	86.317635	92.139786	78.69567	18.47455513	42.045624	42.397697	23.62455	36.02262367	10.73848974	2.184617943	1.13	1.80E-03
Ccnl1	23.108099	65.006653	56.914776	48.343176	22.22558102	28.774118	24.917215	13.858088	22.51647367	7.74239349	2.147013636	1.10	1.79E-03
Malt1	10.545985	18.800154	14.362109	14.569416	4.130987608	6.373423	8.047117	2.379812	5.600117333	2.911715599	2.601626918	1.38	1.94E-03
Ereg	0.628018	0.874722	1.621275	1.041338333	0.517165949	0.174981	0.06827	0.171263	0.138171333	0.060564868	7.53657295	2.91	2.15E-03
Ccna2	1.339687	3.581311	4.508718	3.143238667	1.629300432	0.617612	0.463483	0.608432	0.563175667	0.086458308	5.581275706	2.48	2.32E-03
P2rx7	3.068095	3.238718	3.280267	3.195693333	0.112439232	0.869437	0.935586	0.753537	0.852853333	0.092150546	3.747060847	1.91	3.12E-03
Ccnb1	1.356697	1.467595	1.499939	1.441410333	0.075125197	0.228002	0.205621	0.263491	0.232371333	0.029181373	6.203047134	2.63	3.98E-03
Ccne2	0.392252	2.429292	1.420759	1.414101	1.018536321	0.178941	0.208185	0.200928	0.196018	0.015227737	7.214138497	2.85	4.75E-03
Ccl5	16.810623	29.33164	8.743227	18.29516333	10.37417864	2.201626	1.681839	4.694987	2.859484	1.61069801	6.398064593	2.68	6.44E-03
Tpx2	0.68717	0.893726	1.665002	1.081966	0.515378093	0.156862	0.160174	0.20587	0.174302	0.027388799	6.207421602	2.63	6.95E-03
Ngf	5.876041	8.775425	9.130369	7.927278333	1.785266719	1.183093	1.882611	1.487185	1.517629667	0.35075136	5.223460313	2.39	6.98E-03
Agap2	0.592808	0.835679	1.065768	0.831418333	0.236508785	0.103842	0.311152	0.158079	0.191024333	0.107510024	4.35242107	2.12	7.83E-03
Nck1	8.080517	11.68744	10.598518	10.12215833	1.850043859	4.908957	4.956299	2.63919	4.168148667	1.324328611	2.428454247	1.28	9.44E-03
Areg	0.391533	0.0001	1.480472	0.624035	0.767084219	0.0001	0.0001	0.0001	0.0001	0	6240.35	12.61	1.41E-02
Dbf4	4.486078	6.802551	6.780796	6.023141667	1.331180625	3.172395	2.067281	1.039351	2.093009	1.066754716	2.877742841	1.52	1.58E-02
Ccne1	1.087166	1.372931	1.006685	1.155594	0.192472924	0.25949	0.346499	0.121277	0.242422	0.113576955	4.766869344	2.25	1.58E-02
Igtp	9.930124	19.773777	12.887138	14.197013	5.050861855	7.380785	5.07982	3.933703	5.464769333	1.755486562	2.59791624	1.38	1.59E-02
Tgfb1	8.612462	8.625794	8.650573	8.629609667	0.019339896	4.25825	3.551817	2.511274	3.440447	0.878796759	2.50828153	1.33	2.02E-02
Trib2	2.321892	3.084842	3.514729	2.973821	0.604118593	1.2147	1.234174	0.933278	1.127384	0.168382492	2.637806639	1.40	2.36E-02
Irgm1	21.17094	39.182648	33.632366	31.32865133	9.224192768	14.343912	15.053014	11.606832	13.66791933	1.819826134	2.292130248	1.20	3.95E-02
Pak2	17.107498	22.979727	25.33938	21.80886833	4.239003754	12.236254	11.637875	7.48056	10.451563	2.590300847	2.086660946	1.06	3.98E-02
Inka2	0.11915	0.391799	0.391544	0.300831	0.157340413	0.0001	0.069121	0.041347	0.036856	0.034728971	8.162334491	3.03	4.87E-02
Gstp1	98.116745	167.794693	143.894638	136.6020253	35.40678973	425.866394	614.861938	205.028778	415.25237	205.1226411	0.328961459	-1.60	1.60E-32
Socs2	1.435649	7.076928	4.341187	4.284588	2.821065362	9.44511	11.370255	7.391972	9.402445667	1.98948463	0.455688674	-1.13	4.86E-08
**(G) Cytokine-mediated signaling pathway**													

Gene name	MCD_8	MCD_6	MCD_5	Mean (MCD)	SD (MCD)	Control_1	Control_3	Control_2	Mean (Control)	SD (Control)	Ratio(MCD/Control)	Log2 ratio (MCD/Control)	p value
Mmp12	22.232662	37.03727	38.803802	32.69124467	9.100363859	0.102168	0.0001	0.052717	0.051661667	0.051042183	632.795006	9.31	8.56E-72
Ptprc	63.977993	84.923584	83.666969	77.52284867	11.74700415	9.921238	8.887871	6.109474	8.306194333	1.971331278	9.33313688	3.22	2.38E-53
Stap1	7.24446	7.892395	8.645535	7.927463333	0.7011955	0.364755	0.235671	0.273573	0.291333	0.066349325	27.21100367	4.77	2.09E-27
Il1rn	12.254638	12.866429	12.462228	12.527765	0.311116346	1.807272	1.407876	1.070917	1.428688333	0.368618416	8.768717927	3.13	6.53E-17
Syk	6.345252	7.957227	9.124543	7.809007333	1.395561327	1.439965	1.721636	0.866139	1.34258	0.435983552	5.816418637	2.54	8.03E-17
Trem2	3.313693	5.677338	8.707722	5.899584333	2.703873571	0.0001	0.044638	0.0001	0.014946	0.025714026	394.7266381	8.62	1.25E-16
Ccr2	7.090495	11.523571	13.944882	10.85298267	3.476049744	0.817567	0.777805	1.109865	0.901745667	0.181329801	12.03552517	3.59	2.09E-16
Krt8	59.893848	78.246857	88.916359	75.685688	14.67978972	17.999054	26.412764	9.384347	17.932055	8.514406205	4.220692386	2.08	4.25E-15
Ikbke	14.680729	19.675493	20.932983	18.429735	3.307053789	2.806845	4.314041	1.55882	2.893235333	1.37964059	6.369939834	2.67	1.61E-13
Cxcl10	15.928356	28.626062	18.634588	21.063002	6.688111118	3.475758	1.313396	1.103207	1.964120333	1.313328284	10.72388572	3.42	4.39E-12
Naip6	2.203096	3.657899	3.224239	3.028411333	0.746909833	0.75408	0.648903	0.316057	0.573013333	0.228660159	5.285062593	2.40	9.16E-12
Il10ra	4.465157	5.554547	4.908121	4.975941667	0.547852512	0.811799	0.947713	0.600722	0.786744667	0.174847014	6.324722464	2.66	1.25E-11
Csf2rb2	2.612265	4.138879	3.599563	3.450235667	0.774184452	0.768004	0.447162	0.230204	0.48179	0.27056706	7.161285346	2.84	2.78E-11
Cx3cr1	4.311779	3.838964	7.587977	5.24624	2.041736427	0.194496	0.292297	0.517835	0.334876	0.165821457	15.66621675	3.97	7.39E-11
Cxcr4	3.802295	4.674377	4.624149	4.366940333	0.489641683	0.367861	0.292926	0.352025	0.337604	0.039494145	12.93509654	3.69	3.91E-10
Csf2rb	5.079367	7.701582	7.973214	6.918054333	1.598131508	1.538668	1.420837	1.015963	1.325156	0.274173794	5.220558435	2.38	4.63E-10
Ccl6	33.713238	31.675461	36.657597	34.015432	2.504777573	7.176613	5.440707	5.450871	6.022730333	0.999304625	5.647842443	2.50	5.03E-10
Zbp1	3.805883	9.559266	10.588037	7.984395333	3.655074067	3.441186	2.665846	1.264411	2.457147667	1.103292163	3.249456857	1.70	1.80E-09
Tnfrsf11a	5.17039	4.467984	9.43121	6.356528	2.685813768	0.796195	1.047383	0.730147	0.857908333	0.167380021	7.409332388	2.89	1.83E-09
Il7r	2.532215	2.927172	3.294905	2.918097333	0.381425971	0.214248	0.38572	0.289069	0.296345667	0.085967285	9.846937754	3.30	1.51E-08
Oas2	1.812685	5.137228	6.568295	4.506069333	2.439821216	0.674519	0.558699	0.210717	0.481311667	0.241391121	9.362061312	3.23	3.35E-08
Cxcl1	9.992468	18.591221	15.771691	14.78512667	4.383448434	3.501148	4.993383	1.856167	3.450232667	1.569227624	4.285254965	2.10	1.01E-07
Naip5	1.902557	2.731992	3.480407	2.704985333	0.789271611	0.484983	0.831413	0.328738	0.548378	0.257263937	4.932702139	2.30	1.83E-07
Oas1a	2.919621	4.583645	4.606152	4.036472667	0.96728738	0.459011	0.32856	0.472343	0.419971333	0.079444694	9.611305216	3.26	2.73E-07
Oasl2	12.584593	15.351889	23.692099	17.209527	5.782065945	4.156762	3.37949	2.678174	3.404808667	0.739619088	5.054476972	2.34	4.00E-07
Spi1	6.620032	7.631251	6.473569	6.908284	0.630375931	1.47015	1.335361	1.164391	1.323300667	0.153235865	5.220494612	2.38	4.92E-07
Samhd1	24.825527	32.041122	32.535278	29.80064233	4.315654872	7.484969	7.858655	6.16728	7.170301333	0.888509512	4.156121333	2.06	9.71E-07
Pirb	2.036342	3.97736	3.790556	3.268086	1.070802921	0.689344	0.97674	0.459408	0.708497333	0.259197294	4.612700495	2.21	1.53E-06
Gpr35	1.34397	1.706721	3.935909	2.328866667	1.403508414	0.199482	0.515401	0.322856	0.345913	0.15921659	6.732521376	2.75	1.78E-06
Laptm5	29.207567	28.499283	35.655354	31.12073467	3.943031312	6.191492	6.955821	7.047289	6.731534	0.469920866	4.623126715	2.21	2.23E-06
Ccl4	3.25271	4.010615	1.543785	2.935703333	1.26359902	0.0001	0.0001	0.0001	0.0001	0	29357.03333	14.84	2.52E-06
Naip2	2.712556	4.452533	4.725002	3.963363667	1.091764491	0.951606	1.54478	0.646035	1.047473667	0.456977681	3.783735852	1.92	3.25E-06
Csf3r	1.735277	2.38251	2.118357	2.078714667	0.325432443	0.41673	0.27886	0.087197	0.260929	0.165496647	7.966591167	2.99	7.48E-06
Vrk2	7.728741	12.173872	11.488495	10.46370267	2.393208494	3.195937	4.302728	1.723268	3.073977667	1.294047539	3.403961838	1.77	7.62E-06
Oasl1	8.656121	9.804526	11.925508	10.12871833	1.658628452	1.954883	2.489015	2.08273	2.175542667	0.278899429	4.655720381	2.22	1.35E-05
Klf6	11.209281	17.571312	22.158117	16.97957	5.498351697	6.50269	5.491213	4.156482	5.383461667	1.176809567	3.154024502	1.66	2.19E-05
Ticam2	1.628714	1.887846	1.357173	1.624577667	0.265360679	0.15702	0.35287	0.052257	0.187382333	0.152589146	8.669855038	3.12	2.34E-05
Ccdc3	4.691354	4.045935	7.945193	5.560827333	2.08998592	0.453677	0.590044	1.008364	0.684028333	0.28904015	8.129527773	3.02	3.89E-05
Cxcl5	0.566373	0.37179	1.387416	0.775193	0.539053232	0.0001	0.0001	0.0001	0.0001	0	7751.93	12.92	4.35E-05
Axl	25.17271	27.388927	37.68438	30.08200567	6.676449361	6.885433	9.14217	8.014218	8.013940333	1.128368526	3.753709713	1.91	4.36E-05
Eda2r	0.834397	1.194124	2.406154	1.478225	0.823492593	0.254219	0.146913	0.192477	0.197869667	0.053855873	7.470700411	2.90	4.83E-05
Il10rb	9.76649	9.936423	15.240095	11.64766933	3.11229191	4.766718	5.687536	3.455005	4.636419667	1.121954504	2.512212045	1.33	6.68E-05
Stat1	19.947338	36.482204	28.182756	28.20409933	8.267453663	11.595238	12.004816	6.774473	10.12484233	2.908723008	2.785633436	1.48	7.41E-05
Parp14	12.581502	23.927311	23.030426	19.846413	6.307558922	7.840681	9.578133	4.501817	7.306877	2.579913965	2.716127971	1.44	8.34E-05
Fcer1g	54.785313	52.94714	81.129715	62.954056	15.76739215	13.963125	12.259938	15.892587	14.03855	1.817498662	4.484370252	2.16	9.21E-05
Oas1g	2.398165	2.969632	2.493261	2.620352667	0.30619904	0.393441	0.435047	0.352689	0.393725667	0.041179738	6.655275204	2.73	9.92E-05
Csf2ra	2.184931	7.431961	9.116294	6.244395333	3.615063266	0.959142	1.141866	0.781993	0.961000333	0.179943697	6.497807666	2.70	1.03E-04
Ccl3	2.980034	3.846183	2.176305	3.000840667	0.835133416	0.0001	0.282802	0.0001	0.094334	0.163218076	31.81080699	4.99	1.05E-04
Il17ra	3.298273	6.238379	5.028948	4.8552	1.477733771	1.929725	1.530479	0.767061	1.409088333	0.590761089	3.445632105	1.78	1.22E-04
Oas3	4.125302	4.325319	6.422542	4.957721	1.272508201	0.698692	0.455583	1.072583	0.742286	0.310801508	6.678990308	2.74	1.48E-04
Csf1	7.173495	9.56284	10.742694	9.159676333	1.818433689	2.645988	2.301589	1.963312	2.303629667	0.341342575	3.976193077	1.99	1.55E-04
Traf1	2.083493	1.16752	1.834118	1.695043667	0.473558747	0.179619	0.157265	0.233001	0.189961667	0.038912896	8.923082727	3.16	1.80E-04
Numbl	0.620908	0.822763	1.286725	0.910132	0.341398705	0.153853	0.093125	0.162767	0.136581667	0.037897576	6.663646902	2.74	1.97E-04
Osmr	2.417598	5.07743	5.666309	4.387112333	1.730876845	1.321164	1.242254	1.109822	1.224413333	0.106794556	3.583032146	1.84	2.30E-04
Il1b	3.087222	3.857042	3.995502	3.646588667	0.489347621	0.618111	0.993867	0.429184	0.680387333	0.28744648	5.359577535	2.42	2.37E-04
Ccl2	2.694071	6.763049	3.522325	4.326481667	2.150382861	0.707446	0.0001	0.254816	0.320787333	0.358257943	13.48707139	3.75	2.81E-04
Isg15	5.679106	11.844505	13.93393	10.485847	4.291851988	3.354634	2.184766	1.265491	2.268297	1.047073393	4.622783965	2.21	2.96E-04
Ccr1	0.604507	0.882695	1.751155	1.079452333	0.598109936	0.078895	0.082626	0.117424	0.092981667	0.021249726	11.60930291	3.54	3.86E-04
Krt18	45.209412	69.744835	72.748161	62.56746933	15.10733622	23.147333	37.330559	12.129572	24.202488	12.63358423	2.585166836	1.37	4.05E-04
Ccl22	0.826083	1.946121	3.413007	2.061737	1.297331584	0.2679	0.0001	0.182427	0.150142333	0.136787919	13.7318833	3.78	5.49E-04
Duox2	0.145523	0.097683	0.330126	0.191110667	0.122744098	0.0001	0.0001	0.0001	0.0001	0	1911.106667	10.90	5.50E-04
Cd300lf	1.378338	1.650694	2.08636	1.705130667	0.35713625	0.158773	0.534804	0.216918	0.303498333	0.202415276	5.618253807	2.49	7.90E-04
Casp4	2.398894	5.968969	4.457874	4.275245667	1.79203061	0.746511	1.25659	0.790885	0.931328667	0.282557011	4.590480053	2.20	8.15E-04
Irf7	25.143126	28.023054	25.486742	26.21764067	1.572944998	10.230639	8.802448	6.607296	8.546794333	1.825150055	3.06754084	1.62	1.09E-03
Wnk1	57.629589	86.317635	92.139786	78.69567	18.47455513	42.045624	42.397697	23.62455	36.02262367	10.73848974	2.184617943	1.13	1.80E-03
Ifngr1	12.825019	19.523911	22.495457	18.28146233	4.953493898	8.135872	8.582085	5.356567	7.358174667	1.747741814	2.484510515	1.31	1.80E-03
Adam17	10.066874	14.72175	15.704633	13.49775233	3.011595815	6.851346	7.296618	3.711001	5.952988333	1.954340603	2.267391027	1.18	1.90E-03
Ereg	0.628018	0.874722	1.621275	1.041338333	0.517165949	0.174981	0.06827	0.171263	0.138171333	0.060564868	7.53657295	2.91	2.15E-03
Traf3	4.075067	6.107934	7.910964	6.031321667	1.91909576	2.202983	2.130257	1.03179	1.788343333	0.656202695	3.372574804	1.75	2.25E-03
Aim2	2.687448	3.868723	1.997005	2.851058667	0.946524382	0.43896	0.731736	0.342131	0.504275667	0.202848752	5.653770061	2.50	2.28E-03
Stk39	2.404419	4.108631	5.784153	4.099067667	1.689887295	0.888197	1.624945	0.72024	1.077794	0.481230767	3.803201416	1.93	2.63E-03
Pycard	1.497864	1.693408	1.958261	1.716511	0.231066356	0.4599	0.467403	0.396043	0.441115333	0.039213648	3.891297514	1.96	2.70E-03
Trim6	0.375757	0.915231	1.46873	0.919906	0.546501497	0.132233	0.235735	0.078887	0.148951667	0.079749352	6.17586913	2.63	3.19E-03
Il21r	1.719503	1.736363	1.766342	1.740736	0.023723729	0.0001	0.339512	0.243578	0.194396667	0.174969223	8.954556834	3.16	3.42E-03
Tnf	1.588277	0.836077	0.897892	1.107415333	0.4175838	0.0001	0.0001	0.138356	0.046185333	0.079822139	23.97764081	4.58	3.61E-03
Cxcl2	0.663378	0.872314	0.797186	0.777626	0.105832454	0.031664	0.17666	0.02688	0.078401333	0.085128114	9.918530297	3.31	4.94E-03
Ccl5	16.810623	29.33164	8.743227	18.29516333	10.37417864	2.201626	1.681839	4.694987	2.859484	1.61069801	6.398064593	2.68	6.44E-03
Adar	7.684996	13.304723	11.584495	10.85807133	2.879427269	5.248053	5.558695	2.130342	4.312363333	1.896058395	2.517893437	1.33	6.83E-03
Acsl1	87.225014	158.849152	119.153648	121.7426047	35.88218651	274.578796	293.989746	174.772171	247.7802377	63.9674104	0.491332989	-1.03	2.78E-29
